# Aldehyde dehydrogenase superfamily in sorghum: genome-wide identification, evolution, and transcript profiling during development stages and stress conditions

**DOI:** 10.1186/s12870-022-03708-4

**Published:** 2022-07-04

**Authors:** Md. Sifatul Islam, Munira Mohtasim, Tahmina Islam, Ajit Ghosh

**Affiliations:** 1 Department of Biochemistry and Molecular Biology, Shahjalal University of Science and Technology, Sylhet, 3114 Bangladesh; 2 Plant Breeding and Biotechnology Laboratory, Department of Botany, University of Dhaka, Dhaka, 1000 Bangladesh

**Keywords:** Aldehyde dehydrogenases, *Sorghum bicolor*, Gene duplication, Evolution, Abiotic stress, Stress adaptation, Promoter, Protein modelling

## Abstract

**Background:**

Aldehyde dehydrogenases (ALDHs) are a family of NAD(P)^+^ dependent enzymes that detoxify aldehydes by promoting their oxidation to respective carboxylic acids. The role of ALDH enzymes in various plant species has been extensively studied, revealing their critical role in salinity, drought, heat, and heavy metal stress tolerance. Despite their physiological significance, *ALDH* genes in *Sorghum bicolor* have yet to be studied thoroughly.

**Results:**

In this study, a total of 19 *ALDH* genes have been identified that have been grouped into ten families based on the criteria of the *ALDH* gene nomenclature committee. Segmental duplication assisted more in the enhancement of *SbALDH* gene family members than tandem duplication. All the identified SbALDH members made a cluster with monocot rice and maize in the phylogenetic tree rather than dicot species, suggesting the pre-eudicot-monocot separation of the ALDH superfamily members. The gene structure and protein domain were found to be mostly conserved in separate phylogenetic classes, indicating that each family played an important role in evolution. Expression analysis revealed that several *SbALDH*s were expressed in various tissues, developmental stages, and in response to abiotic stresses, indicating that they can play roles in plant growth, development, or stress adaptation. Interestingly, the majority of the *SbALDH* genes were found to be highly responsive to drought stress, and the *SbALDH*18B1 transcript showed maximum enhancement in all the stress conditions. The presence of cis-acting elements (mainly ABRE and MBS) in the promoter region of these genes might have a significant role in drought tolerance.

**Conclusions:**

Our findings add to the current understanding, evolutionary history, and contribution of SbALDHs in stress tolerance, and smooth the path of further functional validation of these genes.

**Supplementary Information:**

The online version contains supplementary material available at 10.1186/s12870-022-03708-4.

## Background

Endogenous aldehydes are common mediators in a variety of metabolic processes, including the metabolism of amino acids, proteins, lipids, and carbohydrates [1]. Environmental stress conditions such as dehydration, salinity, cold, and extreme temperature often cause them to be generated excessively [2]. Because of their chemical reactivity, these aldehydes may have harmful effects on cellular metabolism when formed in large quantities that can adversely affect cell growth, seed viability, and ultimate yield [3, 4]. Thus, to ensure normal developmental growth processes, aldehyde levels in cells must be controlled. The carbonyl group of reactive aldehydes is either reduced to alcohol or oxidized to the corresponding carboxylic acid to detoxify them [5, 6]. Aldehyde dehydrogenases (ALDHs, EC: 1.2.1.3), also known as "aldehyde scavengers", represent a broad family of NAD(P)^+^ dependent enzymes that can irreversibly oxidize a wide range of aromatic and aliphatic aldehydes to their respective carboxylic acids [1, 7]. In addition, ALDHs also have a variety of other roles such as, (i) involving in secondary metabolism, especially, amino acid and retinoic acid [8]; (ii) generating osmoprotectant, such as glycine betaine [9, 10]; and (iii) generating NAD(P)H to maintain redox homeostasis [11].

ALDHs are multiform enzymes with various amino acid sequences that contain distinct motifs, such as cysteine active site (PS00070), glutamic acid active site (PS00687), and the Rossmann fold [12, 13]. ALDHs can be divided into 24 families across all taxa, according to the criteria defined by the ALDH Gene Nomenclature Committee (AGNC) [14]. ALDH proteins of fourteen different families are found in plants of which, the families ALDH11, ALDH12, ALDH19, ALH21, ALDH22, ALDH23, and ALDH24 are only plant-specific whereas the rest of the families were also found in human [6]. However, the ALDH19 family member has been identified only in *Solanum lycopersicum*, which is believed to encode γ-glutamyl phosphate reductase involved in the biosynthesis of proline from glutamate [15] and the ALDH24 gene family is considered to be precise to *Chlamydomonas reinhardtii* [16].

Previous investigations have analyzed the possible roles of certain *ALDHs* in plants and these *ALDHs* have been discovered to react to a variety of abiotic stresses, including dehydration, high temperature, salt stress, and oxidative stress, implying that plant *ALDHs* can play a major role in stress tolerance [17, 18]. Overexpression of the *Arabidopsis ALDH* genes increased their resistance to a range of environmental stresses [19, 20]. Upregulation of the *AtALDH3I1* and *AtALDH7B4* genes from *Arabidopsis* showed increased resistance to osmotic and oxidative stresses [2] as well as, the encoded proteins from these genes inhibit the lipid peroxidation and scavenge ROS [20]. Overexpression of the *ALDH22A1* gene in maize resulted in enhanced stress tolerance as well as a reduction in MDA content produced by lipid peroxidation [21]. Suppression of the *ALDH2C4* gene in *Nicotiana benthamiana* resulted in more vulnerability against low-temperature stress and stored more ROS and MDA [22]. Ectopic expression of wheat *TraeALDH7B1-5A* gene into Arabidopsis resulted in considerable drought resistance [23]. Likewise, transgenic tobacco seedlings overexpressing the *Brassica BrALDH7B2* gene conferred salinity and drought resistance [24]. Some plant *ALDH* genes have also been reported in regulating or affecting plant growth and development [25].

The completion of genome sequencing for a growing number of plant species has allowed for the identification and analysis of further *ALDHs*. *Sorghum bicolor* is the world’s fifth most vital cereal crop that has been considered the second most essential food grain in the semi-arid region [26]. Sorghum is a gluten-free alternative to staple grains and a potential biofuel feedstock that is commonly cultivated for bread, feed, and forage [27, 28]. It has ten chromosomes with a genome size of approximately 730 Mb [29, 30]. Because of its ideal characteristics, such as high biomass yields, rapid growth, the C4 photosynthesis pathway, stress resistance, and, not least, its small genome size, sorghum has piqued the scientific community’s attention as a model plant for the study of bioenergy crops [31, 32]. Even though the *ALDH* gene superfamily has been predicted in *Sorghum bicolor* [33], relying on the genome v1.0 [30], comprehensive expression and functional analysis are not performed yet.

Thus, it would be fascinating to investigate the presence, distribution, and expression profiling of *ALDH* genes in sorghum, because of their essential role in stress adaptation. In the current study, a systematic *in-silico* analysis of sorghum *ALDH* genes, which included evolutionary relationships, gene structure, cis-regulatory elements, duplication events, and protein structure, was conducted to put the *ALDH* gene family in sorghum into proper perspective. The extensive-expression profiling of different *SbALDH* genes was also investigated in sorghum under various abiotic stress conditions, developmental stages, and anatomical tissues. The findings of this research lay the groundwork for further functional analysis of *ALDH* genes in sorghum and other plant species, as well as provide new target genes for enhancing sorghum stress resistance genetically.

## Results

### Characteristics of the ALDH superfamily in *S. bicolor*

**Fig. 1:**
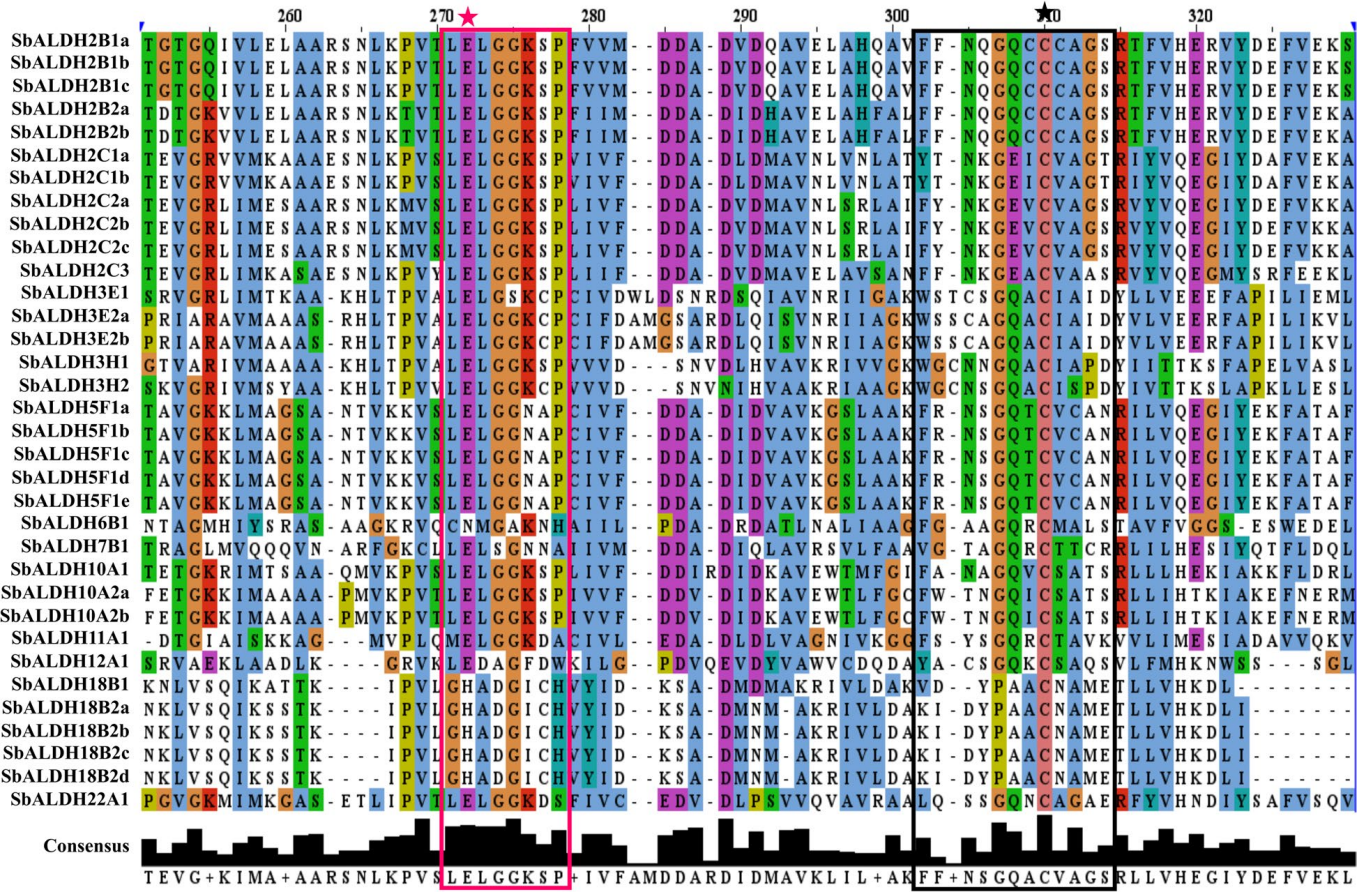
Multiple sequence alignment of the ALDH domains of all the identified SbALDH proteins. The figure was generated by using the Jalview program (https://www.jalview.org/) for multiple sequence alignment editing, visualization and analysis. The conserved motif and active site of glutamic acid residue were marked by a pink-coloured box and star, while the conserved motif and active site of cysteine residue were marked by a black coloured box and star, respectively

HMM profile analysis along with BLASTP search yielded a total of 34 SbALDH proteins encoded by 19 genes which indicting the presence of alternate splicing. With Pfam and NCBI Conserved Domain Database search, the presence of the conserved ALDH domain (PF00171) was confirmed. ScanProsite and multiple sequence alignment analysis revealed that 16 out of 19 *SbALDH* genes encode a protein that contains both the ALDH cysteine (PS00070) and glutamic acid (PS00687) active sites. Interestingly, all the 34 SbALDH protein comprises the cysteine active site but the glutamic acid active site is absent in the SbALDH6 and SbALH18 members. The lack of a catalytic glutamic acid residue in ALDH6 and ALH18 family proteins is related to their activity as Coenzyme A (CoA) dependent acylating and Δ-1-pyrroline-5-carboxylate synthetases, respectively [34] (Fig. 1). According to the AGNC guideline, all the identified SbALDH members were divided into ten families (ALDH- 2, 3, 5, 6, 7, 10, 11, 12, 18, and 22). ALDH2 constituted the largest family in *S. bicolor* with five members, followed by ALDH3 which comprised four members (Table 1). The SbALDH proteins are ranging from 391 to 729 amino acids (aa) in length, with an estimated isoelectric point (pI) ranging from 4.85 to 9.45. SbALDH proteins range in molecular weight (MW) from 41.50 kDa to 78.36 kDa. The average length, pI, and MW of the identified SbALDH proteins were found to be 529 aa, 57.22, and 6.59 kDa, respectively. The chloroplast was predicted to be the center of localizing for most of the SbALDH proteins, followed by mitochondria, peroxisome, and cytoplasm (Table 1).

**Table 1:** Detailed information of the newly identified SbALDH members including their subfamilies, structural arrangements, and subcellular localization

**Family**	**Locus ID**	**Transcript ID**	**Annotation**	**Coordinate** **(5’ to 3’)**	**Length**			**Conserved domain/sites**			**MW (kDa)**	**pI**	**Localization**
					**Transcript** **(nt)**	**CDS** **(nt)**	**Protein** **(aa)**	**PF00171** **start-stop**	**PS00687** **yes/no**	**PS00070 yes/no**			
2	Sobic.004G250900	Sobic.004G250900.2	SbALDH2B1a	59,721,748–59,725,595	2967	1272	423	1–413	Yes	Yes	45.67	6.38	Mitochondrion
		Sobic.004G250900.3	SbALDH2B1b	59,720,135–59,725,595	3507	1656	551	79–541	Yes	Yes	58.79	6.65	Mitochondrion
		Sobic.004G250900.4	SbALDH2B1c	59,720,879–59,725,595	3246	1656	551	79–541	Yes	Yes	58.79	6.65	Mitochondrion
	Sobic.010G113000	Sobic.010G113000.1	SbALDH2B2a	11,756,541–11,766,808	2429	1671	556	84–546	Yes	Yes	60.09	7.20	Mitochondrion
		Sobic.010G113000.2	SbALDH2B2b	11,759,710–11,766,808	2630	1644	547	75–537	Yes	Yes	59.21	6.65	Mitochondrion
	Sobic.003G203500	Sobic.003G203500.1	SbALDH2C1a	53,331,225–53,339,384	1971	1506	501	29–491	Yes	Yes	54.33	5.99	Chloroplast
		Sobic.003G203500.2	SbALDH2C1b	53,331,225–53,339,384	2060	1272	423	29–416	Yes	Yes	45.73	5.84	Chloroplast
	Sobic.003G203600	Sobic.003G203600.1	SbALDH2C2a	53,368,965–53,376,601	2043	1515	504	32–494	Yes	Yes	54.24	5.47	Chloroplast
		Sobic.003G203600.2	SbALDH2C2b	53,368,965–53,376,625	2264	1272	423	1–413	Yes	Yes	45.70	5.34	Chloroplast
		Sobic.003G203600.3	SbALDH2C2c	53,374,514–53,376,601	1570	1245	414	1–404	Yes	Yes	44.65	5.23	Chloroplast
	Sobic.010G178300	Sobic.010G178300.1	SbALDH2C3	51,625,698–51,631,645	2164	1566	521	45–511	Yes	Yes	56.26	5.78	Chloroplast
3	Sobic.004G300800	Sobic.004G300800.1	SbALDH3E1	63,955,597–63,960,188	1971	1461	486	1–442	Yes	Yes	54.19	6.59	Chloroplast
	Sobic.006G163300	Sobic.006G163300.2	SbALDH3E2a	52,091,479–52,098,020	2119	1491	496	24–452	Yes	Yes	54.56	8.64	Chloroplast
		Sobic.006G163300.3	SbALDH3E2b	52,091,484–52,097,728	1741	1491	496	24–452	Yes	Yes	54.56	8.64	Chloroplast
	Sobic.005G064800	Sobic.005G064800.1	SbALDH3H1	7,268,869–7,273,754	2377	1437	478	1–431	Yes	Yes	52.05	8.07	Chloroplast
	Sobic.008G057500	Sobic.008G057500.1	SbALDH3H2	5,994,822–6,001,133	1889	1464	487	1–437	Yes	Yes	52.42	9.45	Chloroplast
5	Sobic.004G058600	Sobic.004G058600.1	SbALDH5F1a	4,685,051–4,695,810	2412	1584	527	62–521	Yes	Yes	56.01	8.31	Mitochondrion
		Sobic.004G058600.2	SbALDH5F1b	4,685,097–4,695,810	2075	1551	516	62–494	Yes	Yes	54.92	8.25	Mitochondrion
		Sobic.004G058600.3	SbALDH5F1c	4,685,092–4,695,810	2261	1482	493	28–487	Yes	Yes	52.40	6.22	Mitochondrion
		Sobic.004G058600.4	SbALDH5F1d	4,687,581–4,695,576	1953	1176	391	1–385	Yes	Yes	41.50	5.70	Mitochondrion
		Sobic.004G058600.5	SbALDH5F1e	4,685,097–4,695,810	2108	1584	527	62–521	Yes	Yes	56.01	8.31	Mitochondrion
6	Sobic.002G062500	Sobic.002G062500.1	SbALDH6B1	6,035,846–6,043,359	2684	1623	540	55–519	No	Yes	57.84	5.89	Mitochondrion
7	Sobic.002G215700	Sobic.002G215700.1	SbALDH7B1	60,779,264–60,786,533	2031	1530	509	30–492	Yes	Yes	54.40	6.06	Mitochondrion
10	Sobic.006G109500	Sobic.006G109500.1	SbALDH10A1	47,850,314–47,855,384	2070	1521	506	18–488	Yes	Yes	55.06	5.94	Chloroplast, Mitochondrion, Peroxisome
	Sobic.007G130800	Sobic.007G130800.1	SbALDH10A2a	54,746,028–54,751,628	1731	1518	505	18–487	Yes	Yes	54.91	5.16	
		Sobic.007G130800.2	SbALDH10A2b	54,745,913–54,751,628	1976	1182	393	1–375	Yes	Yes	42.75	4.85	
11	Sobic.007G140700	Sobic.007G140700.1	SbALDH11A1	56,977,075–56,981,706	2088	1497	498	26–486	Yes	Yes	53.28	6.80	Cytoplasm
12	Sobic.009G212600	Sobic.009G212600.1	SbALDH12A1	55,816,131–55,822,788	2152	1650	549	55–510	Yes	Yes	60.58	6.90	Mitochondrion
18	Sobic.003G356000	Sobic.003G356000.1	SbALDH18B1	67,427,644–67,434,548	2783	2190	729	295–584	No	Yes	78.36	5.99	Cytoplasm
	Sobic.009G160100	Sobic.009G160100.1	SbALDH18B2a	51,779,954–51,800,133	2804	2151	716	288–570	No	Yes	77.71	5.92	Cytoplasm
		Sobic.009G160100.2	SbALDH18B2b	51,779,954–51,800,133	2800	2151	716	288–570	No	Yes	77.71	5.92	Cytoplasm
		Sobic.009G160100.3	SbALDH18B2c	51,779,375–51,787,129	3460	2151	716	288–570	No	Yes	77.71	5.92	Cytoplasm
		Sobic.009G160100.4	SbALDH18B2d	51,779,375–51,787,112	3447	2151	716	288–570	No	Yes	77.71	5.92	Cytoplasm
22	Sobic.002G426100	Sobic.002G426100.1	SbALDH22A1	77,251,658–77,257,367	2414	1782	593	52–521	Yes	Yes	65.45	7.48	Chloroplast

*Abbreviations: CDS* Coding DNA Sequence, *Chr* Chromosome number, *MW* Molecular Weight, *pI* Isoelectric point, *nt* nucleotide, *aa* amino acid, *kDa* kilodalton, *Cp* Chloroplast, *Cy* Cytoplasm, *Mt* Mitochondria, *Vc* Vacuolar, *Pm* Plasma-membrane

Localization prediction by the Plant-mPLoc server (http://www.csbio.sjtu.edu.cn/bioinf/plant-multi/)

### Analysis of the chromosomal distribution and duplication events of *SbALDH* genes

The *SbALDH* genes were found to be distributed unevenly across 9 of the 10 Sorghum chromosomes (Fig. S1). With three genes chromosomes 2, 3, and 4 comprised the largest number of *ALDHs*, followed by chromosomes 6, 7, 9, and 10 with two *ALDH* genes each. Contrastingly, chromosomes 5 and 8 carry a single gene, while chromosome 1 has no *ALDH* gene (Table 1). Gene duplication and divergence are critical steps in the plant genome for the extension of gene families and the development of new functions. Two of the most common causes of gene family expansion are segmental and tandem duplications [35]. Segmental duplication blocks in the sorghum genome have discovered five pairs of *SbALDH* genes: *SbALDH2B1|SbALDH2B2, SbALDH3E1|SbALDH3E2, SbALDH3H1|SbALDH3H2, SbALDH10A1|SbALDH10A2,* and *SbALDH18B1|SbALDH18B2.* A tandem duplication event between *SbALDH2C1* and *SbALDH2C2* was also identified (Table 2)*.* All the duplicated *SbALDH* gene pairs had a Ka/Ks value of less than 0.3, except *SbALDH2B1*|*SbALDH2B2*, indicating the role of purifying selection in their evolution. Furthermore, the approximate divergence period of the duplicated *SbALDH* gene pairs ranges from 25.73 (*SbALDH3E1|SbALDH3E2*) to 84.31 (*SbALDH2C1*|*SbALDH2C2*) million years (Table 2).

**Table 2:** Gene duplication analysis of *SbALDH* genes

Sl no	Locus 1	Locus 2	Ka	Ks	Ka/Ks	Duplication time (Mya)	Duplication type
1	*SbALDH2B1*	*SbALDH2B2*	0	0	∞	Not determinable	Segmental
2	*SbALDH2C1*	*SbALDH2C2*	0.1664	2.5295	0.0657	84.31	Tandem
3	*SbALDH3E1*	*SbALDH3E2*	0.228	0.7719	0.2953	25.73	Segmental
4	*SbALDH3H1*	*SbALDH3H2*	0.2294	1.5649	0.1465	52.16	Segmental
5	*SbALDH10A1*	*SbALDH10A2*	0.1584	1.0913	0.1451	36.37	Segmental
6	*SbALDH18B1*	*SbALDH18B2*	0.1399	1.0607	0.1318	35.35	Segmental

**Fig. 2:**
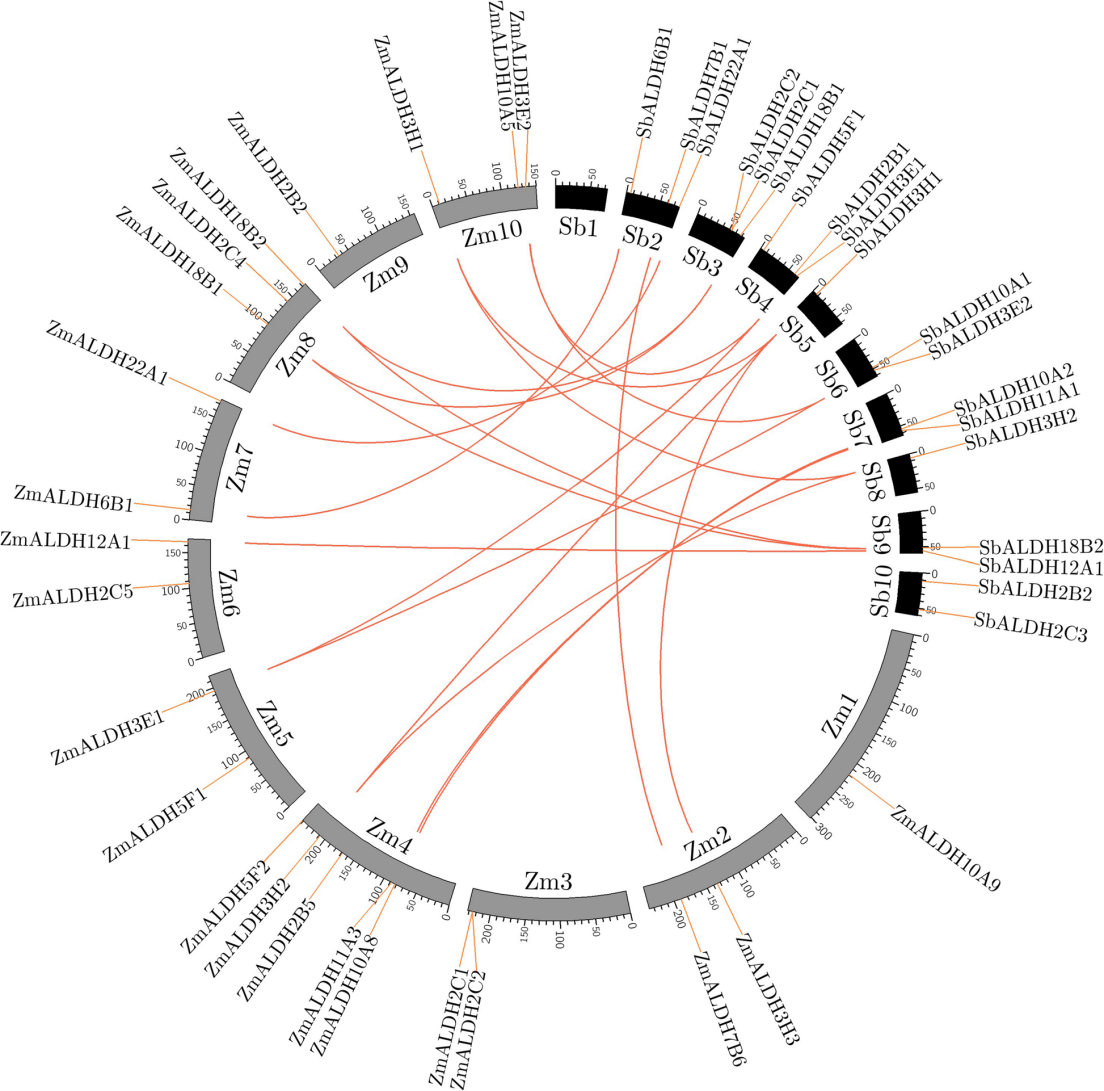
Assessment of the ALDH gene duplication between sorghum and maize. The chromosomes of sorghum and maize are shown as black and grey coloured boxes, respectively. Vertical red lines outside the circles denote ALDH genes. Duplication links were indicated by red lines connecting the chromosomes of sorghum and maize. The figure was created using Circos software (http://circos.ca/)

A relatively simple approach for determining the origin, ancestral history, and function of a gene is to compare the genomes from different species [36]. We studied a comparative duplication map of the sorghum and maize genomes to learn more about the origin and evolution of *SbALDH* (Fig. 2). The species sorghum and maize are closely related as they belong to the same *Panicoideae* subfamily of the *Gramineae* family. Our duplication analysis revealed five duplicated genes among sorghum to maize: *SbALDH6B1*-*ZmALDH6B1*, *SbALDH7B1*-*ZmALDH7B6*, *SbALDH10A2*-*ZmALDH10A8*, *SbALDH11A1*-*ZmALDH11A3*, and *SbALDH12A1*-*ZmALDH12A1.* This suggests that these gene families may have been present in the genome of the sorghum and maize’s last common ancestor*.* Cases in which duplicated sorghum genes corresponded to two or more maize genes were more difficult to interpret in syntenic manner and these correspondences includes *SbALDH3E1*-*ZmALDH3E1*|*ZmALDH3E2*, *SbALDH3E2*-*ZmALDH3E1*|*ZmALDH3E2*, *SbALDH3H1*-*ZmALDH3H1*|*ZmALDH3H2*|*ZmALDH3H3*, *SbALDH3H2*-*ZmALDH3H2*|*ZmALDH3H1*, *SbALDH6B1*-*ZmALDH6B1*, *SbALDH7B1*-*ZmALDH7B6*, *SbALDH10A2*-*ZmALDH10A8*, *SbALDH11A1*-*ZmALDH11A3*, *SbALDH12A1*-*ZmALDH12A1*, *SbALDH18B1*-*ZmALDH18B2*|*ZmALDH18B1*, *SbALDH18B2*-*ZmALDH18B1*|*ZmALDH18B2*, and *SbALDH22A1*|*ZmALDH22A1* (Table S1).

### Distribution and evolution analysis of the ALDH superfamily

**Fig. 3:**
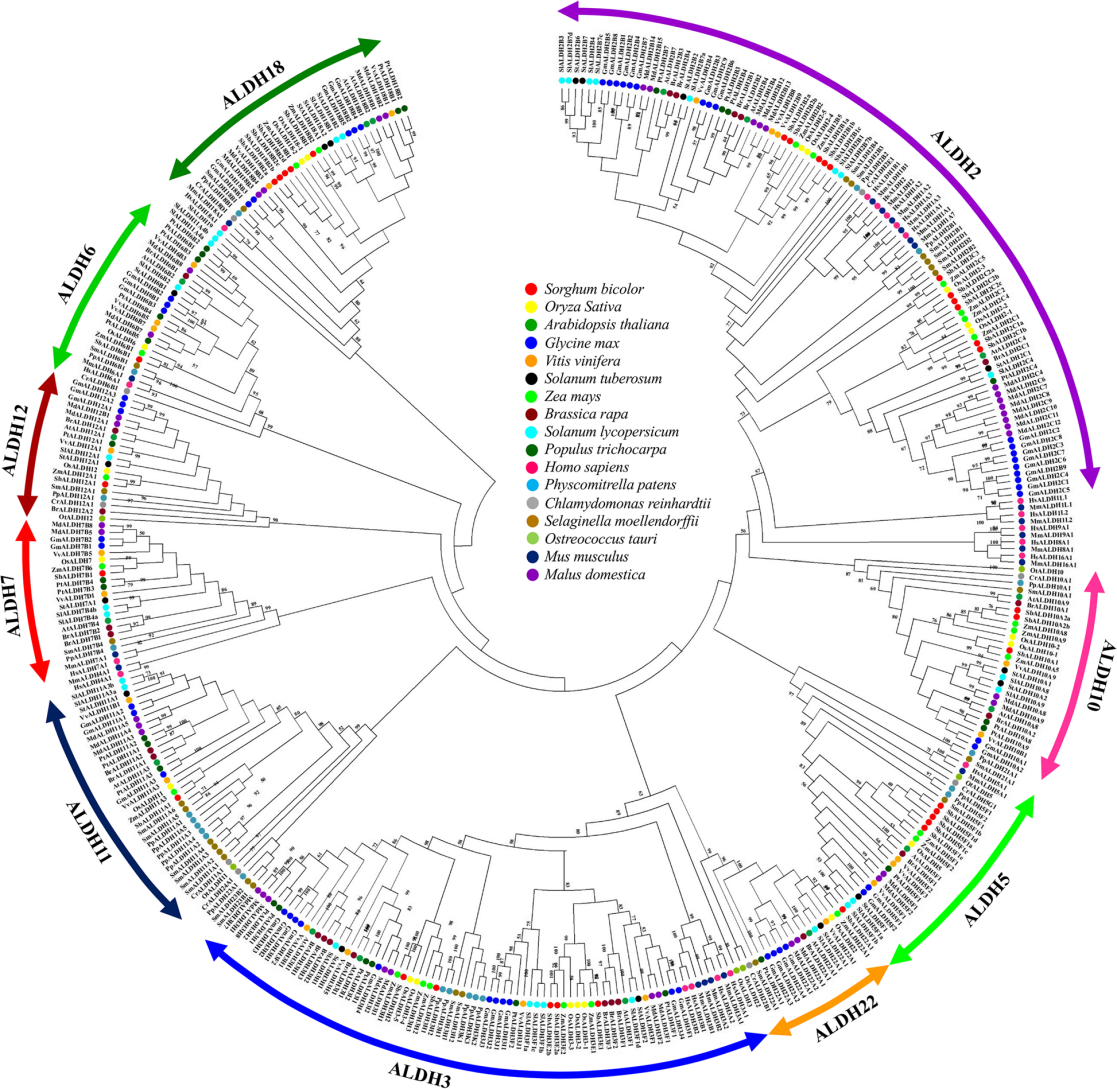
Phylogenetic analysis of ALDH superfamily members from different species. A Maximum-likelihood tree was constructed using the MEGA-X software (https://www.megasoftware.net/) with 402 full-length ALDH protein sequences from 17 different species. Different coloured circle in the tree represents ALDH proteins from various species

To scrutinize the evolutionary history of the sorghum *ALDH* gene superfamily, a maximum likelihood phylogenetic tree was constructed (Fig. 3). The tree was generated using the multiple sequence alignment of 402 ALDH protein sequences from 17 different species, including three monocot species (sorghum, rice, and maize), eight eudicot species (Arabidopsis, apple, grape, mustard, soybean, black cottonwood, potato, and tomato), four lower plant species (unicellular green algae, marine green algae, moss, and Gemmiferous Spike moss), and two mammals (human, and mouse). Investigation reveals that SbALDHs are more closely related to the monocot plants- rice and maize than those from other species in the tree. This finding additionally manifests that ALDH proteins belonging to the same families tended to cluster together and the whole tree can be classified into ten major families (ALDH-2, 3, 5, 6, 7, 10, 11, 12, 18, and 22). The tree also made it clear that ALDH2 is the most enormous family, followed by ALDH3. The ALDH18 family is found to be the most distantly related one among the selected organisms. Furthermore, some families such as ALDH-1, 4, 8, 9, and 16 are unique to animal species that make minor clusters within their members. Similarly, ALDH-21, 23, and 24 were only found in lower plant species and members of them from different species tend to tuft together.

### The *ALDH* gene family has evolved at a molecular level between sorghum and maize/rice

**Fig. 4:**
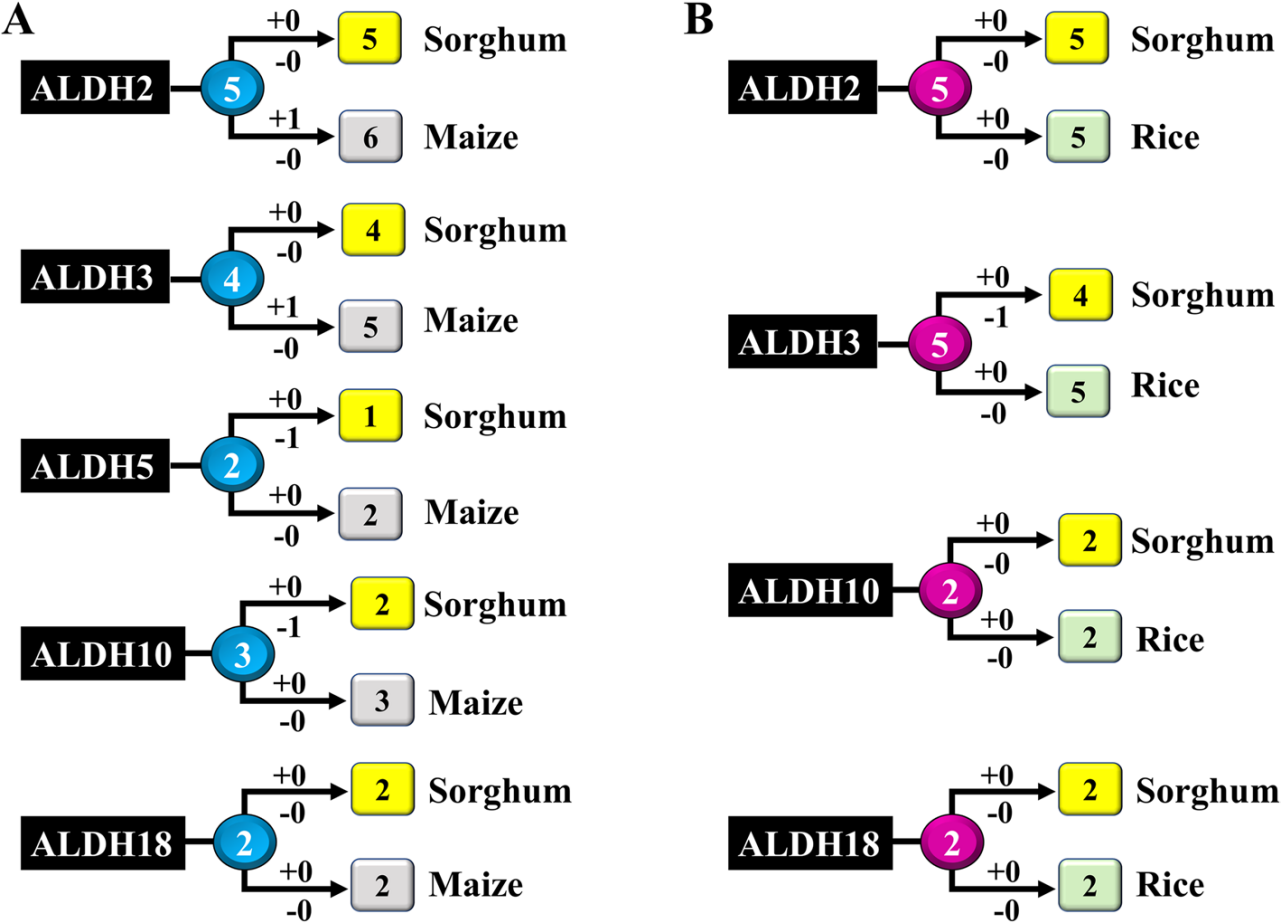
Expansion of ALDH gene family in different species. Changes in the copy number of the *ALDH* genes in **A** sorghum and maize, as well as **B** sorghum and rice, were analyzed. The values in circles and rounded squares show the number of ALDH genes in ancestral and extant species, respectively. Numbers with plus and minus symbols on branches refer to the number of gene expansions and losses, respectively

Evolutionary analysis was conducted using the ALDH protein sequences from sorghum, maize, and rice to explore the lineage-specific expansion of ALDH members in sorghum and maize, as well as in sorghum and rice genome (Fig. 4). ALDH proteins from these three plant species (sorghum, maize, and genome) are distributed among ten specific families. In our analysis between sorghum and maize, the ALDH family- 2, 3, 5, 10, and 18 were considered as other families containing only one member. While ALDH family- 2, 3, 10, and 18 were considered in the analysis between sorghum and rice for the same reason. Certain ALDH members were probably found in both sorghum and maize as well as in both sorghum and rice as the most recent common ancestor (MRCA), but some members could be later extincted or gained in some species. In the MRCA of sorghum and maize, there were at least five ancestral ALDH2 (Fig. S2). Maize obtained one gene after splitting and lost no gene, leading to six family *ALDH2* genes, while no gain or loss was observed in the sorghum (Fig. 4A). For ALDH3, there were four MRCA between sorghum and maize (Fig. S2). During evolution, maize acquired one gene without any loss and sorghum had no gain or loss of the gene, resulting in four sorghum and five maize *ALDH3* genes. For *ALDH*5, there were two MRCA genes, where sorghum lost one gene to remain with one gene, while maize had two genes as in the MRCA. For *ALDH*10, sorghum lost one gene from the MRCA resulting in two genes, where maize had no gain or loss of the gene. For *ALDH*18, sorghum and maize had two MRCA, after splitting they had maintained the same number of genes. On the contrary, there was no gain or loss of the *ALDH* gene numbers between sorghum and rice, except the *ALDH*3 (Fig. 4B). There were at least five MRCA genes between the sorghum and rice *ALDH3* family (Fig. S3). After the split, sorghum lost one gene leading to four *ALDH3* genes, while rice has five *ALDH3* genes without any gain or loss (Fig. 4B).

### Exon–intron organization, and domain architecture analysis of *SbALDH* members

**Fig. 5:**
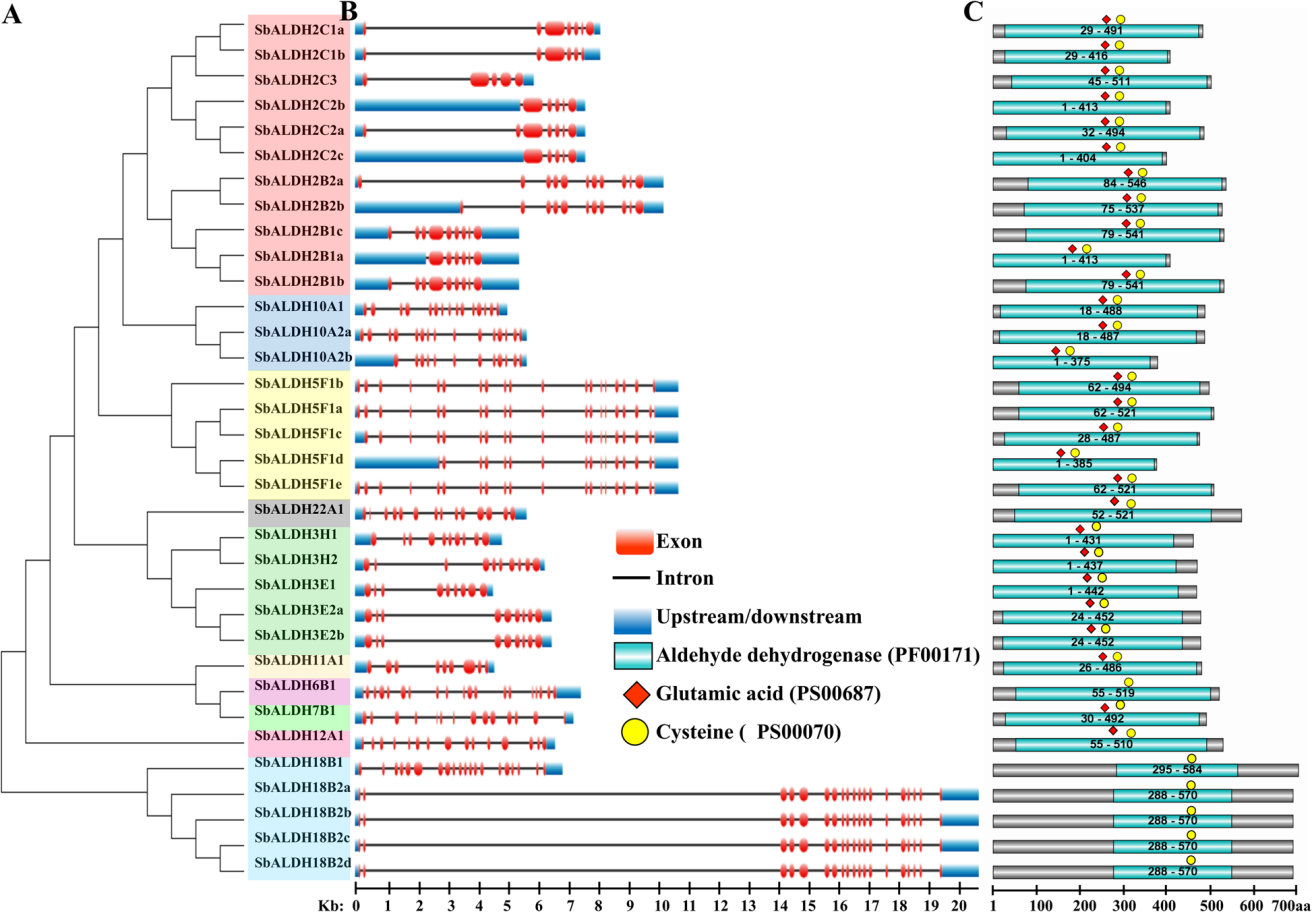
The ALDH superfamily in sorghum. **A** Phylogenetic analysis of the identified Sb ALDH members along with their gene structure in terms of exon–intron pattern; rounded rectangle, black line, and blue rectangle represent exon, intron, and upstream/downstream, respectively. **B** The distribution of conserved ALDH domain in SbALDH proteins; sky blue rectangle, red diamond, and yellow circle illustrate the ALDH domain (PF00171), glutamic acid (PS00687) active site, and cysteine (PS00070) active site, respectively

The amino acid sequences of the 34 SbALDH proteins were used to establish a phylogenetic tree. ALDH proteins from the same families were clustered together, like the phylogeny generated with ALDH members from the twelve different organisms (Fig. 5A). Moreover, the *SbALDH* gene’s exon–intron structure was investigated to learn more about their potential structural evolution. Our findings revealed that genes in the same family usually had identical exon–intron structures, but nearly all families had several variations, with one or two exons being gained or lost in specific members in each case. Each of the *ALDH* genes in subfamilies of *SbALDH* -*2B*, *2C*, *3E*, *3H*, *10A*, and *18B* has an almost equal number of exons as well as introns. The number of exons ranges from 5 to 20, with the most exons in *SbALDH18B1* [20] and the fewest exons in *SbALDH2C2b*, *SbALDH2C2c*, and *SbALDH2C3* (5 each). In contrast, the number of introns varies from 4 to 19, where SbALDH18B1 has the highest number of 19 introns while *SbALDH2C2c* and *SbALDH2C3* have only four introns.

Allocation of the ALDH conserved domain (PF00171) was analyzed using Pfam for each SbALDH protein (Fig. 5B). Each putative SbALDH protein contains the conserved ALDH domain, while members of the same family share several unique structural similarities. Each SbALDH protein from family- 3, 10, and 18 has almost the same protein length as well as domain size. Apart from this, members of the ALDH family- 2, 3, 5, 6, 7, 10, 11, 12, and 22 comprised both the catalytic glutamic acid (PS00687) and cysteine (PS00070) active sites. On the contrary, proteins from the ALDH family 18 contained only cysteine active site but no glutamic acid active site. Besides, the conserved motifs of SbALDH proteins were analyzed using the MEME website to discover ten motifs (Table S2). These conserved motifs ranged in length between 21 to 41 amino acids. Interestingly, all these motifs were found to be highly conserved among the family-wise cluster of the phylogenetic tree (Fig. S4). All the identified SbALDH proteins comprised motif 1 and motif 4, while ALDH2 and ALDH5 members have motif 8.

### Expression profiling of *SbALDH* genes in various development stages and tissues

**Fig. 6:**
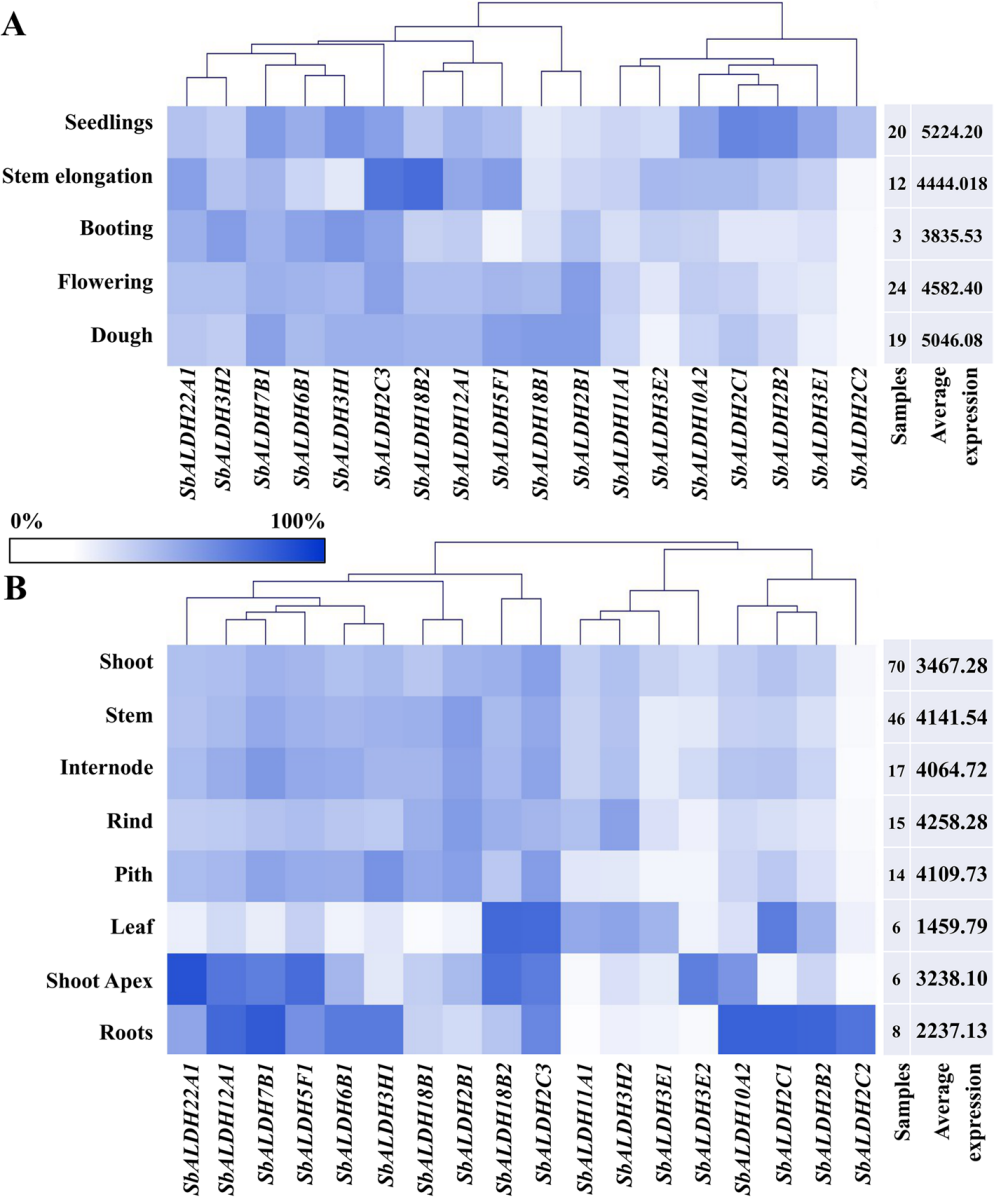
Expression analysis of *SbALDH* genes. The expression of *SbALDH* genes was analyzed at **A** different developmental stages, and **B** various anatomical tissues. The differential expression pattern of *SbALDH* genes is depicted by a heat map and a hierarchical cluster. The colour bar indicates the relative expression values, with white being the lowest level of expression and blue representing the highest level of expression. The description of samples and average expression is given on the right side of the heatmap. MeV 4.9 software (http://mev.tm4.org/) was used to create the heatmap using hierarchical clustering of Manhattan distance correlation criteria

Microarray expression data of *SbALDH* genes were retrieved from the Genevestigator to study their developmental and anatomical modulation in *S. bicolor*. However, no data could be found for the *SbALDH10A1* gene. The expression dataset for five developmental stages covering seedlings, stem elongation, booting, flowering, and dough that included a minimum of 3 samples for booting to a maximum of 24 samples for flowering (Fig. 6A). At each developmental stage, different *SbALDH* genes exhibited a differential expression pattern. Among all the analyzed genes, *SbALDH2C3* showed a high level of expression at all the developmental stages, while *SbALDH2C2* had the lowest level of expression (except in seedlings). Expression of *SbALDH* genes was moderately higher during the seedling and dough periods with an average expression value of 5224.20 and 5046.08, respectively, implying their involvement in the plant and grain maturation.

The expression of *SbALDH* genes was also investigated in various anatomical tissues including shoot, stem, internode, rind, pith, leaf, shoot apex, and roots (Fig. 6B). The number of transcripts with high levels of expression (> 60%) varied across tissues, with roots having the largest number of highly expressed *SbALDH* genes [8], subsequently rhizome with seven genes, and shoot apex with three genes. The *SbALDH* genes with the highest levels of expression in various tissues were *SbALDH22A1* (85%), *SbALDH7B1* (81%), *SbALDH2C1* (78%), *SbALDH10A2* (78%), *SbALDH2B2* (76%), and *SbALDH12A1* (74%). Remarkably, the highest average expression was found in the rind with a value of 4258.28, while the leaf had the lowest average expression value of 1459.79 (Fig. 6B).

### Expression analysis of *SbALDH* genes in response to abiotic stresses

**Fig. 7:**
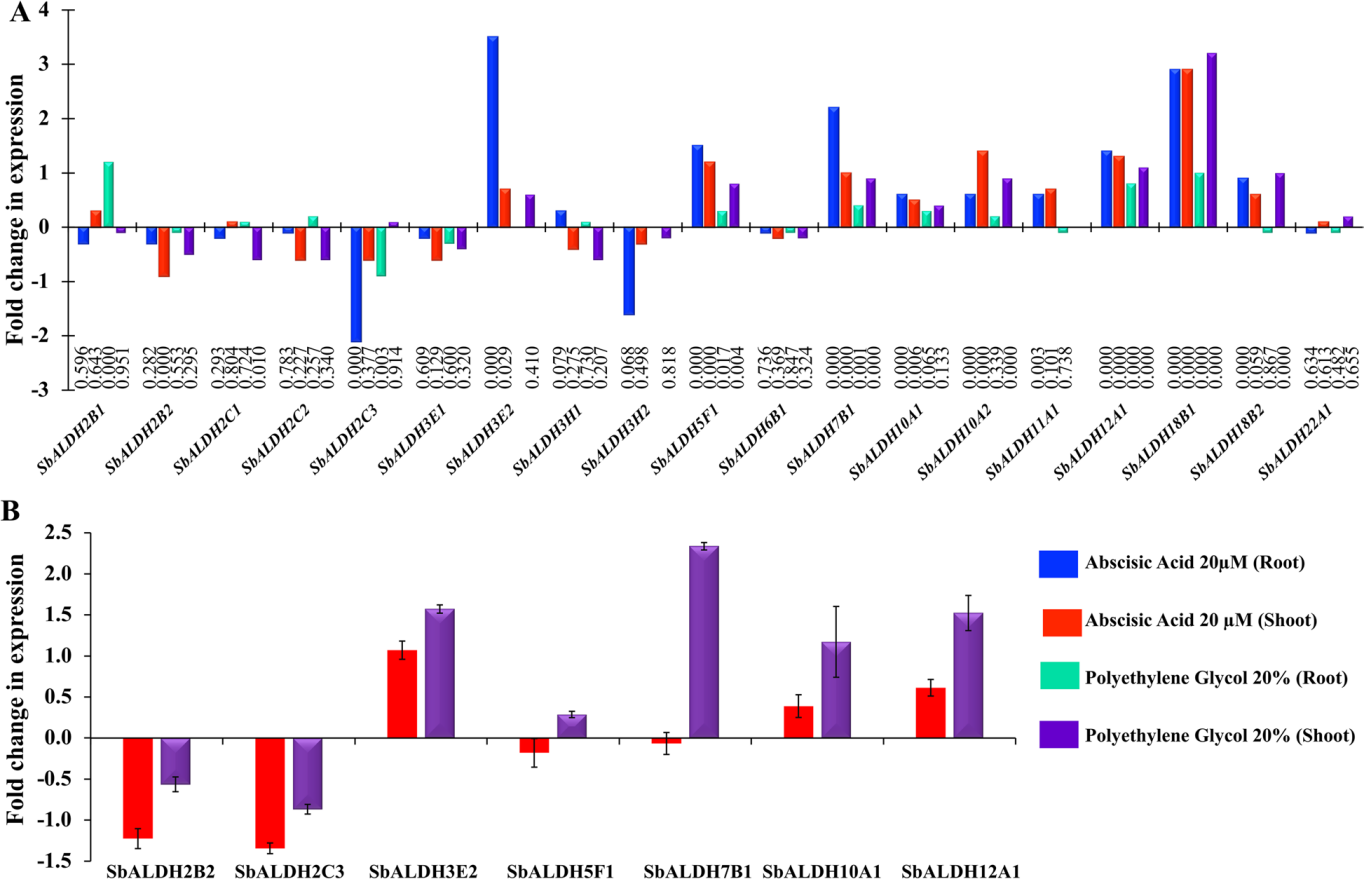
Stress mediated transcriptomic regulation of *SbALDH* genes. **A** Expression data for *SbALDH* genes were obtained from the Expression Atlas database. The coloured bars reflect the relative fold change in expression in response to abscisic acid (20 µM) and polyethylene glycol (20%). The statistical significance of the observed fold change for each gene is shown by the *p*-value at the bottom of the bar diagram. **B** The expression pattern of seven selected *SbALDH* genes was verified in a Bangladeshi variety in response to ABA and PEG using qRT-PCR. The average fold change in expression for each gene was calculated compared to their respective control and housekeeping gene values and presented in the bar diagram (*n* = 3)

To study the stress-mediated modulation of *SbALDH* genes, the curated perturbation and normalized expression data were obtained from the publicly accessible Expression Atlas database. Interestingly, *SbALDH5F1*, *SbALDH7B1*, *SbALDH10A1*, *SbALDH10A2*, *SbALDH12A*, and *SbALDH18B1* genes were found to be upregulated in response to 20 µM abscisic acid (ABA) and 20% polyethylene glycol (PEG) treatment in both root and shoot tissues (Fig. 7A). Among them, *SbALDH18B1* had shown the highest upregulation in both the mentioned stress conditions with a *p*-value of 0. Some of the genes were upregulated in a treatment-specific manner in both the tissues, viz. *SbALDH3E2*, *SbALDH11A1*, and *SbALDH18B2* were upregulated in both roots and shoots in response to ABA treatment only. Few genes showed tissue-specific expression. For example, *SbALDH2B1*, *SbALDH2C1*, and *SbALDH22A1* showed upregulation in shoots in response to ABA treatment, while downregulated in roots. Contrarily, *SbALDH3H1* had upregulation in roots but downregulated in shoots in response to ABA treatment (Fig. 7A). Similarly, responding to PEG treatment, *SbALDH2B1*, *SbALDH2C1*, *SbALDH2C2*, and *SbALDH3H1* genes were upregulated in roots while showing downregulation in shoots. On the other hand, *SbALDH18B2* and *SbALDH22A1* had upregulation in shoots but downregulation roots in response to PEG treatment. Few genes, such as *SbALDH2B2*, *SbALDH3E1*, and *SbALDH6B1* revealed complete downregulation in both the given treatments at both tissues.

### Verification of abiotic stress-responsiveness expression of a few selected *SbALDH* genes using qRT-PCR

The differential expression of seven selected *SbALDH* genes (*SbALDH*-2B2, 2C3, 3E2, 5F1, 7B1, 10A1, and 12A1) was verified in response to the same 20 µM abscisic acid (ABA) and 20% polyethylene glycol (PEG) treatment in one of the Bangladeshi varieties. Data analysis revealed that most of the analyzed *SbALDH* genes showed upregulation in response to both treatments except *SbALDH2B2* and *SbALDH2C3* (Fig. 7B). ABA and PEG induced down-regulation was found to be consistent for *SbALDH2B2* and *SbALDH2C3* in both RNA-seq and qRT-PCR analysis (Fig. 7). Transcripts of *SbALDH3E2* and *SbALDH12A1* showed a high level of upregulation in both conditions. This result confirmed the stress-specific transcript alteration of *SbALDH* members.

### Comparative analysis of the putative promoter regions of *SbALDH* genes

**Fig. 8:**
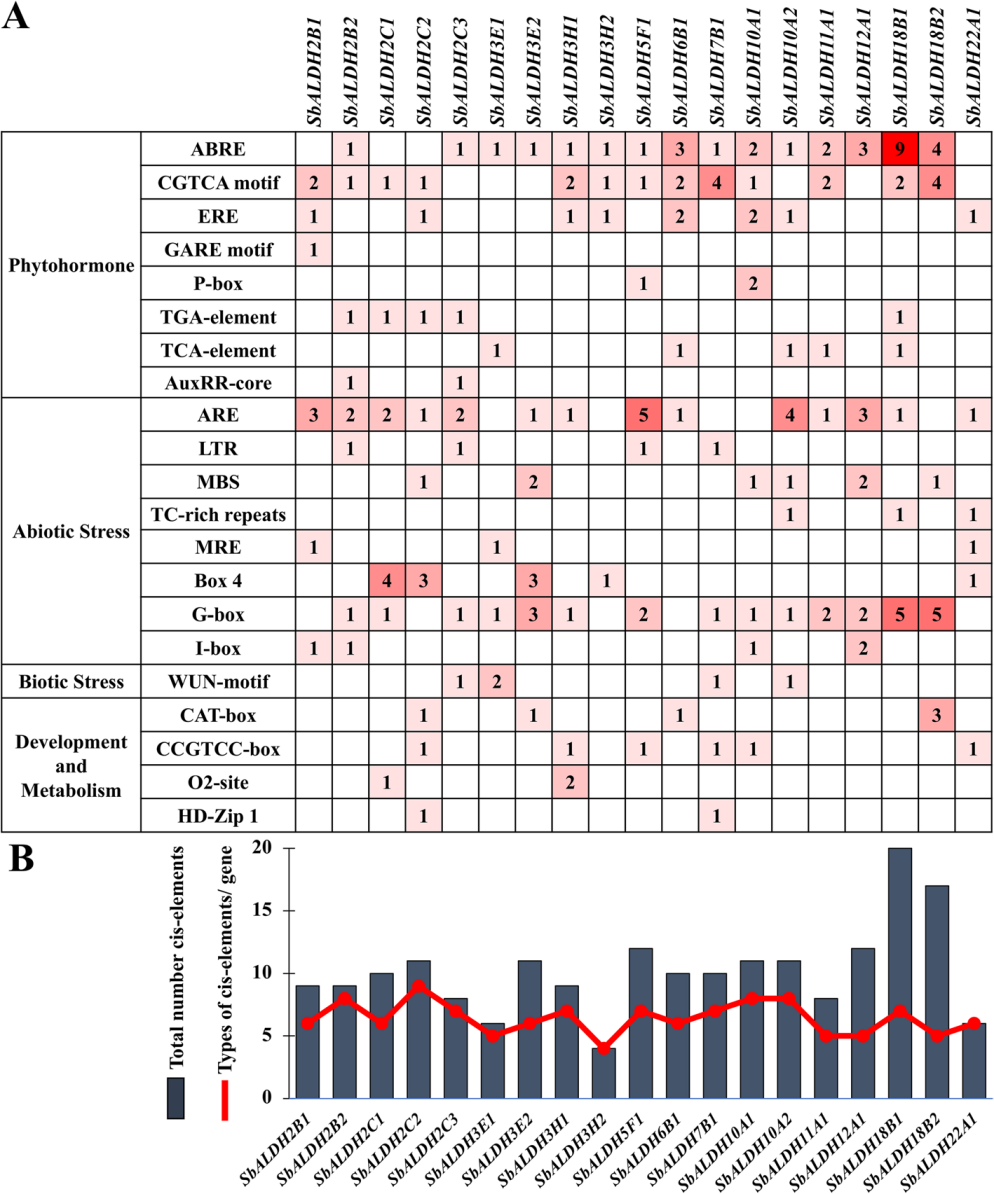
Analysis of cis-regulatory elements in the putative promoter region of *SbALDH* genes. **A** The number of each cis-regulatory element in the putative *SbALDH* promoter region. **B** Statistics on the overall number of *SbALDHs*, including the types of cis-elements per gene (red dot) and the total number of cis-elements in the *SbALDH* gene (charcoal grey box)

Cis-elements play a vital role in regulating molecular networks in a variety of biological activities as a core factor of transcriptional regulation [37]. The 1 kb upstream sequences from the translation start sites of *SbALDH* genes were submitted to PlantCARE to identify the cis-elements and for learning more about the possible regulatory mechanisms of *SbALDH* during abiotic stress responses. Therefore, eight phytohormone responsive cis-elements, eight abiotic responsive cis-elements, one biotic stress-responsive element, and four development and metabolism-related cis-elements were investigated in the putative promoter regions of *SbALDHs* (Fig. 8). The phytohormone responsive elements- ABRE, CGTCA motif, ERE, GARE, P-box, TGA-element, TCA-element, and AuxRR-core were identified in the promoter regions of 32, 24, 10, 1, 3, 5, 5, and 2 *SbALDH* genes, respectively (Fig. 8A) that indicates the abundant presence of ABRE motif in the promoter region of most of the *SbALDH* genes. Various abiotic and biotic stress-related cis-elements such as ARE, LTR, MBS, TC-rich element, MRE, Box 4, G-box, I-box, and WUN-motif were detected in the promoters of 28, 4, 8, 3, 3, 12, 27, 5, and 5 *SbALDH* genes, respectively. Besides, some of the development and metabolism-related elements viz. CAT-box (6), CCGTCC (6), O_2_-site (3), and HD-Zip 1 (2) were also found to be present in the putative promoter regions of *SbALDH* genes. As shown in Fig. 8B, *SbALDH18B1* comprised the highest number of cis-elements in its putative promoter region, while *SbALDH2C2* has the highest number of cis-element types. In contrast, the putative promoter sequence of *SbALDH3H2* had the lowest number and types of cis-acting elements (Fig. 8B).

### Structural modelling of SbALDH18B1 protein and its interaction with NADP.^+^ cofactor

**Fig. 9:**
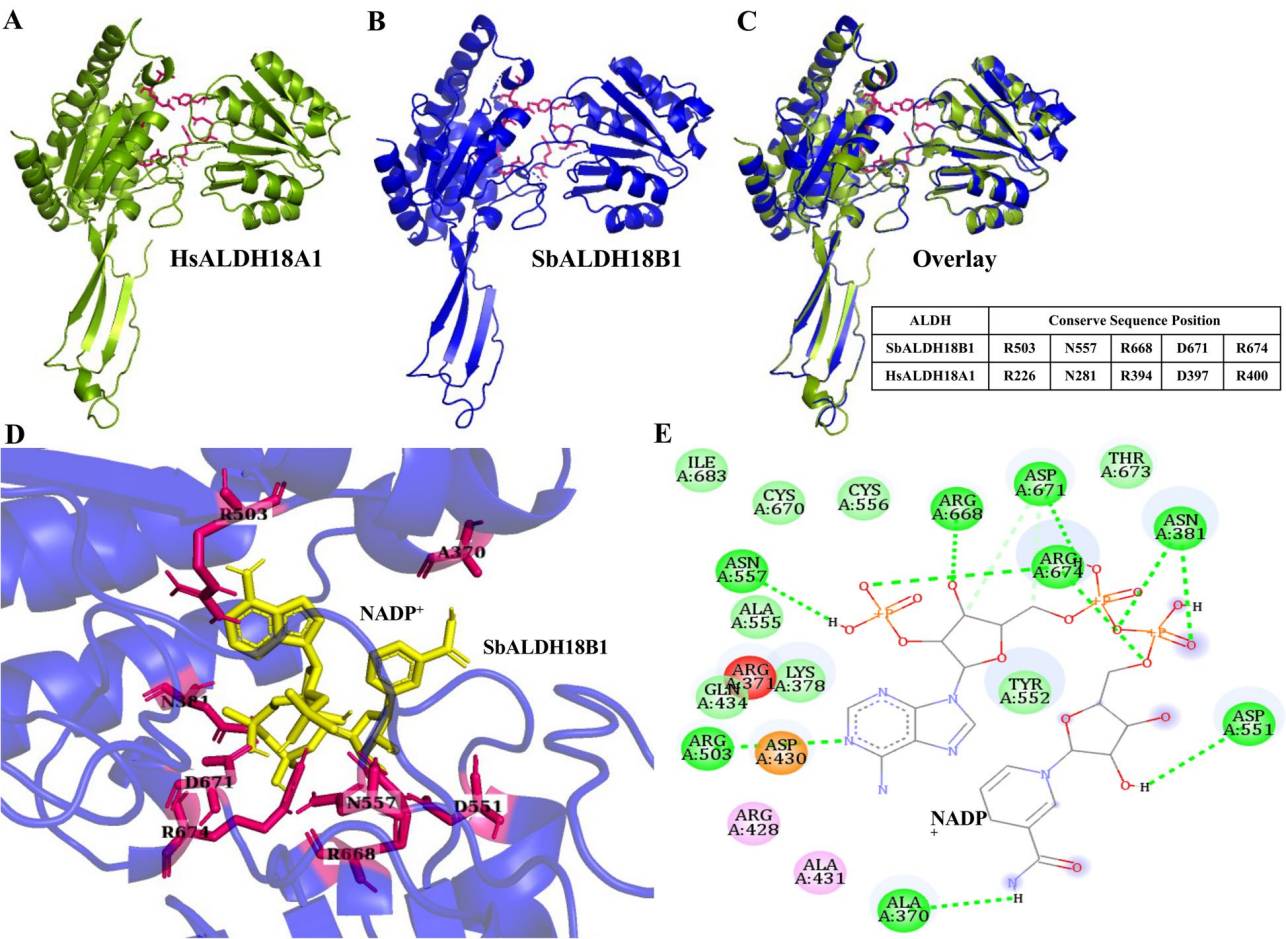
Homology modelling of the highly stress-responsive SbALDH18B1 protein. The Target-Template Alignment tool of the SWISS-MODEL server (https://swissmodel.expasy.org/) was used to create the SbALDH18B1 protein model by using the human ALDH18A1 protein structure as a templet. 3D structure of **A** HsALDH18A1 and **B** SbALDH18B1 illustrating conserved NADP^+^ binding site (shown as a pink stick). **C** Overlaying the structure of HsALDH18A1 with SbALDH18B1 to depict their structural similarity and conserved sequence position. **D** Interaction of SbALDH18B1 protein with NADP cofactor (shown as 3D). **E** Interaction of SbALDH18B1 protein with NADP^+^ cofactor (shown as 2D)

The structures and functional associated characteristics of ALDH proteins could be investigated to better understand the substrate specificity/range and enhancement of enzymatic activity. The homology model of a highly stress-responsive SbALDH18B1 protein was generated (Fig. 9B) using the closely related template structure of *H. sapiens* ALDH18A1 (PDB: 2H5G_A, Fig. 9A) to understand the overall 3D coordination and its interaction with NADP^+^ cofactor through 2D and 3D plot (Fig. 9, D and E). Moreover, the MolProbity Ramachandran analysis concluded that 96.2% (405/421) of modelled SbALDH18B1 residues were in favoured regions, while 99.3% (418/421) of residues were in the allowed regions (Fig. S5). The approximate QMEAN score for the predicted model was -1.57. Interestingly, some of the NADP^+^-binding domain residues in SbALDH18B1 viz. R503, N557, R668, D671, and R674 were recognized to be conserved after structural alignment and overlaying on the HsALDH18A1 protein (Fig. 9C). Moreover, the interaction of the SbALDH18B1 protein with the NADP^+^ cofactor revealed that NADP^+^ formed a conventional hydrogen bond with A370, N381, R503, D551, N557, R668, D671, and R674 residues of the protein. Additionally, the SbALDH18B1 and its substrate (NADP^+^) had binding energy of -8.7 kcal/mol.

## Discussion

Active aldehyde dehydrogenases are key to the detoxification mechanism for reactive aldehydes originating during developmental stages and in response to environmental stresses [20]. ALDHs are found in both prokaryotic and eukaryotic organisms and are well-represented in all plant species studied to date [33]. Many plant species have undergone comprehensive research and expression analysis for the ALDH superfamily, but it has not been performed yet in sorghum. *Sorghum bicolor* is regarded as a high-energy, drought persistent plant because of its high efficiency in terms of solar energy conversion and water usage [38]. The completion of sorghum genome sequencing [30] offered great scope for conducting a genome-wide characterization and expression profiling of *SbALDH* genes. The current study represents the identification, nomenclature, characterization, family expansion, evolution, and transcript abundance of *SbALDH* genes.

A total of 19 *ALDH* genes were found in the genome of *Sorghum bicolor* which is comparable with the previously reported number of 16 *ALDH* genes in *Arabidopsis thaliana* [11], 20 in *Oryza Sativa* [17], 39 in *Malus domestica* [39], 22 in *Zea mays* [40], 53 in *Glycine max* [18], 23 in *Vitis vinifera* [8], 23 in *Brassica rapa* [41], 29 *Solanum lycopersicum* [42], 30 in *Gossypium Raimondi* [43], 26 in *Populus trichocarpa* [44] and 22 in *Solanum tuberosum* [34]. Each of the *SbALDH* members comprises a conserved ALDH domain. A total of 24 ALDH family has been observed across all organisms, where 14 families are specific for plants. The number of *ALDH* genes has risen in the higher plants due to several rounds of genome duplication and expansion during evolution [45]. The lengths of the Sorghum ALDH proteins ranged from 391 to 729 amino acids, while the lengths of the ALDH proteins in Arabidopsis and rice ranged from 484 to 726 aa [11] and 423 to 735 aa [17], respectively. The gene structure of these *SbALDH* genes showed a lot of variances, suggesting a huge complexity among the SbALDH family. Exon–intron increases and declines are caused by the fusion and realignment of the gene fragments [46]. As a result, changes in gene structure play a significant role in gene family evolution [46]. The number of exons in the SbALDH genes was found to differ among different families but almost identical among the same family members. Furthermore, members of the same family had identical motif arrangements across the SbALDH proteins. This indicates the structural and functional variation among different SbALDH proteins.

Unlike plant ALDH proteins, which were classified into 14 families, SbALDH proteins were classified into ten major families (ALDH- 2, 3, 5, 6, 7, 10, 11, 12, 18, and 22) in the tree, which is consistent with the previous results in other higher plant species, viz. Arabidopsis [11], rice [47], apple [39], grape [8], soybean [18], mustard [41], and potato [34], except tomato which had 11 ALDH families [15, 42]. In contrast, ALDH family- 19, 21, 23, and 24 were not found in higher plants because only genes from primitive terrestrial plants were discovered with ALDH21 and ALDH23 families [48], only *Chlamydomonas reinhardtii* had ALDH 24 family members [16], and ALDH19 has been only reported in tomato till date [15, 42]. It is possible that ALDH-21, 23, and 24 families played significant roles in the evolution of lower plants before extinct in higher plants. There are also other ALDH families in the phylogenetic tree, like the ALDH family- 1, 4, 8, 9, and 16 which have not yet been found in any plant species, but these families can be found in mammalian species (such as human and mouse). Apart from these findings, ALDH family- 2, 5, and 10 tended to cluster together in the phylogenetic tree, while a node with a high bootstrap value linked families- 22 and 3 (closely related), which is resembling the previous studies in Arabidopsis [13], rice [17], and soybean [18]. Interestingly, each of the *SbALDH* genes was discovered to be more closely related to rice and maize *ALDH* genes than other higher plants (Fig. 3), which is coherent with the fact that sorghum, maize, and rice are all monocots that diverged more anciently than the eudicot lineage.

The functions of *ALDH* genes had been thoroughly investigated in many plants. Expansion of ALDH isoforms in the higher plants might provide higher plasticity and neofunctionalization in their actions to achieve diversified roles. Members of the ALDH2 family metabolize acetaldehyde, while ALDH6 family members, function as methyl malonyl semialdehyde dehydrogenases, promote reactions related to valine and pyrimidine catabolism [33]. Members of the ALDH5 are involved in the GABA ‘shunt’ pathway, which helps species to avoid the tricarboxylic acid pathway in the metabolic process [33]. Besides their important roles in different metabolic processes, several plant *ALDH* genes have been documented to act on a variety of abiotic stresses, including drought, salinity, cold, heat, and in the treatment of ABA and PEG [21, 23, 49]. Overexpression of *ALDH3I1* in transgenic plants had shown resistance to a variety of stresses [19]. The expression of *OsALDH3-*4 and *GmALDH7B1* was found to be upregulated in response to ABA in young rice leaves [17], and PEG treatment in soybean [18], respectively. *ALDH* genes from different plant or crop species showed a similar pattern of differential expression under various abiotic stress conditions. Transcript upregulation of *StALDH12A1*, *StALDH7A1*, and *StALDH2B6* was observed in one of the Bangladeshi potato varieties (BARI Alu-7) in response to salinity, drought, and heat [34]. Similarly, transcript enhancement for most of the *AtALDH* and *OsALDH* genes was observed in response to salinity, drought, osmotic, and cold stresses [34]. In the present study, *SbALDH3E2, SbALDH7B1* and *SbALDH18B1* were found to be highly upregulated in response to ABA and PEG treatments (Fig. 7). Thus, the abiotic stress-specific transcript alteration of *ALDH* was found to be evolutionarily conserved in both monocot and dicotyledons plant species.

The cis-acting regulatory elements in the putative promoter region of the plant have a prominent role in different stress responses [50]. At least one cis-regulatory element was found in the promoter region of each *SbALDH* gene that was linked to phytohormones or abiotic/biotic stress. The putative promoter of *SbALDH18B1* has the highest amount of ABRE cis-elements (involve in ABA responsiveness) which is consistent with the fact that this gene had shown the highest upregulation in response to ABA treatment (Figs. 7 and 8). Other genes including *SbALDH-3E2, 5F1, 7B1, 10A1, 10A2, 11A1, 12A1, 18B1,* and *18B2* which contained the ABRE element in their putative promoter region, were also found to be responsive in ABA treatment. Similarly, promoters of genes such as *SbALDH3E2*, *SbALDH10A1, SbALDH10A2, SbALDH12A1,* and *SbALDH18B2* with MBS (MYB binding site involved in drought inducibility) element, showed upregulation in PEG induced drought stress. Overall, our findings indicate that the abundance of cis-elements essential regulator of *SbALDH* gene expression in response to ABA and PEG treatments.

## Conclusion

In brief, a systematic genome-wide analysis was conducted and hypothesized extensive knowledge of the *ALDH* gene family from *Sorghum bicolor*. The extension of the *ALDH* gene family in sorghum has been aided by segmental and tandem gene duplication. Additionally, several *ALDH* genes from sorghum and maize were found in duplication blocks, indicating that they are possibly orthologues. The identified SbALDH members can be divided into ten phylogenetically conserved families as analogous to other plant species. Expression profile analysis gave insight into the potential functional differences between *SbALDH* members. Although the exact functions of multiple SbALDH members are uncertain, the phylogenetic, structural, and expression analyses may aid in the selection of suitable genes for further functional characterization and making stress-resistant crops.

## Methods

### Database search, gene annotation, and characterization of ALDH superfamily in *S. bicolor*

Hidden Markov Model (HMM) profile of the ALDH domain PF00171 was searched in the PhytoMine tool of the Phytozome v.12 databases (https://phytozome.jgi.doe.gov/phytomine/template.do?name=PFAM_Proteins&scope=all) against the annotated proteins of sorghum to find out the ALDH protein superfamily in *S. bicolor*. Following that, blastP searches (with an E-value < 1e-3) were also conducted using all Arabidopsis, rice, tomato, maize, *Selaginella moellendorffii*, moss, and algae ALDHs sequences as queries. All the identified protein sequences were checked to verify the presence of the conserved ALDH domain (PF00171) using Pfam (http://pfam.xfam.org/) and NCBI Conserved Domain Database (https://www.ncbi.nlm.nih.gov/Structure/cdd/wrpsb.cgi). The presence of the ALDH cysteine active site (PS00070) and glutamic active site (PS00687) was confirmed using the ScanProsite tool (https://prosite.expasy.org/scanprosite/) as well as using multiple sequence alignment. Putative sorghum ALDHs were annotated based on the nomenclature criteria of the ALDH Gene Nomenclature Committee (AGNC) [51]. According to this criteria, protein sequences, more than 40% identical to the previously identified ALDH sequences comprise a family and protein sequences having a similarity of more than 60% comprise a subfamily. Protein sequences having less than 40% identity with previously identified ALDH sequences represent a novel ALDH family. For nomenclature, the prefix “Sb” for *Sorghum bicolor* was added to the gene root symbol “ALDH” followed by a family specifier (2, 3, 5, etc.), a subfamily indicator (B, C, E, etc.), a number as per the chromosomal position of the gene with each subfamily, and a low case letter (a, b, c, etc.) for labelling the variants. Precise information about the locus ID, transcript ID, coordinate (5’ to 3’), length of the transcript, CDS, and protein were collected from the PhytoMine tool of the Phytozome v.12. Physiochemical parameters of the identified proteins such as molecular weight and theoretical isoelectric point were collected from the ProtParam tool (https://web.expasy.org/protparam/). Subcellular localization of each protein was predicted using the Plant-mPLoc server (http://www.csbio.sjtu.edu.cn/bioinf/plant-multi/) [52].

### Chromosomal localization, and duplication analysis

All the *SbALDH* genes were mapped to sorghum chromosomes based on the chromosomal location information available at the PhytoMine tool of the Phytozome v.12 databases. For synteny analysis, syntenic blocks within the *Sorghum bicolor* genome and between *Sorghum bicolor* and *Zea mays* genomes were extracted from the Plant Genome Duplication Database (PGDD) (http://chibba.agtec.uga.edu/duplication/index/downloads) [53]. The syntenic relationship and chromosomal distribution of *ALDH* genes were visualized using the Circos software [54]. Tandem duplication was identified with a criterion that two or more homologous genes on the same chromosome within a 100 kb region [55], while more than 90% of sequence identities within genes were regarded as segmental duplication [56]. Synonymous (Ks) and nonsynonymous (Ka) substitution rates were also collected from the Plant Genome Duplication Database. The Ka/Ks ratio was used to measure the selective pressure of the duplicated genes, with Ka/Ks ratios of > 1, < 1, and = 1 indicating positive, negative, and neutral selection, respectively [57]. The duplication time (T) of each *SbALDH* duplicated gene pair was estimated by using the formula: T = Ks/(2 × 6.1 × 10^−9^) × 10^−6^ Mya [58].

### Analysis of exon–intron organization, protein domain architecture and motif

Genomic and CDS sequences of *SbALDH* genes were used in the Gene Structure Display Server 2.0 (http://gsds.gao-lab.org/) to analyze the exon–intron organization. The position of the conserved ALDH domain in the SbALDH proteins was detected from the Pfam (http://pfam.xfam.org/) database. SMART (https://prosite.expasy.org/scanprosite/) was used to identify the presence and position of the conserved cysteine and glutamic acid residues. Domain architecture of the proteins along with the active sites was illustrated using the IBS 1.0 (Illustrator of Biological Sequences) software package [59]. The MEME software was used to find the conserved motifs in the SbALDH sequences, with the following criteria: zero or one occurrence per sequence (zoops) site distribution, a limit of 10 motif findings, and a motif width of 6—50 amino acid residues.

### Phylogenetic analysis of SbALDH proteins

The evolutionary relationships among ALDH proteins from sorghum, rice, Arabidopsis, apple, maize, soybean, grapevine, field mustard, potato, tomato, black cottonwood, human, mouse, moss, gemmiferous spike moss, unicellular green algae, and marine green algae were analyzed using the MEGA-X software [60] after protein sequences were aligned using the ClustalW program [61]. Evolutionary analysis was performed with the Maximum-likelihood algorithm [62] and the criteria were set as follows: Jones-Taylor-Thornton (JTT) model, partial deletion with 95% site coverage cutoff, and bootstraps test with 1000 replicates.

### Lineage-specific expansion of SbALDH in comparison with maize and rice

To investigate the lineage-specific expansion of SbALDH members in comparison with maize and rice, ALDH families (ALDH family- 2, 3, 5, 10, and 18) with multiple members were considered. Family-specific phylogenetic trees were constructed among sorghum and maize; and sorghum and rice members using MEGA-X software with the above-mentioned criteria (Islam et al. 2019). Evolutionary analysis was conducted by identifying the most recent common ancestor (MRCA) based on the node branches of the tree.

### Expression profiling of *SbALDH* genes

Microarray expression data of *S. bicolor ALDH* genes at various anatomical parts (shoot, stem, internode, rind, pith, leaf, shoot apex, rhizome, and root) and developmental stages (seedlings, stem elongation, booting, flowering, and dough) were obtained from the publicly available Genevestigator database [63]. Generation of the heatmap for the anatomical and developmental expression data was executed using MeV 4.9 software package [64]. The mRNA level of nine days old *Sorghum bicolor* (BTx623) was analyzed in two tissue types (roots and shoots) in response to two treatments (20 uM Abscisic Acid, ABA and 20% Polyethene Glycol, PEG) with the corresponding control of 0.2 M NaOH and H_2_O, respectively for 27 h (E-GEOD-30249). The normalized and curated RNA-seq expression data of *SbALDH* genes in response to 20 µM ABA and 20% PEG were retrieved from the Expression Atlas database (https://www.ebi.ac.uk/gxa/experiments/E-GEOD-30249/Results) experiment no E-GEOD-30249 [65]. Expression patterns in response to ABA and PEG were illustrated using the histogram.

### Plant materials and stress treatments

Expression profiles of selected *SbALDH* genes were evaluated in one of the Bangladeshi sorghum varieties (BARI sorghum 1). Seeds were collected from Bangladesh Agricultural Research Institute (BARI), Bangladesh. Seedlings were grown in a greenhouse at 28 ± 2 °C for nine days according to Bhowal et al. [66]. The seedlings were sprayed with 20 µM ABA, irrigated with 20% PEG, or irrigated with normal water as control. Shoot tissues were harvested after 24 h of treatment from the control and both treated seedlings, and directly immersed in liquid N_2_ followed by − 80 °C preservation. All assessments were performed with three biological replicates.

### RNA Isolation, cDNA synthesis and qRT-PCR

Total plant RNA was isolated from all the harvested samples using TRIzol reagent (Invitrogen, USA) according to the manufacturer’s instructions. ProtoScript® II First Strand cDNA Synthesis Kit (NEB, UK) was used for the synthesis of first-strand cDNA using RNase-free DNaseI treated total RNA. Gene-specific primers were designed using the Primer-BLAST program (http://www.ncbi.nlm.nih.gov/tools/primer-blast/), and previously reported *SbEIF*-1α [66] was used as a reference gene to normalize the data (Table S3). GoTaq® qPCR Master Mix (Promega, USA) was used according to the manufacturer’s instructions to perform the quantitative real-time PCR assay via Bio-Rad CFX96 Real-Time PCR Detection System (Bio-Rad, USA). The specificity of the amplicon was confirmed by melt curve analysis. The cycling program included an initial denaturation at 94 °C for 5 min, followed by 40 cycles of 94 °C denaturation for 10 s and 60 °C for 30 s. Three technical replicates were analysed for each sample and the data was represented as the mean values ± SE. The relative expression in fold change for each candidate gene was calculated using the 2^−∆∆CT^ method [67].

### Inquisition of putative cis-regulatory elements and their enrichment

For analyzing the cis-acting elements in the promoter region of *SbALDH* genes*,* 1 kb 5’ upstream sequences from each of the *SbALDH* genes were obtained from the Phytozome v.12 databases (https://phytozome.jgi.doe.gov/pz/portal.html). Hereafter, retrieved sequences were submitted to the PlantCARE database (http://bioinformatics.psb.ugent.be/webtools/plantcare/html/) [68] to identify the presence of cis-acting regulatory elements. The cis-regulatory elements that participated in response to various abiotic and biotic stresses, as well as hormonal responses and during the development of plants, are illustrated.

### Protein modelling and structural features analysis

For homology-based modelling, the amino acid sequence of the highest stress-responsive member SbALDH18B1 was searched against the protein data bank in the NCBI BLASTp suite. The best homologous structure, 2H5G_A corresponding to human ALDH18A1 (identity 49%) was used as a temple structure. SbALDH18B1 protein model was generated using the Target-Template Alignment tool of the SWISS-MODEL server (https://swissmodel.expasy.org/) [69]. The model structure was validated and the number of protein residues in the favoured and allowed regions was also computed using the Ramachandran plot statistics (https://montelionelab.chem.rpi.edu/PSVS/). The built protein model was illustrated using PyMOL v2.4 software and compared with the human ALDH18A1 model by overlaying it.

For protein cofactor binding analysis, docking of SbALDH18B1 (substrate) with NADP^+^ cofactor (ligand) (PubChem CID: 5893) was carried out using the AutoDock Vina v1.1.2 [70], and the PDBQT file was created by using the MGL tools [57]. 2D diagram of the protein and cofactor interaction was illustrated using BIOVIA Discovery Studio Visualizer v.4.5.

## Supplementary Information

**Additional file 1:** **Table S1.** Duplication analysis of ALDH genes between *S. bicolor* and *Z. mays*. **Table S2.** Detailed information of conserved motifs in the SbALDH proteins. **Table S3.** List primers used for the qRT-PCR analysis. **Fig. S1.** Distribution and duplication of ALDH genes on sorghum chromosomes. **Fig. S2.** The evolutionary links of sorghum and maize ALDH. **Fig. S3.** The evolutionary links of sorghum and rice ALDH. **Fig. S4.** The amino acid motifs of the SbALDH protein are depicted in a schematic diagram. **Fig. S5.** MolProbity Ramachandran plot for validating the 3d model of SbALDH18B1 protein.

**Additional file 2: Appendix 1.** All the sequences are used for the construction of phylogenetic tree.

**Additional file 3.** 

## Data Availability

The authors declare that all the data and plant materials will be available without restrictions. The datasets used in this study are included in the article and its supplementary files. The sequence data obtained from PhytoMine tool of the Phytozome v.12 databases (https://phytozome.jgi.doe.gov/phytomine/template.do?name=PFAM_Proteins&scope=all) for Sorghum bicolour. Other sequences used in the study have been provided as Additional file 2. The sequencing reads data of sorghum bicolor in response to osmotic stress and abscisic acid are available in the Expression Atlas database repository (https://www.ebi.ac.uk/gxa/experiments/E-GEOD-30249/Results).

## Abbreviations

ALDH: Aldehyde dehydrogenase
PGDD: Plant Genome Duplication Database
Ka/ Ks: Ratio of nonsynonymous substitutions (Ka) to synonymous substitutions (Ks)
MW: Molecular weight
MRCA: Most Recent Common Ancestor
ABA: Abscisic Acid
PEG: Polyethylene Glycol
