# Supplementary material for: Aldehyde dehydrogenase superfamily in sorghum: genome-wide identification, evolution, and transcript profiling during development stages and stress conditions
Source: BMC Plant Biol. 2022 Jul 4;22:316. doi: 10.1186/s12870-022-03708-4 (PMC9252066; doi:10.1186/s12870-022-03708-4)
Supplement: Supplementary file 1 — Additional file 1: Table S1. Duplication analysis of ALDH genes between S. bicolor and Z. mays. Table S2. Detailed information of conserved motifs in the SbALDH proteins. Table S3. List primers used for the qRT-PCR analysis. Fig. S1. Distribution and duplication of ALDH genes on sorghum chromosomes. Fig. S2. The evolutionary links of sorghum and maize ALDH. Fig. S3. The evolutionary links of sorghum and rice ALDH. Fig. S4. The amino acid motifs of the SbALDH protein are depicted in a schematic diagram. Fig. S5. MolProbity Ramachandran plot for validating the 3d model of SbALDH18B1 protein. [file 12870_2022_3708_MOESM1_ESM.pdf]

Aldehyde dehydrogenase superfamily in sorghum: Genome-wide identification, evolution, and transcript profiling during development stages and stress conditions

Short title: Aldehyde dehydrogenase superfamily in sorghum

Md. Sifatul Islam[1], Munira Mohtasim[2], Tahmina Islam[2] and Ajit Ghosh[1*]

[1]Department of Biochemistry and Molecular Biology, Shahjalal University of Science and Technology, Sylhet-3114, Bangladesh.
[2]Plant Breeding and Biotechnology Laboratory, Department of Botany, University of Dhaka, Dhaka 1000, Bangladesh.

*To whom correspondence should be addressed:
Ajit Ghosh, PhD,
e-mail: aghosh-bmb@sust.edu

Table S1. Duplication analysis of ALDH genes between *S. bicolor* and *Z. mays*.

Sl no	Sorghum ALDH genes	Maize ALDH genes
1	SbALDH3E1	ZmALDH3E1
		ZmALDH3E2
2	SbALDH3E2	ZmALDH3E1
		ZmALDH3E2
3	SbALDH3H1	ZmALDH3H2
		ZmALDH3H3
		ZmALDH3H1
4	SbALDH3H2	ZmALDH3H2
		ZmALDH3H1
5	SbALDH6B1	ZmALDH6B1
6	SbALDH7B1	ZmALDH7B6
7	SbALDH10A2	ZmALDH10A8
8	SbALDH11A1	ZmALDH11A3
9	SbALDH12A1	ZmALDH12A1
10	SbALDH18B1	ZmALDH18B2
		ZmALDH18B1
11	SbALDH18B2	ZmALDH18B1
		ZmALDH18B2
12	SbALDH22A1	ZmALDH22A1

Table S2: Detailed information of conserved motifs in the SbALDH proteins.

No.	Motif	Width	Site	E-value
1	KEPVGVVGLITPWNFPLLMFT	21	34	7.5e-314
2	DMKIAREEIFGPVLPJIKFKTEEEAIERANDTPYGLA	37	27	1.5e-446
3	FDDADIDMAVELSLFAKFFNKGQTCVAGSRILVQEGIYDEF	41	21	2.2e-435
4	WKVGPALAAGNTVVLKPSEQTPLSALYLA	29	34	4.5e-369
5	LAKEAGLPDGVLNVVPGFGPTAGAALASHMDVDKVAFTGST	41	20	5.9e-422
6	PFGGVKMSGFGREKGKYGLDNYLQVKAV	28	23	2.3e-295
7	AAASNLKPVSLELGGKSPCIV	21	32	1.1e-266
8	VEVLFTKLLINGKFVDAASGKTFETRDPRTGEVJANVAEGD	41	12	1.6e-232
9	LTAYERSKILLKFADLIEEHKEEJAALETJDVG	33	22	2.8e-206
10	TNQGPQIDEDQFEKVLKYINDGKSEGATJ	29	21	3.4e-244

Table S3: List primers used for the qRT-PCR analysis

Sl	Primer Name	Forward Primer (5′→3′)	Reverse Primer (5′→3′)
1	**SbALDH2B2**	GCAGCAGTCACCCAATTGCTC	TACCACCCTATCCCACGTGTC
2	**SbALDH2C3**	TTGAATCGCTTCGAGGCCCA	CGCTCGCTGTCAATCTGTGC
3	**SbALDH3E2**	AGCTGGAAATCGCGAGACGA	CTCGCTAGCCCGCTCTTGTA
4	**SbALDH5F1**	CGTTACAGGCTGTCGGTGGT	GCCTGGTTGAAAGATCGGCG
5	**SbALDH7B1**	GTCTCGTCCCGTGAACAAACA	GGCGTGGTGAAGTAGTGAGGT
6	**SbALDH10A1**	TTTGCACAGTTTGCACGGGA	GTGCCCTCCGGTGACTGTTT
7	**SbALDH12A1**	TCACAGCTGCCGTGGAAAGT	TTGGGGAGAGTGGGCTGTTG
8	**SbEIF4α**	CAACTTTGTCACCCGCGATGA	TCCAGAAACCTTAGCAGCCCA

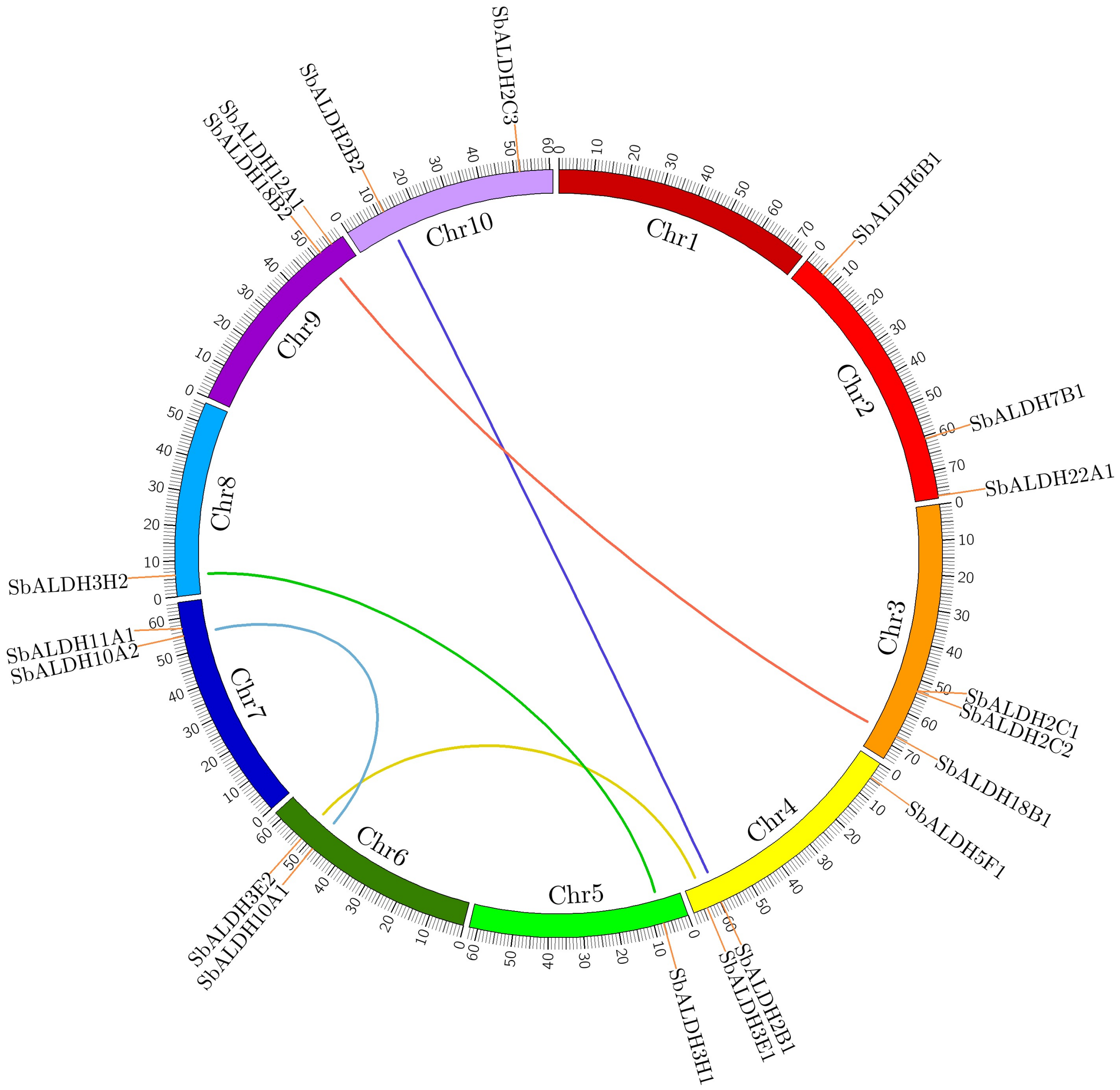

Fig. S1. Distribution and duplication of ALDH genes on different chromosomes of sorghum.

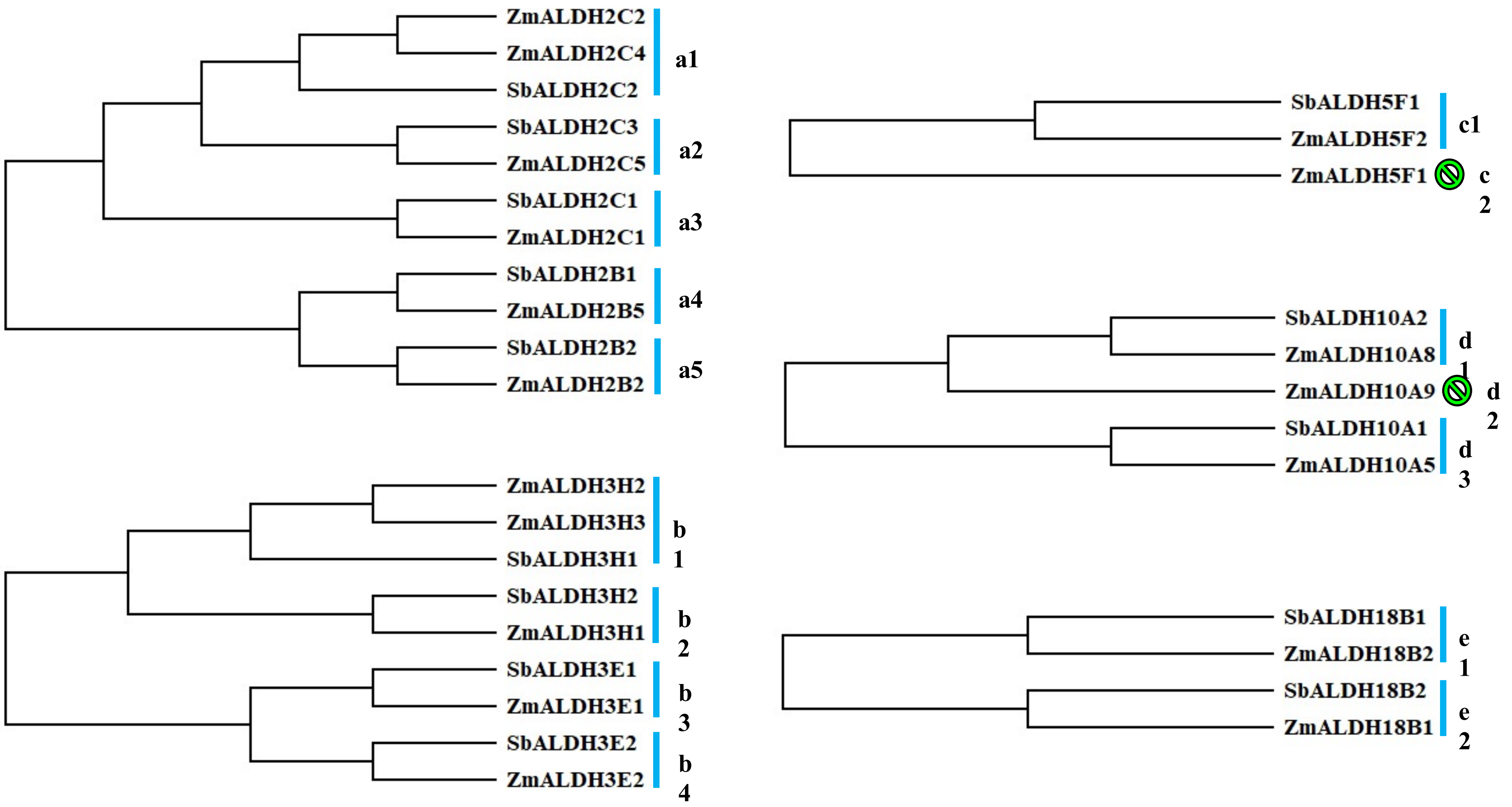

Fig. S2. The evolutionary links of sorghum and maize ALDH

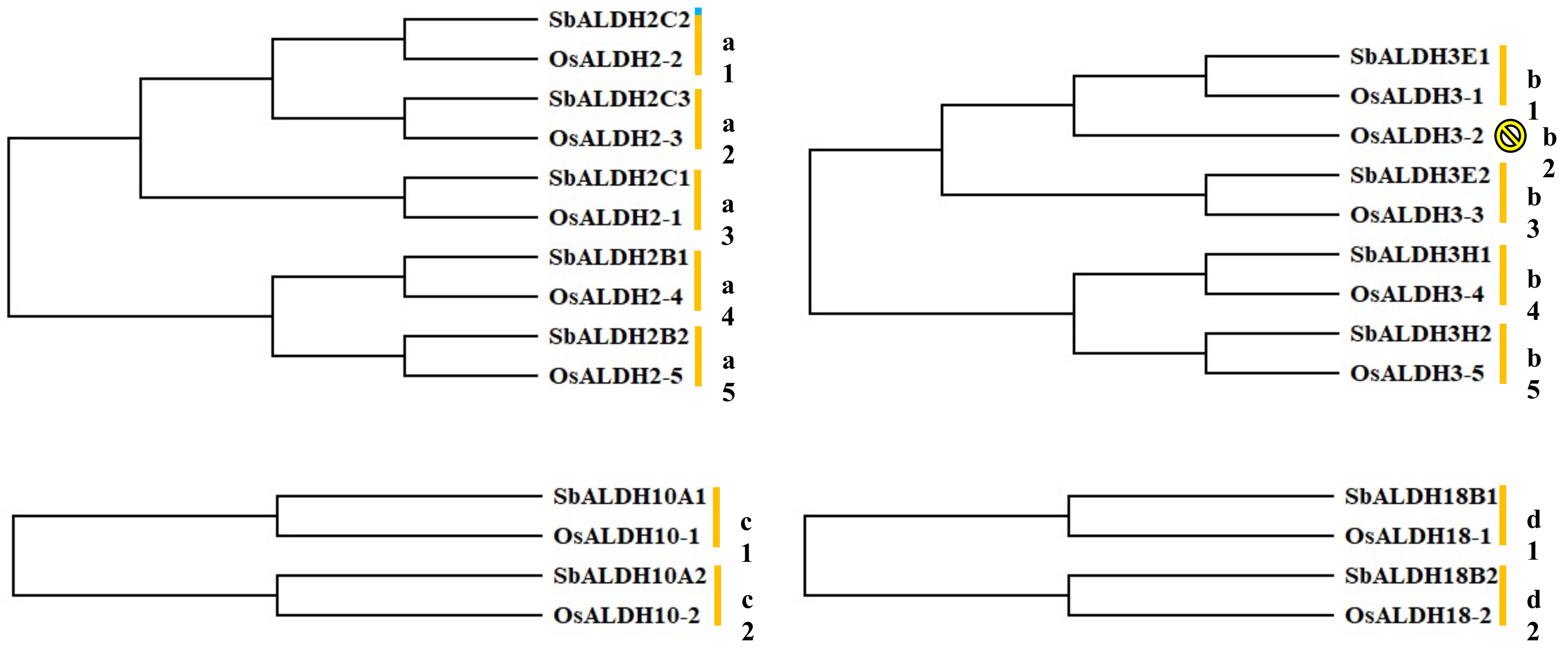

Fig. S3. The evolutionary links of sorghum and rice ALDH

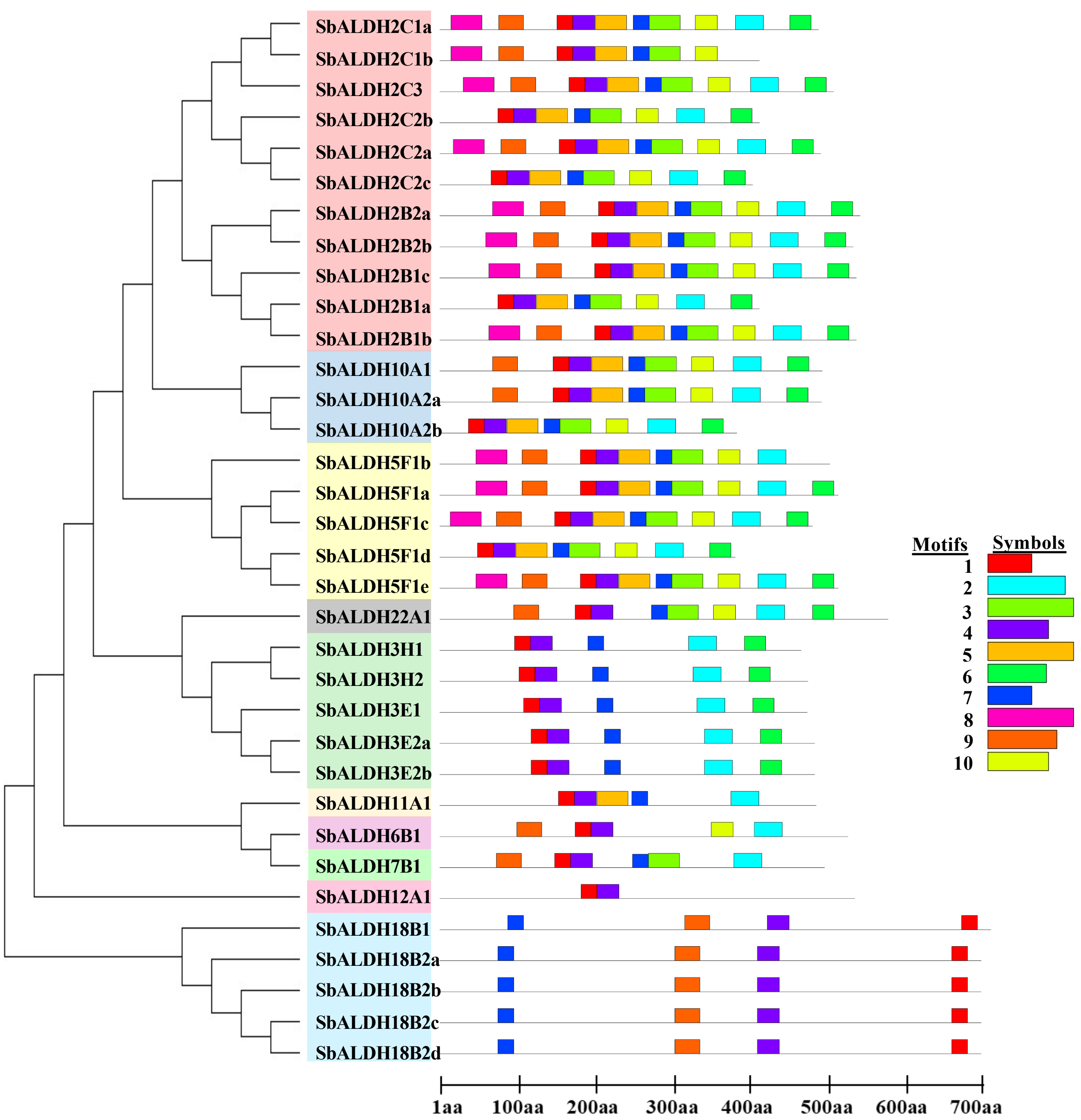

Fig. S4. The amino acid motifs of the SbALDH protein are depicted in a schematic diagram. The various coloured boxes represent different motifs and their positions in each ALDH sequence.

MolProbity Ramachandran analysis

SbALDH18B1.pdb, model 1

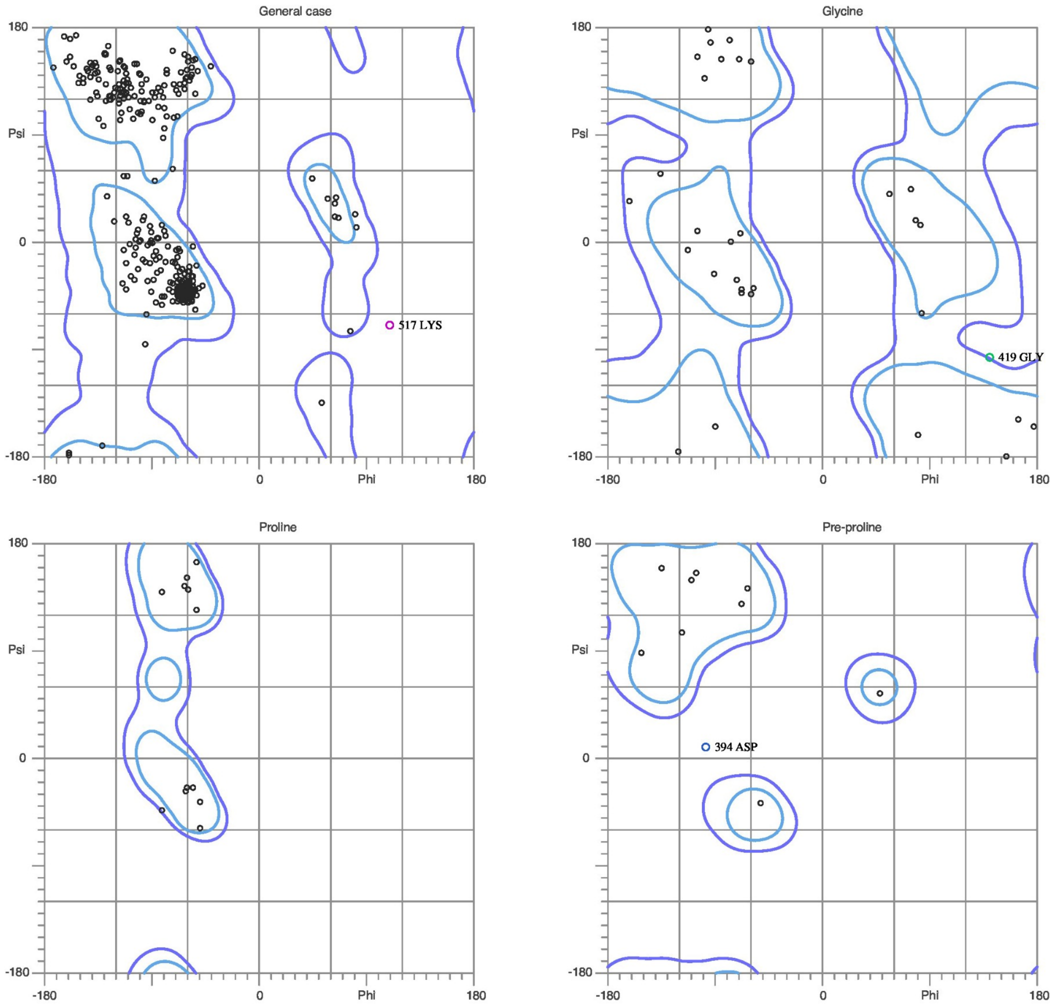

96.2% (405/421) of all residues were in favored (98%) regions.
99.3% (418/421) of all residues were in allowed (>99.8%) regions.

There were 3 outliers (phi, psi):
394 ASP (-98.8, 10.2)
419 GLY (140.5, -96.8)
517 LYS (109.5, -69.4)

Fig. S5. MolProbity Ramachandran plot for validating the 3d model of SbALDH18B1 protein
