# Supplementary material for: Aldehyde dehydrogenase superfamily in sorghum: genome-wide identification, evolution, and transcript profiling during development stages and stress conditions
Source: BMC Plant Biol. 2022 Jul 4;22:316. doi: 10.1186/s12870-022-03708-4 (PMC9252066; doi:10.1186/s12870-022-03708-4)
Supplement: Supplementary file 2 — Additional file 2: Appendix 1. All the sequences are used for the construction of phylogenetic tree. [file 12870_2022_3708_MOESM2_ESM.pdf]

Appendix 1. All the sequences are used for the construction of phylogenetic tree.

>SbALDH2B1a
MTAYERCRVLLRFADLIERHAEEIAALETWDNGKTLAQAAGAEVPMVARCIRYYAGWADKIHGLVVPGDG
AHHVQVLHEPVGVAGQIIPWNFPLLMFAWKVGPALACGNTVVLKTAEQTPLSALYVANLLHEAGLPEGVL
NVVSGFGPTAGAALCSHMGVDKLAFTGSTGTGQIVLELAARSNLKPVTLELGGKSPFVVMDDADVDQAVE
LAHQAVFFNQGQCCCAGSRTFVHERVYDEFVEKSKARALKRVVGDPFRNGVEQGPQIDGDQFNKILRYVQ
SGVDSGATLVTGGDRVGSRGFYIQPTVFADAKDDMKIAREEIFGPVQTILKFSGMEEVIRRANATHYGLA
AGVFTRSLDAANTLSRALRAGTVWVNCYDVFDATIPFGGYKMSGVGREKGVYALRNYLQTKAVVTPIKDP
AWL
>SbALDH2B1b
MAARRAASSVLSRFLLTRPSPSPSPASAASPAGKSALLGAGGPLHRFSTAPAAAAATAEE
PIQPAVEVKHTQLLINGNFVDAASGKTFPTLDPRTGEVIARVAEGDSEDIDRAVAAARRA
FDEGPWPRMTAYERCRVLLRFADLIERHAEEIAALETWDNGKTLAQAAGAEVPMVARCIR
YYAGWADKIHGLVVPGDGAHHVQVLHEPVGVAGQIIPWNFPLLMFAWKVGPALACGNTVV
LKTAEQTPLSALYVANLLHEAGLPEGVLNVVSGFGPTAGAALCSHMGVDKLAFTGSTGTG
QIVLELAARSNLKPVTLELGGKSPFVVMDDADVDQAVELAHQAVFFNQGQCCCAGSRTFV
HERVYDEFVEKSKARALKRVVGDPFRNGVEQGPQIDGDQFNKILRYVQSGVDSGATLVTG
GDRVGSRGFYIQPTVFADAKDDMKIAREEIFGPVQTILKFSGMEEVIRRANATHYGLAAG
VFTRSLDAANTLSRALRAGTVWVNCYDVFDATIPFGGYKMSGVGREKGVYALRNYLQTKA
VVTPIKDPAWL
>SbALDH2B1c
MAARRAASSVLSRFLLTRPSPSPSPASAASPAGKSALLGAGGPLHRFSTAPAAAAATAEE
PIQPAVEVKHTQLLINGNFVDAASGKTFPTLDPRTGEVIARVAEGDSEDIDRAVAAARRA
FDEGPWPRMTAYERCRVLLRFADLIERHAEEIAALETWDNGKTLAQAAGAEVPMVARCIR
YYAGWADKIHGLVVPGDGAHHVQVLHEPVGVAGQIIPWNFPLLMFAWKVGPALACGNTVV
LKTAEQTPLSALYVANLLHEAGLPEGVLNVVSGFGPTAGAALCSHMGVDKLAFTGSTGTG
QIVLELAARSNLKPVTLELGGKSPFVVMDDADVDQAVELAHQAVFFNQGQCCCAGSRTFV
HERVYDEFVEKSKARALKRVVGDPFRNGVEQGPQIDGDQFNKILRYVQSGVDSGATLVTG
GDRVGSRGFYIQPTVFADAKDDMKIAREEIFGPVQTILKFSGMEEVIRRANATHYGLAAG
VFTRSLDAANTLSRALRAGTVWVNCYDVFDATIPFGGYKMSGVGREKGVYALRNYLQTKA
VVTPIKDPAWL
>SbALDH2B2a
MASAAAATRRAAASLASRRRLSGSFPLVAAGRRAAVPSALRMPDGMRGLLPGVLQRFSTA
AAVEEPITPSVQVNYTKLLINGNFVDAASGKTFPTLDPRTGEVIAHVAEGDAEDINRAVA
AARKAFDEGPWPKMTAYERSRILLRFADLIEKHNDELAALETWDNGKPYEQAAHIEVPMV
ARLMRYYAGWADKIHGLVVPADGPHHVQILHEPIGVAGQIIPWNFPLLMFAWKVGPALAC
GNTLVLKTAEQTPLSALYISKLLHEAGLPEGVVNVVSGFGPTAGAALASHMDVDKLAFTG
STDTGKVVLELAARSNLKTVTLELGGKSPFIIMDDADIDHAVELAHFALFFNQGQCCCAG
SRTFVHERVYDEFVEKAKARALKRVVGDPFRKGVEQGPQIDDEQFNKILRYIRSGVDSGA
NLVTGGDRLGEKGYYIQPTIFSDVQDGMKIAQEEIFGPVQSILKFKDLNEVIKRANASPY
GLAAGVFTNSLDTANTLTRALRAGTVWINCFDIFDAAIPFGGYKMSGIGREKGIDSLKNY
LQVKAVVTPIKNAAWL
>SbALDH2B2b
MARRAASSLVSRCLLARASAPAAPPAVPSALRRPDGMRGLLPGVLQRFSTAAAVEEPITP
SVQVNYTKLLINGNFVDAASGKTFPTLDPRTGEVIAHVAEGDAEDINRAVAAARKAFDEG
PWPKMTAYERSRILLRFADLIEKHNDELAALETWDNGKPYEQAAHIEVPMVARLMRYYAG

WADKIHGLVVPADGPHHVQILHEPIGVAGQIIPWNFPLLMFAWKVGPALACGNTLVLKTA
EQTPLSALYISKLLHEAGLPEGVVNVVSGFGPTAGAALASHMDVDKLAFTGSTDTGKVVL
ELAARSNLKTVTLELGGKSPFIIMDDADIDHAVELAHFALFFNQGQCCCAGSRTFVHERV
YDEFVEKAKARALKRVVGDPFRKGVEQGPQIDDEQFNKILRYIRSGVDSGANLVTGGDRL
GEKGYYIQPTIFSDVQDGMKIAQEEIFGPVQSILKFKDLNEVIKRANASPYGLAAGVFTN
SLDTANTLTRALRAGTVWINCFDIFDAAIPFGGYKMSGIGREKGIDSLKNYLQVKAVVTP
IKNAAWL
>SbALDH2C1a
MATTNGSSKVFEVPKVEVRFTKLFIDGKFVDAVSGKTFETRDPRTGEVIATVAEGDKADV
DLAVKAAREAFDNGPWPRMTGYERGRILHKFADLIDEHVEELAMLDTVDAGKLFLVGKVR
DIPGAAHLLRYYAGAADKIHGETLKMAQRMHGYTLKEPVGVVGHIVPWNYPSTMFFFKVG
PALAAGCTVVVKPAEQTPLSALFYAHLAREAGVPDGVLNVVPGFGPTAGAAVASHMDVDK
VSFTGSTEVGRVVMKAAAESNLKPVSLELGGKSPVIVFDDADLDMAVNLVNLATYTNKGE
ICVAGTRIYVQEGIYDAFVEKAAELAKKSVVGDPFNPRVNQGPQVDKDQYQKVLKYIDMG
KREGATLVTGGKPCGDKGYYIEPTIFTDVKDNMTIAQDEIFGPVMALMKFKTVEEVIQKA
NNTRYGLAAGIVTKNIDIANTVSRSIRAGAIWINCYFAFDPDAPFGGYKMSGFGKDMGMD
ALEKYLQTKTVVTPLYNTPWL
>SbALDH2C1b
MATTNGSSKVFEVPKVEVRFTKLFIDGKFVDAVSGKTFETRDPRTGEVIATVAEGDKADV
DLAVKAAREAFDNGPWPRMTGYERGRILHKFADLIDEHVEELAMLDTVDAGKLFLVGKVR
DIPGAAHLLRYYAGAADKIHGETLKMAQRMHGYTLKEPVGVVGHIVPWNYPSTMFFFKVG
PALAAGCTVVVKPAEQTPLSALFYAHLAREAGVPDGVLNVVPGFGPTAGAAVASHMDVDK
VSFTGSTEVGRVVMKAAAESNLKPVSLELGGKSPVIVFDDADLDMAVNLVNLATYTNKGE
ICVAGTRIYVQEGIYDAFVEKAAELAKKSVVGDPFNPRVNQGPQVDKDQYQKVLKYIDMG
KREGATLVTGGKPCGDKGYYIEPTIFTDVKDNMTIAQDEIFGPVMALMKFKYIYASSLNI
LSV
>SbALDH2C2a
MASNGSNGKAAPAAGVVVPEIKFTKLFINGEFVDAASGKTFETRDPRTGDVLAHVAEADK
ADVDLAVKSARDAFEHGKWPRMSGYERGRIMSKLADLVEQHTEELAALDGADAGKLVLLG
KIIDIPAATQMLRYYAGAADKIHGEVLRVSGKYQGYTLKEPVGVVGVIIPWNFPTMMFFL
KVSPALAAGCTVVVKPAEQTPLSALYYAHLAKLAGVPDGVINVVPGFGPTAGAALTSHMD
VDSVAFTGSTEVGRLIMESAARSNLKMVSLELGGKSPLIVFDDADVDMAVNLSRLAIFYN
KGEVCVAGSRVYVQEGIYDEFVKKAVEAAQNWKVGDPFDVTTNMGPQVDKDQFERVLKYI
EHGKSEGATLLTGGKPAADKGYYIEPTIFVDVTEDMKIAQEEIFGPVMSLMKFRSVDEVI
EKANCTKYGLAAGIVTKSLDIANRVSRSVRAGTVWVNCYYAFDPDAPFGGYKMSGFGRDQ
GLAAMDKYLQVKSVITALPDSPWY
>SbALDH2C2b
MSGYERGRIMSKLADLVEQHTEELAALDGADAGKLVLLGKIIDIPAATQMLRYYAGAADK
IHGEVLRVSGKYQGYTLKEPVGVVGVIIPWNFPTMMFFLKVSPALAAGCTVVVKPAEQTP
LSALYYAHLAKLAGVPDGVINVVPGFGPTAGAALTSHMDVDSVAFTGSTEVGRLIMESAA
RSNLKMVSLELGGKSPLIVFDDADVDMAVNLSRLAIFYNKGEVCVAGSRVYVQEGIYDEF
VKKAVEAAQNWKVGDPFDVTTNMGPQVDKDQFERVLKYIEHGKSEGATLLTGGKPAADKG
YYIEPTIFVDVTEDMKIAQEEIFGPVMSLMKFRSVDEVIEKANCTKYGLAAGIVTKSLDI
ANRVSRSVRAGTVWVNCYYAFDPDAPFGGYKMSGFGRDQGLAAMDKYLQVKSVITALPDS
PWY
>SbALDH2C2c
MSKLADLVEQHTEELAALDGADAGKLVLLGKIIDIPAATQMLRYYAGAADKIHGEVLRVS
GKYQGYTLKEPVGVVGVIIPWNFPTMMFFLKVSPALAAGCTVVVKPAEQTPLSALYYAHL

AKLAGVPDGVINVVPGFGPTAGAALTSHMDVDSVAFTGSTEVGRLIMESAARSNLKMVSL
ELGGKSPLIVFDDADVDMAVNLSRLAIFYNKGEVCVAGSRVYVQEGIYDEFVKKAVEAAQ
NWKVGDPFDVTTNMGPQVDKDQFERVLKYIEHGKSEGATLLTGGKPAADKGYYIEPTIFV
DVTEDMKIAQEEIFGPVMSLMKFRSVDEVIEKANCTKYGLAAGIVTKSLDIANRVSRSVR
AGTVWVNCYYAFDPDAPFGGYKMSGFGRDQGLAAMDKYLQVKSVITALPDSPWY
>SbALDH2C3
MGSESNRGGAADRMVAEEEEKKKRGGRLFEMPEIRFTKLFINGCFVDAASGRTFETRDPR
TGDVIATVAEADKDDVDSAVRAARDAFDHGEWPRMSGSERGRIMARFADLVEQHADELAA
LESLDAGKHPAITKAVDIGNAAGSLRYFAGAADKIHGETLKMQAQFQGYTLREPLGVAGI
IIPWNFPSTMFAIKVAPALAAGCAMVVKPAEQTPLSALFFAQLAKRAGVPDGVVNVVPGF
GPTAGAALASHMDVDMVTFTGSTEVGRLIMKASAESNLKPVYLELGGKSPLIIFDDADVD
MAVELAVSANFFNKGEACVAASRVYVQEGMYSRFEEKLAERMKSWVVGDPFSDARANQGP
QVDKAQYERVLSYIDHGKREGATLLTGGGRPAACGHKGYYIEPTVFTNVTEDMVIAKEEI
FGPVMCLIKFKTVEEAIWRANDSRYGLGAGVVTRDVDVANRVVRSVRAGVVWVNCYFAMG
SDCPFGGRKMSGFGKDEGMHALDKYLAIKSVVTPLRASPWM
>SbALDH3E1
MGSVPEETAALDNIGDLVSDLREVYESGRTQDMEWRQSQLRGLVRLLEDEEEAIFDALHE
DLGKHRVEAFRDEVGVLKKSVVDKLQNLKKWAAPEKAHTPLVAFPATALVVPEPLGVVLI
FSCWNLPIGLALEPLSGALAAGNAAVVKPSELAPSTSAFLAANIPKYLDSKAVKVVEGGP
EVGDKLMDYPWDKVLFTGSSRVGRLIMTKAAKHLTPVALELGSKCPCIVDWLDSNRDSQI
AVNRIIGAKWSTCSGQACIAIDYLLVEEEFAPILIEMLKSTLERFFTRPEYMARILNEKH
FQRLSGFLADRRVASSVVHGGHFNPKTLSIEPTLLLNPPLDSDIMTEEIFGPLLPIITVK
KIEDSIKFLKSKPKPLAIYAFTRNEKLKQRIIDETSSGSVTFNDAIVQYGLDSIPFGGVG
HSGFGQYHGKYTFEMFSHKKAVLKRSLLVEFMFRYPPWDETKIGMLRRVYRFDYVSLFLA
IVGLRR
>SbALDH3E2a
MGATATEAAEDGAAVAGGALGLGLGETVRELREAYECGRTRSVAWRRAQLRGLLRLLEEK
EAEAFQALRTDLGKHHAEAYRDEVGVLIKSTNGALQQLGKWMTPEKVWVPLIAFPATAQV
VPEPLGVVLVFSCWNVPLGLSLEPLIGAIAAGNAVALKPSEISPCTARFLADNIGRYMDP
SAVKVILGGPEVGEQLMEHRWDKVLFTGSPRIARAVMAAASRHLTPVALELGGKCPCIFD
AMGSARDLQISVNRIIAGKWSSCAGQACIAIDYVLVEERFAPILIKVLKSTLKRFFQETD
HMARIVNERHFERLSSLLKDRSVAPSVLHGGSMDAKNLYIEPTILLNPPLDSAIMTEEIF
GPLLPIITVKKIEDSIAFVKAMPKPLAIYAFTQDAALRRRIVDETSSGCVTFNDAVVQYA
IDGLPFGGVGQSGFGQYHGKYSFEMFSHKKAVMKRGYLLELTLRYPPWDESKVTMMRYLY
RFNYFAFVLSFLGLRR
>SbALDH3E2b
MGATATEAAEDGAAVAGGALGLGLGETVRELREAYECGRTRSVAWRRAQLRGLLRLLEEK
EAEAFQALRTDLGKHHAEAYRDEVGVLIKSTNGALQQLGKWMTPEKVWVPLIAFPATAQV
VPEPLGVVLVFSCWNVPLGLSLEPLIGAIAAGNAVALKPSEISPCTARFLADNIGRYMDP
SAVKVILGGPEVGEQLMEHRWDKVLFTGSPRIARAVMAAASRHLTPVALELGGKCPCIFD
AMGSARDLQISVNRIIAGKWSSCAGQACIAIDYVLVEERFAPILIKVLKSTLKRFFQETD
HMARIVNERHFERLSSLLKDRSVAPSVLHGGSMDAKNLYIEPTILLNPPLDSAIMTEEIF
GPLLPIITVKKIEDSIAFVKAMPKPLAIYAFTQDAALRRRIVDETSSGCVTFNDAVVQYA
IDGLPFGGVGQSGFGQYHGKYSFEMFSHKKAVMKRGYLLELTLRYPPWDESKVTMMRYLY
RFNYFAFVLSFLGLRR
>SbALDH3H1
MAEETVRELRASFAAGRTRSPEWRAEQLRGLIRMIDEKEAEISAALHEDLAKPHMESYLH
EISLTKSSCKFAINGLKNWMKPEKVPASITTFPSSAQIVPEPLGVVLIISAWNYPFILSI

DPVIGAIAAGNAVVLKPSEIAPATSSVLANLLPKYVDNSCIKVVEGGVPETTALLEQRWD
KIFYTGSGTVARIVMAAAAKHLTPVALELGGKSPVVVDSNVDLHVAVKRIVVGKWGCNNG
QACIAPDYIITTKSFAPELVASLKRVLVRFYGEDPLQSADLSRIVNSKQFKRLQDLIEEK
RVADKIVFGGEADEEQLKIAPTVLLDVPQDSAIMTGEIFGPLLPIVTVEKIEESFNLINA
KPKPLAAYLFTKNKKLQEEFVANVPAGGMLVNDTALHLANPYLPFGGVGDSGMGCYHGKF
GFDCFSHKKAVLIRGFGGEANARYPPYTTEKQKILRGLINGSFIALILALLGFPREKR
>SbALDH3H2
MDAAVAAAAEARERVRASFATGRTRAAAWREAQLRGLLRMAAEMEDDVCAALRADLAKPQ
TESYVHEISLVTTSCKFALKNLKKWMKPQKVPGGVLTFPSAARVTAEPLGVVLVISAWNY
PFLLAIDPVVGAIAAGNAVVLKPSEVAPATSSLLAELLPRYVDASCVRVVQGGIPETTAL
LELTWDKIFYTGNSKVGRIVMSYAAKHLTPVVLELGGKCPVVVDSNVNIHVAAKRIAAGK
WGCNSGQACISPDYIVTTKSLAPKLLESLKKVLRKFYGEDPLRSPDLSRIVNSNHFNRLR
TLMDDGTVAGKIAFGGQSDEQQLRIAPTLLLDVPLDSAIMKEEIFGPLLPIITVDKISES
FAVINSVSKPLAAYLFTNDSRLKQQFERNISSGGMIFNDTAIHLTNPHLPFGGVGESGMG
AYHGAFSFDAFTHRKAVLDRSSFLGEARARYPPYTPAKLGILRGVLKGNPLAMVLAAVGY
TGGRRRA
>SbALDH5F1a
MAMAMMTLRRAVALGARHIPAVAAASSRIVPLRHMSAGAGAAVEKIRAAGLLRTQGLIAG
KWVDAYDGKTIEVQNPATGEVLANVPCMGSRETSDAIASAHSTFYSWSKLTAGERSKALR
KWYDLIISHKEELALLMTLEQGKPMKEALGEVNYGASFIEYFAEEAKRIYGDIIPPTLSD
RRLLVLKQPVGVVGAITPWNFPLAMITRKVGPALACGCTVVVKPSEFTPLTALAAADLAL
QAGIPAGALNVVMGNAPEIGDALLQSTQVRKITFTGSTAVGKKLMAGSANTVKKVSLELG
GNAPCIVFDDADIDVAVKGSLAAKFRNSGQTCVCANRILVQEGIYEKFATAFIKAVQSLK
VGNGLEESTSQGPLINEAAVQKVEKFINDATSKGANIMLGGKRHSLGMTFYEPTVVGNVS
NDMLLFREEVFGPVAPLIPFKTEEEAVHMANDTNAGLAAYIFTKSIPRSWRVSESLEYGL
VGVNEGIISTEVAPFGGVKQSGLGREGSKYGIDEYLELKYICMGNLG
>SbALDH5F1b
MAMAMMTLRRAVALGARHIPAVAAASSRIVPLRHMSAGAGAAVEKIRAAGLLRTQGLIAG
KWVDAYDGKTIEVQNPATGEVLANVPCMGSRETSDAIASAHSTFYSWSKLTAGERSKALR
KWYDLIISHKEELALLMTLEQGKPMKEALGEVNYGASFIEYFAEEAKRIYGDIIPPTLSD
RRLLVLKQPVGVVGAITPWNFPLAMITRKVGPALACGCTVVVKPSEFTPLTALAAADLAL
QAGIPAGALNVVMGNAPEIGDALLQSTQVRKITFTGSTAVGKKLMAGSANTVKKVSLELG
GNAPCIVFDDADIDVAVKGSLAAKFRNSGQTCVCANRILVQEGIYEKFATAFIKAVQSLK
VGNGLEESTSQGPLINEAAVQKVEKFINDATSKGANIMLGGKRHSLGMTFYEPTVVGNVS
NDMLLFREEVFGPVAPLIPFKTEEEAVHMANDTNAGLAAYIFTKSIPRSWRVSESLEYGL
VGVNEGIISTECCSNSDMSVLHVQLQLKYICMGNLG
>SbALDH5F1c
MSAGAGAAVEKIRAAGLLRTQGLIAGKWVDAYDGKTIEVQNPATGEVLANVPCMGSRETS
DAIASAHSTFYSWSKLTAGERSKALRKWYDLIISHKEELALLMTLEQGKPMKEALGEVNY
GASFIEYFAEEAKRIYGDIIPPTLSDRRLLVLKQPVGVVGAITPWNFPLAMITRKVGPAL
ACGCTVVVKPSEFTPLTALAAADLALQAGIPAGALNVVMGNAPEIGDALLQSTQVRKITF
TGSTAVGKKLMAGSANTVKKVSLELGGNAPCIVFDDADIDVAVKGSLAAKFRNSGQTCVC
ANRILVQEGIYEKFATAFIKAVQSLKVGNGLEESTSQGPLINEAAVQKVEKFINDATSKG
ANIMLGGKRHSLGMTFYEPTVVGNVSNDMLLFREEVFGPVAPLIPFKTEEEAVHMANDTN
AGLAAYIFTKSIPRSWRVSESLEYGLVGVNEGIISTEVAPFGGVKQSGLGREGSKYGIDE
YLELKYICMGNLG
>SbALDH5F1d
MTLEQGKPMKEALGEVNYGASFIEYFAEEAKRIYGDIIPPTLSDRRLLVLKQPVGVVGAI

TPWNFPLAMITRKVGPALACGCTVVVKPSEFTPLTALAAADLALQAGIPAGALNVVMGNA
PEIGDALLQSTQVRKITFTGSTAVGKKLMAGSANTVKKVSLELGGNAPCIVFDDADIDVA
VKGSLAAKFRNSGQTCVCANRILVQEGIYEKFATAFIKAVQSLKVGNGLEESTSQGPLIN
EAAVQKVEKFINDATSKGANIMLGGKRHSLGMTFYEPTVVGNVSNDMLLFREEVFGPVAP
LIPFKTEEEAVHMANDTNAGLAAYIFTKSIPRSWRVSESLEYGLVGVNEGIISTEVAPFG
GVKQSGLGREGSKYGIDEYLELKYICMGNLG
>SbALDH5F1e
MAMAMMTLRRAVALGARHIPAVAAASSRIVPLRHMSAGAGAAVEKIRAAGLLRTQGLIAG
KWVDAYDGKTIEVQNPATGEVLANVPCMGSRETSDAIASAHSTFYSWSKLTAGERSKALR
KWYDLIISHKEELALLMTLEQGKPMKEALGEVNYGASFIEYFAEEAKRIYGDIIPPTLSD
RRLLVLKQPVGVVGAITPWNFPLAMITRKVGPALACGCTVVVKPSEFTPLTALAAADLAL
QAGIPAGALNVVMGNAPEIGDALLQSTQVRKITFTGSTAVGKKLMAGSANTVKKVSLELG
GNAPCIVFDDADIDVAVKGSLAAKFRNSGQTCVCANRILVQEGIYEKFATAFIKAVQSLK
VGNGLEESTSQGPLINEAAVQKVEKFINDATSKGANIMLGGKRHSLGMTFYEPTVVGNVS
NDMLLFREEVFGPVAPLIPFKTEEEAVHMANDTNAGLAAYIFTKSIPRSWRVSESLEYGL
VGVNEGIISTEVAPFGGVKQSGLGREGSKYGIDEYLELKYICMGNLG
>SbALDH6B1
MLRAALFRSAPGLRRSPAMAAAAAPFSTAAAAAAWLSNGPASTPPRVRLLIGGEFVESRA
DEHVDVTNPATQEVVSRIPLTTADEFRAAVDAARTAFPGWRNTPVTTRQRVMFKFQELIR
ANMDKLAENITTEQGKTLKDAWGDVFRGLEVVEHACGMGTLQMGEYVSNVSNGIDTFSIR
EPLGVCAGICPFNFPAMIPLWMFPIAVTCGNTFVLKPSEKDPGAAMMLAELAMEAGLPKG
VLNIVHGTNDVVNNICDDEDIKAVSFVGSNTAGMHIYSRASAAGKRVQCNMGAKNHAIIL
PDADRDATLNALIAAGFGAAGQRCMALSTAVFVGGSESWEDELVNRASGLIVNSGMVNDA
DLGPVISRQAKDRICKLVQSGVDSGARILLDGRKIVVPQYEDGNFVGPTILADVKSDMEC
YKEEIFGPVLLLMKAESLDDAIQIINRNKYGNGASIFTTSGVYARKFQTDIEAGQVGINV
PIPVPLPFFSFTGSKASFAGDLNFYGKAGVQFFTQIKTITQQWKESPAQRVSLSMPTSQK
>SbALDH7B1
MGAFAKEEHQFLAELGLAQRNPGAFVCGAWGGSGPAVTSTSPTNNQVIAEVVEASVQDYE
EGMRACFDAAKTWMAFPAPKRGEIVRQIGDALRAKLHHLGRLVSLEMGKILPEGIGEVQE
IIDMCDYAVGLSRQLNGSIIPSERPNHMMMEVWNPLGVVGVITAFNFPCAVLGWNACIAL
VCGNCVVWKGAPTTPLITIAMTKIVASVLEKNNLPGAIFTSFCGGTEIGQAIAVDTRIPL
VSFTGSTRAGLMVQQQVNARFGKCLLELSGNNAIIVMDDADIQLAVRSVLFAAVGTAGQR
CTTCRRLILHESIYQTFLDQLVEVYKQVRIGDPLEKGTLLGPLHTPASKENFLKGVQTIK
SQGGKILFGGSAIESEGNFVQPTIVEITPSAAVVKEELFGPVLYVMKFQSLKEAIEINNS
VPQGLSSSIFTKRPEIIFKWLGPHGSDCGIVNVNIPTNGAEIGGAFGGEKATGGGREAGS
DSWKQYMRRATCTINYGSELPLAQGINFG
>SbALDH10A1
MAPPQTVPRRGLFIGGAWREPCLGRRLPVVNPATEATIGDIPAGTAEDVEIAVAAARDAF
SRDGGRHWSRASGAMRANFLRAIAAKIKDRKSELALLETLDSGKPLDEASADMDDVAACF
EYYADLAEALDGKQRSPISLPMENFKSYVLKEPIGVVGLITPWNYPLLMATWKVAPALAA
GCTAVLKPSELASVSCLELGAICMEIGLPPGVFNVITGLGPEAGAPLSSHPHVDKIAFTG
STETGKRIMTSAAQMVKPVSLELGGKSPLIVFDDIRDIDKAVEWTMFGIFANAGQVCSAT
SRLLLHEKIAKKFLDRLVAWAKNIKVSDPLEEGCRLGSVVSEGQYEKIKKFISTARSEGA
TILYGGARPQHLKRGFFLEPTIITDISTSMQIWREEVFGPVICVKEFRRESEAVELANDT
QYGLAGAVISDDQERCERISKALQSGIIWINCSQPCFVQAPWGGNKRSGFGRELGEWGLD
NYMTVKQVTKYCSDEPWGWYQPPSKL
>SbALDH10A2a
MATPAMVPLRQLFVDGEWRPPAQGRRLPVVNPTTEAHIGEIPAGTAEDVDAAVAAARAAL

KRNRGRDWARAPGAVRAKYLRAIAAKVIERKPELAKLEALDCGKPYDEAVWDMDDVAGCF
EYFADQAEALDKRQNSPVSLPMETFKCHLRREPIGVVGLITPWNYPLLMATWKVAPALAA
GCTAVLKPSELASVTCLELADICKEVGLPSGVLNIVTGLGPDAGAPLSGHPDVDKVAFTG
SFETGKKIMAAAAPMVKPVTLELGGKSPIVVFDDVDIDKAVEWTLFGCFWTNGQICSATS
RLLIHTKIAKEFNERMVAWAKNIKVSDPLEEGCRLGPVVSEGQYEKIKKFISNAKSEGAT
ILTGGVRPAHLEKGFFIEPTIITDITTSMEIWREEVFGPVLCVKEFSTEDEAIELANDTQ
YGLAGAVISGDRERCQRLSEEIDAGCIWVNCSQPCFCQAPWGGNKRSGFGRELGEGGIDN
YLSVKQVTEYISDEPWGWYQSPSKL
>SbALDH10A2b
MDDVAGCFEYFADQAEALDKRQNSPVSLPMETFKCHLRREPIGVVGLITPWNYPLLMATW
KVAPALAAGCTAVLKPSELASVTCLELADICKEVGLPSGVLNIVTGLGPDAGAPLSGHPD
VDKVAFTGSFETGKKIMAAAAPMVKPVTLELGGKSPIVVFDDVDIDKAVEWTLFGCFWTN
GQICSATSRLLIHTKIAKEFNERMVAWAKNIKVSDPLEEGCRLGPVVSEGQYEKIKKFIS
NAKSEGATILTGGVRPAHLEKGFFIEPTIITDITTSMEIWREEVFGPVLCVKEFSTEDEA
IELANDTQYGLAGAVISGDRERCQRLSEEIDAGCIWVNCSQPCFCQAPWGGNKRSGFGRE
LGEGGIDNYLSVKQVTEYISDEPWGWYQSPSKL
>SbALDH11A1
MALAGTGVFAEILDGEVYRYYADGEWRSSASGKSVAIVNPTTRKTQYRVQACTQEEVNKV
MDAAKVAQKAWARTPLWKRAELLHKAAAILKEHKAPIAECLVKEIAKPAKDAVSEVVRSG
DLISYTAEEGVRILGEGKLLVSDSFPGNERNKYCLSSKIPLGVVLAIPPFNYPVNLAVSK
IGPALIAGNSLVLKPPTQGAVAALHMVHCFHLAGFPKGLISCVTGKGSEIGDFLTMHPGV
NCISFTGGDTGIAISKKAGMVPLQMELGGKDACIVLEDADLDLVAGNIVKGGFSYSGQRC
TAVKVVLIMESIADAVVQKVNAKLAKLKVGPPEDDSDITPVVTESSANFIEGLVMDAKEK
GATFCQEYRREGNLIWPLLLDHVRPDMRIAWEEPFGPVLPVIRINSVEEGIHHCNASNFG
LQGCIFTRDINKAILISDAMETGTVQINSAPARGPDHFPFQGLKDSGIGSQGITNSINMM
TKVKSTVINLPSPSYTMG
>SbALDH12A1
MSRLLSRRQIDAVRRSAPLACVSRWLHTPSFATVSPHEVSGSSPAEVQNFVQGSWTASAN
WNWIVDPLNGDQFIKVAEVQGTEIKPFVESLSKCPKHGLHNPLKAPERYLMYGDISAKAA
HMLGQPAVSDFFAKLIQRVSPKSYQQALAEVQVSQKFLENFCGDQVRFLARSFAVPGNHL
GQRSNGYRWPYGPVAIITPFNFPLEIPLLQVMGALYMGNKPVLKVDSKVSIVMEQMIRLL
HDCGLPAEDMDFINSDGVTMNKLLLEANPKMTLFTGSSRVAEKLAADLKGRVKLEDAGFD
WKILGPDVQEVDYVAWVCDQDAYACSGQKCSAQSVLFMHKNWSSSGLLEKMKKLSERRKL
EDLTIGPVLTVTTEAMIEHMNNLLKIQGSKVLFGGEPLANHSIPKIYGAMKPTAVFVPLE
EILKSGNFELVTKEIFGPFQVVTEYSEDQLELVLEACERMNAHLTAAVVSNDPLFLQDVL
GRSVNGTTYAGIRARTTGAPQNHWFGPAGDPRGAGIGTPEAIKLVWSCHREIIYDVGPVP
KSWALPSAT
>SbALDH18B1
MGRGGIGGAVGMAMENADSARAFVKDVKRIIIKVGTAVVTGQNGRLAMGRLGSLCEQVKQ
LNFQGYEVILVTSGAVGVGRQRLQYRKLIHSSFADLQNPQMNFDGKACAAVGQSGLMAIY
DTLFSQLDVTSSQLLVTDRDFKDPSFGDQLRETVFALLNLKVIPLFNENDAISTRRQSDE
DSSGVFWDNDSLAALLAAELNADLLIMLSDVEGLYSGPPSDPQSKIIHTYVNEKHGKLIS
FGEKSNVGRGGMQAKVAAAANAASKGVPVVIASGFATDSIIKVLKGEKIGTLFHNEANLW
ECSKEATAREMAVAARDCSRRLQKLSSDERKKILLDIADALEANEGAIRSENEADVEAAQ
GAGYEKSLVARMTLKPGKITNLARSIRAIADMEDPISHTLKRTEVAKDLVFEKAYCPLGV
LLIIFESRPDALVQIASLAIRSGNGLLLKGGKEAMRSNAILHKVITGVIPDIVGKKLIGH
VTSKDEIADLLALDDVIDLVIPRGSKNLVSQIKATTKIPVLGHADGICHVYIDKSADMDM
AKRIVLDAKVDYPAACNAMETLLVHKDLNKSEGLDDLLVELEKEGVVIYGGPVAHDKLKV

PKVDSFRHEYSSMACTLEFVDDVQSAIDHINRYGSAHTDCIITTDESAAEAFLQQVDSAA
VFHNASTRFCDGTRFGLGAEVGISTGRIHARGPVGVDGLLTTRCILRGSGQVVNGDKGVV
YTHKDLPLQ
>SbALDH18B2a
MATADPTRTFMKDVKRVIIKLGTAVVTRHDGRLALGRLGALCEQVKELNALGYEVIIVTS
GAVGVGKQRLKYRKLVNSSFADLQKPQMELDGKACAAVGQSGLMALYDMLFTQLDVSSSQ
LLVTDSDFENPNFRERLRETVESLLDLKVVPIFNENDAISTRKAPYEDSSGIFWDNDSLA
GLLAIELKADLLVLLSDVDGLYSGPPSEPQSKIIHTYIKEKHHNEITFGDKSRVGRGGMT
AKVKAAFVASNSGTPVVITSGFASQSIVRVLQGEKIGTLFHKDASLWEPSKDVSAREMAL
GARESSRRLQNLSSDERKKILLDVADALEENVDLIRTENEIDVSAAQEAGYEPSLVARLT
LKPGKIASLAKSIRTLAYMEDPINQIIKRTEVAEDLVLEKTSCPLGVLLIVFESRPDALV
QIASLAIRSGNGLLLKGGKEAMRSNTILHKVITSAIPSNVGEKLIGLVTSRDEIADLLKL
DDVIDLVIPRGSNKLVSQIKSSTKIPVLGHADGICHVYIDKSADMNMAKRIVLDAKIDYP
AACNAMETLLVHKDLINAPGLDDLLLALKTEGVAIYGGPVAHELLCIPKADSLHHEYSSM
ACTIEFVDDVQSAIDHIHRYGSAHTDCIVTTDDKVAETFLRQVDSAAVFYNASTRFSDGA
RFGLGAEVGISTGRIHARGPVGVEGLLTTRWIMRGSGQVVNGDKDIAYTHKNLPLQ
>SbALDH18B2b
MATADPTRTFMKDVKRVIIKLGTAVVTRHDGRLALGRLGALCEQVKELNALGYEVIIVTS
GAVGVGKQRLKYRKLVNSSFADLQKPQMELDGKACAAVGQSGLMALYDMLFTQLDVSSSQ
LLVTDSDFENPNFRERLRETVESLLDLKVVPIFNENDAISTRKAPYEDSSGIFWDNDSLA
GLLAIELKADLLVLLSDVDGLYSGPPSEPQSKIIHTYIKEKHHNEITFGDKSRVGRGGMT
AKVKAAFVASNSGTPVVITSGFASQSIVRVLQGEKIGTLFHKDASLWEPSKDVSAREMAL
GARESSRRLQNLSSDERKKILLDVADALEENVDLIRTENEIDVSAAQEAGYEPSLVARLT
LKPGKIASLAKSIRTLAYMEDPINQIIKRTEVAEDLVLEKTSCPLGVLLIVFESRPDALV
QIASLAIRSGNGLLLKGGKEAMRSNTILHKVITSAIPSNVGEKLIGLVTSRDEIADLLKL
DDVIDLVIPRGSNKLVSQIKSSTKIPVLGHADGICHVYIDKSADMNMAKRIVLDAKIDYP
AACNAMETLLVHKDLINAPGLDDLLLALKTEGVAIYGGPVAHELLCIPKADSLHHEYSSM
ACTIEFVDDVQSAIDHIHRYGSAHTDCIVTTDDKVAETFLRQVDSAAVFYNASTRFSDGA
RFGLGAEVGISTGRIHARGPVGVEGLLTTRWIMRGSGQVVNGDKDIAYTHKNLPLQ
>SbALDH18B2c
MATADPTRTFMKDVKRVIIKLGTAVVTRHDGRLALGRLGALCEQVKELNALGYEVIIVTS
GAVGVGKQRLKYRKLVNSSFADLQKPQMELDGKACAAVGQSGLMALYDMLFTQLDVSSSQ
LLVTDSDFENPNFRERLRETVESLLDLKVVPIFNENDAISTRKAPYEDSSGIFWDNDSLA
GLLAIELKADLLVLLSDVDGLYSGPPSEPQSKIIHTYIKEKHHNEITFGDKSRVGRGGMT
AKVKAAFVASNSGTPVVITSGFASQSIVRVLQGEKIGTLFHKDASLWEPSKDVSAREMAL
GARESSRRLQNLSSDERKKILLDVADALEENVDLIRTENEIDVSAAQEAGYEPSLVARLT
LKPGKIASLAKSIRTLAYMEDPINQIIKRTEVAEDLVLEKTSCPLGVLLIVFESRPDALV
QIASLAIRSGNGLLLKGGKEAMRSNTILHKVITSAIPSNVGEKLIGLVTSRDEIADLLKL
DDVIDLVIPRGSNKLVSQIKSSTKIPVLGHADGICHVYIDKSADMNMAKRIVLDAKIDYP
AACNAMETLLVHKDLINAPGLDDLLLALKTEGVAIYGGPVAHELLCIPKADSLHHEYSSM
ACTIEFVDDVQSAIDHIHRYGSAHTDCIVTTDDKVAETFLRQVDSAAVFYNASTRFSDGA
RFGLGAEVGISTGRIHARGPVGVEGLLTTRWIMRGSGQVVNGDKDIAYTHKNLPLQ
>SbALDH18B2d
MATADPTRTFMKDVKRVIIKLGTAVVTRHDGRLALGRLGALCEQVKELNALGYEVIIVTS
GAVGVGKQRLKYRKLVNSSFADLQKPQMELDGKACAAVGQSGLMALYDMLFTQLDVSSSQ
LLVTDSDFENPNFRERLRETVESLLDLKVVPIFNENDAISTRKAPYEDSSGIFWDNDSLA
GLLAIELKADLLVLLSDVDGLYSGPPSEPQSKIIHTYIKEKHHNEITFGDKSRVGRGGMT
AKVKAAFVASNSGTPVVITSGFASQSIVRVLQGEKIGTLFHKDASLWEPSKDVSAREMAL

GARESSRRLQNLSSDERKKILLDVADALEENVDLIRTENEIDVSAAQEAGYEPSLVARLT
LKPGKIASLAKSIRTLAYMEDPINQIIKRTEVAEDLVLEKTSCPLGVLLIVFESRPDALV
QIASLAIRSGNGLLLKGGKEAMRSNTILHKVITSAIPSNVGEKLIGLVTSRDEIADLLKL
DDVIDLVIPRGSNKLVSQIKSSTKIPVLGHADGICHVYIDKSADMNMAKRIVLDAKIDYP
AACNAMETLLVHKDLINAPGLDDLLLALKTEGVAIYGGPVAHELLCIPKADSLHHEYSSM
ACTIEFVDDVQSAIDHIHRYGSAHTDCIVTTDDKVAETFLRQVDSAAVFYNASTRFSDGA
RFGLGAEVGISTGRIHARGPVGVEGLLTTRWIMRGSGQVVNGDKDIAYTHKNLPLQ
>SbALDH22A1
MAFWWPLLVLAAAYALCRLLLFLIPPTVPSIDVDASDVLAKEDSFIYIPRKGKAAQTDKV
QCYEPATMKYLGYFPALTPDEVKEHVAQARKAQKIWAKSSFKQRRQFLRILLKYILEHQD
LICEVSSRDTGKTMVDASLGEIMTTCEKITWLLDEGEKWLKPEYRSTGRSMLHKRAKVEF
YPLGVIGAIVSWNYPFHNVFNPMLAAVFSGNAAVIKVSEYASWSGCFYFRIIQAALSAVG
APENLVHVITGFAETGQALVSSVDKIIFVGSPGVGKMIMKGASETLIPVTLELGGKDSFI
VCEDVDLPSVVQVAVRAALQSSGQNCAGAERFYVHNDIYSAFVSQVAKIVKSICVGPPLS
GRYDMGAICMMEHSEKLQSLVNDALDKGAEIAVRGSFGNLGEDAVDQFFPPTVLVNVDHT
MKIMQEEAFGPILPIMKFNSDEEAIKLANDSKYGLGCAVFSGDQKRAIRIASQIHCGVAA
INDFASSYMCQSLPFGGVKDSGFGRFAGVEGLRACCLVKSVVEDRLWPYIKTVIPKPIQY
PVSEHGFEFQQLLVETLYGYSMWDRLRSLVNLIKMVTEQNSASVSNATTKKRR
>StALDH2B2
MAARRLSSLLSRSLHLPSASASLGRSHGVARHINRFSTAAAVEELITPPVQVNHTKLLIN
GQFVDSASGKTFPTLDPRTGEVIANVAEGDLEDVNRAVAAARKAFDEGPWPKMSAYERSR
IMLKFADLVEKHNDEIAALETWDNGKPYLQAAQAEVPSFVRLFRYYAGWADKIHGLTVPA
DGPYHVQTLHEPIGVAGQIIPWNFPLLMMAWKVGPALACGNTIVLKTAEQTPLTALYVAN
LFHEAGLPPGVLNIVSGFGPTAGAALASHMDVDKLAFTGSTETGQTVLQLAAKSNLKPVT
LELGGKSPFIICEDADVDHAVELAHFALFFNQGQCCCAGSRTYVHERVYDEFVEKAKARA
MRRVVGDPFKKGVEQGPQIDSEQFKKILRYIREGRDSSATLECGGDRIGSKGYFIQPTVF
SNVKEDMSIAQDEIFGPVQCVFKFKDIGEVIKRANNTRYGLAAGVFTKNIDTANTLTRGL
RAGTVWINCYDIFDAGIPFGGYKMSGTGREKGIYSLNNYLQVKAVVTPLKNPAWI
>StALDH2B6
MAARVFLSRSVHLLSKGKRSHLGRIAAYKYSTAAALEEPIKPTVNVDHTKLFINGQFVDS
ASGKTFPTLDPRTGEVIAHIAEGDAEDINRAVAAARKAFDEGPWPRMTAYERSKILLRLA
DLIEKHNDQIATLETWDTGKPYAQAAKIEVPMVVRLLRYYAGWADKIHGMTIPADGPYHV
QTLHEPIGVAGQIIPWNFPLLMFSWKIGPALACGNTIVLKTAEQTPLSALYVANLLQEAG
LPEGVLNIISGFGATAGASLCSHMDVDKLAFTGSTETGKTILELAAKSNLKPVTLELGGK
SPFIVYEDADIDTAVEQAHFALFFNQGQCCCAGSRTYVHEKVYDEFLEKAKARALKRVVG
DPFKSGTEQGPQIDSKQFDKIMKYIRSGVDSGATLETGGEQFGKKGYYIKPTVFSNVKDD
MLIAQDEIFGPVQSILKFKDLDEVVRRANSSRYGLAAGVFSQNIDTANTLARALRVGTVW
INCFDTFDATIPFGGYKMSGQGREKGEYGLRNYLQVKAVVTPLKNPAWL
>StALDH2B7
MGKGTGSFGKRRNKTHTLCVRCGRRSFHIQKSRCSACAYPAARLRKYNWSVKALRRKTTG
TGRMRYLRNVPRRFKTNFREGRNSRVAATAALRYTTAAPIAQDPIKPSVNVEYTKLFING
QFVDSTSGKTFPTLDPRTGEVIAHVAEGDVEDINRAVVAARNAFDEGPWPKMSAYERSKV
LFRIADLIEKHNDEIATLETWDSGKLYQQVATIEIPMIVRILRYYAGWADKIHGMTVPAD
GPYHVQTLHEPIGVVGQIIPWNFPLLMFAWKIGPALACGNTVVLKTAEQTPLSALYVSKL
LQEAGLPEGVVNVISGFGPTAGAALCSHMDVDKLAFTGSTDTGKTIMSLAANSNLKPVTL
ELGGKSPFIVCEDADVDQAVEFAHFALFFNQGQCCCAGSRTYVHESIYDEFVEKAKARAL
KRTVGDPFESGNEQGPQISSEQFEKVLKYIRSGIESGATLETGGDRLGTRGYYIKPTVFS
NVKDDMLIATDEIFGPVQSILKFKDHDEVIRRANATKYGLAAGVFTKNIDTANTFMRALR

VGTIWINCFDIFDAAIPFGGYKMSGQGREKGEYSLKQYLQVKAVVTSLKNPAWL
>StALDH2C1
MAESNGNSETQFQIPKIKFTKLFINGEFVDSVSGNTFETIDPRNEEVIARISEGDKEDVD
LAVKAAREAFDDGPWPRLSPSERRRIMLKFADLIVENAEEIAALDAMDAGKLFAPVKNMD
IPAAAEVIRYYAGAADKIHGTTLKMSCELQGYTLLEPIGVVGHIIPWNFPTQMFVMKVGP
ALAAGCTMIVKPAEQTPLSALYYAQLAKQAGVPDGVINVVTGFGSTAGAALCSHMDVDKI
SFTGSTEVGRLVMQAAALSNLKPVSLELGGKSPFIVFDDVDVDKVAPLALVGILYNKGEI
CVAGSRLFIQEGIYDKFVKKLEEMAKTWVVGDPFDPNSHQGPQVDKKQYERVLSYIEHGK
REGAKLLTGGNALDRKGYFIEPTIFIDVEDDMTIAKEEIFGPVLAVMKFKTVEEVIKRAN
CTNYGLAAGVMTNDLNIANTVSRSIRAGVIWINCYFAFDPDCPYGGYKCSGFERDLGMEG
LHKYLQVKSVATPIYNSPWL
>StALDH3F2
MNGVEEDVLGELRTTFRSGRTRSVAWRKAQLQAILKLLDENEEEIFEALKQDLGKHPVES
YRDEVGVVRKSATNALRCVEKWMAPQKAPIPLVLFPARGAVVSEPLGVVLLFVSWNFPIS
LTLDPAIGAISAGNTIVLKPSELAPKCSSVLANTIPRYLDPEAIKVVEGGQDVSEQLLQL
KWDKIFFTGSPRVGRLIMSAAAKHLTPVTLELGGKCPTILDTLSSSYDLQVAVKRIAGGK
WGPCNGQACIGIDYVLVETQFAPVLIELLEKIIKTFYGENLKTLGNLARIVNKHHFDRVH
NLLKDPKVAASVVYGGSVDEENMVIEPTILLNPPLDADIMTEEIFGPLLPIITLNNIEES
IQFINSRPKPLAIYAFTKNDSLKEKILQETSSGSLTFNDAMIQFICDTLPFGGVGQSGYG
RYHGKFSFDTFSHEKAVLHRSLLIELESRYPPWNNFKLEFVRLAYDYDYLGLILLLLGLR
GIFRTNRRQ
>StALDH3H1
MDAEAIVKELRGTYGTGKTKSYEWRVSQLKALFKIAENHEKEITDALYSDLSKPELEAFI
HEISMMKTACKLALKELKRWMKPEKVKTSLTSFPSSAEIVPEPLGVVLVISAWNYPFLLS
LDPVIGAIAAGNAVVLKPSEIAPATSSVLAKLLGQYMDVSAIRVVEGAVPETTALLEQKW
DKIFYTGNGKVGRIVLAAAAKHLTPVVLELGGKSPVVVDSNIDYKIAVRRIIAGKWGCNN
GQACISPDYIITTKENVPKLLDAMKQELEKFYGKDPLKSGDLSRIVNANHFQRLSKLLDD
KKVVDKVVHGGQRDEDNLKISPTILLDVPEDSLIMKEEIFGPLLPIITVNKVEDSIQFIN
AREKPLAAYLFTSNKKLEEEFVMNISAGGLLINDTTLQVALSTLPFGGVGESGMGSCHGK
FSFDSFSHKKAVLRRSFAGDVPARYPPYTTGKARFLKALLNGDILGLIRALIGW
>StALDH5F1
MALSACAMLHRSSISGPVRLMTTATQSIAAKLSSSGLLRSQALIGGKWVDAYDGKTIKVH
NPATGEVITDVPCMGGRETNDAISSAYDAFSSWSKLTAAERSKYLRKWYDLIMAHKEELG
QLMTLEQGKPLKEAIGEVSYGAGFIEFSAEEGKRIYGDIIPSPLADRRLFVLKQPVGVVG
AITPWNFPLAMITRKVGPALACGCTVVIKPSELTPLTALAAAELSIQAGIPPGVVNVVMG
NAPAIGDALLASPQVRKITFTGSTKVGKKLMEGAAATVKKVSLELGGNAPCIIFDDADLE
VALKGALATKFRNTGQTCVCANRILVQEGIYDKFANAFAKAVQNMKVGDGFTEGVEQGPL
INEAAVQKVESFVEEATSKGAKVLVGGKRHSLGMTFYEPTVVTGVNSEMLLAKEEVFGPV
APLLKFKTDEEAIQMANDTNAGLAAYIFSTNIKRAWRVTEALEYGIVGVNEGLVSTEVAP
FGGVKQSGLGREGSKYGMDEYLEMKYVCLGSMS
>StALDH6B1
MMQFSVHRVRKVRSLTPGIFALANHHFSVATESSWKHRTSLRVPNLIGGSFVDSQSSEFV
DVINPATQEVVSQIPLTTDKEFKSAVSAAKEAFPSWKNTPITTRQRVMLKFQELIRKNMD
KLAFNVTTEQGKTLKDAQGDVFRGLEVVEHACGMATLQMGEYVSNVSNGIDTYSLREPLG
VCAGICPFNFPAMIPLWMFPVAATCGNTFILKPSEKDPGASMMLAELAMEAGLPDGVLNI
VHGTHDVVNAICDDDDIRAISFVGSNTAGMHIYSRASAKGKRVQSNMGAKNHGVVMPDAN
IDSTINALVAAGFGAAGQRCMALSTVVFVGDSKPWEEKLLERAKTLKVSAGTEPDADLGP
VISKQAKERVCQLVQSGVDSGAKLLLDGRDIVVPGYEKGNFVGPTILSGVTPDMECYKEE

IFGPVLLCMQANSLDEAINIVNQNKYGNGAAIFTTSGVAARRFQTEIESGQIGINVPIPV
PLPFFSFTGSKASFAGDLNFYGKAGVQFYTQIKTVTQQWKDLSGGSGVSLAMPTSQK
>StALDH7A1
MTSFTKKEYEFLKELGIGPQNLGCYVNGTWKATGPVISTFNPANNQIIAEVVEASAQDYE
EGMSACAEAAKIWVQVPAPKRGEIVRQIGDALRANLQEFGRLVSLEMGKILPEGIGEVQE
VIDMCDFAVGLSRQLNGSVIPSERPNHMMLEMWNPLGIVGVITAFNFPCAVLGWNACIAL
VCGNCVVWKGAPTTPLVTIAMTKIVASVLEKNNLPGSIFTAFCGGADVGQAIAKDTRIPL
VSFTGSSKVGLAVQQTVSQRFGKCLLELSGNNAIIIMDDADIKLAVRSVLFAAVGTAGQR
CTTCRRLLVHESIYEKVLEPLVDVYKQVKIGDPLEKGTLLGPLHTCTSRENFEKGIHNIK
SQGGKILTGGSVVESEGNFVHPTIVEISSKAEIVKEELFAPVLYVMKFKTFEEAVEINNS
VPQGLSSSIFTRNPENIFKWIGPQGSDCGIVNVNIPTNGAEIGGAFGGEKGTGGGREAGS
DSWKQYMRRSTCTINYGSELPLAQGINFG
>StALDH10A1
MAIPNIRIPCRQLFIDGEWREPLKKNRLPIINPANEEIIGYIPAATEEDVDIAVKAARSA
LRRDDWGSTTGAQRAKYLRAIAAKVLEKKPELATLETIDNGKPWFEAASDIDDVVACFEY
YADLAEALDSKKKTEVKLHLDSFKTHVLREPLGVVGLITPWNYPLLMTTWKVAPALAAGC
AAILKPSELASITSLELGEICREVGLPPGALSILTGLGHEAGSPLVSHPDVDKIAFTGSG
PTGVKIMTAAAQLVKPVTLELGGKSPIVVFDDIHDLDIAVEWTLFGCFWTNGQICSATSR
LIIQETIAPQFLARLLEWTKNIKISDPLEEDCKLGPVISRGQYEKVLKFISTAKDEGATI
LYGGDRPEHLKKGYYIQPTIITDVDTSMEIWNEEVFGPVLCVKTFKTEEEAIELANDTKY
GLGAAILSKDLERCERFTKAFQSGVVWINCSQPCFWQPPWGGKKRSGFGRELGEWSLENY
LNIKQVTQYVTPDEPWAFYKSPSKL
>StALDH10A2
MANRNVPISRRQLYIGGEWREPVKKNRIPIINPATEEIIGDIPAATAEDVDIAVEAARKA
IARDDWGSTTGAQRAKYLRAIAAKVLEKKSVLATLESLDSGKTLFESAADMDDVAGCFEY
YADLAEALDSRRKTPVNLNSDSFKTYVLREPLGVVGLITPWNYPLLMAIWKVAPALAAGC
AAILKPSELASVTCLELGEICREIGLPSGALNILTGLGPEAGGPLASHPHVDKISFTGSG
PTGSKIMTAAAQLVKPVSLELGGKSPIVVFDDIDNLDIAAEWTLFGIFANTGQVCSATSR
LIVQESIASAFLDRLLKWTKNIKISDPLEEDCKLGPVVSAGQYEKVLKFISNAKSEGATI
LYGGKRPQHLKKGYYVQPTIITDVNTSMEIWKEEVFGPVLCVKTFKTEEEAIELANDTKY
GLAAAVMSKDVKRCERFTKAFQTGIIWINCSQPTFNQLPWGGKKRSGFGRDLGEWGLESF
LNIKQVTEYTSAEPWAFYKSPSRN
>StALDH11A1
MAGNGVFAEIIDGEVYKYYCEGEWRKSASGKSVAIINPTTRKTQYKVQACTQEEVNKVME
IAKAAQKSWAKTPLWKRAELLHKAAAILKEHKAPIAECLVKEIAKPAKDAVTEVVRSGDL
VSYTAEEGVRILGEGKFLVSDSFPGNERTKYCLTSKIPLGVILAIPPFNYPVNLAVSKIA
PALIAGNSLVLKPPTQGAVAALHMVHCFHLAGFPKGLISCVTGKGSEIGDFLTMHPGVNC
ISFTGGDTGVAISKKAGMVPLQMELGGKDACIVLEDADLDLAAGNIVKGGFSYSGQRCTA
VKVVLVMESVADTLVEKVNAKVAKLTVGPPEDNCDITPVVSESSANFIEGLVMDAKEKDA
TFCQPYKREGNLIWPLLLDNVRPDMRIAWEEPFGPVLPVIRINSVEEGIHHCNASNFGLQ
GCVFTKDINKAILISDAMETGTVQINSAPARGPDHFPFQGIKDSGIGSQGITNSINMMTK
VKTTVINLPTPSYTMG
>StALDH12A1
MYRLSAYRQLKNRASSSHLNWITLFNSTRSNHTLSFATVKAEEVSGSQPAEVHNLVQGKW
TKSSSWNTILDPLNGQPFIKVAEVNESELQPFVESLSKCPKHGLHNPFKAPERYLMLGDV
STKAAHALGLPEVSDFFAKLIQRVSPKSYQQALIEVLVTQKFLENFCGDQVRFLARSFAV
PGNHLGQQSHGFRWPYGPVAVIAPFNFPLEIPLLQLMGALYMGNKPVLKVDSKVCIVMEQ
MLRLLHECGLPVDDVDFINSDGKTMNKLLVEAKPRMTLFTGSSRVAEKLADDLSGRVKLE

DAGFDWKILGPDVNEVDYVAWVCDQDAYACSGQKCSAESILFMHENWSKSSLIDKMTELA
ARRKLDDLTIGPVLTVTTETMLDHAKKLLQIPGSRLLFGGEALQNHSIPKIYGAIKPTAI
FVPLEEILKDEHYPLVTKEIFGPFQVVTEYKDNQLPLVLDALEKMHAHLTAAVVSNDILF
LQKVIGNSVNGTTYAGLRARTTGAPQNHWFGPAGDPRGAGIGTPEAIKLVWSCHREIIYD
VGPMPLGWKVPAST
>StALDH18A1
MDSADPARAFVKDVKRIIIKVGTAVVTRGDGRLALGRMGSLCEQIRELTSQGFEVILVTS
GAVGVGRQRLRYRKLINSSFADLQKPQGDLDGKACAAVGQNGLMALYDTLFSQLDVTSAQ
LMVTDNDFRDPDFRRQLNETVNSLLCLKVVPIFNENDAISTRKAPYEDSSGIFWDNDSLA
ALLAMELKADLLVLLSDVEGLYTGPPSDPQSELIHTYVKEKHEGLITFGDKSRVGRGGMT
AKVKAAVYAAYAGIPVVITSGFANNNIIKALDGQRVGTLFHREAIKWASIGDFDAREMAV
SARECARRLQTLSSQERSKILLDIADALEAKEEEILAENEADVAAAQQSGYENSLISRLA
MKPGKISSLANSVRVLANMDEPVGRILKRTELADGIILEKTSSPLGVLLIIFESRPDALV
QIASLAVRSGNGLLLKGGKEAKRSNAILHKVITSSIPPTVGERLIGLVTSREEIPELLKL
DDVIDLVIPRGSNKLVSQIKAATKIPVLGHADGICHVFIDKSADLDMAKRIVLDAKTDYP
AACNAMETLLVHEDLVQTGGLNDLILELQVKGVSLFGGPKASSVLSIPEANSFHHEYGAL
ACTVEIVEDVNTAIEHIHRHGSAHTDSIITEDKEVAELFLRQVDSAAVLHNASTRFSDGF
RFGLGAEVGISTSRIHARGPVGVEGLLTTRWLARGSGQVVDGDKEIVYTHRDLNLEA
>StALDH18A2
MDSADPARAFVKDVKRIIIKVGTAVVTRGDGRLALGRMGSLCEQIRELTSQGFEVILVTS
GAVGVGRQRLRYRKLINSSFADLQKPQGDLDGKACAAVGQNGLMALYDTLFSQLDVTSAQ
LMVTDNDFRDPDFRRQLNETVNSLLCLKVVPIFNENDAISTRKAPYEDSSGIFWDNDSLA
ALLAMELKADLLVLLSDVEGLYTGPPSDPQSELIHTYVKEKHEGLITFGDKSRVGRGGMT
AKVKAAVYAAYAGIPVVITSGFANNNIIKALDGQRVGTLFHREAIKWASIGDFDAREMAV
SARECARRLQTLSSQERSKILLDIADALEAKEEEILAENEADVAAAQQSGYENSLISRLA
MKPGKISSLANSVRVLANMDEPVGRILKRTELADGIILEKTSSPLGVLLIIFESRPDALV
QIASLAVRSGNGLLLKGGKEAKRSNAILHKVITSSIPPTVGERLIGLVTSREEIPELLKL
DDVIDLVIPRGSNKLVSQIKAATKIPVLGHADGICHVFIDKSADLDMAKRIVLDAKTDYP
AACNAMETLLVHEDLVQTGGLNDLILELQVKGVSLFGGPKASSVLSIPEANSFHHEYGAL
ACTVEIVEDVNTAIEHIHRHGRQVTFLITIQF
>StALDH22A1
MAFWWPLIVIAIAFAICKLLLMLIPDNVPSIDVDTSDVLDDGNQAKDNSFIYIPSRRHTD
KVQCYEPATMKYLGYFPALKPDEVKERVVQARKAQKIWAKSSFKQRRLFLRILLKYIIEH
QDLICNISSRDTGKTMVDASLGEIMTTCEKIHWLLSEGEKWLKPEYRSCGRSMLHKVAKV
EFSPFGVVGAIVSWNYPFHNIFNPMLAAVFSGNSIVIKVSEHASWSGCFYLRIIQTALAA
VGAPENLVEVITGFAETGEALVSSVDKIIFVGSPGVGKKIMRSASNTLIPVTLELGGKDA
FIVCEDVDVPHVAQIAARGALQSSGQNCAGAERFYVHKDVYSSFVAEIVKIVKSVTAGPP
LSGKYDMGAICMQEHSERLQYLVNDALDKGAEIVARGSVGNIGEGAVDQYFPPTVIVNVN
HTMKLMQEEAFGPILPIMKFSSDEEVVQLANDSSYGLGCAVFSGSQRRARHIASQLHCGV
AAINDFASNYMCQSLPFGGVKDSGFGRFAGIEGLRACCLVKSVVEDRWWPFIKTKIPKPI
QYPIAENGFEFQESLVHTLYGLNIWDRLRALVNVLKILSQQPPAPTSNRRRND
>AtALDH2C4
MENGKCNGATTVKLPEIKFTKLFINGQFIDAASGKTFETIDPRNGEVIATIAEGDKEDVDLAVNAARYAF
DHGPWPRMTGFERAKLINKFADLIEENIEELAKLDAVDGGKLFQLGKYADIPATAGHFRYNAGAADKIHG
ETLKMTRQSLFGYTLKEPIGVVGNIIPWNFPSIMFATKVAPAMAAGCTMVVKPAEQTSLSALFYAHLSKE
AGIPDGVLNIVTGFGSTAGAAIASHMDVDKVSFTGSTDVGRKIMQAAAASNLKKVSLELGGKSPLLIFND
ADIDKAADLALLGCFYNKGEICVASSRVFVQEGIYDKVVEKLVEKAKDWTVGDPFDSTARQGPQVDKRQF
EKILSYIEHGKNEGATLLTGGKAIGDKGYFIQPTIFADVTEDMKIYQDEIFGPVMSLMKFKTVEEGIKCA

NNTKYGLAAGILSQDIDLINTVSRSIKAGIIWVNCYFGFDLDCPYGGYKMSGNCRESGMDALDNYLQTKS
VVMPLHNSPWM
>AtALDH2B4
MAARRVSSLLSRSFSASSPLLFRSQGRNCYNGGILRRFGTSSAAAEEIINPSVQVSHTQLLINGNFVDSA
SGKTFPTLDPRTGEVIAHVAEGDAEDINRAVKAARTAFDEGPWPKMSAYERSRVLLRFADLVEKHSEELA
SLETWDNGKPYQQSLTAEIPMFARLFRYYAGWADKIHGLTIPADGNYQVHTLHEPIGVAGQIIPWNFPLL
MFAWKVGPALACGNTIVLKTAEQTPLTAFYAGKLFLEAGLPPGVLNIVSGFGATAGAALASHMDVDKLAF
TGSTDTGKVILGLAANSNLKPVTLELGGKSPFIVFEDADIDKAVELAHFALFFNQGQCCCAGSRTFVHEK
VYDEFVEKSKARALKRVVGDPFRKGIEQGPQIDLKQFEKVMKYIKSGIESNATLECGGDQIGDKGYFIQP
TVFSNVKDDMLIAQDEIFGPVQSILKFSDVDEVIKRANETKYGLAAGVFTKNLDTANRVSRALKAGTVWV
NCFDVFDAAIPFGGYKMSGNGREKGIYSLNNYLQIKAVVTALNKPAWI
>AtALDH2B7
MASRRVSSLLSRSFMSSSRSIFSLRGMNRGAQRYSNLAAAVENTITPPVKVEHTQLLIGGRFVDAVSGKT
FPTLDPRNGEVIAQVSEGDAEDVNRAVAAARKAFDEGPWPKMTAYERSKILFRFADLIEKHNDEIAALET
WDNGKPYEQSAQIEVPMLARVFRYYAGWADKIHGMTMPGDGPHHVQTLHEPIGVAGQIIPWNFPLLMLSW
KLGPALACGNTVVLKTAEQTPLSALLVGKLLHEAGLPDGVVNIVSGFGATAGAAIASHMDVDKVAFTGST
DVGKIILELASKSNLKAVTLELEESHHSFVCEDADVDQAVELAHFALFFNQGQCCCAGSRTFVHERVYDE
FVEKAKARALKRNVGDPFKSGIEQGPQVDSEQFNKILKYIKHGVEAGATLQAGGDRLGSKGYYIQPTVFS
DVKDDMLIATDEIFGPVQTILKFKDLDEVIARANNSRYGLAAGVFTQNLDTAHRLMRALRVGTVWINCFD
VLDASIPFGGYKMSGIGREKGIYSLNNYLQVKAVVTSLKNPAWL
>AtALDH3I1
MTKLLEINHIQTLCFAKGFSPARLNVATSPFLISRRGGGGYCSNACIPYRLKFTCYATLSAVVKEQASDF
RGKEAALLVDELRSNFNSGRTKSYEWRISQLQNIARMIDEKEKCITEALYQDLSKPELEAFLAEISNTKS
SCMLAIKELKNWMAPETVKTSVTTFPSSAQIVSEPLGVVLVISAWNFPFLLSVEPVIGAIAAGNAVVLKP
SEIAPAASSLLAKLFSEYLDNTTIRVIEGGVPETTALLDQKWDKIFFTGGARVARIIMAAAARNLTPVVL
ELGGKCPALVDSDVNLQVAARRIIAGKWACNSGQACIGVDYVITTKDFASKLIDALKTELETFFGQNALE
SKDLSRIVNSFHFKRLESMLKENGVANKIVHGGRITEDKLKISPTILLDVPEASSMMQEEIFGPLLPIIT
VQKIEDGFQVIRSKSKPLAAYLFTNNKELEKQFVQDVSAGGITINDTVLHVTVKDLPFGGVGESGIGAYH
GKFSYETFSHKKGVLYRSFSGDADLRYPPYTPKKKMVLKALLSSNMFAAILAFFGFSKDS
>AtALDH3H1
MAAKKVFGSAEASNLVTELRRSFDDGVTRGYEWRVTQLKKLMIICDNHEPEIVAALRDDLGKPELESSVY
EVSLLRNSIKLALKQLKNWMAPEKAKTSLTTFPASAEIVSEPLGVVLVISAWNYPFLLSIDPVIGAISAG
NAVVLKPSELAPASSALLTKLLEQYLDPSAVRVVEGAVTETSALLEQKWDKIFYTGSSKIGRVIMAAAAK
HLTPVVLELGGKSPVVVDSDTDLKVTVRRIIVGKWGCNNGQACVSPDYILTTKEYAPKLIDAMKLELEKF
YGKNPIESKDMSRIVNSNHFDRLSKLLDEKEVSDKIVYGGEKDRENLKIAPTILLDVPLDSLIMSEEIFG
PLLPILTLNNLEESFDVIRSRPKPLAAYLFTHNKKLKERFAATVSAGGIVVNDIAVHLALHTLPFGGVGE
SGMGAYHGKFSFDAFSHKKAVLYRSLFGDSAVRYPPYSRGKLRLLKALVDSNIFDLFKVLLGLA
>AtALDH3F1
MEAMKETVEESLREMRETFASGRTRSLKWRKAQIGAIYEMVKDNEDKICNALFQDLGKLSTEAFRDELGV
VLRTATVAINCLDKWAVPKHSKLPLLFYPAKGKVISEPYGTVLVLSSWNFPISLSLDPLIGAIAAGNTVL
LKSSELSPNASAFLAKTIPAYLDTKAIKVIEGGPDVATILLQHQWDKIFFTGSPKIGRIIMAAAAQHLTP
VTLELGGKCPTIVDHTISKNIKSVVKRIAGGKWGSCNGQACISVDYVLIEKSFAPTLIDMLKPTIKSFFG
ENPKESGCLSRIANKHHVQRLSRLLSDPRVQASIVYGGSIDEDKLYVEPTILLDPPLDSEIMNEEIFGPI
LPIITVRDIQESIGIINTKPKPLAIYAFTNDENLKTRILSETSSGSVTFNDVMIQYMCDALPFGGVGESG
IGRYHGKYSFDCFSHEKAIMEGSLGMDLEARYPPWNNFKLTFIRLAFREAYFKLILLMLGLKR
>AtALDH5F1
MVIGAAARVAIGGCRKLISSHTSLLLVSSQCRQMSMDAQSVSEKLRSSGLLRTQGLIGGKWLDSYDNKTI
KVNNPATGEIIADVACMGTKETNDAIASSYEAFTSWSRLTAGERSKVLRRWYDLLIAHKEELGQLITLEQ

GKPLKEAIGEVAYGASFIEYYAEEAKRVYGDIIPPNLSDRRLLVLKQPVGVVGAITPWNFPLAMITRKVG
PALASGCTVVVKPSELTPLTALAAAELALQAGVPPGALNVVMGNAPEIGDALLTSPQVRKITFTGSTAVG
KKLMAAAAPTVKKVSLELGGNAPSIVFDDADLDVAVKGTLAAKFRNSGQTCVCANRVLVQDGIYDKFAEA
FSEAVQKLEVGDGFRDGTTQGPLINDAAVQKVETFVQDAVSKGAKIIIGGKRHSLGMTFYEPTVIRDVSD
NMIMSKEEIFGPVAPLIRFKTEEDAIRIANDTIAGLAAYIFTNSVQRSWRVFEALEYGLVGVNEGLISTE
VAPFGGVKQSGLGREGSKYGMDEYLEIKYVCLGDMNRH
>AtALDH6B2
MVRVKQKNLESYRSNGTYPPTWRNPTTSFAPDQHRVSIHSSLKSKTKRRRLYKEADDNTKLRSSSSTTTT
TTTMLLRISGNNLRPLRPQFLALRSSWLSTSPEQSTQPQMPPRVPNLIGGSFVESQSSSFIDVINPATQE
VVSKVPLTTNEEFKAAVSAAKQAFPLWRNTPITTRQRVMLKFQELIRKNMDKLAMNITTEQGKTLKDSHG
DIFRGLEVVEHACGMATLQMGEYLPNVSNGVDTYSIREPLGVCAGICPFNFPAMIPLWMFPVAVTCGNTF
ILKPSEKDPGASVILAELAMEAGLPDGVLNIVHGTNDTVNAICDDEDIRAVSFVGSNTAGMHIYARAAAK
GKRIQSNMGAKNHGLVLPDANIDATLNALLAAGFGAAGQRCMALSTVVFVGDAKSWEDKLVERAKALKVT
CGSEPDADLGPVISKQAKERICRLIQSGVDDGAKLLLDGRDIVVPGYEKGNFIGPTILSGVTPDMECYKE
EIFGPVLVCMQANSFDEAISIINKNKYGNGAAIFTSSGAAARKFQMDIEAGQIGINVPIPVPLPFFSFTG
NKASFAGDLNFYGKAGVDFFTQIKTVTQQWKDIPTSVSLAMPTSQKQ
>AtALDH7B4
MGSANNEYEFLSEIGLTSHNLGSYVAGKWQANGPLVSTLNPANNQPIAQVVEASLEDYEQGLKACEEAAK
IWMQVTAPKRGDIVRQIGDALRSKLDYLGRLLSLEMGKILAEGIGEVQEVIDMCDFAVGLSRQLNGSVIP
SERPNHMMLEMWNPLGIVGVITAFNFPCAVLGWNACIALVCGNCVVWKGAPTTPLITIAMTKLVAEVLEK
NNLPGAIFTAMCGGAEIGEAIAKDTRIPLVSFTGSSRVGSMVQQTVNARSGKTLLELSGNNAIIVMDDAD
IQLAARSVLFAAVGTAGQRCTTCRRLLLHESVYDKVLEQLLTSYKQVKIGNPLEKGTLLGPLHTPESKKN
FEKGIEVIKSQGGKILTGGKAVEGEGNFVEPTIIEISADAAVVKEELFAPVLYVLKFKSFGEAVAINNSV
PQGLSSSIFTRNPENIFRWIGPLGSDCGIVNVNIPTNGAEIGGAFGGEKATGGGREAGSDSWKQYMRRST
CTINYGNELPLAQGINFG
>AtALDH10A8
MAIPMPTRQLFIDGEWREPILKKRIPIVNPATEEVIGDIPAATTEDVDVAVNAARRALSRNKGKDWAKAP
GAVRAKYLRAIAAKVNERKTDLAKLEALDCGKPLDEAVWDMDDVAGCFEFYADLAEGLDAKQKAPVSLPM
ESFKSYVLKQPLGVVGLITPWNYPLLMAVWKVAPSLAAGCTAILKPSELASVTCLELADICREVGLPPGV
LNVLTGFGSEAGAPLASHPGVDKIAFTGSFATGSKVMTAAAQLVKPVSMELGGKSPLIVFDDVDLDKAAE
WALFGCFWTNGQICSATSRLLVHESIASEFIEKLVKWSKNIKISDPMEEGCRLGPVVSKGQYEKILKFIS
TAKSEGATILHGGSRPEHLEKGFFIEPTIITDVTTSMQIWREEVFGPVLCVKTFASEDEAIELANDSHYG
LGAAVISNDTERCDRISEAFEAGIVWINCSQPCFTQAPWGGVKRSGFGRELGEWGLDNYLSVKQVTLYTS
NDPWGWYKSPN
>AtALDH10A9
MAITVPRRQLFIGGQWTEPVLRKTLPVVNPATEDIIGYIPAATSEDVELAVEAARKAFTRNNGKDWARAT
GAVRAKYLRAIAAKVIERKSELANLEAIDCGKPLDEAAWDMDDVAGCFEYYADLAEGLDAKQKTPLSLPM
DTFKGYILKEPIGVVGMITPWNYPLLMAVWKVAPSLAAGCTAILKPSELASLTCLELADICREVGLPPGV
LNILTGLGTEAGAPLASHPHVDKIVFTGSTTTGSSIMTSAAKLVKPVSLELGGKSPIIVFDDVDIDKAVE
WTMFGCFWTNGQICSATSRLLVHERIADEFLDKLVKWTKNIKISDPFEEGCRLGPVVSKGQYERVLKFVS
NARNEGATVLCGGVRPEHLKKGYFVEPAIVSNVTTSMEIWREEVFGPALCVKTFSTEDEAIQLANDSQYG
LAGAVLSNDLERCDRVSKAFQAGIVWVNCSQPCFCQAPWGGTKRSGFGRELGEWGLENYLSVKQVTQYIS
DEPWGWYKPPSKL
>AtALDH11A3
MAGTGLFAEILDGEVYKYYADGEWKTSSSGKSVAIMNPATRKTQYKVQACTQEEVNAVMELAKSAQKSWA
KTPLWKRAELLHKAAAILKDNKAPMAESLVKEIAKPAKDSVTEVVRSGDLISYCAEEGVRILGEGKFLLS
DSFPGNDRTKYCLTSKIPLGVVLAIPPFNYPVNLAVSKIAPALIAGNSLVLKPPTQGAVSCLHMVHCFHL
AGFPKGLISCITGKGSEIGDFLTMHPAVNCISFTGGDTGISISKKAGMIPLQMELGGKDACIVLDDADLD

LVASNIIKGGFSYSGQRCTAVKVVLVMESVADELVEKVKAKVAKLTVGPPEENSDITAVVSESSANFIEG
LVMDAKEKGATFCQEYKREGNLIWPLLLDNVRPDMRIAWEEPFGPVVPVLRINSVEEGINHCNASNFGLQ
GCVFTKDINKAILISDAMETGTVQINSAPARGPDHFPFQGLKDSGIGSQGVTNSINLMTKVKTTVINLPT
PSYSMG
>AtALDH12A1
MYRVFASRALRAKSLCDKSSTSLASLTLSRLNHSIPFATVDAEELSGSHPAEVQSFVQGKWIGSSNHNTL
LDPLNGEPFIKVAEVDESGTQPFVDSLSQCPKHGLHNPFKSPERYLLYGDISTKAAHMLALPKVADFFAR
LIQRVAPKSYQQAAGEVFVTRKFLENFCGDQVRFLARSFAIPGNHLGQQSHGYRWPYGPVTIVTPFNFPL
EIPLLQLMGALYMGNKPLLKVDSKVSIVMEQMMRLLHYCGLPAEDVDFINSDGKTMNKILLEANPRMTLF
TGSSRVAEKLALDLKGRIRLEDAGFDWKVLGPDVQEVDYVAWQCDQDAYACSGQKCSAQSMLFVHENWSK
TPLVSKLKELAERRKLEDLTIGPVLTFTTEAMLEHMENLLQIPGSKLLFGGKELKNHSIPSIYGALEPTA
VYVPIEEILKDNKTYELVTKEIFGPFQIVTEYKKDQLPLVLDALERMHAHLTAAVVSNDPIFLQEVIGNS
VNGTTYAGLRGRTTGAPQNHWFGPAGDPRGAGIGTPEAIKLVWSCHREVIYDYGPVPQGWELPPST
>AtALDH18B1
MEELDRSRAFARDVKRIVVKVGTAVVTGKGGRLALGRLGALCEQLAELNSDGFEVILVSSGAVGLGRQRL
RYRQLVNSSFADLQKPQTELDGKACAGVGQSSLMAYYETMFDQLDVTAAQLLVNDSSFRDKDFRKQLNET
VKSMLDLRVIPIFNENDAISTRRAPYQDSSGIFWDNDSLAALLALELKADLLILLSDVEGLYTGPPSDPN
SKLIHTFVKEKHQDEITFGDKSRLGRGGMTAKVKAAVNAAYAGIPVIITSGYSAENIDKVLRGLRVGTLF
HQDARLWAPITDSNARDMAVAARESSRKLQALSSEDRKKILLDIADALEANVTTIKAENELDVASAQEAG
LEESMVARLVMTPGKISSLAASVRKLADMEDPIGRVLKKTEVADGLVLEKTSSPLGVLLIVFESRPDALV
QIASLAIRSGNGLLLKGGKEARRSNAILHKVITDAIPETVGGKLIGLVTSREEIPDLLKLDDVIDLVIPR
GSNKLVTQIKNTTKIPVLGHADGICHVYVDKACDTDMAKRIVSDAKLDYPAACNAMETLLVHKDLEQNAV
LNELIFALQSNGVTLYGGPRASKILNIPEARSFNHEYCAKACTVEVVEDVYGAIDHIHRHGSAHTDCIVT
EDHEVAELFLRQVDSAAVFHNASTRFSDGFRFGLGAEVGVSTGRIHARGPVGVEGLLTTRWIMRGKGQVV
DGDNGIVYTHQDIPIQA
>AtALDH18B2
MTEIDRSRAFAKDVKRIVVKVGTAVVTGKGGRLALGRLGAICEQLAELNSDGFEVILVSSGAVGLGRQRL
RYRQLVNSSFADLQKPQMELDGKACAGVGQSSLMAYYETMFDQLDVTVAQMLVTDSSFRDKDFRKQLSET
VKAMLRMRVIPVFNENDAISTRRAPYKDSTGIFWDNDSLAALLSLELKADLLILLSDVEGLYTGPPSDST
SKLIHTFIKEKHQDEITFGEKSKLGRGGMTAKVKAAVNAAYGGVPVIITSGYAAENISKVLRGLRVGTLF
HQDAHLWAPVVDTTSRDMAVAARESSRKLQALSSEDRKQILHDIANALEVNEKTIKAENDLDVAAAQEAG
YEESLVARLVMKPGKISSLAASVRQLAEMEDPIGRVLKKTQVADDLILEKTSSPIGVLLIVFESRPDALV
QIASLAIRSGNGLLLKGGKEARRSNAILHKVITDAIPETVGGKLIGLVTSREEIPDLLKLDDVIDLVIPR
GSNKLVSQIKNSTKIPVLGHADGICHVYVDKSGKLDMAKRIVSDAKLDYPAACNAMETLLVHKDLEQNGF
LDDLIYVLQTKGVTLYGGPRASAKLNIPETKSFHHEYSSKACTVEIVEDVYGAIDHIHQHGSAHTDCIVT
EDSEVAEIFLRQVDSAAVFHNASTRFSDGFRFGLGAEVGISTSRIHARGPVGVEGLLTTRWIMRGKGQVV
DGDNGIVYTHKDLPVLQRTEAVENGI
>AtALDH22A1
MPFWWPLIVLAFAYAICKFLLMLIPPNVPSIDVDASDVLAHGKDTEENSFIYIPPRGRSQQSDKKVQCYE
PATMKYLGYFPALSPTEVEERVTLSRKAQKTWAQSSFKLRRQFLRILLKYIIEHQELICEVSSRDTGKTM
VDASLGEIMTTCEKITWLLSEGERWLKPESRSSGRAMLHKVSRVEFHPLGVIGAIVPWNYPFHNIFNPML
AAVFSGNGIVIKVSEHASWSGCFYFRIIQAALAAVGAPENLVDVITGFAETGEALVSSVDKMIFVGSTAV
GKMIMRNAAETLTPVTLELGGKDAFIICEDADVSHVAQVAVRGTLQSSGQNCAGAERFYVHKDIYTAFIG
QVTKIVKSVSAGPPLTGRYDMGAICLQEHSEHLQSLVNDALDKGAEIAVRGSFGHLGEDAVDQYFPPTVL
INVNHNMKIMKEEAFGPIMPIMQFSTDEEVIKLANDSRYALGCAVFSGSKHRAKQIASQIQCGVAAINDF
ASNYMCQSLPFGGVKDSGFGRFAGIEGLRACCLVKSVVEDRFWPLIKTKIPKPIQYPVAENAFEFQEALV
ETLYGLNIWDRLRSLIDVLKFLTDQSSNVSRTRKSH
>OsALDH2-1

MAAANGGDSKGFEVPKLEIKFTKLFINGRFVDAVSGKTFETRDPRTGEVIAKIAEGDKAD
IDLAVKAAREAFDHGPWPRMSGFARGRILHKFADLVEQHVEELAALDTVDAGKLFAMGKL
VDIPGGANLLRYYAGAADKVHGETLKMARPCHGYTLKEPVGVVGHIVPWNYPTTMFFFKA
SPALAAGCTMVVKPAEQTPLSALFYAHLAKLAGVPDGVLNVVPGFGPTAGAAISSHMDID
KVSFTGSTEVGRLVMEAAAKSNLKPVSLELGGKSPVIVFDDADLDTAVNLVHMASYTNKG
EICVAGSRIYVQEGIYDAFVKKATEMAKKSVVGDPFNPRVHQGPQIDKEQYEKILKYIDI
GKREGATLVTGGKPCGENGYYIEPTIFTDVKEEMSIAQEEIFGPVMALMKFKTVEEAIQK
ANSTRYGLAAGIVTKNIDVANTVSRSIRAGAIWINCYLGFDPDVPFGGYKMSGFGKDMGM
DALEKYLHTKAVVTPLYNTPWL
>OsALDH2-2
MGSTGDCGNGKAAAGGGGLVVPEIKFTKLFINGEFVDAASGKTFKTRDPRTGDVLAHIAE
ADKADVDLAVKAAREAFEHGKWPRMSGYERSRVMNKLADLVEQHADELAALDGADAGKLL
TLGKIIDMPAAAQMMRYYAGAADKIHGESLRVAGKYQGYTLREPIGVVGVIIPWNFPTMM
FFLKVSPALAAGCTIVVKPAEQTPLSALYYAHLAKLAGVPDGVINVVPGFGPTAGAALSS
HMDVDSVAFTGSAEIGRAIMESAARSNLKNVSLELGGKSPMIVFDDADVDMAVSLSSLAV
FFNKGEICVAGSRVYVQEGIYDEFVKKAVEAAKNWKVGDPFDAATNMGPQVDKVQFERVL
KYIEIGKNEGATLLTGGKPTGDKGYYIEPTIFVDVKEEMTIAQEEIFGPVMSLMKFKTVE
EAIEKANCTKYGLAAGIVTKNLNIANMVSRSVRAGTVWVNCYFAFDPDAPFGGYKMSGFG
RDQGMVAMDKYLQVKTVITAVPDSPWY
>OsALDH2-3
MSGSERGRVMAKYAEVVERHADELAALESLDAGKPLAAARAVDVGECVGILRYFAGAADK
IHGETLKMSRQLQGYTLREPLGVAGLIVPWNFPAIMFFSKVSPALAAGCTVVVKPAEQTP
LSALFLAHLSKQAGVPDGVINVVTGFGPTAGAAISSHMDVDVVAFTGSTEVGRLIMEASA
KSNLKPVALELGGKSPFIVFDDADLDKAVELAIGGNFFNKGEACVAGSRVFVQEGIYDRF
EQKLADTMKSWVVGDPFDPRVNQGPQVDKAQYERVLGYIEQGKAEGATVLTGGKPCGKKG
YYIEPTIFTNVKDDMVIAREEIFGPVMCLMKFKTVEEAIERANGTRYGLAAGLVTRDIDV
ANRMARSIRAGVVWVNCYFAMDRSCPFGGRKMSGFGKDDSMHALDKFLAVKSVVTPVHGS
PWF
>OsALDH2-4
MAARRAASSLLSRGLIARPSAASSTGDSAILGAGSARGFLPGSLHRFSAAPAAAATAAAT
EEPIQPPVDVKYTKLLINGNFVDAASGKTFATVDPRTGDVIARVAEGDAEDVNRAVAAAR
RAFDEGPWPRMTAYERCRVLLRFADLIEQHADEIAALETWDGGKTLEQTTGTEVPMVARY
MRYYGGWADKIHGLVVPADGPHHVQVLHEPIGVAGQIIPWNFPLLMFAWKVGPALACGNA
VVLKTAEQTPLSALFVASLLHEAGLPDGVLNVVSGFGPTAGAALSSHMGVDKLAFTGSTG
TGKIVLELAARSNLKPVTLELGGKSPFIVMDDADVDQAVELAHRALFFNQGQCCCAGSRT
FVHERVYDEFVEKARARALQRVVGDPFRTGVEQGPQIDGEQFKKILQYVKSGVDSGATLV
AGGDRAGSRGFYIQPTVFADVEDEMKIAQEEIFGPVQSILKFRFATLEFPRIPSDRDRLS
TGIYTNTLRAMCSTVEEVVRRANATPYGLAAGVFTQRLDAANTLARALRVGTVWVNTYDV
FDAAVPFGGYKMSGVGREKGVYSLRNYLQTKAVVTPIKDAAWL
>OsALDH2-5
MAAAAARRGSSLLSRCLLSRPAAAASPAVPSALRRADGTQGLLPGILQRFSTAAVAEEPI
SPPVQVNYTQLLIDGKFVDSASGKTFPTLDPRTGELIAHVAEGDAEDINRAVHAARKAFD
EGPWPKMTAYERSRILLRFADLIEKHNDEIAALETWDNGKPYAQAANIEVPMVARLMRYY
AGWADKIHGLVVPADGPHHVQVLHEPIGVAGQIIPWNFPLLMFAWKVGPALACGNTVVLK
TAEQTPLSALFASKLLHEAGLPDGVVNVVSGFGPTAGAALASHMDVDKIAFTGSTDTGKV
VLELAARSNLKSVTLELGGKSPFIIMDDADVDHAVELAHFALFFNQGQCCCAGSRTFVHE
RIYDEFVEKAKARALKRVVGDPFKNGVEQGPQIDDEQFNKILRYIKYGVDSGANLVTGGD
RLGDKGYYIQPTIFSDVQDNMRIAQEEIFGPVQSILKFNDLNEVIKRANASQYGLAAGVF

TNNLNTANTLTRALRVGTVWVNCFDVFDAAIPFGGYKQSGIGREKGIDSLKNYLQVKAVV
TPIKNAAWL
>OsALDH3-1
MEEKPQHGSLGGLVAGVREEYESGRTKELEWRKAQLGGLIRMITEEEDAIFDALHDDLGK
HRVESFRDEVGVLAKSVRNTLQNLKKWASPEKVDVPLISFPCNARVVPEPIGVVLIFSCW
NLPIGLALEPLSGAIAAGNAVVLKPSEFAPSTAAFLAANIPKYLDANAVKVVQGGAEVGE
ELMEHRWDKVLFTGNARVGRIIMTKAAKHLTPVALELGSKCPCIVDCLDSKRECQVAVNR
IIGAKWSTCAGQACVAIDYILVEEQFAPFLIELLKSTLKRFFTEPEYMARILNEKHFHRL
TNLLEDDQVKSSIVHGGNADPKTLWIEPTIVLNPPFDSDIMMEEIFGPLLPIITVKKTED
CIAFLKSKPKPLAIYAFTNNEKLKQRIVAETSSGSVLFNDAIVQYGLDSVPFGGIGESGF
GQYHGKYTFELFSHRKAVVRRSLLVEFMFRYPPWDEYKMGMLRRVFRFDYVSLVLALLAF
WLLGIRR
>OsALDH3-2
MAPAMVAAMGEKPKPAVVLGGMVSGLREVYESGRTKDLEWRQSQLKALIRLLTDKEEEIF
AVLHDDLGKHRGESFRDELGILVKSIKYTLQNLKKWAASERAESPLVAFPATAMVVPEPL
GVVLVFSCWNLPLGLALEPLSGAIAAGNAVVLKPSELAPSTAAFLAANIPRYLDSRAVKV
VLGGPNVGEELMEHRWDKVLFTGSARIGRIIMAKAVKHLTPVALELGSKCPCIVDWLDSK
RDRQIAVNRIIGAKWSTCAGQACIAIDHVIVEERFAPILIELLKSTLKRFMAKPGGMARI
LNAKHFERLSGYLEDNRVAASVVHGGYMDPKKLNIEPTLLLNPPADSDVMTEEVFGPILP
IITVKKIEDCIAYLKSKPKPIAMYAFTNNERLKRRIVEETSSGSVTFNDAVVQYALESVP
FGGVGHSGFGQYHGKYSFELFSHKKAVFKRSFLIEFMFRYPPWDERKIGTLRHVFSYNYF
LLFFNLLGFRR
>OsALDH3-3
MGRVAPSVEEVGGEQPPPALGPGETVSGTVAELRAAYESGRTRSLEWRQSQLRGLLRLLA
EEEAAAFRALREDLGKHQAEAYRDEIGVLVKSANAALREVGKWMAPEKVWVPLIAFPARA
QLEPQPLGVILVFSCWNVPLGLSLEPLVGALAAGNAVALKPSELAPATAKFLGDNVGKYM
DATAVKVIQGGPEVGEQLMEHRWDKVLFTGSPRIARVVMAAAAKHLTPVALELGGKCPCI
FDTIGGSARDLQTAVNRVVGGKWSSCAGQACLAIDYVLVEERFVPVLIKALKSTLKKFFA
DSDHMARIVNARHFQRLSDLLKDKSVAASVLHGGTLDAKNLCIEPTILLNPPLDSAIMTE
EIFGPLLPIITVKKIEDSIAFVRARPRPLAVYAFTKNAALRRRIVEETSSGSVTFNDAVV
QYGIDSLPFGGVGESGFGQYHGKYSFEMFSHKKAVLTRGYLIELTARYPPWDDSKISMMR
QLYRYNYVGFVLTFLGLKK
>OsALDH3-4
MAEEEVAAVVGELRGSFRSGRTRAAEWRAAQLRGIVRMVEEREGDISDALHSDLAKPRME
SYLHEISLAKAACTFALKGLKNWMKPEKVPAALTTFPSTAQIVSEPLGVVLVISAWNYPF
LLSIDPVIGAIAAGNAVVLKPSEIAPATSALFAKLLPEYVDSSCIKVVEGGVPETTALLE
QKWDKIFYTGSGNVGRIVMAAAAKHLTPVALELGGKCPAIVDSNTDLHVTMKRLAVGKWG
CNNGQACIAPDYVITTKSFAPELVDSLKRVLKRFYGEDPLQSEDLSRIVNSNHFRRLTNL
IEDKKVAQKIVYGGQTDEKQLKIAPTVLLDVPLDTTLMAEEIFGPLLPIVTVDKIEDSIQ
FINSRTKPLAAYLFTKDKKLQEEFVSNVPAGGMLVNDVALHLANPHLPFGGVGDSGIGSY
HGKFSFDCFTHKKAVLIRGFGGEATARYPPYTIEKQKILRGLINGSFFALILALLGFPKE
RR
>OsALDH3-5
MAAARSVGMEAEVAALRGRFAAGGTRGAEWRAAQLRGILRMAAEAEAEVCRALHADLAKP
YTESYVHEIALVKSSCKFALKNLKKWMKPQKVTAPLMTFPSTARVAAEPLGVVLVISAWN
YPFLLSIDPIIGAIAAGNAVVLKPSEVAPATSSLLAELLPRYVDGSCIKVVEGGVAETTT
LLEQKWDKIFYTGNGKVGRIVMASAAKHLTPVVLELGGKCPVVVDSNVNLHVTAKRIAAG
KWGCNNGQACISPDFIITTKSFAPKLLEALEKVLEKFYGRDPLRSSDLSRIVNSNHFNRL

KKLMDDENVSDKIVFGGQRDEHQLKIAPTIFMDVPLDSGIMKEEIFGPLLPIITVDKIHE
SFALINSMTKALAAYLFTKDSKLQEQYEAAISAGGMLVNDTAVHLTNQYLPFGGVGESGM
GAYHGRFSFEAFSHKKAVLVRRFAGEAAARYPPYSPAKLKILRGVLKGNLGAMIKAILGF
PRGK
>OsALDH5
MAMAMAMRRAAALGARHILAASSTSSSGVLLRRHMSVDAGAAMEKVRAAGLLRTQGLIGG
KWVDAYDGKTIEVQNPATGETLANVSCMGSKETSDAIASAHSTFYSWSKLTANERSKALR
KWHDLIISHKEELALLMTLEQGKPMKEALVEVTYGASFIEYFAEEAKRIYGDIIPPTLSD
RRLLVLKQPVGVVGAVTPWNFPLAMITRKVGPALACGCTVVVKPSEFTPLTALAAADLAL
QAGIPAGAINVVMGNAPEIGDALLQSTQVRKITFTGSTAVGKKLMAGSANTVKKVSLELG
GNAPCIVFDDADIDVAIKGSLAAKFRNSGQTCVCANRILVQEGIYEKFASAFIKAVQSLK
VGNGLEESTSQGPLINEAAVQKVEKFINDATSKGANIMLGGKRHSLGMSFYEPTVVGNVS
NDMLLFREEVFGPVAPLVPFKTEEDAIRMANDTNAGLAAYIFTKSIPRSWRVSEALEYGL
VGVNEGIISTEVAPFGGVKQSGLGREGSKYGMDEYLELKYICMGNLN
>OsALDH6
MLRAALLRSGSGLRRPPMAAPLSTAAAASWLSDSASSPPRVRLLIGGEFVESRADEHVDV
TNPATQEVVSRIPLTTADEFRAAVDAARTAFPGWRNTPVTTRQRIMLKYQELIRANMDKL
AENITTEQGKTLKDAWGDVFRGLEVVEHACGMGTLQMGEYVSNVSNGIDTFSIREPLGVC
AGICPFNFPAMIPLWMFPIAVTCGNTFVLKPSEKDPGAAMMLAELAMEAGLPKGVLNIVH
GTHDVVNNICDDEDIKAVSFVGSNIAGMHIYSRASAKGKRVQSNMGAKNHAIILPDADRD
ATLNALIAAGFGAAGQRCMALSTAVFVGGSEPWEDELVKRASSLVVNSGMASDADLGPVI
SKQAKERICKLIQSGADNGARVLLDGRDIVVPNFENGNFVGPTLLADVKSEMECYKEEIF
GPVLLLMKAESLDDAIQIVNRNKYGNGASIFTTSGVSARKFQTDIEAGQVGINVPIPVPL
PFFSFTGSKASFAGDLNFYGKAGVQFFTQIKTVTQQWKESPAQRVSLSMPTSQK
>OsALDH7
MGSFARKEHQFLAELGLAPRNPGSFACGAWGGSGPVVTSTNPTNNQVIAEVVEASAREYE
EGMRACYDAAKTWMAIPAPKRGEIVRQIGDALRAKLHHLGRLVSLEMGKILPEGIGEVQE
IIDMCDYAVGLSRQLNGSIIPSERPNHMMMEVWNPLGVVGVITAFNFPCAVLGWNACIAL
VCGNCVVWKGAPTTPLITIAMTKIVASVLERNNLPGSIFTAFCGGADIGQAISLDTRIPL
VSFTGSTKVGLMVQQQVNARFGKCLLELSGNNAIIVMDDADIQLAVRSVLFAAVGTAGQR
CTTCRRLLLHESIYRTFLDQLVEVYKQVRIGDPLENGTLLGPLHTPASRDAFLKGIQTIR
SQGGKILYGGSAIESEGNFVQPTIVEISPSAPVVREELFGPVLYVMKVQNLKEAVEINNS
VPQGLSSSIFTKRPDIIFKWIGPHGSDCGIVNVNIPTNGAEIGGAFGGEKATGGGREAGS
DSWKQYMRRATCTINYGSELPLAQGINFG
>OsALDH10-1
MAAPSAIPRRGLFIGGGWREPSLGRRLPVVNPATEATIGDIPAATAEDVELAVSAARDAF
GRDGGRHWSRAPGAVRAKYLKAIAAKIKDKKSYLALLETLDSGKPLDEAAGDMEDVAACF
EYYADLAEALDGKQRAPISLPMENFESYVLKEPIGVVGLITPWNYPLLMATWKVAPALAA
GCTAVLKPSELASLTCLELGGICAEIGLPPGVLNIITGLGTEAGAPLASHPHVDKIAFTG
STETGKRIMITASQMVKPVSLELGGKSPLIVFDDVDIDKAVEWAMFGCFANAGQVCSATS
RLLLHEKIAKRFLDRLVAWAKSIKISDPLEEGCRLGSVVSEGQYQKIMKFISTARCEGAT
ILYGGARPQHLKRGFFIEPTIITNVSTSMQIWREEVFGPVICVKEFRTEREAVELANDTH
YGLAGAVISNDLERCERISKAIQSGIVWINCSQPCFVQAPWGGNKRSGFGRELGQWGLDN
YLSVKQVTKYCSDEPYGWYRPPSKL
>OsALDH10-2
MATAIPQRQLFVAGEWRAPALGRRLPVVNPATESPIGEIPAGTAEDVDAAVAAAREALKR
NRGRDWARAPGAVRAKYLRAIAAKIIERKSELARLETLDCGKPLDEAAWDMDDVAGCFEY
FADLAESLDKRQNAPVSLPMENFKCYLRKEPIGVVGLITPWNYPLLMATWKVAPALAAGC

TAVLKPSELASVTCLELADVCKEVGLPSGVLNIVTGLGSEAGAPLSSHPGVDKVAFTGSY
ETGKKIMASAAPMVKPVSLELGGKSPIVVFDDVDVEKAVEWTLFGCFWTNGQICSATSRL
ILHKKIAKEFQERMVAWAKNIKVSDPLEEGCRLGPVVSEGQYEKIKQFVSTAKSQGATIL
TGGVRPKHLEKGFYIEPTIITDVDTSMQIWREEVFGPVLCVKEFSTEEEAIELANDTHYG
LAGAVLSGDRERCQRLTEEIDAGIIWVNCSQPCFCQAPWGGNKRSGFGRELGEGGIDNYL
SVKQVTEYASDEPWGWYKSPSKL
>OsALDH11
MAAVAGTGVFAEILEGEVYRYYADGEWRVSASGKSVAIVNPTTRLTQYRVQACTQEEVNK
VMETAKVAQKAWARTPLWKRAELLHKAAAILKEHKTPIAECLVKEIAKPAKDAISEVVRS
GDLVSYTAEEGVRILGEGKLLVSDSFPGNERNKYCLSSKVPLGVVLAIPPFNYPVNLAVS
KIGPALIAGNALVLKPPTQGAVAALHMVHCFHLAGFPKGLINCVTGKGSEIGDFLTMHPG
VNCISFTGGDTGIAISKKAGMVPLQMELGGKDACVVLEDADLDLVAANIVKGGFSYSGQR
CTAVKVVLIMESVADIVVEKVKAKLAKLTVGPPEADSDITPVVTESSANFIEGLVMDAKE
KGATFCQEYRREGNLIWPLLLDHVRPDMRIAWEEPFGPVLPVIRINSVEEGIHHCNASNF
GLQGCVFTKDINKAIMISDAMETGTVQINSAPARGPDHFPFQGLKDSGIGSQGITNSINM
MTKVKSTVINLPSPSYTMG
>OsALDH12
MSLILSRRRLAAAVRRSGPAALASRWMHTPPFATVSPQEISGSSPAEVQNFVQGSWTTSGNWNWLVDPLN
GEKFIKVAEVQEAEIKPFVESLSNCPKHGLHNPLKAPERYLMYGDISAKAANMLGQPVVSDFFAKLIQRV
SPKSYQQALAEVQVSQKFLENFCGDQVRFLARSFAVPGNHLGQSSNGYRWPYGPVAIITPFNFPLEIPLL
QLMGALYMGNKPVLKVDSKVSIVMDQMLRLLHACGMPAEDVDFINSDGITMNKLLLEANPKMTLFTGSSR
IAEKLAADLKGKIKLEDAGFDWKILGPDVQEVDYIAWVCDQDAYACSGQKCSAQSILFMHKNWSSSGLLD
KMKSLSERRKLEDLTIGPVLTVTTSSMIEHMKNLLKIPGSKVLFGGEPLENHSIPEIYGAFKPTAVFVPL
SEILKSGNFELVTREIFGPFQVVTEYSDDELELVLEACERMNAHLTAAVVSNDPLFLQEVLGRSVNGTTY
AGIRARTTGAPQNHWFGPAGDPRGAGIGTPEAIKLVWSCHREIIYDIGPLPKNRALPSAT
>OsALDH18-1
MGRGGIGGAGLVAAVAKADVENTDSTRGFVKDVKRIIIKVGTAVVTGPNGRLAMGRLGAL
CEQVKQLNFEGYEVILVTSGAVGVGRQRLKYRKLVNSSFADLQNPQMDMDGKACAAVGQS
VLMAIYDTLFSQLDVTSSQLLVTDRDFMDPSFGNQLRETVNSLLDLKVIPVFNENDAIST
RRQPYEDSSGIFWDNDSLARLLAQELKADLLIMLSDVEGLYSGPPSDPQSKIIHTYVHEQ
HGKLISFGEKSRVGRGGMQAKVAAAFTASSKGIPVVIASGFAIDSIIKVMRGEKIGTLFH
REANQWGCSKEATAREMAVAARDCSRHLQKLSSEERKKILLDIADALEANEDLITSENQA
DLDLAQDIGYDKSLVARMTIKPGKIKSLAGSIREIADMEDPISHTLKRTEVAKDLVFEKT
YCPLGVLLIIFESRPDALVQIASLAIRSGNGLLLKGGKEAMRSNTILHKVITGAIPDVVG
KKLIGLVKNKDEIADLLKLDDVIDLVIPRGSNKLVSQIKAATKIPVLGHADGICHVYIDK
SADMDMAKRIVLDAKVDYPAACNAMETLLVHKDLNRTEGLDDLLVELEKEGVVIYGGPVA
HDTLKLPKVDSFHHEYNSMACTLEFVDDVQSAIDHINRYGSAHTDCIITTDGKAAETFLQ
QVDSAAVFHNASTRFCDGARFGLGAEVGISTGRIHARGPVGVDGLLTTRCILRGSGQVVN
GDKGVVYTHRELPLQ
>OsALDH18-2
MASVDPSRSFVRDVKRVIIKVGTAVVSRQDGRLALGRVGALCEQVKELNSLGYEVILVTS
GAVGVGRQRLRYRKLVNSSFADLQKPQMELDGKACAAVGQSGLMALYDMLFNQLDVSSSQ
LLVTDSDFENPKFREQLTETVESLLDLKVIPIFNENDAISTRKAPYEDSSGIFWDNDSLA
GLLALELKADLLILLSDVDGLYSGPPSEPSSKIIHTYIKEKHQQEITFGDKSRVGRGGMT
AKVKAAVLASNSGTPVVITSGFENRSILKVLHGEKIGTLFHKNANLWESSKDVSTREMAV
AARDCSRHLQNLSSEERKKILLDVADALEANEDLIRSENEADVAAAQVAGYEKPLVARLT
IKPGKIASLAKSIRTLANMEDPINQILKKTEVADDLVLEKTSCPLGVLLIVFESRPDALV
QIASLAIRSGNGLLLKGGKEAIRSNTILHKVITDAIPRNVGEKLIGLVTTRDEIADLLKL

DDVIDLVIPRGSNKLVSQIKASTKIPVLGHADGICHVYIDKSADMDMAKHIVMDAKIDYP
AACNAMETLLVHKDLMKSPGLDDILVALKTEGVNIYGGPIAHKALGFPKAVSFHHEYSSM
ACTVEFVDDVQSAIDHIHRYGSAHTDCIVTTDDKVAETFLRRVDSAAVFHNASTRFSDGA
RFGLGAEVGISTGRIHARGPVGVEGLLTTRWILRGRGQVVNGDKDVVYTHKSLPLQ
>OsALDH22
MALWWPLLVLAAAYALCRILLFLIPPTVPSIDVDASDVLEDANQNKEDSYIYIPPRKGKG
AQTDKVQCYEPATMKYLGYFPALTPDEVKEHVAQARKAQKIWAKSSFKQRRQFLRILLKY
ILEHQDLICEISSRDTGKTMVDASLGEIMTTCEKITWLLDEGEKWLKPEYRSCGRSMLHK
KAKVEFYPLGVIGAIVSWNYPFHNVFNPMLAAIFSGNAAVIKVSEHASWSGCFYFRIIQA
ALAAVGAPDNLVHIITGFAETGQALVSSVDKIIFVGSPGVGRMIMNRASDTLIPVTLELG
GKDAFIVCEDVDLPSVVQVAVRAALQSSGQNCAGAERFYVHKDIYSTFVSQVVKIIKSIS
VGPPLSGRYDMGAICMIEHSEKLQNLVNDAVDKGAEIAGRGSFGHLGEDAVDQFFPPTVL
VNVNHTMKIMQEEAFGPILPIMKFNSDEEVVKLANDSKYGLGCAVFSGNQKRAIKIASQL
HCGVAAINDFASSYMCQSLPFGGVKDSGFGRFAGVEGLRACCLVKAVVEDRWWPYVKTMI
PKPIQYPVSENGFEFQELLVETLYGLSVWDRLRSLVNLLKMISEQNNSPANTRKKSR
>GmALDH2B1
MASSMRISRLLSRSFLSASTTPLFSRGGSGALGAGLSKFSTAAAIEEPIKPPVKVEHTQLLIDGKFVDAA
TGKTFPTLDPRTG
DVISHVAEGDHEDVDRAVAAARKAFDHGPWPKMTAYERQRILLRAADLFEKHNDELAALETWDNGKPYEQ
SAQIEIPMLVRLFRYYAGWADKIHGLTVPA
DGPYHVQTLHEPIGVAGQIIPWNFPLVMFAWKVGPALACGNTIVLKTAEQTPLSALYASKLLHEAGLPPG
VLNVISGFGPTAGAAIASHMDIDKLAFTGS
TETGKVVLELAARSNLKPVTLELGGKSPFIVCEDADVDEAVELAHFALFFNQGQCCCAGSRTFVHERVYD
EFIEKAKARALKRAVGDPFKGGIEQGPQID
SEQFQKILKYIRSGVESGATLETGGDRFGNSGFYIQPTVFSNVKDDMLIAKEEIFGPVQTILKFKDLDDV
IQRANNTHYGLAAGVFTKNINTANTLTRAL
RVGTVWINCFDTFDAAIPFGGYKMSGQGREKGEYSLKNYLQVKAVVTSLKNPAWL
>GmALDH2B2
MASSLRISRLLSRSFLSASTTTPLFSRGGSGALGAGLSKFSTAAAIEEPIKPPLKVEHTQLLIDGKFVDA
ATGKTFPTLDPRT
GDVISHVAEGDHEDVDRAVAAARKAFDRGPWPKMTAYERQRILLRAADLFEKHNDDLAALETWDNGKPYE
QSAQIEIPMLVRLFRYYAGWADKIHGLTVP
ADGPYHVQTLHEPIGVAGQIIPWNFPLVMFAWKVGPALACGNTIVLKTAEQTPLSALYASKLLHEAGLPP
GVLNIISGFGPTAGAAIASHMDIDKLAFTG
STETGKIVLELAARSNLKPVTLELGGKSPFIVCEDADVDEAVELAHFALFFNQGQCCCAGSRTFVHERVY
DEFIEKAKARALKRAVGDPFKGGIEQGPQI
DSEQFQKILKYIRSGVESGATLETGGDRFGNSGFYIQPTVFSNVKDDMLIAKEEIFGPVQSILKFKDLDD
VIQRANNTHYGLAAGVFTKNINTANTLTRA
LRAGTVWVNCFDTFDAAIPFGGYKMSGQGREKGEYSLKNYLQVKAVVTSLKNPAWL
>GmALDH2B3
MATRRLSLLLSRSLSSTSFQAASLLHSLGRNSGKWGNFNRFSTAAAVEDLITPQVPITYTKHLINGQFVD
AASGKTFPTYDPR
TGEVIAQVAEGDAEDINRAVSAARKAFDEGPWPKLTAYERCKIILRFADLVEKHGDELAALETWNNGKPY
EQSATAELPTFVRLFRYYAGWADKIHGLTV
PADGNYHVETLHEPIGVAGQIIPWNFPLLMFAWKVGPALACGNTVILKTAEQTPLTALYVAKLFHEAGLP
PGVLNVVSGYGPTAGAALASHMDVDKLAFT
GSTETGKVVLGLAAQSNLKPVTLELGGKSPFIVCEDADVDQAVELAHFALFFNQGQCCCAGSRTFVHEHI
YDEFLEKAKARALKRVVGDPFKKGVEQGPQ

IDVEQFQKVLRYIKSGIESKATLECGGDQIGSKGFFVQPTVFSNVQDDMLIAKDEIFGPVQTILKFKDID
EVIRRSNATHYGLAAGVFTKNVHTANTLMR
ALRVGTVWINCFDVFDAAIPFGGYKMSGIGREKGIYSLNNYLQVKAVVSPVKKPAWL
>GmALDH2B4
MASSLRISRLISRSFSSTSFFSRGGNGFLGSRQSKFSTSAAIEEEPIKPSIQVEHTQLLIDGKFVDAASG
KTFQTLDPRTGEV
IAHVAEGHSEDVDRAVSAARKAFDHGPWPKMTAYERQRILLRVADLIEKHNDELAALETWDNGKPYEQAA
KIEVPMLVRLIRYYAGWADKIHGLTVPADG
PYHVQTLHEPIGVAGQIIPWNFPLLMFAWKVGPALACGNTIVLKTAEQTPLSALYAAKLFHEAGLPAGVL
NVVSGFGPTAGAALASHMEVDKLAFTGSTD
TGKVVLELAAKSNLKPVTLELGGKSPFIVCEDADVDQAVELAHFALFFNQGQCCCAGSRTFVHENVYEEF
VQKAKARALRRVVGDPFKGGIEQGPQIDSD
QFEKILRYIRSGVESGATLETGGDKLGNKGFYIQPTVFSNVKDGMLIAKDEIFGPVQSILKFKDLGEVVQ
RANNTRYGLAAGVFTKNMDTANTLTRALRV
GTVWINCFDTFDAAIPFGGYKMSGQGREKGEYSLKNYLQVKAVVNPLKNPAWL
>GmALDH2B5
MQVMASRILSTLHYVCSSSASATKRCLGLYSHWQRSISGIAASVVADVEPSIAPVQIDQSQLLIDGKFVD
AASGKTFPTFDPR
TGDVIANVAEGDAEDVNRAVHAARKAFDEGPWPKMTAYERSRIILRFADLLEKHNDEVAAIETWDSGKTY
EQAANVEIPMVVRLFRYYAGWADKIHGLTV
PADGPYHVQTLHEPIGVAGQIVPWNFPLLIFSWKVAPALACGNTVVMKTAEQTPLSALYVSKLFLEAGLP
PGVLNVISGFGPTAGAALCSHMDVDKLAFT
GSTSTGKRVLELSAHSNLKPVTLELGGKSPFIVCKDADVDAAVEASHFALFFNQGQCCCAGSRTFVHESI
YGEFVEKAKARALKRVVGDPFKNGVEQGPQ
IDSVQFEKIMKYIRSGVESGAQLESGGQRIGSKGYYIQPTVFSNVQDNMLIAKDEIFGPVQSILKFKDLE
EVIRRANATSYGLAAGVFTKNMDTANTLMR
ALQAGTVWINCYDVFDAAIPFGGYKMSGQGRVRGIYSLRSYLQVKAVVTALKNPAWL
>GmALDH2B6
MLLKGMLKISTRAVSAARKAFDEGPWPKMTAYERSRILLRFADLVEKHSDELAALETWNNGKTYEQAAKT
ELPMFVRLFHYYA
GWADKIHGLTVPADGDYHVQTLHEPIGVAGQIIPWNFPLVMFAWKVGPALACGNTIVLKTAEQTPLTALF
VAKLFHEAGLPDGVLNVVSGYGPTAGAALA
SHMDVDKLAFTGSTDTGKVVLELAARSNLKPVTLELGGKSPFIICEDADVDKAVELAHFALFFNQGQCCC
AGSRTFVHERVYDEFLEKSKKRALRRVVGD
PFKKGVEQGPQIDVEQFEKVLRYIRSGIESHATLECGGDRLGSKGFFVQPTVFSNVQDDMLIAQDEIFGP
VQSILKFKDIDEVIRRANKTRYGLAAGVFT
KNVSTANTLMRALRAGTVWINCFDVFDAAIPFGGYKMSGIGREKGIYSLHNYLQVKAVVSPVKNPAWL
>GmALDH2B7
MASSLRISRLISRSFSSTSFFSRGGNGFLGSRHCKYSTSSAIEEEPVKPSVQVEHTQLLIDGKFVDAASG
KTFPTLDPRTGEV
IAHVAEGHSEDVDRAVAAARKAFDHGPWPKMTAYERQRILLRAADLLEKHNDELAALETWDNGKPYEQAA
KIEVPMLVRLIRYYAGWADKIHGLTVPADG
PYHVQTLHEPIGVAGQIIPWNFPLLMFAWKVGPALACGNTIVLKTAEQTPLSALYAAKLFHEAGLPAGVL
NVVSGFGPTAGAALASHMEVDKLAFTGSTD
TGKVVLELAAKSNLKPVTLELGGKSPFIVCEDADVDQAVELAHFALFFNQGQCCCAGSRTFVHESVYDEF
VEKAKARALKRVVGDPFKGGIEQGPQIDSD
QFEKILRYIRSGVESGATLETGGDKLGNKGFYIQPTVFSNVKDGMLIARDEIFGPVQSILKFKDLGEVVQ
RANNTRYGLAAGVFTTNMDTAYTLTRALRV

GTVWINCFDTFDAAIPFGGYKMSGQGREKGEYSLKNYLQVKAVVNPLKNPAWL
>GmALDH2B8
MTSIRQCESDESSLKSAFEEVSTFSLCSHWHRSISGIGASAAADVEPSIAPVQIDHSQLLIDGQFVDAAS
GKTFPTFDPRTGD
VIANVAEGDTEDVNRAVRAARKAFDEGPWPKMTAYERSRIILRFADLLEKHNDEVAAIETWDSGKTYEQA
AKVEIPMVVRLFRYYAGWVDKIHGLTVPAD
GPYHVQTLHEPIGVAGQIVPWNFPLLIFSWMAAPALACGNTVVIKTSEQAPLSALYVSKPFLEAGLPPGV
LNVITGFGATAGASLCSHMDVDKLAFTGST
STGKRQSEVTLELGGKSPFIVCEDADVDAAVEAAHFALFFNQGQCCCAGSRTFVHESIYDEFVEKAKARA
LKRVVGDPFKNGVEQGPQIDSAQFEKIMKY
IRSGVENGATLESGGQRIGSKGYYIQPTVFSNVQDNMLIAKDEIFGPVQSILKFKDLEEVIRRANATSYG
LASGVFTQNMDTANTLMRALRVGTVWINCY
DVFDAAIPFGGYKMSGQGRVRGIYSLRSYLQVKAVVTALKNPAWL
>GmALDH2B9
MTFNNGDAAAASLNKVPTVNFTKLFIDGHFVHSVSGKTFETIDPRTGDVIARISEGDKEDIDIAVKAARH
AFDNGPWPRLPGS
ERGRILLKWAELIEENAEELAALDAIDAGKLYHMCRNLEVPAAANTLRYYAGAADKIHGEVLKMSRDFHA
YTLLEPLGVVGHITPWNFPNTMFYIKVAPS
LAAGCTMVLKPAEQTPLSALFNAHLAKLAGIPDGVINVVPGFGPTAGAALSSHMDVDKVSFTGSTQTGRE
IMQAAAKSNLKQVSLELGGKSPLIIFDDAD
IDKAAELALLGILYNKGEVCVASSRVLVQEGIYDEFEKKLVEKAKAWVVGDPFDPKVQQGPQVDKEQFEK
VLSYIEHGKKEGATLLTGGKTVGNKGYFIE
PTIFSNIREDMLIAQDEIFGPVMALKKFKTIEEAIKSANNTKYGLAAGIVTKNLDTANTVSRSIRAGTIW
INCYFAFGDDVPFGGYKMSGFGKDHGLEAL
HKYLQVKSVVTPLYNSPWL
>GmALDH2C1
MFLSLHIVLINHIATFHLPTPSLRQPPFSLSLARMSALSNSSSSHGNSFLKMPAIKFTKLFINGDFVDSI
SGRTFETIDPRKE
EVIARVSEGDKEDIDIAVKAARQAFDSGPWPRLPGSERAKIMMKWADLVDENIEELAALDTIDAGKLYYI
NKVAEIPSATNALRYYAGAADKIHGDVLKM
NGDFHAYTLLEPIGVVGHIIPWNAPSLSFFIKVSPSLAAGCTMVLKPAEQTPLSALFYAHLAKLAGIPDG
VLNIVPGFGPTAGAAISSHMDIDAVSFTGS
IEVGREVLQAAAWSNLKPVSLELGGKSPLIIFNDADIDKASELALFGIMSNKGEICVAGSRVFVQEEIYD
EFEKKLVEKAKSWVVGDPFDPKSLQGPQAD
RNQLEKILSYIEHGKREGATLLTGGNTVGNKGYYIEPTIFSNVKEDMLIARDEIFGPVLALMKFKTMEEA
IKSANNTKYGLAAGIVTKNLDTANTMSRSI
RAGIVWINCYFTVGSDVPFGGYKMSGFGRDLGLQALHKYLQVKSVVTPIHNSPWL
>GmALDH2C2
MENLSNGHLESFVKIPTIKFTKLFINGEFLDSVSGKTFETVDPRTEEVIAEIAEANKEDVDIAVKAAREA
FDCGPWPRMPGAE
RAKIMLKWSELIEQNAEEIAALDTIDGGKLFSWCKAVDVPEASNILRYYAGAADKIHGDVFKTSRDLHLY
SLMEPVGVVGHIIPWNFPTVMFFAKVAPAL
AAGCTMVIKPAEQTPLSSLFYAHLARLAGIPDGVLNVVPGFGSIAGAAISSHMDIDAVSFTGSTETGRKI
MQAAALSNLKPVSLELGGKSPVLIFDDADV
DKAVDLALFGILHNKGEICVAFSRVYVQEGIYDEFEKKVVEKAKTWVVGDPFDPKVQQGPQTSKAQYDKI
ISYIEHGKSEGATLLTGGKPAGNKGYYIEP
TIFVNVKEDMLIAQEEIFGPVMTLSKFKTIEDAIKKANNSKYGLAAGIVTKNLDIANTVSRSIRAGIIWI
NCFFAFDIDCPFGGYKMSGFGRDYGLEALH

KFLKVKSVATPIYDSPWL
>GmALDH2C3
MAALSNGHDASFFKMPSIKFTKLFINGEFVDSLSGKEFETIDPRTGEVITRIAEGAKEDIDVAVKAARDA
FDYGPWPRMPGAE
RAKIMMKWADLIDQNIEEIAALDAIDAGKLYHWCKAVDIPAAANTIRYYAGAADKIHGEVLKASREFHAY
TLLEPIGVVGHIIPWNFPSTMFVAKVSPSL
AAGCTMVLKPAEQTPLSALFYAHLAKLAGIPDGVLNVVPGFGQTAGAAISSHMDIDKVSFTGSTEVGREV
MRAAANSNLKPVSLELGGKSPVIVFDDADV
DKAAGLALMGILFNKGEICVAGSRVLVQEGIYDEFEKKLVEKANAWVVGDPFDPKVQQGPQVDKKQFEKI
LSYIEHGKKEGATLLTGGKRVGNKGYYIEP
TIFSNVKEDMLIVQDEIFGPVMALMKFKTIEDAIKIANNTRYGLASGIVTKSLDTANTVSRSIRAGIVWI
NCYFAFGDDIPYGGYKMSGFGRDFGMEALH
KYLQVKSVVTPIYNSPWL
>GmALDH2C4
MTSLTNGDAGSLNKVPTIKFTKLFINGDFVDSLSGKTFETIDPRTGDVIARISEGDKEDIDIAVKAARHA
FDNGPWPRLPGSE
RARILLKWAEIIEENAEELAALDAIDAGKLYHMCRNVEVPAAANTLRYYAGAADKIHGEVLKMSREFHAY
TLLEPLGVVGHITPWNFPNTMFYIKVAPSL
AAGCTMVLKPAEQTPLSALFSAHLAKLAGIPDGVINVVPGFGPTAGAALSSHMDVDKVSFTGSTQTGRVI
MQAAAKSNLKQVSLELGGKSPLIIFDDADI
DKATELALLGILYNKGEVCVASSRVFVQEGIYDEFEKKLVEKAKAWVVGDPFDPKVQQGPQVDKEQFEKV
LSYIEHGKKEGATLLTGGKTVGNKGYFIEP
TIFSNIREDMLIAQDEIFGPVMALKKFKTTEEAIKSANNTKYGLAAGIVTKNLDTANTVSRSIRAGTIWI
NCYFAFGDDVPFGGYKMSGFGKDHGLEALH
KYLQVKSVVTPLYNSPWL
>GmALDH2C5
MSSLSNNSSSSHGNSFLQMPPIKFTKLFINGDFVDSLSGRTFETIDPRTEEVIARVSEGDKEDIDIAVKA
ARQAFDSGPWPRL
PASERAKIMMKWADLIDENIEELAALDTVDAGKLNYINKVVEIPSATNALRYYAGAADKIHGEVLKMNGD
FHAYTLLEPIGVVGHIIPWNAPSLSFFIKV
SPSLAAGCTMVLKPAEQTPLSALFYAHLAKLAGIPDGVLNIVPGFGPTAGAAISSHMDIDVVSFTGSIEV
GREVMQAAARSNLKPVSLELGGKSPLIIFN
DADIDKAAQLALFGIMSNKGEICVASSRVFVQEEIYDEFEKKLVEKAKSWVVGDPFDPKSLQGPQADRNQ
LEKILSYIEHGKREGATLLTGGNTVGNKGY
YIEPTIFCNVKEDMLIARDEIFGPVLALMKFKTMEEAIKSANNTKYGLAAGIVTKNLDTANTMSRSIRAG
IVWINCYLTVGSDVPFGGYKMSGFGRDLGL
QALHKYLQVKSVVTPIHNSPWL
>GmALDH2C6
MNSNGYPASSFKIPTVKFTKLFINGHFVDSLSGGEFETIDPRTGEVIARIAEGTKEDIDLAVKASRLAFD
HGPWPRMPAVERA
RIMMKWADLIDQHVEEIAALDAIDAGKLYHMLKAIEIPATANTIRYYAGAADKIHGEVLKPAREFHAYTL
LEPVGVVGHIIPWNFPSIMFVSKVSPCLAA
GCTMVLKPAEQTPLSALFYAHLAKLAGIPDGVLNVVPGFGATAGAAICSDMDIDKVSFTGSTEVGREVMR
AAANSNLKPVSLELGGKSPFIIFDDADLDK
AVELALMAVVYNKQQHIFISDNYLLLLSGFQGEVCAAGSRVFVQEGIYDEFEKRLVEKAKAWVVGDPFDP
NVQQGPQVDKKQFEKILSYIEHGKREGATL
LTGGKRVGNKGYYIEPTIFSNVKEDMLIAQDEIFGPVIALMKFKTIEEAIKSANNSRYGLVAGVVTKSLD
TANTMSRSIRAGVVWINCYFAFENDIPYGG

CKMSGFGKDSGLEALHKYLHVKSVVTPIYNSPWL
>GmALDH2C7
MAALSNGHGSSFFKMPPIKFTKLFINGEFVDSLSGREFETRDPRTGEVITRIAEGAKEDVDVAVKAARAA
FDYGPWPRMPGAE
RAKIMMKWADLVDQNIEEIAALDAIDAGKLYHWCKAVDIPAAASTIRYYAGAADKIHGEVLKASREFHAY
TLLEPIGVVGHIIPWNFPSTMFVAKVSPSL
AAGCTMVLKPAEQTPLSALFYAHLAKLAGIPDGVLNVVPGFGQTAGVAISLHMDIDKVSFTGSTEVGREV
MRAAANSNLKPVSLELGGKSPVIVFDDADV
DKAAELALLGILFNKGEICVAGSRVLVQEGIYDEFEKKLVEKAKAWVVGDPFDPKVQQGPQVDKKQFEKI
LSYIEQGKKEGATLLTGGKRVGNKGYYIEP
TIFSNVKEDMLIVQDEIFGPVMALMKFKTIEDAIKIANNTRYGLASGIVTKSLDTANTVSRSIRAGIVWI
NCYFAFGNDIPYGGYKMSGFGRDFGMEALH
KYLQVKSVVTPIYNSPWL
>GmALDH2C8
MANLSNSHSESFVKIPTVKFAKLFINGEFLDSVSGKTFETVDPRTEEVIAEIAEANKEDVDIAVKAAREA
FDFGPWPRIPGAE
RAKIMLKWSQLIEQNAEEIAALDTIDGGKLFSWCKAVDVPEASNILRYYAGAADKIHGDVFKTSRNLHLY
SLMEPVGVVGHIIPWNFPTVMFFAKVAPAL
AAGCTVVIKPSEQTPLSSLFYAHLSKLAGIPDGVLNVVPGFGSIAGAAISSHMDIDAVSFTGSTETGRKI
MQAAALSNLKPVSLELGGKSPLLIFDDADV
DKAVDLALFGILHNKGEICVAFSRVYVQKGIYDEFEKKVVEKAKTWVVGDPFDPKVQQGPQTSKAQYDKI
LSYIEHGKSEGATLLTGGNPAGNKGYYIEP
TIFANVKEDMLIAQEEIFGPVMTLSKFKTIEDGIKKANSSKYGLAAGIVTKNLDIANTVSRSIRAGIIWI
NCFFAFDIDCPFGGYKMSGFGRDYGLEALH
KFLKVKSVATPIYNSPWL
>GmALDH2C9
MAVFNSLHKTSTRFFVPTGRNSGKWGNVNRFSTAAAVEELIIPQVPITYTKHLINGQFVDADAASGKTFP
TYDPRTGEVIARV
AEGDAEDINRAVSAARKAFDEGPWPKMTAYERCQIILRFADLTWNNGKPYEQWATSELPTFVRLFRYYAA
DKIHGLTVPADGNYHVETLHEPIGVAGQII
PWNFPLLMFAWKVGPALACGNTVILKTAEQTPLTALYVAKAGLPPGVLNVVSGYGPTAGAALASHMDVDK
LAFTGSTETGKVVLELAARSNLKPVILNLE
GNLLSLGQCCCAGSRTFVHERIYDEFLEKAKARALKRVVGDPFIKGVEQGPQVCFASTLRQNIIDCVLSY
HFCSFYSYYKATLECGGDRIGSKGFFVQPT
VFSNVQRVGTVWINCFDVFDAAIPFGGYKMSGISREKGIYSLNNYLQVKAVVSPVKNPAWL
>GmALDH3F1
MDIGGGVEEPVRELRQYFKTGKTKSVTWRKNQLTSLIDLVHENEDAIFKALHKDLGKHPVEAYRDEVGGV
EKSASKALSCVEK
WMAPKKSDIPFLFFPAKGEVLSEPLGVVLIISSWNFPIILALDPIIGAISAGNVVVIKPSEQAPACSSFL
ANTIPRYLDSNAIKVIEGGEDVCEQLLRQK
WDKIFFTGSPRVASVVMSAAAKNLTPVTLELGGKCPAILDSLPNPSEFELAVKRIVGGKWGPCSGQACIG
IDYLLVEEKFSSAVIKLLKKFIRRFYGENP
VESKVISRIINKQHFERLCNLLKDPLVAASIVHGGSVDEENLFIEPTILLDPPLDSEIMAEEIFGPLLPI
ITLDKIQESIEFINAKPKPLAIYAFTKDET
FKRKILSETSSGSVVFNDTMVQFLCDTLPFGGVGQSGLGRYHGKYSFDTFSHEKAVMHRKLFLEIEPRYP
PWNKFKLEFIRLAYRLNYFGLVLHMLGLKR
YN
>GmALDH3F2

MSGEETQRNVFGAETASSLVKELRDNFGKGTTRSYEWRVSQVKALLKAVVENEDQIVGALCSDLAKPPLE
TVVYEIGMFQNSC
EVILKELKHWMTPEKVKTSIRTFPSSAEIVPEPLGVVLVISAWNYPILLSLDPVVGAIAAGNAVVLKPSE
IAPATSSVLAKLIEKYMDNSFVRVVEGAVD
ETTALLQQKWNKIFYTGNGRVGKIVMTAAAKHLTPVVLELGGKSPVVVDSNNNLLVAARRIIAGKWGLNN
GQACISPDYVITTKDYAPKLVDTLKTELES
FYGRNPLESEDLSRIVSSNHFARLSKLLNDDKVSGKIVYGGEKDEKKLRIAPTILLDVPQDSSIMGEEIF
GPLLPIITVNKLEESIDVINSGAKPLAAYV
FTTDNKFKEQFVKNVSAGGLLVNDTALHLVVDTLPFGGVGESGMGAYHGKFSFDAFTHKKAVLYRSFAGD
SAIRYPPYTDTKLRLMKALVGGRILGIIRA
LFGWS
>GmALDH3H1
MSVEEMQSQKRNVFDAETASSLVKELRDNFGSGRTRSYEWRVSQVKALLKAVVDNEEQIVDALRSDLAKP
PLETIVYEVGMFK
NSCEVILKELKQWMKPEKVKTSIRTFPSSAEIVPEPLGVVLVISAWNYPILLSLDPVVGAIAAGNAVVLK
PSEIAPASSSLLLKLIEKYCDNSFIRVVEG
AVDETTALLQQKWDKIFYTGNGKVGRIVMTAAAKHLTPVVLELGGKSPVVVDSNVDLQIAARRIISGKWG
LNNGQACISPDYVITTKDCAPKLVDALKTE
LEKCYGKNPLESEDLSRIVTSNHFARLSKLLDDDKVAGKIVYGGEKDEKKLRIAPTLLLDVPRDSLIMGE
EIFGPLLPIITVNKVEESIDLINSGTKPLA
AYIFTTNKKLKEQFVMNVPAGGLLVNDTVLHLVVDTLPFGGVGESGMGAYHGKFSFDAFTHKKAVLYRSF
AGDSSLRYPPYTDTKLRLMKALIGGRFLGI
IRALFGWS
>GmALDH3H2
MSSTPQDSVKTTASAKNTAFDAEAASRLVNELRRNFASNKTRSYEWRLSQLNALEKLVVVHEQEIVDALR
NDLGKPPLETVAY
EIAMLKNSCRIALKELKHWMTPEKVKTSIATFPSSAEIVSEPLGVVLVISAWNYPFLLSLDPVVGAIAAG
NAVVLKPSEIAPATSSLLAKLIGDYLDNSC
IRVVEGAVDETSALLQQKWDKIFYTGNGRVARIVMAAASKHLTPVVLELGGKSPVVVDSNINLKVATRRI
IAGKWGSNNGQACISPDYIITTKDYAPKLV
DALKTELEKFYGKNPLESKDLSRVVNSNHFNRLTKLLDDDKVSGKIVYGGQKDENKLKISPTVLLDVPRD
SLIMNEEIFGPLLPILTVDKLEESFDVINS
GPKPLAAYIFTNNKKLKEQFVMTISAGGLVVNDTTLHLAVHTLPFGGVGESGVGAYHGKFSFEAFSHKKA
VLYRKFIGDAPVRYPPYTNTKMRLLKAIIG
GGIHGIVRALFGW
>GmALDH3H3
MSSSTPDSDKTTTSSKKSAFDALAASRLVTELRGNFASGKTRSYEWRLLQLNAIAKLVVDHEQEIVDALR
NDLGKPPLETVAY
EIAMLKNSCRIALKELKHWMTPEKVKTSIATFPSSAEIVSEPLGVVLVISAWNYPFLLSLDPVIGAIAAG
NAVVLKPSEIAPATSSLLAKLLGDYLDNSC
IKVVEGAVDETSALLQQKWDKIFYTGNGRVARIVMAAASKHLTPVVLELGGKSPVVVDSNINLKVATRRI
IAGKWGSNNGQACISPDYIITTKDYAPKLV
DALKTELEKFYGKNPLESKDLSRIVNSNHFNRLTKLLDDDKVSGKIVYGGEKDESKLKISPTVLLDVPRD
SLIMNEEIFGPLLPILTVDKIEESFDVINS
GSKPLAAYIFTNTKKLKEQFVMTISAGGLVVNDTTLHLAVHTLPFGGVGESGVGAYHGKFTFEAFSHKKA
VLYRRFIGDAPVRYPPYTNTKMRLLKALIG
GGILGIIRALFGW
>GmALDH3H4

MKSLCLGPFLAASAPVGRRAYGGHLSRKCFQKQLHFHSRCVAFSSFICSATISVMPELEEKQVFDGEKAN
LLVKDLRKSFDSG
MTKSYGWRVSQLEAIAKMLEEKEKEITEALYKDLGKPRLEAFITEISQAKSSCSEALKELKEWMKPEKVN
TSITTYPSSAEIVPEPLGVVLVISTWNFPF
LLSMDPVIGAISAGNAVVLKPSEISPATSSLLANLIEQYLDNSTIRVVEGAIPETSALLDQKWDKILYTG
SARVGRIVMAAAAKHLTPVILELGGKCPAV
VESDVNLQVTARRIIAGKWACNSGQACISVDYIITRKEFAPKLVDALKEELEQFFGKDPMESKDMSRIVS
PNQFARLVNLLDEDKVSDKIVLGGQRDEKK
LKIAPTIILGVPEDAMIMQEEIFGPIMPIVTVDNIEDCYSIIKSKPKPLAAYLFTNNEQLKKDYVDKISS
GGMLINDAVIHVATRGLPFGGVEESGMGCY
HGKFSFDSFSHRKSVLYRSFDADSTIRYPPYTPQKEKLLKALISGNIVQIILSLLGWS
>GmALDH3I1
MEITMQTLERDLNDTRGYYESGKTKEESWRESQLKGLRRFLLEKQVDIMNALMHDLGKHQLEAFRDEIGT
LIKTVNLALKSLK
DWMSGKKAALPQLALLTSAEIVPEPLGLVLIISSWNFPIGISLEPLIGAVAAGNAAVLKPSELSPACSSL
LASSLPTYLDDKAIKVIQGGPQETQQLLEQ
RWDKIFFTGSARVGRIVMSSAVKHLTPVTLELGGKCPAVVDSLSSSWDKEVTVKRIIVGKYGTCAGQACI
TIDYVLVEKGYCLKLVELMKVWIKKMFGQN
PRKSKTIAKIVNKHHFSRLKNLLADKQVKGSVVYGGSMDEQNLFIEPTILVDPPLEAAIMSEEIFGPLLP
IITVEKIEDSIKFINARPKPLALYVFTKNH
TLQRRMISETSSGSVTINDAVLQYAADTIPFGGVGESGFGMYHGKFSFDTFSHQKAIVRRSFLTDFWYRY
PPWTLNKLQLLEVSYNYDYLGLLLVLLGLK
RPSKRLIADHV
>GmALDH3J1
MEIIMPSLERDLNDTRGYYESGKTKEASWRESQLKGLRRFLIEKQEDIMNALMHDLGKHQLEAFRDEIGT
LIKTLNLALKSLK
HWMSGKKAALPQLALLTSAEIVPEPLGVVLIISSWNFPFGISLEPLIGAVAAGNAAVLKPSELSPACSSL
LASNLSTYLDNKAIKVIQGGPKETQQLLEQ
RWDKIFFTGSAHVGKIVMSAAVKHLTPVTLELGGKCPAVVDSLSSSWNIEVAVKRIIVGKYGACAGQACI
AIDYVLVEKVYCFKLVELMKVWIKKMCGEN
PQQSKTIAKIVNKHHFSRLKNLLADKKVKESVIYGGSMDEQNLFIEPTILVDPPLEAAIMSEEIFGPLLP
IITVEKIEDSIKFINSRPKPLALYVFTKNQ
TLQRRMISETSSGSVTINDAILQYAVDTVPFGGVGESGFGMYHGKFSFDTFSHQKAIVRRSFLTDFWYRY
PPWTLNKLQLLEVSYNYDYLGLLLVLLGLK
RPSKRLISDHV
>GmALDH3J2
MKYTGEALGRDLENVRKYYGSGKTKEASWRESQLKGLHNFLVEKEEEILRALKHDLGKHYVEAFRDEVGT
LMKTLNLASKSLK
NWMAGKEAKLPRIALLSSAEIVPEPLGLVLIISSWNFPFGLSLEPLIGAIAAGNSVVLKPSELSPTCSSL
LATFLPTYLDNNAIKVIQGGPEVGELLLQQ
RWDKIFFTGSARVGRIVMSAAAVHLTPVTLELGGKCPAIIDSLSSSWDKEVAVKRILVAKFGACGGQACI
AIDYVLVEKSFSSTLVTLMKEWIKKLFGEN
PKVSNTIARIVNKNHFMRLKNLLTEPRVKESVVYGGSMDENDLFIEPTILLDPPLDSAIMAEEIFGPVLP
IITVEKIEESVEFISSRPKALAIYAFTKNQ
TLQRRLVSETSSGSLVFNDAILQYVADTLPFGGVGECGFGKYHGKFSFDAFSHHKAVARRSYLTDFWFRF
PPWTLNKLQLLEVSYNLDYLGILLVLLGLK
KSKRSLFQACN
>GmALDH3J3

MEYSVETLERDLKNTRKYYGSGKTKEAPWRESQLKGLHNFLVEKEEEIVTALKHDLGKHYVEAFRDELGT
LMKTLNLATKSLK
NWMAGKEAKLPRIALLSSAEIVPEPLGLVLIISSWNFPFGLSLEPLIGAVAAGNSVVLKPSELSPTCSSL
LATFLPTYLDNNAIKVIQGGPEVGKLLLQQ
RWDKIFFTGSARVGRIVMSAAAVHLTPVTLELGGKCPALIDSLSSSWDKEVAVKRILVAKFGSCAGQACI
AIDYVLVEKSFSSTLVTLMKEWIKKMFGEN
PKASNSIARIVNKNHFMRLQNLLTEPRVKESVVYGGSMDENDLFIEPTILLDPPLDSAVMAEEIFGPVLP
IITLEKIEDSVEFISSRPKALAIYAFTKNQ
TLQRRMVSETSSGSLVFNDAILQYVADTLPFGGVGECGFGKYHGKFSFDAFSHHKAVARRSYLTDFWFRF
PPWTLDKLQLLEVSYNLDYLGILLVLLGLK
KSKRSLFQACN
>GmALDH3J4
MRPPHYKYSCPHEWEGSVEENKKEKENNQYDNFAILVVDQGRSIAMDIGGEVEETVRELRQYFKTGKTKS
VTWRKNQLTALLD
LVHENEDAIFKALHQDLGKHPVEAYRDEVGGVEKSASNALSCVEKWMAPKKSDIPFLFFPAKGEVLSEPL
GVVLIFSSWNFPIILTLDPIIGAISAGNVV
VIKPSEQSPASSSFLATTIPRYLDSNAIKVIEGGPDVCEQLLLQKWDKIFFTGSPRVASVVMSAAAKNLT
PVTLELGGKCPAILDSLPNPLEFKLAVKRI
VGGKWGPCSGQACIAIDYLLVEKKFSYALIELLKKIIRRFYGENPVESKVISRILNKQHFERLCNLLKDP
LVAASIVHGGSVDEENLFIEPTILLDPPLD
SQIMSEEIFGPLLPIITMDKIQESIEFINAKPKPLAIYAFTKDETFKRNILSETSSGSVVFNDTMVQFLC
DTLPFGGVGQSGFGRYHGKYSFDTFSHEKA
VMHRKLFLEIEPRYPPWSKFKLEFIRLAYRLNYFGLLLHMLGLKRYK
>GmALDH5F1
MAALNLCRMALRSSKLLYRPYNLLSVQLQMQMQPSSPPLTRKMSTDAQSIASQLNSSGLLRTQGLIAGKW
SDAYDGKTIKVYN
PATGESVVDVACMGGRETNDAISAAYDAYGSWSKTTAAERSKLLRKWYDLLMVHKEELAQLITLEQGKPL
KESVGEIVYGAGFIEFAAEEAKRIYGDIVP
APFSDRRLFVLKQPVGVVGAITPWNFPLAMITRKVGPALACGCTVVIKPSELTPLTALAAVELSIQAGIP
PGVVNVVMGNAPDIGDALLASPQVRKITFT
GSTAVGKKLMAGSAETVKKVSLELGGNAPCIVFDDADLDVAVKGTLAAKFRNSGQTCVCANRIIVQEGIY
EKFANALRDAVQNMKVGDGFSEGVSQGPLI
NEAAVKKVESLIHDATSKGAKVILGGKRHSLGLTFYEPTVISDVNSDMHISREEAFGPVAPLLRFKTEEE
AIRIANDTNAGLGSYVFTNSIQRSWRVAEA
LEYGLVGVNEGVISTEVAPFGGFKQSGLGREGSKYGMDEYLEIKYVCFGNMNKE
>GmALDH5F2
MAALNLCRMALRSSKLLSRPYHRLSVQLQMQMQPSSPPLTRKMSMDAQSVASQLNSSGLLRTQGLIGGKW
SDAYDGKTIKVYN
PATGESIVDVACMGGRETNDAISAAYDAYGSWSKTTAAERSKFLRKWYDLLMVHKEELAQLITLEQGKPL
KESVGEINYGAGFIEFAAEEAKRIYGDIIP
APLSDRRLFVLKQPVGVVGAITPWNFPLAMITRKVGPALACGCTVVIKPSELTPLTALAAAELSIQAGIP
PGVVNVVMGNAPDIGDALLASPQVRKITFT
GSTAVGKKLMAGSAETVKKVSLELGGNAPCIVFDDADLDVAVKGTLAAKFRNSGQTCVCANRIIVQEGIY
EKFANALRDTVQNMKVGDGFSEGVAQGPLI
NEAAVKKVESLIHDATSKGAKVILGGKRHSLGFTFYEPTVISDVNSDMRISREEAFGPVAPLLRFKTEED
AIRIANDTNAGLGSYIFTNSIQRSWRVAEA
LEYGLVGVNEGVISTEVAPFGGFKQSGLGREGSKYGMDEYLEIKYVCLGNMHKA
>GmALDH6B1

MLRLSIQRVRKLNFLSPQISALGRSHLSTAAEPSSSKSNPPRVPNLIGGSFVDSKASTVIDVINPATQEV
VSQVPLSTDEEFK
EAVSAAKKAFPSWRNTPITTRQRVMLKLQELIRRDMDKLALNVTTEQGKTLKDAQGDVFRGLEVVEHACG
MATLQMGEYVSNVSHGIDTYSIREPLGVCA
GICPFNFPAMIPLWMFPMAITCGNTFVLKPSEKDPGASVMLAELALEAGLPEGVLNIVHGTHDIVNAICD
DDDIKAISFVGSNVAGMHIYSRAAAKGKRV
QSNMGAKNHAIVMADANVDATLNALVAAGFGAAGQRCMALSTVVFVGGSKPWEDKLLEHAKALKVNAGTE
PDTDLGPVISKQAKERIHRLVQSGVESGAR
LLLDGRNIVVPGYESGNFIGPTILSDINANMECYKEEIFGPVLLFMEADSLEEAINIINSNKYGNGASIF
TTSGVAARKFQTEIEAGQVGINVPIPVPLP
FFSFTGNKASFAGDLNFYGKAGVNFYTQIKTITQQWKDSTGGSKINLAMPTSQK
>GmALDH6B2
METRMLRLSIQRVRKLNFLRPQISALGRSHLSTAAEPSSSKSNPPRVPNLIGGSFVDSKASTVIDVINPA
TQEVVSQVPLSTH
EEFKAAVSAAKEAFPSWRNTPITTRQRVMLKLQELIRRDMDKLALNVTTEQGKTLKDAQGDVFRGLEVVE
HACGMATLQMGEYVSNVSHGIDTYSIREPL
GVCAGICPFNFPAMIPLWMFPMAVTCGNTFVLKPSEKDPGASVMLAELALEAGLPEGVLNIVHGTHDIVN
AICDDENIKAISFVGSNVAGMHIYSRAAAK
GKRVQSNMGAKNHAIVMPDANVDATLNALVASGFGAAGQRCMALSTVVFVGGSKPWEDKLLERAKALKVN
AGTEPDTDLGPVISKQAKERIHRLVQSGVE
SGARLLLDGRNIVVPGYESGNFIGPTILSDINANMECYKEEIFGPVLLFMEADSLEEAINIINSNKYGNG
ASIFTTSGVAARKFQTEIEAGQVGINVPIP
VPLPFFSFTGNKASFAGDLNFYGKAGVNFYTQIKTITQQWKDSTGGSRINLAMPTSQK
>GmALDH6B3
MANSHLSTPSELFSRQHKPPRVPNLIGGSFLDSKSLTFIDVINPATQEVVSQVPCTTDEEFKAAVSAAKK
AFPSWRKTPITKR
QRVMLKFQELIRRDMDKLALNVTTEQGKTLKDAQGDVFRGLEVVEHACGMATLQMGEYVSDVSSGIDTYS
IREPLGVCAGICPFNFPAMIPLWMFPVAVT
CGNTFILKPSEKVPGASVMLAELAMEAGLPEGVLNIVHGTHDIVNAICDDDDIKAISFVGSNVAGMHIYA
RAAAKGKRVQANMGAKNHAVVMPDASVDAT
VNALVAAGFGAAGQRCMALSTVVFVGDSKLWESKLVEHAKALKVNVGTEPDADLGPVISKQAKERIHRLI
QSGVESGARLVLDGRNIVVPGYESGNFIGP
TILSDVTANMECYKEEIFGPVLLLTEADNLEEAINIINENKYGNGASIFTTSGVAARKFQTEIEAGQVGI
NVPIPVPLPFFSFTGNKASFAGDLNFYGKA
GVNFYTQIKTVTQQWKDSASESKINLAMPTSQKS
>GmALDH7B1
MGSDNHQNLEFLKEIGLGSSNIGSYINGQWKATGSSVTSVNPSNNQSIAQVTEATLQDFEEGLRACSEAA
KTWMTIPAPKRGE
IVRQIGEALRAKLDPLGRLVSLEMGKILPEGIGEVQEIIDMCDYCVGLSRQLNGSIIPSERPDHMMFEVW
NPLGIVGVISAFNFPCAVLGWNACIALVCG
NCVVWKGAPTTPLITIAVTKLVAEVLERNKLPGAIFTSFCGGADIGQAIAKDTRIPLVSFTGSSKVGLMV
QQTVNERFGKCLLELSGNNAIIVMDDADIK
LAVRSILFAAVGTTGQRCTTCRRLFLHESIYTDVLDQLVEVYKQVKIGNPLEKGTLVGPLHTRTSVENFQ
KGISVIKSQGGKILTGGSVLESGGNFVQPT
IVEISPDAPVVKEELFGPVLYVMKFQTLEEAIALNNSVPQGLSSSIFTQRPGTIFKWIGPRGSDCGIVNA
NIPTNGAEIGGAFGGEKATGGGREAGSDSW
KQYMRRSTCTINYGSELPLAQGINFG
>GmALDH7B2

MGSDNTNLEFLKEIGLGSSNIGSYINGQWKATGSSVTSVNPSNNQSIAQVTEATLQDYEEGLQACSEAAK
TWMTIPAPKRGEI
VRQIGEALRAKLDPLGRLVSLEMGKILPEGIGEVQEIIDMCDYCVGLSRQLNGSIIPSERPDHMMFEVWN
PLGIVGVITAFNFPCAVLGWNACIALVCGN
CVVWKGAPTTPLITIAVTKLVAEVLERNKLPGAIFTSFCGGADIGQAIAKDTRIPLVSFTGSSKVGLMVQ
QTVNERFGKCLLELSGNNAIIVMDDADIKL
AVRSILFAAVGTAGQRCTTCRRLFLHESIYADVLDQLIGVYKQVKIGNPLEKGTLVGPLHTPTSVENFQK
GISVIKSQGGKILTGGSVLESAGNFVQPTI
VEISPDAPVVKEELFGPVLYVMKFQTLEEAIALNNSVPQGLSSSIFTQRPGTIFKWIGPRGSDCGIVNAN
IPTNGAEIGGAFGGEKATGGGREAGSDSWK
QYMRRSTCTINYGSELPLAQGINFG
>GmALDH10A1
MSIPIPHRQLFIDGDWKVPVLKNRIPIINPSTQHIIGDIPAATKEDVDLAVAAAKAALSRNKGADWASAS
GSVRARYLRAIAA
KITEKKPELAKLEAIDCGKPLDEAAWDIDDVAGCFEFYADLAEKLDAQQKAHVSLPMDTFKSYVLKEPIG
VVALITPWNYPLLMATWKVAPALAAGCAAI
LKPSELASVTCLELAEICKEVGLPPGVLNILTGLGPEAGAPLAAHPDVDKIAFTGSSATGSKIMTAAAQL
IKPVSLELGGKSPIIVFEDVDLDKAAEWTI
FGCFWTNGQICSATSRLIESIATEFLNRIVKWVKNIKISDPLEEGCRLGPIVSEGQYEKILKFISNAKSE
GATILTGGSRPEHLKKGFFVDQLEEVFGPV
LCVKTFSTEEEAIDLANDTVYGLGSAVISNDLERCERITKAFKAGIVWINCSQPCFTQAPWGGIKRSGFG
RELGEWGLDNYLSVKQVTQYISDEPWGWYQ
SPSRL
>GmALDH10A2
MAISIPSRQLFIDGEWKVPLLNNRFPIINPATEDIIGHIPAATKEDVDLAVDAAKRAFSHNKGKDWSSAP
GSVRARYLRAIAS
KITEKKDELGKLEAIDCGKPLDEALADLDDVIGCFNYYAELAEGLDAKQNAPVSLPMETFKSYVLKEPIG
VVALITPWNYPLLMATWKVAPALAAGCTAI
LKPSELASVTCLELAEICREVGLPPGVLNIVTGLGNEAGAPLSSHPDVDKISFTGSSATGSRIMTAAAQL
TKPVSLELGGKSPIIVFEDVDLDKTAEWTI
FGCFFTNGQICSATSRLIVHESIATEFVNRLVQWAKNIKISDPFEEGCRLGPIVSEGQYKKVLNCISTAK
SEGATILIGGSRPEHLKKGYFVEPTIITDV
TTSMQIWREEVFGPVLCVKTFSTEEEAIELANDTHYGLGSAVMSKDLERCERISKAIQAGIVWINCAQPS
FIQAPWGGVKRSGFGRELGEWGLENYLSVK
QVTKYISDEPWGWYQSPSKL
>GmALDH11A1
MAAGTGLFAEILDGDAYKYYADGEWKKSASGKSVSIINPTTRKTQYKVQACSQEEVNKVMDLAKSAQKLW
AKTPLWKRAELLH
KAAAILKEHKTPIAECLVKEIAKPAKDAVMEVVRSGDLVSYTAEEGVRILGEGKFLVSDSFPGNERTKYC
LTSKIPLGVILAIPPFNYPVNLAVSKIAPA
LIAGNSIVLKPPTQGAVSALHMVHCFHLAGFPKGLINCVTGKGSEIGDFLTMHPGVNCISFTGGDTGISI
SKKAGMIPLQMELGGKDACIVLEDADLDLV
AANIIKGGFSYSGQRCTAVKVVLVMESVADALVEKVKAKVAKLTVGPPEDDCDITPVVSESSANFIEGLV
LDAKEKGATFCQEYKREGNLIWPLLLDNVR
PDMRIAWEEPFGPVLPVIRINSVEEGIHHCNASNFGLQGCVFTKDVNKAIMISDAMETGTVQINSAPARG
PDHFPFQGIKDSGIGSQGITNSINMMTKVK
TTVINLPSPSYTMG
>GmALDH11A2

MAAGTGLFAEILDGDVYKYYADGEWKKSASGKSVAIINPTTRKTQYKVQACSQEEVNKVMDLAKSAQKLW
AKTPLWKRAELLH
KAAAILKEHKAPIAECLVKEIAKPAKDAVTEVVRSGDLVSYTAEEGVRILGEGKFLVSDSFPGNERTKYC
LTSKIPLGVILAIPPFNYPVNLAVSKIAPA
LIAGNSIVLKPPTQGAVSALHMVHCFHLAGFPKGLINCVTGKGSEIGDFLTMHPGVNCISFTGGDTGIAI
SKKAGMIPLQMELGGKDACIVLEDADLDLV
AANIIKGGFSYSGQRCTAVKVVLVMESAADALVEKVKAKVAKLTVGPPEDDCDITPVVSESSANFIEGLV
LDAKEKGATFCQEYKREGNLIWPLLLDNVR
PDMRIAWEEPFGPVLPVIRINSVEEGIHHCNASNFGLQGCVFTKDVNKAIMISDAMETGTVQINSAPARG
PDHFPFQGIKDSGIGSQGITNSINMMTKVK
TTVINLPSPSYTMG
>GmALDH11A3
MAGSGTFAEIIDGDVFKYYAQGHWNKSSSGKFVPIINPTTRKTHFKVQACTQKEVNRVMESAKTAQKSWA
KTPLWKRAELLHK
AAAILKEHKAPIAECLVKEIAKPAKDAVTEVIRSGDLVSYCAEEGVRILGEGKFLVSDSFPGNERTKYCL
TSKIPLGVVLAIPPFNYPVNLAVSKIAPAL
IAGNSIVLKPPTQGAVAALHMVHCFHLAGFPEGLISCVTGKGSEIGDFLTMHPGVNCISFTGGDTGIAIS
KKAGMVPLQMELGGKDACIVLEDADLDLAA
ANIVKGGFSYSGQRCTAVKVALVMESVANTLVKRINDKIAKLTVGPPEIDSDVTPVVTESSANFIEGLVM
DAKEKGATFCQEYVREGNLIWPLLLDNVRP
DMRIAWEEPFGPVLPVIRINSVEEGIHHCNASNFGLQGCVFTRDINKAMLISDAMETGTVQINSAPARGP
DHFPFQGLKDSGIGSQGITNSINMMTKVKT
TIINLPAPSYTMG
>GmALDH12A1
MCLLRVLSAEFIFTICRFAHSLPFATVQAEEISDSRPAEVLNLVQGKWAGSSNWNTVVDPLNGDSFIKVA
EVDETGIQPFVES
LSSCPKHGVHNPFKAPERYLMFGEISAKAAHMLSLPKVSDFFTRLIQRVSPKSYQQAFGEVYVTQKFLEN
FCGDQVRFLARSFGVPGNHLGQQSHGFRWP
YGPVAIITPFNFPLEIPVLQLMGALYMGNKPVLKVDSKVSIVMDQMLRLLHNCGLPLEDVDFINSDGKTM
NKLLLEANPRMTLFTGSSRVAEKLAVDLKG
RVKLEDAGFDWKILGPDVLQEDYIAWVCDQDAYACSGQKCSAQSLLFMHENWSKTSLLSKLKDLADRRKL
ADLTVGPVLTVTTDSMLEHINKLLEIPGSK
LLFGGQPLEDHSIPPIYGAMKPTAVYVPLEEIMKAKNFELVTREIFGPFQIVTDYKSSQLSVVLDALERM
HNHLTAAVVSNDPLFLQEVIGQSVNGTAYA
GLRARTTGAPQNHWFGPAGDARGAGIGTPEAIKLVWSCHREIIYDFGPVPKNWEVPPST
>GmALDH12A2
MFMFLVSRVTKDSISRNRNAFASFAFSSRCAHSLSFATVEAEEISGSRPAEVLNLVQGKWVGSSNWNTIA
DPLNGDSFIKVAE
VDETGIQPFIKSLSSCPKHGVHNPFKAPERYLMYGDISTKAAHMLSLPKVSDFFTKLIQRVSPKSYQQAF
GEVYVTQKFLENFCGDQVRFLARSFGVPGN
HLGQQSHGFRWPYGPVAIITPFNFPLEIPVLQLMGALYMGNKPVLKVDSKVSIVMEQMLRLLHTCGLPAE
DVDFINSDGKTMNRLLLEANPRMTLFTGSS
RVADKLAVDLKGRVKLEDAGFDWKILGPDVHQEDYIAWVCDQDAYACSGQKCSAQSLLFMHENWSKTSLL
SKLKDLAERRKLEDLTIGPVLTCTTGMMLE
HKNKLLEIPGSKLLFGGSPLENHSIPPIYGAIKPTAVYVPLEEIMKDKNFDLVTKEIFGPFQVITDYKNS
QLSVVLDAVERMHNHLTAAVVSNDPLFLQE
VVGNSVNGTTYAGLRARTTGAPQNHWFGPAGDARGAGIGTPEAIKLVWSCHREVIYDFGPVPKDWKTPQS
T

>GmALDH12A3
MFKLLVSRAARVSTPHNHNAFASFAFSRYAHSLPFATVEAEEISGSRAAEVLNLVQGKWVGSSNWNTVVD
PLNGDSFIKVAEV
DETGIQPFVESLSSCPKHGAHNPFKAPESLLGVNGMVVLILFRYLMFGEISAKAAHMLSLPKVLDFFTRL
IQRVSPKSYQQAFGEVYVTQKFLENFCGDQ
VRFLARSFAVPGNHLGQQSHGFRWPYGPVAIITPFNFPLEIPVLQLMGALYMGNKPVLKVDSKVSIVMEQ
MLRLLHTCGLPLEDVDFINSDGKTMNKLLL
EGNPRMTLFTGSSRVAEKLAVDLKGRVKLEDAGFDWKILGPDVHQEDYVAWVCDQDAYACSGQKCSAQSL
LFMHENWSKTSLLSKLKDLAERRKLADLTI
GPVLTVTTDSMLEHVNKLLEIPGSKLLFGGSPLENHSIPPIYGAIKPTAVYVPLEEIMKDKNFELVTKEI
FGPFQVITDYQNSQLAVVLDALERMHNHLT
AAVVSNDPLFLQEVIGKSVNGTTYAGLRARTTGAPQNHWFGPAGDARGAGIGTPEAIKLVWSCHREIIYD
FGPVPKNWEVPPST
>GmALDH18B1
MELLQNGHKNFVSIKPSELPLTNGAALTLLNSLSKTQYLGNIDPSRVFVTKVKRIIVKVGTAVVTRSDGR
LALGRIGALCEQL
KELSSQGYEVILVTSGAVGLGRQRLRYRKLANSSFSDLQKPQEELDGKACAAVGQSSLMALYDTMFSQLD
VTSSQLLVNDGFFRDSGFRKQLSDTVNSLL
DLRVIPIFNENDAVSTRKAPYEDSSGIFWDNDSLAGLLALELKADLLVLLSDVEGLYSGPPSDPNSRLIH
TYIKEKHQGEITFGDKSRLGRGGMTAKVNA
AVCAAHAGIPVIITSGYATNNIIRVLQGERIGTVFHKDAHLWTNIKEVSAREMAVAAREGSRRLQILKSE
ERRKILLAIADALETSESMIRHENEADVAD
AVATGYEKSLMSRLILKQEKISSLAKSVRMLADMEEPIGQILKRTELVDKLILEKISCPLGVLLVIFESR
PDALVQIAALAIRSGNGLLLKGGKEARRSN
AILHKVITSVMPDTVGDKLIGLVTSRDEILDLLKLDDVIDLVVPRGSNKLVSQIKESTKIPVLGHADGIC
HVYVDKSANIDMAKQIVRDAKTDYPAACNA
METLLVHKDLSNNGGLHELVLELQREGVKMFGGPRASGLLNIAETNTFHHEYSSLACTVEIVEDVFAAID
HINQHGSAHTECIVTEDSEVAETFLSQVDS
AAVFHNASTRFCDGARFGLGAEVGISTSRIHARGPVGVEGLLTNRWILRGSGHVVDGDQGINYTYKELPL
KA
>GmALDH18B2
MADPSRSFMKDVKRVIIKVGTAVVTREEGRLAVGRLGALCEQIKQLNSLGYDIILVSSGAVGIGRQRLRY
RKLINSSFADLQK
PQHELDGKACAAVGQNSLMALYDTLFTQLDVTSAQLLVTDNDFRDKDFRKQLTETVKSLLSLKVIPVFNE
NDAVSTRKAPYEDSSGIFWDNDSLSALLAL
ELKADLLVLLSDVEGLYSGPPSDPHSKLIHTYIKEKHQNEITFGDKSRVGRGGMTAKVKAAVHAADAGIP
VVITSGFAAENIINVLQGQRIGTLFHKDAH
EWVQVKEVDAREMAVAARECSRRLQAISSEERNQILHKIADALEANEKIIRTENEADIAVAQEAGYEKSL
VARLAIKPGKIASLANNMRIIANMEDPIGQ
VLKRTELSDGLILEKTSSPLGVLLIVFESRPDALVQIASLAIRSGNGLLLKGGKEARRSNAILHKVITEA
IPDTVGGKLIGLVTSREEIPELLKLDDVID
LVIPRGSNKLVSQIKSSTKIPVLGHADGVCHVYVDKSANVEMARRIVLDAKIDYPAACNAMETLLVHKDL
IEKGWLNDIVVDLRTEGVKLYGGPRASSLL
NIPQAQTFHHEYSSLACTVEIVDDVYAAIDHINLYGSAHTDSIVAEDKEVANVFLRQVDSAAVFHNASTR
FSDGARFGLGAEVGISTSRIHARGPVGVEG
LLTTRWILKGSGQVVDGDKGIVYTHKDIAT
>GmALDH18B3

MELLQNGHKNLVSIKPSELPLLNGAALTLLNSLSETHEYYGNIDPSRVFVTKVKRIIVKVGTAVVTRSDG
RLALGRIGALCEQ
LKELSSQGYEVILVTSGAVGLGRQRLRYRKLANSSFSDLQKPQGELDGKACAAVGQSSLMALYDTMFSQL
DVTSSQLLVNDGFFRDSGFRKQLSDTVNSL
LDLRVIPIFNENDAVSTRKAPYEDSSGIFWDNDSLAGLLALELKADLLVLLSDVEGLYSGPPSDPNSKLI
HTYVKEKHQGEITFGDKSRLGRGGMTAKVN
AAVCAAHAGIPVIITSGYATNNIIRVLQGERIGTVFHKDAHLWTNIKEMSAREMAVAAREGSRQLQILKS
EDRRKILLAIADALEKNESMIRHENEADVA
DAVVAGYEKSLISRLTLKQEKISSLAKSVRLLADMEEPIGQILKRTELVDKLILEKTSCPLGVLLVIFES
RPDALVQIAALAIRSGNGLLLKGGKEARRS
NAILHKVITSVMPDTVGDKLIGLVTSRDEIPDLLKLDDVIDLVVPRGSNKLVSQIKESTKIPVLGHADGI
CHVYVDKSANFDMAKQIVRDAKTDYPAACN
AMETLLIHKDLSNNGGLNELVLELQREGVKMFGGPRASGLLNIAETNTFHHEYSSLACTVEIVEDVFAAI
DHINQHGSAHTECIVTEDSEVAETFLSQVD
SAAVFHNASTRFCDGARFGLGAEVGISTSRIHARGPVGVEGLLTNRWILRGSGHVVDGDQGIDYTYKELP
LKA
>GmALDH18B4
MADRSRSFMKDVKRVVIKVGTAVVTREEGRLAVGRLGALCEQIKQLNSLGYDIILVSSGAVGIGRQRLRY
RKLINSSFADLQK
PQLELDGKACAAVGQNSLMALYDILFTQLDVTSAQLLVTDNDFRDEDFRKQLTETVKSLLSLKVIPVFNE
NDAVSTRKAPYEDSSGIFWDNDSLSALLAL
ELKADLLVLLSDVEGLYSGPPSDPHSKLIHTYIKEKHQNEITFGDKSRVGRGGMTAKVKAAVHAADAGIP
VVITSGFAAENIINVLQGQRIGTLFHKDAH
EWVQVKEVDAREMAVAARECSRRLQAISSEERKQILLKIADDLEANEKIIRTENEADVAVAQQAGYENSL
VARLALKPGKIASLANNVRIIANMEDPIGQ
VLKRTELSDGLILEKTSSPLGVLLIVFESRPDALVQIASLAIRSGNGLLLKGGKEAKRSNAILHKVITEA
IPDTVGGKLIGLVTSREEIPELLKLDDVID
LVIPRGSNKLVSQIKSSTKIPVLGHADGVCHVYVDKSANVEMARGIVLDAKLDYPAACNAMETLLIHKDL
IEKGWLNDIVVDLRTEGVKLYGGPRASSLL
NIPQAHSFHHEYSSLACTVEIVDDVYAAIEHINLYGSAHTDSIIAEDKEVANVFLRQVDSAAVFHNASTR
FSDGARFGLGAEVGISTSRIHARGPVGVEG
LLTTRWILKGSGQVVDGDKGIVYTHKDLAA
>GmALDH18B5
MENTDPCRHFLKDVKRIIIKVGTAVVTRQDGRLAVGKLGALCEQIKELNSLGYEIILVSSGAVGLGRQRL
RYRKLINSSFADL
QKPQVELDGKACAAVGQNSLMALYDVLFSQLDVTSAQLLVTDNDFRDKDFRMQLSETMKSLLALKVIPIF
NENDAVSTRKAPYEDSSGIFWDNDSLSALL
ALELKADLLILLSDVEGLYSGPPSDPRSKLIHTYIKEKHQSEITFGDKSRVGRGGMTAKVKASIHAAEAG
IPVIITSGYAAENIIKVLQGQRIGTLFHKD
AHKWAPVKEVDAREMAVAARDCSRRLQALSSEERKQILLKIADALEAHQNEIRIENEADVADAKEAGYEK
SLVARLVLKNEKLASLANNIRIIANMEDPI
GRVLKRTELAEGLILEKTSSSLGVLLIVFESRPDALVQIASLAIRSGNGLLLKGGKEAKRSNAILHKVIT
EAIPDIVGSKLIGLVTSRAEIPELLKLDDV
IDLVIPRGSNKLVTQIKSSTKIPVLGHADGICHVYVDKSADLEMARRIVLDAKIDYPAGCNAMETLLVHK
DLVEKGWLNSIIIDLRTEGVTLYGGPKASP
LLNIPMARMLHHEYNSLACTVEIVDDVYAAIDHINLYGSAHTDSVVAEDHEVANVFLRQVDSAAVFHNAS
TRFSDGARFGLGAEVGISTSRIHARGPVGV
DGLLTTRWILKGSGQIVDGDKAVNYTHRDLSI

```
>GmALDH22A1
MAFWWPLLVLAFAYGICRFLLMLIPPKVPSIDVDTSDVLDDGNQAQENSFIYVPPRGTSQQSGKIVQCYE
PATMKYLGYVPAL
THEEVKDRVSKVRKAQKMWAKSSFKQRRLFLRILLKYIIKHQALICEISSRDTGKTMVDASLGEIMTTCE
KINWLLSEGEQWLKPEYRSSGRSMLHKRAK
VEFHPLGVIGAIVSWNYPFHNIFNPMLAAIFSGNGIVIKISEHASWSGCFYFRIIQSALAAIGAPEDLVE
VITGFAETGEALVSSVDKVIFVGSPGVGKM
IMNNASNTLIPVTLELGGKDAFIVCEDVDLDHVAQIAVRAVLQSSGQNCAGAERFYVHREIYSSFVSKVT
KIVKSVTAGPPLVGKYDMGALCMHEHSEKL
EGLVNDALDKGAEIVARGNLGHIGEDAVDQYFPPTVIVNVNHTMRLMQEEAFGPIMPIMKFSSDEEVVRL
ANDSKYGLGCAVFSGNQSRAREIASQIHAG
VAAVNDFASTYMCQSLPFGGVKHSGFGRFGGVEGLRACCLVKAVVEDRWWPFVKTKIPKPIQYPVAENGF
EFQESLVEALYGLGIWDRLRALVNVLKMLT
EQNPGGSSNKRRND
>GmALDH22A2
MAFWWPLLVLALAFAICKFLLILIPPKVPSIDVDASDVLDDGSQAQENSFIYVPPRGTAQQSSGKVQCYE
PATMKYLGYVPAL
TPDEAREQVEKVRKAQKMWAKTSFKKRRQFLRILLKYIIKHQALICEISSRDTGKTMVDASLGEIMTTCE
KINWLLSEGEQCLKPEYRSSGRAMLHKRAK
VEFHPLGVIGAIVSWNYPFHNIFNPMLAAVFSGNGVVIKISEHASWSGCFYFRIIQSALAAIGAPEDLVE
VITGFAETGEALVSSADKVIFVGSPGVGKM
IMSNAAETLIPVTLELGGKDAFIVCEDVDVDLVAQIAVRAALQSSGQNCAGAERFYVHRKIYASFGPPLA
GKYDMGALCMHAHSEMLEALINDALDKGAE
IIARGSFGPIGEDAVDQYFPPTVIVNVNHSMRLMQEEAFGPIMPIMKFSSDEEVVRLANDSKYGLGCNVF
SGSQSRAREIASQIHCGLAAVNDFASTYMC
QSLPFGGVKNSGFGRFGGVEGLRACCLVKSVVEDRWWPFIKTVIPKPIQYPVAENGFEFQESLVEALYGL
SVWDRLQALVNVLKMLTEQNSTSGSRKKKN
D
>GmALDH22A3
MAFWWPLLVLALAFAICKFLLILIPPKVPSIDVDASDVLDDGSLTQENSFIYVPPRGTAQQSSGKVQCYE
PATMKYLGYVPAL
TPDEVKEQVEKVRKAQKMWAKTSFKKRRHFLRILLKYIIKHQALICEISSRDTGKTMVDASLGEIMTTCE
KINWLLSEGEQCLKPEYRSSGRAMLHKRSK
VEFLPLGVIGAIVSWNYPFHNIFNPMLAAVFSGNGIVIKISEHASWSGCFYFRIIQSALAAIGAPEELVE
VITGFAETGEALVASADKVIFVGSPGVGKM
IMSNAAETLIPVTLELGGKDVFIVCEDADVDHVAQVAVRAALQSSGQNCAGAERFYVHRNIYASFVSKVT
KIIKSVTAGPPLAGKYDMGALCMHAHSEKL
EALINDALDKGAEIIARGSFGHIGEDAVDQYFPPTVIVNVNHSMRLMQEEAFGPIMPIMKFSSDEEVVRL
ANDSKYGLGCNVFSGSQSRAREIASQIHCG
LAAVNDFAATYMCQSLPFGGVKNSGFGRFGGVEGLRACCLVKSVVEDRWWPFIKTVIPKPIQYPVAENGF
EFQESLVEALYGLSVWDRLQALVNVLKMLT
EQNSTSGSRKKKND
> GmALDH22A4
MAFWWPLLVLAFAYGICRFLLMLIPPKVPSIDVDTSDVLDDGNQAQENSFIYVPPRGTSQQSGKIVQCYE
PATMKYLGYVPAL
TRDEVKDRVAKVRKAQKMWAKSSFKQRRLFLRILLKYIIKHQALICEISSRDTGKTMVDASLGEIMTTCE
KINWLLSEGEQWLKPEYRSSGRSMLHKRAK
```

VEFHPLGVIGAIVSWNYPFHNIFNPMLAAIFSGNGIVIKISEHASWSGCFYFRIIQSALAAIGAPEDLVE
VITGFAETGEALVSSVDKVIFVGSPGVGKM
IMNNAANTLTPVTLELGGKDAFIVCEDVDLDHVAQIAVRAVLQSSGQNCAGAERFYVHREIYSSFVSLVT
KIVKSVTAGPPLVGKYDMGALCMHEHSEKL
EGLVNDALDKGAEIVARGSFGHIGEDAVDQYFPPTVIVNVNHTMRLMQEEAFGPIMPIMKFSSDEEVVRL
ANESKYGLGCAVFSGNQSRAREIASQIHAG
VAAVNDFASTYMCQSLPFGGVKHSGFGRFGGVEGLRACCLVKAVAEDRWWPFVKTKIPKPIQYPVAENGF
EFQESLVEALYGIGIWDRLRALVNVLKMLT
EQHPGGGGKRRND
>BrALDH2C1
MENGKCNGGATAKLPEIKFTKLFINGQFLDAASGKTFETIDPRNGEVIAK
IAAGDKEDVDLAVNAARHAFDHGPWPRMTGFERARIINKYTDLIQQNIE
ELAALDAVDGGKLFQVGKMNDIPAAAGHFRYYAGAADKIHGETLRMTRP
SLFGYTLKEPIGVVGHIIPWNFPSIMFAMKVAPALAAGCTMVVKPAEQT
PLSALFYAHLSKEAGFPDGVINLVTGFGSTAGAAIASHMDIDKVSFTGS
TDVGRKIMQAAATSNLKKVSLELGGKSPLLIFDDADVNKAAELALLGCF
YNKGEICVASSRVFVQEGIYDKVVAKMVEKVKDWPVGDPFDSTSRQGPQ
VDKKQYEKVLSYIEHGKNEGATLLTGGNAIGDKGYYIEPTIFADVTDDM
KIYKEEIFGPVMSLMKFKTMEEGIKCANNTKYGLAAGIVSQNVDVINTV
SRSIKAGVIWVNCYFAFDLDSPYGGYKMSGNCRESGMDALDSYLQVKSI
AMPLHNSPWM
>BrALDH2B1
MAARSRVSSLLSRSFSASSPFLSRSQGRNLNNGSRIVRRFGTSSAAEEVI
SPSVQVSYTKLLIDGNFVDAASGKTFPTLDPRTGEVIAHVAEGDAEDIN
RAVKAARKAFDEGPWPKMTAYERSRVMLRFADLVEKHSEELAALESWDN
GKTYEQALTAEIPMVARLFRYYAGWADKIHGLTVPADGNYHVQTLHEPI
GVAGQIIPWNFPLLMFAWKVGPALACGNTIVLKTAEQTPLTAFYVGKLF
LEAGLPPGVLNIVSGFGATAGASLASHMDVDKLAFTGSTDTGKVILGLA
ANSNLKPVTLELGGKSPFIVFEDADIDKAVELAHFALFFNQGQCCCAGS
RTYVHEKVYDEFVEKAKARALKRVVGDPFKKGIEQGPQIDSKQFEKVMK
YIRSGVESNATLECGGGQVGDRGYFIQPTVFSNVKDDMLIAQDEIFGPV
QSILKFSDVDEVIKRANDTRYGLAAGVFTKSLDTANRVSRALKAGTVWV
NCFDVFDAAIPFGGYKMSGNGREKGIYSLNNYLQVKAVVTPLNNPAWI
>BrALDH2B2
MAARRVSSLLSRSFSTSSPFPFRSQGRNCYNGSRVVRRFGTSSAAEKIIS
PSVQVSCTQLLIDGNFVDATSGKTFQTLDPRTGEVIADVAEGDAEDIDR
AVKAARKAFDKGPWPRMTAYERSRIMLRFADLVEKHSEELAALETWDNG
KTYQQAKTAEIPMLARLFRYYAGWADKIHGLTVPADGNYHVQTLHEPIG
VAGQIIPWNFPLLMFAWKVGPALACGNTIVLKTAEQTPLTAFYAGKLFL
EAGLPPGVLNIVSGFGPTAGASLASHMDVDKLAFTGSTDTGKVILGLAA
NSNLKPVTLELGGKSPFIVFEDANINKAVELAHFALFFNQGQCCCAGSR
TYVHEKVYDEFVEKAKARALKRVVGDPFKKGIEQGPQIDSKQFEKVMRY
IRSGVESNATLECGGDQIGNKGYFIQPTVFSNVKDDMLIAQDEIFGPVQ
SILKFRDVDEVIRRANETRYGLAAGSLDTANRVSRALKAGTVWVNCFDV
FDAAIPFGGYKMSGNGREKGIYSLNNYLQIKAVVTALNNPAWI
>BrALDH2B3
MASRRVSSLLSRSLISSSSLFSLRGKDPLLNRGARRYSNLAASLEDTITP
PVKVEHTQLLINGKFVDSASGKTFPTLDPRTGEVIAQVAEGDVEDVNRA

VVAARKAFDQGPWPRMTAYERSKILFRFADLIEKHNDEIAALETWDNGK
PYEQSANIEVPMLARVFRYYAGWADKIHGMTVPGDGSHHVQTLHEPIGV
AGQIIPWNFPLLMLSWKLGPALACGNTVVLKTAEQTPLSALLVGRLLHE
AGLPEGVVNIVSGFGPTAGAAIASHMDIDKVAFTGSTDVGKIILELASK
SNLKAVTLELGGKSPFIVCEDADVDQAVEMAHFALFFNQGQCCCAGSRP
FVQERVYDEFVEKAKARAINRAVGDPFKSGIEQGPQVDSEQFEKILKYI
RHGVDSGATLQAGGDRHGSKGYYIQPTVFSDVKDDMLIAKDEIFGPVQT
ILKFKNLDEVIARANNSRYGLAAGVFTQNLDTANRLMRALRVGSVWINC
FDVFDATIPFGGYKMSGIGREKGIYSLNNYLQVKAVVTSIKNPAWL
>BrALDH2B4
MASRRVSSMLSRSFMSSPSLFALRGKHHNMNRGVYGYSNVAAGEDTITPP
VKVEHTQLLIGGKFVDAASGKTFPTLDPRTGEVIAQVAEGDVEDVNRAV
SAARKAFDEGPWPRMTAYERSKILLRFADLVDKHNDEIAAIETWDNGKP
FEQSSKIEVPMLARVFRYYAGWADKIHGMTVPGDGSHHVQTLHEPIGVA
GQIIPWNFPLLMLSWKLGPALACGNTVVLKTAEQTPLSALLVGRLLHEA
GLPEGVVNIVSGFGPTAGAAIASHMDIDKVAFTGSTDVGKIILQLASKS
NLKAVTLELGGKSPFIVCEDADVDQAVELAHFALFFNQGQCCCAGSRTF
VHERVYDEFVEKAKARAIKRAVGDPFKSGIEQGPQVDSEQFKKILKFIK
HGVESGATLQAGGDRFGSKGYYIQPTVFSDVKDDMLIATDEIFGPVQTI
LKFKNLDEVIARANNSRYGLAAGVFTQNLDTANRLMRALRVGSVWINCF
DVFDATIPFGGYKMSGIGREKGIYSLNNYLQVKAVVTAIKNPAWL
>BrALDH3H1
MFKQPLTDLMLSIDAVIGAISAGNTVVLKASELAPASSSLLAKLLEQYLD
PCAVRVVEGAVTETTLLLEQKWNKIFYTCSSRIGRIIMKAAVKHLTPVS
LELGGKSLIVIDSDTNLDVKKYSPFPSGITITVRRIISGKWGCNNGQVC
ISPDYILTTKEYAPKVIDALKQELEAFYGNKSRESKDMSRIVNLNQFDR
LSKMLEEKEVSDKIIYGGQKNRDNLNISPTILLDVPLDSLIMSEEIFGP
LLPILMMYGQDGALVVHKVRHELRHQTRIQGLHESVKEH
>BrALDH3H2
MAKVFEAADASNLMTELRMSFDAGVTRSYEWRVSQLKKLQVICDNHEPEI
VSALHDDLGTPELESSVYEVALLRNSIKLALKQLKNWMAPDKAKTSLTT
FPASAEIVSEPLGVVLVISAWNYPFLLSIDPVIGAISAGNAVVLKPSEL
APASSSLLAKLLEQYLDPSAVRVIEGAVTETTLLLEQKWDKIFYTGSSK
IGRIIMMAAAKHLTPVVLELGGKSPVVIDSDTNLKITVKRIIAGKWGCN
NGQACISPDYILTTKEYAPKVIDAMKQELEAFYGKNPMESKDMSRIVNS
NHFDRLSKILEEKEVSDKIVYGGQKNRDNLKIAPTIFLDVPLDSLIMSE
EIFGPLLPILTLNNLEECFDVIRSRPKPLAAYLFTQNQKLKERFAMTVS
AGGIVVNDIAVHLSLPTLPFGGVGESGMGSYHGKFSFDAFSHKKAVLYK
SFIGDAAIRYPPYSRGKLRLLKALVNSNLVEVFKVLLGLS
>BrALDH3H3
MVKVFQAADATDLVTELRRSFDDGVTRGYEWRVTQLKKLLLICDNHEPEI
VSALHDDLGKPELESSVYEVALLRNSINLAVKQLKDWMAPDKAKTSLTT
FPASAEIVYEPLGVVLVISAWNYPFLLSIDPVIGAISAGNAVVLKPSEL
APASSSLLAKLLEQYLDSSAVRVVEGAVTETTLLLEQKWDKIFYTGSSR
IGRIIMMAAAKHLTPVVLELGGKSPVVIDSDTNLKITAKRIIAGKWGCN
NGQACISPDYILTTKEFSPKVIDALKQELEAFYGKNPMESKDMSRIVNS
NHFDRLSKMLEEKEVSDKIVYGGQKNRDKLKIAPTILVDVPLDSQIMSE
EIFGPLLPIITLNNLEECFDVIRSRPKPLAAYLFTQNQKLKERFALTVS

AGGIVVNDIAVHLAVPTLPFGGVGESGMGSYHGKFSFDAFSHKKAVLYK
SFIGDAAIRYPPYSTGKLRLLKALVNSNILEIFRVILGLS
>BrALDH3I1
MTKLLKINHHHTIPFAGGLYRTTTRSNVASLALATSPFQFSSGYCSKTCI
PSRLKLVSSTCYATLSAVVKPQESAFDGKEAALLVDELRTNFNTGRTRS
YEWRISQLQNIAKMIDEKEKCITEALYQDLSKPELEAFLAELSNTKSSC
MLAIKELKNWMAPETVKTSVTTFPSSAQIVSEPLGVVLVISAWNFPFLL
SVEPVIGAISAGNAVVLKPSEIAPATSSLLAKLFSEYLDETAIRVVEGG
VPETTALLDQKWDKIFFTGGARVGRIVMAAAAKNLTPVVLELGGKCPAL
VDSDVNLQVAARRIITGKWACNNGQACIGVDYVITTKDFAPKLIDALKT
ELKTFFGENPLKSKDVSRIVNSFHFKRLESMMKENGVANKIVHGGQTME
DKLKISPTILVDVPEESSMMQEEIFGPLLPVITVSKIEDGFQVIRSKPK
PLAAYLFTDNKVLQNRFVENVSAGGMGINETVLHVTLKDLPFGGVGESG
IGAYHGKFSYETFSHKKGVLYRSFDGDSDLRYPPYTPEKKRVLKALLSS
DIFGAILAFFGFSKDS
>BrALDH3F1
MEEVVDQSLREMRDTFASGRTRSVKWRKTQLEAIIEMVKDNEDKMCDVLF
QDLGKHSTEAFRDELGFVMRSATTALNCLDKWVVPKKSNLPLLFYPSTG
KVISEPYGTVLVLSSWNFPISLSLDPLIGAISAGNTVLLKASELSPNAS
AFLAKTIPSYLDNKAIKVIEGGPDVATILLKHQWDKIFFTGSPRIGKII
MAAAAEHLTPVTLELGGKCPTIVDHHSVSKDMKSVVKRISGGKWGSCSG
QACISVDYVLVEKSFASSLIEMLKPMIRSFFGENPKESGCLSRIVNKKH
FQRLARLLNDPGVQASIVYGGSMDEEKLYIEPTILLDPPLDSEIMNEEI
FGPILPIITLRDIQESIGFIKSKPKPLAIYAFTKDENLKTRILSETSSG
SVTFNDVMIQYMCDALPFGGVGESGIGRYHGKYSFECFSHEKAIMEGSL
AMDLEARYPPWNSFKLTFLRLAFREAYFKLVLFMLGLKK
>BrALDH3F2
MEAMKETVDQSLREMRDTFASGRTRSVKWRKTQLEAIIEMVKDNEDKMCD
VLFQDLGKHSIEAFRDELGFVMRSATTALNSLDKWVVPRKSNLPLLFYP
ATGKVISEPYGTVLVLSSWNFPISLSLDPMIGAISAGNTVLLKASELSP
NASALLAKTIPSYLDNKAIKVIEGGPDVATILLQHQWDKIFFTGSPRIG
KIIMAAAAENLTPVTLELGGKCPTIIDHHSVSKDMKSVVKRISGGKWGS
CSGQACISVDYVLVEQSFASSLIEMFKPVIKSFFGENPKESGCVARIVT
KKHFQRLSRLLNDPRVQASIVYGGSMDEEKLYVEPTILLNPPLDSEIMN
EEIFGPVLPIITLRDIQESIGFIKSKPKPLAIYAFTKDEKLKTRILSET
SSGSVTFNDVMIQYMCDALPFGGVGESGMGRYHGKYSFECFSHEKAIME
GSLAMDLEARYPPWNNFKLTFIRLAFREAYFKLVLLMLGLKR
>BrALDH3F3
MEAVKETVDQSLREMRDTFASGRTRSVKWRKAQLGAIIEMVKDNEEKMSD
VLFQDLGKHSTEAFRDELGFVMRSATTALNCLDKWVVPRNSNLPLLFYP
ATGKVISEPYGTVLVLSSWNFPISLSLDPMIGAIAAGNTVLLKASELSP
NASALLAKLIPSYLDTKAIKVIEGGPDIATILLQHQWDKIFFTGSPKIG
KIIMAAAAEHLTPVTLELGGKCPTIIDHHSVSKDMKAYYVLVEQSFAST
LIDMFKPVIRSFFGENPKESGCLAKIVTKKHFQRLSRLLNDPRVKASIV
YGGSMDEEKLYVEPTILLDPPLDSEIVNEEIFGPILPIITLRDIQESIG
FIKSKPKPLAIYAFTKDENLKTRILSETSSGSVTFNDLMIQYMCDALPF
GGVGQSGMGRYHGKYSFECFSHEKAIMEGSLAMDPEARYPPWNNFKLNF
IRLAFREAYFKLVLLMLGLTKGVRK

>BrALDH5F1
MVLGAAARVAIVGCRRLVCSSSHASPLLVSSQCRQMSMDAQSVSEKLRSS
GLLRTQGLIGGKWIDSYDKTTIKVNNPATGEIVADVACMGVKETNDAIA
SSYEAFQSWSRRTAGERSRVLRRWFDLLVAHKEELGQLITLEQGKPLKE
AIGEVAYGASFIEYYAEEAKRVYGDIIPPNASDRRLLVLKQPVGVVGAI
TPWNFPLAMITRKVGPALASGCTVVVKPSELTPLTALAAAELALQAGVP
PGALNVVMGNAPEIGDALLASPQVRKITFTGSTAVGKKLMAAAAPTVKK
VSLELGGNAPSIIFDDADLDVAVKGTLAAKFRNSGQTCVCANRVLVQDG
IYDKFAEAFSEAVQKLEVGDGFKEGTTQGPLINDAAIQKVESFVQDAVS
KGAKILLGGKKHSLGMTFYEPTVIRDVTSNMIMSKEEIFGPVAPLIRFK
TEEDAIRIANDTIAGLAAYIFTNSVQRSWRVSEALEYGLVGVNEGIIST
EVAPFGGVKQSGLGREGSKYGMDEYHEIKYICMGDMNRQ
>BrALDH6B1
MLLRTSLQQGKTNLRPRFLRCLLSTMSSTQPPRVPNLIGGSFVDSQASSH
IDVINPATQEVVSQVPLTTNEEFKAAVSSAKKAFPSWRNTPITTRQRVM
LKFQELIRKNMDKLALSITTEQGKTLKDAHGDIFRGLEVVEHACGMATL
QMGEYVPNVSNGVDTYSLREPLGVCAGICPFNFPAMIPLWMFPIAVTCG
NTFVLKPSEKDPGASVMLAELAMEAGLPDGVLNIVHGTNDTVNAICDDD
DIRAVSFVGSNTAGMHIYARAAAKGKRIQSNMGAKNHGVVLPDANVDAT
LNALLAAGFGAAGQRCMALSTVVFVGNSKSWEDKLVERAKALKVSCGTE
PDADLGPVISIQAKERICRLIQSGVDDGAKLLLDGRNIVVPGYEKGNFI
GPTILSGVTPDMECYKEEIFGPVLVCMEASSFDEAIDILNRNKYGNGAA
IFTASGAAARKFQMEIEAGQIGINVPIPVPLPFFSFTGNKASFAGDLNF
YGKAGVDFFTQIKTVTQQWKDIPTSVSLAMPTSQKQ
>BrALDH7B1
MVQQVSGRSGKTLLELSGNNAIIVMDDADIHLLHVSVYDKVLEQLLTSYK
QVKIGDPLQKGTLLGPLHTPESRKNFKKGIEVIKSQASTSSPKSSLNIF
NFQLTLLHSQGGKVLTGGKAIEGEGNFVEPTILEISSDAVLVKEELFAP
VLYALSHLKKQVAINNSIPQGLSSSIFTRKPKNIFKWIGPMGSDCGIVN
VNIPTNGAEIGGDFGGEKATGGGRETGSDSWKQ
>BrALDH7B2
MGSASKEYEFLSEIGLSSSHNLGNYVGGKWLGNGPLVSTLNPANNQPIAQ
VVEASLEDYEIGLKACEEAAKTWMQVPAPKRGDIVRQIGDALRSKLDYL
GRLLSLEMGKILAEGIGEVQEVIDMCDFAVGLSRQLNGSVIPSERPNHM
MLEMWNPLGIVGVITAFNFPCAVLGWNACIALVCGNCVVWKGAPTTPLI
TIAMTKLVAEVLEKNHLPGAIFTAMCGGAEIGEAIAKDTRIPLVSFTGS
SKVGLTVQQTVSARSGKTLLELSGNNAIIVMDDADIQLAARSVLFAAVG
TAGQRCTTCRRLLLHESVYDKVLEQLLTSYKQVKIGDPLEKGTLLGPLH
TPESKKNFEKGIEGGKVLTGGKAVEGEGNFVEPTIIEISSDAAVVKEEL
FAPVLYALKFKTFEEAVAINNSVPQGLSSSIFTRSPENIFKWIGPMGSD
CGIVNVNIPTNGAEIGGAFGGEKATGGGREAGSDSWKQYMRRSTCTINY
GNELPLAQGINFG
>BrALDH10A1
MAIRVPRRQLFIGGQWTEPIRRQTLPVVNPATEDIIGYIPAATSEDVELA
VEAARKALTRNQGKDWSKASGAVRARYLRAIAAKVTERKSELANLEAID
CGKPLDEAAWDMDDVAGCFEYYADLAQGLDAKQKAPLSLPLDTFKGYVL
KEPIGVVGLITPWNYPLLMAVWKVAPALAAGCTAILKPSELASVTCLEL
ADICREVGLPPGVLNILTGLGTEAGAPLASHPHVDKIVFTGSTATGSNI

MTSAAKLVKPVSLELGGKSPIIVFDDVEIDKAVEWTMFGCFWTNGQICS
ATSRLLVHEKIADEFLDKLVKWTKNIKISDPFEEGCRLGPVVSKGQYER
VVKFVSNARKEGATVLCGGARPGHLKKGYFVEPAIISNVTTSMEIWRDE
VFGPVLCVKTFSTEDEAIQLANDSQYGLAGAVLSNDLERCDRVSKAFEA
GIVWVNCSQPCFCQAPWGGTKRSGFGRELGEWGLENYLSVKQVTQYISN
EPWGWYKPPSKL
>BrALDH10A2
MAIPMPTRQLFIDGEWRAPILKNRIPIVNPATQDVIGDIPAATKEDVEVA
VNAARRAFSRNKGKDWAKAPGALRAKYLRAIAAKVTERKSHLATLESLD
SGKPLDETVWDMEDVAGCFEFYADLAEGLDAKQKAPVSLPMETFKSYVL
KQPIGVVGLITPWNYPLLMAVWKVAPSLAAGCTAVLKPSELASVTCLEL
ADICREVGLPPGVLNVLTGYGSEAGAPLASHPSVDKIAFTGSFATGSKV
MTAAAQLVKPVSMELGGKSPLIVFDDVDLDKAAEWALFGCFWTNGQICS
ATSRLLVHENIASEFIEKLVKWSKNIKVSDPLEEGCRLGPVVSEGQYEK
ILKFISTAKSEGATILHGGSRPEHLKKGFFIEPTIITDVTTSMQIWREE
VFGPVLCVKTFSSEDEAIELANDSHYGLGAAVISNDAERCDRVSQDFEA
GIVWINCSQPCFTQAPWGGVKRSGFGRELGEWGLDNYLSVKQVTLYTSN
DPWGWYKPPC
>BrALDH11A1
MAGTGIFTDILDGDVYKYYSDGEWKTSSSGKSVAIINPATRKTQYKVQAC
TQEEVNKVMEMAKSAQKSWAKTPLWKRAELLHKAAAILKDNKAPIAESL
VKEIAKPAKDSVTEVVRSGDLISYCAEEGVRILGEGKFLLSDSFPGNER
TKYCLTSKIPLGVVLAIPPFNYPVNLAVSKIAPALIAGNSLVLKPPTQG
AVSCLHMVHCFHLAGFPKGLISCITGKGSEIGDFLTMHPAVNCISFTGG
DTGISISKKAGMIPLQMELGGKDACIVLEDADLDLVASNIIKGGFSYSG
QRCTAVKVVLVIKSVADELVEKVKAKVAKLTVGPPEENCDITAVVSESS
ANFIEGLVMDAKDKGATFCQEYKREGNLIWPLLLDNVRPDMRIAWEEPF
GPVLPVLRINSVEEGINHCNASNFGLQGCVFTKDINKAILISDAMETGT
VQINSAPARGPDHFPFQGLKDSGIGSQGVTNSINLMTKLKTTVINLPTP
SYSMG
>BrALDH11A2
MAGTGIFTEILDGEVYKYYSDGEWRTSSSGKSVAIVNPATRKTQYKVQAC
TQEEVNKVMEMAKSAQKSWAKTPLWKRAELLHKAAAILKDNKAPIAESL
VKEIAKPAKDSVTEVVRSGDLISYCAEEGVRILGEGKFLLSDSFPGNER
TKYCLTSKIPLGVVLAIPPFNYPVNLAVSKIAPALIAGNSLVLKPPTQG
AVSCLHMVHCFHLAGFPKGLISCITGKGSEIGDFLTMHPAVNCISFTGG
DTGISISKKAGMIPLQMELGGKDACIVLEDADLDLVASNIIKGGFSYSG
QRCTAVKVVLVMESVADELVEKVKAKVAKLTVGPPEENCDITAVVSESS
ANFIEGLVMDAKEKGATFCQEYKREGNLIWPLLLDNVRPDMRIAWEEPF
GPVLPVLRISSVEEGINHCNASNFGLQGCVFTKDINKAMLISDAMETGT
VQINSAPARGPDHFPFQGLKDSGIGSQGVTNSINLMTKVKTTVINLPTP
SYSMG
>BrALDH12A1
MYRVLASRGLRAKSLCDNKASSFLASFTSSRLNHSIPFATVDAEEISGAR
PAEVQSFVQGKWIGSSNYNTLLDPLNGEPFIKVAEVEESGVQPFIESLA
Q CPKHGLHNPFKSPERYLLYGDISTKAAHMLALPKVSDFFTRLIQRVAP
KS YQQAAGEVFVTRKFLENFCGDQVRFLARSFAVPGNHLGQQSHGYRWP
YGP VTIVTPFNFPLEIPLLQLMGALYMGNKPLLKVDSKVSIVMEQMMRL

LHYC GLPVEDVDFINSDGKTMNKILLEANPRMTLFTGSSRVAEKLALDL
KGRIR LEDAGFDWKVLGPDVQEVDYVAWVCDQDAYACSGQKCSAQSMLF
VHENWS KTPLLSKLKDLAGRRKLEDLTIGPVLTFTTEAMVEHMENLLQI
PGSKLLF GGKPLKNHSIPSIYGALEPTAVYVPIEEILKDSKTYELVTKE
IFGPFQIV TEYKKDQLPLVLDALERMHAHLTAAEVIGNSVNGTTYAGLR
GRTTGAPQN HWFGPAGDPRGAGIGTPEAIKLVWSCHREVIYDYGPIPQG
WELPPST
>BrALDH12A2
MSHDEKRLISSLLDLNNVQIFDEVISGAHPAEVQSFVQGKWIGSSNYNTL
LDPLNGEPFIKVSEVDESGVQPFVESLSQCPKHGLHIPFKSPERYFKSP
ERYLLYCDISTKAAHMLALPKVSDFFTRLIQRVAPKSYQQAAGEVFVTR
KFLDNFCGDQVMQCSKKSV
>BrALDH22A1
MAFWWPLIVLAFAYAICRFLLMLIPPNVPSIDVDASDVLAHGKETEENSF
IYIPPRGRSQQSDKKVQCYEPATMKYLGYFPALSTSEVKERVALSRKAQ
KTWAQSSFKVRRQFLRILLKYIIEHQELICEVSSRDTGKTMVDASLGEI
MTTCEKITWLLSEGERWLKPEYRSSGRAMLHKVSRVEFHPLGVIGAIVP
WNYPFHNIFNPMLAAVFSGNGIVIKVSEHASWSGCFYFRIIQAALAAVG
APENLVDVITGFAETGEALVSSVDKMIFVGSTAVGKMIMRNAAETLTPV
TLELGGKDAFIICEDADVSHVAQVAVRGTLQSSGQNCAGAERFYVHKDI
YTAFITQVTKIVKSVSAGPPLTGRYDMGAICLQEHSEHLQSLVNDALDK
GAEIAVRGSFGHLGEDAVDQYFPPTVLINVNHTMKIMKEEAFGPIMPIM
QFSTDEEVIKLANDSRYALGCAVFSGSQRRAKQIASQIQCGVAAINDFA
SNYMCQSLPFGGVKDSGFGRFAGIEGLRACCLVKSVVEDRFWPLIKTKI
PKPIQAKNAFEFQEALVETLYGLNIWDRLRSLIDVLKFLTDQSSHVSRT
RKSH
>VvALDH2B4
MAARRISSLLSRSLSVSSAFSLSLGKNFNRGKSIHRFSTAAAAAVEELITPTVQINYTQLLINGQFVDAA
SGKTFPTFDPRTG
EVIANVAEGDAEDINRAVSAARKAFDEGPWPRMSPYERSRILLRFADLAEKHNDELAALETWNNGKPYEQ
AAKAELPLFVRLFRYYAGWADKIHGLTVQA
DGPHHVQILHEPIGVAGQIIPWNFPLMMFAWKVGPALACGNTIVLKTAEQTPLTALFAAKLFHEAGLPPG
ILNIVSGYGPTAGAALASHMDVDKIAFTGS
TDTGKIVQELASKSNLKPVTLELGGKSPFIVCEDADIDQAVELAHFALFFNQGQCCCAGSRTFVHESVYD
EFIEKAKARALSRTVGDPFKKGIEQGPQID
PEQFAKVLRYIRSGIESNATLECGGGRIGSKGYFVQPTVFSNVQDDMLIAKDEIFGPVQSILKYKDLDEV
IRRANSTRYGLAAGVFTKNINTANTLTRAL
RVGTVWVNCFDVFDAAIPFGGYKMSGVGREKGIYSLNNYLQVKAVITPLKNPAWL
>VvALDH2B8
MTIPRISSLLSRSFTSSASSALLSSIGRNSSRRGGIFRYSTAAVVEEPINPSVNVNYTQLLINGQFVDAA
TGKTFETLDPRTG
NVIASVAEGDAEDVNRAVSAARKAFDEGPWPRMSPYERSKILLRFADLLEKHNDEIAALETWDNGKPFEQ
AAKAEVPLVIRLMRYYAGWADKIHGLTVPA
DGLHQVQTLHEPIGVAGQIIPWNFPLLMYAWKIGPALACGNTIVLKTAEQTPLSALYASKLLHEAGLPPG
VLNVVSGYGPTAGAALASHMDVDKLAFTGS
TATGKIVLQLAARSNLKPVTLELGGKSPFIVCEDANVDEAVELAHFALFFNQGQCCCAGSRTFVHESIYD
EFVEKAKARALRRTVGDPFKAGIEQGPQID

SDQFEKILRYIRSGVENGATLETGGERFGKEGFFIKPTVFSNVQDGMLIAQDEIFGPVQSILKFKDLGEV
IRRANATSYGLAAGVFTQNLDTANTLTRAL
KVGTVWINCFDVFDAAIPFGGYKMSGHGREKGIYSLQNYLQVKAVITPLKNPAWL
>VvALDH2B9
MAAPRIFSLLSASSTAASLRSFGRYSRWGRGSSRFSTAVATEEEPITPPVQIDYTQLLINGRFVDAASGK
TFPTLDPRTGDVI
AHVAEGEAEDINRAVSAARKAFDEGPWPKMTPYERSCILFRFADLLEKHCSEIAALESWDNGKPYEQAAN
VEIPMVIRVFRYYAGWADKIHGLTVPADGL
HHVQTLHEPIGVAGQIIPWNFPLLLYGWKVGPALACGNTIVLKTAEQTPLSALYASKLLYEAGLPPGVLN
VVSGFGPTAGAALSSHMDVDKLAFTGSTGT
GKIVLGLAAKSNLKPVTLELGGKSPFIVCEDADVDKAVELSHTALFYNQGQSCCSGSRTFVHESIYDEFI
EKAKARALKRVVGDPFKKGVEQGPQIDSQQ
FNKILGYIKSGIEAGATLEAGGEKFSSKGYYIQPTVFSNVHDNMLIAKEEIFGPVQSILKFKDLDEVIRR
ANATHYGLAAGIFTQNLDTANTLTRALRVG
TVWINCFFVFDAAIPFGGRKMSGHGREKGIYGLSNYMQVKAVVTPLKNPAWL
>VvALDH3F1
MTGSGVNGMIGMEGGRVEESIGELRRTFRSGETRSAAWRKAQLKALLQLLRDNENKIFEALKQDLGKHPV
ESYRDELGVVEKS
VKYSLSHVDEWMAPKKSSLPLIFFPGKGQVLPEPLGLVLIFSSWNFPISLALDPVIGAISAGNSVVLKPS
EQAPACSSFLANTIPLYLDSKAIKVIEGGA
AISQQLLQQKWDKIFFTGSPSVARIVMSAAVKHLTPVTIELGGKCPTIFDNLSSPSDTEVAVKRVVGGKW
GPCNGQACIGVDYVLVEEKFASHLIEMLKK
TIKKFYGENPKELKDISKIVNKHHFQRLHNLLKEPLVAASIVHGGLIDEEKLFIEPTILLDPPLDAEIMT
EEIFGPLLPIITLKNIEESIEFINSRPKPL
ALYAFTNDEAFKRRILSETSSGSVTFNDIIIQFVCDTLPFGGVGQSGFGRYHGKYSFDTFSHEKAVLRRS
FFLELEPRFPPWNDFKLKFIRLVYSFDYLG
LILLLLGLKR
>VvALDH3H1
MGVTPIKLNFCIFFVNIIIIIKYKILLVCLIFQREGKRLLIDLIVVFPRSSISKSCCGRKSSMAEDSETK
KVFDAEAAASLMK
ELRGTYASGKTRSYEWRVAQLKNLMKIVDDHEKDILDAIRADLSKPEQESYIAEISIIKSSCTRALGELN
RWMKPEKVKTSITTFPSSAEIVSEPLGVVL
IIGAWNFPLLLALDPAIGAIAAGNAVVLKPSELCPATSSLIAKLVGKYLDSSCIKVVEGAVAETSALLEQ
KWDKIFFTGSGRVGRIVMAAAAKHLTPVAL
ELGGKCPVVVDSDINLPVAIRRIAGGKWASNNGQACIAPDYIVTTKDFAPKLIDALKHELEAWYGKDPLE
SKDLAHIVNSNHFARLAKLLDDDKVSGKII
HGGQRDKANLKFAPTILLDVPEDSLVMNEEIFGPLLPILTVDKLEDSFDMITSRGKPLAAYLFTNNKKLK
EKFVKTVSAGGLVINDTVLHFAEKTLPFGG
VGESGMGSYHGKFSYEAFSHRKSVLYRGFAGDASARYPPYSDRKLKLLKALLSGSVVGVILALIGWS
>VvALDH3H5
MRSLSPELFHVCSVDGGYTRSLRNQFPKQRNPPLPIPVLKKRKRLRFTHSSFICSATLAVMADEKKKVFD
VESAASLVKELRG
SFNAGKTKSYEWRIAQLKGIEKMIDEREKDIIEALHEDLSKPELEAFVSEISMSKGACKLALKELGHWMK
PEKAKTSMTTYPSSAEIVSEPLGVVLVIST
WNYPLLLSIDPVIGAIAAGNAVVLKPSEIAPATSTLLSKLLEEYLDNSSIRVVEGAVAETTALLEQKWDK
IFYTGSPRVGRIVMAAAAKHLTPVTLELGG
KCPVVVDSNVNLQVAARRLIAGKWACNNGQACISPDYIITTKDFAPKLIDVLRHELEEFFGKNPIESEDM
SRIVSVQHFKRLTRLLDEDEVSDKIIIGGQ

SDENQLKIAPTILVDVPEDTEIMKEEIFGPLLPILTVENLEESFDVINSKSKPLAAYLFSENKQLQKDFV
NNISAGGMLINDTILHLTVSSLPFGGVGES
GMGSYHGKFSFDAFSHKKAVLYRGFTGESPARYPPYTPGKLKLLKTLTSGNIVSILLALLGFSKD
>VvALDH3J1
MAEIENLEPDLEELRESYRSGKTKEASWRKSQLKGLLTLLKEQEKDIFKALEQDLGKHYAESYRDEVGTL
TKSVNLALSSLKD
WMSSRKAKLPIATFPSTAEVFPEPLGLVLIISSWNFPFGLSLEPVIGAIAAGNSVVLKPSELAPASSSLL
AKTIPTYLDKKAVKVIEGGAAVGEHLLRCK
WDKIFFTGNPRVGRVVMTAAANHLTPVTLELGGKCPAIFDSFSSSWDKEMVIKRVLGGKFGACAGQACIA
IDYILVQEGFAPTLLELLRNMTKKMFGENP
RETKSMARIINKKHFLRLKNILDDPSVQSCIVHGGGVDEDNLFIEPTILMNPPLKASIMTDEIFGPLLPI
ITLKKIEDSIEFINSRPKALAIYVFTKNET
LKRRIISETSSGSVTFNDAIIQYAADTIPFGGVGESGFGRYHGKFSFDTFTHEKAILRRSLLTEFWFRFP
PWNDFKLALTKSAYRFDYFEFLLVLLGLKK
NS
>VvALDH5F1
MGLLRSSCSALCHGPRTASVLRPSAVLTRQISMDTQNLVARLNSSGLLRSQCLIGGKWTEAYDGKTIPVH
NPATGDVLVNVPC
MGGQETNDAISVAYEAFLSWSKLTAAERSKRLRKWYDLLIANKEELGQIITLEQGKPLKEAIGEVNYGAA
FIEFSAEEAKRIYGDIIPSPLADRRLLVLK
QPVGVVGAITPWNFPLAMITRKVGPALACGCTVVIKPSELTPLTALAAAELALQAGIPPGAVNVVFGNAP
EIGDALLASRQVRKITFTGSTAVGKKLMAG
AAQTVKKVSLELGGNAPCIIFDDADLEVAVKGALGTKFRNSGQTCVCANRILVQEGIYEKFAIAFSQAVQ
SMQVGEGFTEGVVQGPLINEAAVQKVESFV
KDAVSKGAKVLLGGKRHSLGMTFYEPTVIGDIKNDMLIARNEVFGPVAPLLRFKTEEEAIRIANDTNAGL
AAYVFTENVQRMWRVTEALEYGLVGVNEGL
VSTEVAPFGGVKESGLGREGSKYGMDEFLEMKYVCFGNISSN
>VvALDH5F2
MEAQSVITRFQNSGVFRTQGLIGGKWTEAYDGKTIQVHNPATGEVIADVPCMGQPETNDAISSAYEMFNS
WSKVTAIERSQCL
WKWHDLLIAHKEELGQLITLEQGKPLNEAIIEVIIAAGYLEFFAEEAKHVYSDIIPSTVADCQLFVIKQP
VGVVGAITPWNFPLAMLTSKVGPALACGCT
VVLEPSELTPLIAFAAAGLALEAGIPSGALNVVTGNAPDIEHALLASPKVRKITFTGLSAVEKKIMPGAG
ETLRKVSLEPGGNAPCIVFDDTDLGVAVKS
ILAVKFHNSGQTCISANRILVQEGIYEKFATAFSKAVTSLQVGDGFCEGVTQGPLINEAAVQTVESLVQD
AISKGAKLLLGGKRHNLGMTFYEPTVIGDV
NNKMLISRNKICGPIAALLRFKTEEEAICIANDTDEGLAAYIFTKNLQRSWRVSEVLEYGLVGVSEGLIP
TVMAPVSGFKNTGLGQEGSKKGMLEYLELK
YICLGNMNNI
>VvALDH5F3
MAKFLFDYYFQYNFQNRPTHLASWHYNSVMTYGSQLLFIVSEGFSRFGFHNLLLICHFYRQNFLIQISMD
PQSAIAQVKRAGL
FRTQGLIGGKWMDAYDGKTFEVYNPATGEVLANVACMGKKEANDAIASAHAAFTSWSKLTTAERSKLMRK
WYDLLIAHQTELGQLITLEQGKPVAEGYNE
VLIGASCLEFFLEEVKHCYGDIIPQTQADRRMFVLKQPVGVVGAITPWNLPLAMALRKVSPAMACGCTVV
IKPSELTPLSALAVAELALQAGIPPGVFNM
VMGFAPEIGDAFLASPKVRAITFTGSTAVGKMLLAGAAQTVKKTSMELGGNAPSMIFDDADLEVTVKGLM
IVKFFNCGQTCISPNRILVQEGIHDKFAAA

LTKAVQTLRVGHGFDEGVTQGPLINQAALHKIEALVEDAVSQGAKVLVGGKRHSLGLTFYEPTVLVDVTS
DMLISSTEIFGPVLALQRFKTEEEAIHLAN
DSNAGLAGYIYTENLRRCWRVAEAIEFGIVGVNDGLIPAASAPFGGFKQSGLGREGSKYGLDDFLEIKYL
CLGNMV
>VvALDH6B3
MLRFSLQRVRNFKSLRPELFALGNSRFSTATEPSSKQRNPPRVPNLIGGSFVDSQSSEFIDVINPATQQV
VSQIPLTSNEEFK
AAVSAAKQAFPAWRNTPVTTRQRIMFKLQQLIRRDIDKLAMNITTEQGKTLKDAHGDVFRGLEVVEHACG
MATLQMGEFVPNVSSGIDTYSIREPLGVCA
GICPFNFPAMIPLWMFPIAVTCGNTFVLKPSEKDPGASLMLAELAMEAGLPDGVLNIVHGTRNIVNAICD
DEDIRAVSFVGSNTAGMHIYSRASAKGKRV
QSNMGAKNHAIVLPDASLDATLNALVAAGFGAAGQRCMALSTVVFVGDSKSWEDKLLERAKALKVNSGTE
PDADLGPVISKEAKERICKLIQNGVDSGAR
LVLDGRNIVVPGYEHGNFIGPTILSDVTADMECYKEEIFGPVLLCIQADSLDEAINIVNRNKYGNGASIF
TSSGVDARKFQTEIEAGQVGINVPIPVPLP
FFSFTGSKASFAGDLNFYGKAGVQFYTQIKTVTQQWKGLASGSGVSLAMPTSQKSQ
>VvALDH6B5
MDIQDCTELNEIPQMLPPPPGSFIDREELIQHVGDFALSQGYVVTIKQSKKDKVVVLGCDRGGVYRNRRK
LVDESSAEQVRKR
KTGSRLTNCPFEVVGKKEDGLWVLAIKNGEHNHDPIRDISEHPSSRRFTEREVLLIKDMTEAGLKPRQIL
KRLRQNNPELLSTPKHVYNVKAKLRQGNLT
VRNFKSLRVKSSVENSHISTANEPSWRQRNPPRVPNLIGGRFVDSQSFASIDVTNPATQKVVSQVPLTTN
EEFRAAIFAAKRAFPSWRDTPVTTRQRIMF
KFQELIRRDIDKIAMNITTEHGKTLKDAYTDVHRGLEVVEHACGMATLQMGEFVSNVSNGIDTYSIREPL
GVCAGICPFDFPAMIPLWMFPIAVTCGNTF
ILKPSEKDPGATIIILAELAMEAGLPNGVLNIVHGTVDIINAICDDDDIKAISFVGSNTDGMYIYARASAK
GKRVQSNIGAKNHAIVMPDASKDATLNALV
SAGFGAAGQRCMVLSTVVFVGGSKSWEDKLVECAKALKVNAGIEPDADLGPVISKQVKERICRLIQAGVD
SGARLVLDGRNIEACLTTYLSMIILLDFFW
SNQSFSYIP
>VvALDH6B7
MTDAGIKPRQVLKALKKNNPELQSTPRHLYNLKAKIRQGNISEKSFKSWRPNRSVPVNTTNPLESSSKHN
IHPLKVPNLIGGK
FVDSQACAIIDVINPATQEVVSEVPLTTYEEFKAAVSAAKQAYPSWRNTPVTTRQRIMFKLQELIRRDID
KLAMNITIEQGKTLKGAQGDVLRGLEVVEH
ACGMATLQMGEFVPNASNGIDTYCLREPLGVCAGICPFNFPAMISLWMFPIAVTCGNTFILKPSEKNPGY
CPYTFGKSSENFKRYIFFWTNKIMLLHESV
NWDATHCGAFIYLWNDLKRVKEDQGKENLWQSCLGFLSKHHLGEDRETFQRSLIATKKFLNKLLREIGEN
GSKPPERPQVENSKKGFLKQKDSILQKSKS
SLNGHLLGLFIGILVGSENCWERKKEIRSNLHVSCSVVMGDVKGELFENLYIFKLLIVFLPLLFEKVNKL
ESRKKRRDQTCFFIVFLLIFLHFLFSFPYD
LSLLFLRYKQGFRASMILAALAMEAGLPHGVLNIVHGTNDIVNYICDDDDIKAVSFVGSNTAGMNIYARA
AARGKRVQSNMGAKNHAIIMPDASMEATLN
ALVAAGFGAAGQRCMALSTAVFVGGSIPWEEELVACAKALKVNAGTEPGADLGPVISKEAKDRICRLVQN
DVGSGARLVLDGRNIVWSDKERSTLVLMMF
PVSTLVNCPRSERYSLSECQVVNSHTLPKLRKASSKSHFWLGRLSESLCLRKHLGLWVIFIFLLFDSNVV
AHCLILQACILSCFKVVGMDIYLKRKMHVP

GYEYGNFVGPTILCDVTTNMECYKEEIFGPVLLCMKADSLEEAITIVNRNKCSNGASIFTTSGVAARKFQ
NEVEAGLVGINVPVPVPLPFSSFTGSKLSF
AGDLNFCGKAGVQFYTQIKTVAQQWKDLPSRGHLRGCICLKNLIGMTLCSPHLKELKLLHQLLRGFMHLQ
RPLRGGRRKEEEERRMQCSNTCWASSVHPP
GRFFTQSSHHNEDTSCPVVTLPLPSHVKSITSPSNPFVKHCFKLRHSSSYRYSHGSALVVGTTPIREIYK
FQQSTQERTVEMDCLLILDKAEIPEGLDDF
SVRLVRVSSMVMKKLSGLQSTESVEAIALMRIPTSFFSVNDDTYEKDCRRWFQSPHRILVLDRIQDPGNL
GTLLRSAMAFRWGGVFLLSGCCDPFNGKAL
RASRGASFQLPIVSGSWIHLETLKNEFQMKMIAGHPDSNQKRKPVSPLSQGLADSLADVPLCLVLGSEGS
GLSEKSWQLCELVSIPMAGEFESLNVSVAG
GIFLYMLQPQNRRVGIGTEIGWL
>VvALDH7B5
MSFERKEYQFLAEIGVGPANPGCYINGEWKARGPLVSSVNPSNNQRIAVVTEASIEDYEEGLMACSEAAK
TWMKIPAPKRGEI
VRQIGEALRAKLGSLGRLVSLEMGKILAEGIGEVQEIVDMCDYAVGLSRQINGSIIPSERPDHMMCEVWN
PMGIVGVITAFNFPCAVLGWNACLALVCGN
CVVWKGAPTTPLVTIAVTKLVAEVLEKNNLPGAIFTSFCGGAEIGEAISKDARIPLVSFTGSSKVGLMVQ
QAVNQRFGKCLLELSGNNAIIIMEDADIGL
AVRSVLFAAVGTAGQRCTTCRRLLVHESIYETVLNQLIDVYKQVKMGDPLEKSTLVGPLHTRASKENFEK
GIEIIKFQGGNILIGGSTVESEGNFVQPTI
VEISPNASVVKEELFGPVLYVMKFQTFEEAVEMNNSVPQGLSSSIFTRKPEVIFKWIGPHGSDCGIVNVN
IPTNGAEIGGAFGGEKATGGGREAGSDSWK
QYMRRSTCTINYGNELPLAQGINFG
>VvALDH7D1
MIVECADMFARSEYMFLSEIDLSVVHPGYYVNGKWKGRSSSMVTSVNPVDNETIAAVTEGSIEDYEEGIQ
ACSKAAKLWMKTP
VSKRCEIVRQIGDALRAKLQLFGRLVSLEVGKILVAGIGEVQEVIDMCDYAAGLSEKLNLNASIRHERQN
HVTLQLRNPFGVVGVITPFNFPCAVLGRNA
CMALVTGNCVVWKGSRTTPLVTIAITKLVAGVLKNNNLPGAIFTSFCGGAPIGQAMAEDKRIPLVSFTGT
SKVGLMVQQRVNDRFGKCLLELSGNNAITV
MGDADIPLVVQAVLLDAVGIAGQCRITCHRLFIQETIYELVIERLLLEYTLVTIGMGDPLKTGTLLGPLH
TKALKRNFRTVMQKIKSQGGKVFIGDVVST
VGNFVRPTIVEISPNADVVKEELFVPVLYVIKFTTFEEAMQINNSISPGSSNSIFTRKPHLVVPGIRSLG
IDCGIVNVNLPTRGRGGAGSDSWEQYTRRT
IW
>VvALDH10A9
METKIPCRQLFIDGKWVEPITKRRIPVINPATEQTIGLIPAATGEDVELAVDAARRAFARNKGADWAKAP
GAVRAKYLRAIAA
KITERKTELAKLEALDCGKPLDEAAWDIDDVASCFEYFADHAEALDAQQKAPLSLPMETFKCHILKEPVG
VVGLITPWNYPLLMATWKVAPALAAGCTAI
LKPSELASVTCLELADVCIEVGLPPGVLNILTGLGSEAGAPLSSHPHVDKIAFTGSTVTGSKIMTAAAQL
VKPVSLELGGKSPILVFEDVDLDKAAEWTA
FGCFWTNGQICSATSRLLVHESIAAEFLDKLVKWTKNIKISDPFEEGCRLGPVVSREQYEKILKLVSTAK
SEGATILCGGGRPQHLKMGFYIEPTIISDV
TTSMQIWREEVFGPVLCVKTFSSEDEAIELANDTQYGLAGAVISNDLERCERVSKALNAGIVWINCSQPC
FCQAPWGGNKRSGFGRELGPRGLDNYLSVK
QVTQYLSNEPWGWYQSPSKL
>VvALDH10B1

MMAVPIPSRQLFIDGEWREPLLKKRIPIINPATQEIIGDIPAATAEDVDIAVEAARRAFSKPDSWASTSG
SFRAKFLRAIADK
ILERKIELAKLEVVDCGKPIDEAISDMVSVAGCFKYYAELAEALDAKQRIPISIPMESFKTHVLKEPIGV
VALITPWNYPLLMAAWKVAPALAAGCTAIL
KPSELASVTCLELAEVCRDVALPPGVLNILTGLGPEAGAPLASHPHVDKIAFTGSTATGIKIMTTAAQTI
KPISLELGGKSPILVFEDVDLDNAIEWTLY
GCFPNNGQICSATSRLLVHENIAAKFVEKLIQWSKSIKISDPLEEGCRLGAIVSEGQYEKILNFISTAKS
EGATILYGGVRPQHLKKGFFIEPTIITDVS
TSMQIWREEVFGPVLCVKTFATEEEATQLANDTHYGLGAAVVSNDLERCERLTKVLQAGVVWVNCSQPCF
DQAPWGGIKHSGIGRELGEWGLENYLTVKQ
VTQYTSDKQWGWYSPP
>VvALDH11A3
MAGSGVFAEILDGDVFKYYTDGAWKTSSSGKSVPIINPTTRSTQYKVQACTQEEVNKVMETAKNAQKLWA
KTPLWKRAELLHK
AAAILKEQKAPIAECLVKEIAKPAKDAVTEVVRSGDLVSYCAEEGVRILGEGKFLVSDSFPGNERSKYCL
TSKIPLGVILAIPPFNYPVNLAVSKIGPAL
IAGNSLVLKPPTQGAVAALHMVHCFHLAGFPKGLINCVTGRGSEIGDFLTMHPGVDCISFTGGDTGIAIS
KKAGMVPLQMELGGKDACIILEDADLDLAA
SSIVKGGFSYSGQRCTAIKVVLVMESVADALVEKVNVKLAKLTVGAPEDDCDITPVVTESSANFIEGLIT
DAKQKEATFCQEYKREGNLIYPLLLDNVRP
DMRIAWEEPFGPVLPVIRINSVEEGIHHCNASNFGLQGCIFTRDINKAILIGDAMETGTVQINSAPARGP
DHFPFQGFKDSGIGSQGITNSINMMTKTKS
MVINLPTPTYSMG
>VvALDH11B1
MAGTGVFAEIIDGDVYKYYSEGEWRKAVSGKSVAIINPTTRKTQYRVQACSQEEVNKAMEIAKSAQKIWA
KTPLWKRAELLHK
AAAILKEHKAPIAECLVKEIAKPAKDAVTEVVRSGDLVSYTAEEGVRILGEGKFLVSDSFPGNERSKYCL
TSKIPLGVILAIPPFNYPVNLAVSKIAPAL
IAGNSIVLKPPTQGAVSALHMVHCFHLAGFPKGVISCVTGKGSEIGDFLTMHPGVNCISFTGGDTGVAIS
KKAGMIPLQMELGGKDACIVLEDADLDLVA
ANIVKGGFSYSGQRCTAVKVVLAMESIADTLVEKVNAKVAKLTVGPPEDDCDITPVVSESSANFIEGLVM
DAKQKGATFCQEYRREGNLIWPLLLDNVRP
DMRIAWEEPFGPVLPVLRINSVEEGIHHCNASNFGLQGCVFTRDINKAILISDAMETGTVQINSAPARGP
DHFPFQGLKDSGIGSQGITNSINMMTKIKS
TVINLPSPSYTMG
>VvALDH12A1
MRGRVPQIAALNWLGSLSLSRSIHNLPFATIEVEEISGSQPAEVQNLVQGKWTGSTSGETIVDPLNGEPF
IQVAEVDETGIQP
FVDSLSKCPKHGLHNPFKAPERYLMLGDISNKAAHMLSLPKVSDFFTRLIQRVAPKSYQQALGEVYVTQK
FLENFSGDQVRFLARSFAVPGNHLGQQSHG
FRWPYGPVAIVTPFNFPLEIPVLQLMGALYMGNKPILKVDSKVSIVMEQMIRLLHHCGLPMEDLDFINSD
GKTMNKLLLEANPRMTLFTGSSRVADKLAV
DLKGRIKLEDAGFDWKILGPDVQEVDYVAWVCDQDAYACSGQKCSAQSIVFMHENWFKSSLISRMKDLAA
RRKLEDLTIGPVLSFTTEAMLEHMNKLLQI
PGSELLFGGKALENHSIPPIYGALKPTAIYIPLEEMLKDGNYELVTREIFGPFQVVTDYKDNQLPRVLEA
LERMHAHLTAAVVSNDSLFLQEVIGKSVNG
TTYAGLRARTTGAPQNHWFGPAGDPRGAGIGTPEAIKLVWSCHREIIYDIGPLPSHWEIPPAT
>VvALDH18B1

MHMDAMDPTRAFVKDVKRLVIKFGTAVVTRSDGRLALGRLGALCEQIKELNSQGYQVIVVTSGAVGLGRQ
RLRYRSLLNSSFA
DLQKPQAELDGKACAAVGQNNLMALYDTLFSQLDVTSAQLLVTDNDFRDEAFRNQLTQTVDSLLALRVIP
IFNENDAVSTRKAPYEDSSGIFWDNDSLAG
LLALQLKADLLVLLSDVDGLYSGPPSDPRSKLIHTYLEKGHQGQITFGDKSRVGRGGMTAKVKSAVYSSQ
AGIPVVITSGYATGSILKVLNGERIGTLFH
RDAYKWVQVKEVGAREMAVAARESSRRLQAMSSQDRKKILLDIANALETNEELIKIENDADVEAAQLAGY
EKSLVSRLVLKPGKISSLANSIRVLANMEE
PIGHVLKRTEVADGLILEKMSCPLGVLLIVFESRPDALVQIASLAIRSGNGLLLKGGKEAKRSNAILHKV
ITEAIPDSVGKKLIGLVTSREEIPNLLKLD
DVIDLVIPRGSNKLVSQIKDSTKIPVLGHADGICHVYVDKSANMDTAKHIVLDAKVDYPAACNAMETLLV
HKDLVQTGGLNQLIVELRNEGVTLYGGPRA
SALLNLPEAHSFHHEYNSMACTVEIVDDVHSAIDHIHRHGSAHTDCIIAEDLEVAEVFLRQVDSAAVFHN
ASTRFCDGARFGLGAEVGISTSRIHARGPV
GVEGLLTTRWILRGNGQVVNGDKGVTYTHKELTLQP
>VvALDH18B3
MDSSREFVKDVKRVIVKVGTAVVTRADGRLAVGRVGALCEQLKELNSQGYEVILVASGAVGVGIQRLRYR
RLVNSSLDDLQKP
QIELDSKACAAVGQSSLMALYDTLFSQLDVTASQLLVTNSDFRNPDFRMQLSETVGSLLDLRVIPIFNEN
DAISTRDHPNEDSSGIFCDNDSLATLLALE
LKADLLVLLSDVEGLYNAPPNKPHAELIHTYVKEKHEGEITFGDKSKVGRGGMTAKVKAANQAADAGTPT
VITSGYATDNIIKVLQGQRVGTLFHKDAHS
WTLVKEIGAREMAVAARECSRRLQALPSKDRRKILLDIADALEANESLIKVENEVDVAAAQEAGYDKSLL
SRLTLKPGKISGLAKSIRMLADMKEPIGHV
LSRMELADGLILEKTSCPMGVLLVVFEARPDALVQIASLAIRSGNGLLLKGGKEANRSNAILHKVITEAI
PDTVGEKLIGLVTTREQIPDLLKLDDVIDL
VIPRGSNKLVSQIKDSTKIPVLGHADGICHVYIDKSANMKMAKHIVLDAKTDYPAACNAMETLLVHKDFL
SNGGFNKLVTELQRKGVTLHGGPRASALLN
ISQVNSFHHEYNSMACTVEIVDDVHSAIDHIHRHGSSHTDCIITEDSKIAEIFLLQVDSAAVFHNASTRF
CDGARFGLGAEVGISTSRIHARGPVGVEGL
LTSRWILKGNGHIVDGDEGVIYTHKSLALQS
>VvALDH22A1
MAFWWPLLVLGFAFALCRFLLMLIPPNVPSIDVDASDVLDDGKTKENSFIYIPSRGRTPEKVQCYEPATM
KYLGFCPALRPDE
VREHVAQARKAQKIWARSSFKQRRQFLRILLKYIIEHQELICEISSRDTGKTMVDASLGEIMTTCEKITW
LLSEGERWLKPEYRSTGRSMLHKTAKVEFH
PLGVIGAIVSWNYPFHNIFNPMLAAVFSGNGVVIKVSEHASWSGCFYLRIIQAALAAVGAPENLVDVITG
FAETGEALVSSVDKIIFVGSPGVGKTIMRN
ASDTLIPVTLELGGKDAFIVCEDVDVPHVAQIAVRAALQSSGQNCAGAERFYVHQDIYSKFVAEVVRIVK
SVTAGPPLSGKYDMGAICMQEHSEKLQNLV
NEALDKGAEFAGRGSFGNLGEDAVDQFFPPTVLVNVNHSMQLMQEEAFGPILPIMKFSSDEEVVKLANDS
RYGLGCAVFSGSQRRAKAIASQIHCGMAAI
NDFASTYMCQSLPFGGVKDSGFGRFAGIEGLRACCLVKSVVEDRWWPFIKTKIPKPIQYPVADNGFEFQE
SLVEALYGLNVWDRLRALVHVLKMLTEQNT
PSNSTKRKND
>SlALDH2B1
MDSLLMQPQTFPTFDPRTGEAITTVAEADTEDVNRAVFAARKAFDEGPWPKMTCAERSCIMLQFADLLER
HSDELAALETWD

KGKPYEQAANEEIPMLIRLFRYYAGWADKIHGLTAPADSLHHVQTLHEPIGVAGQIIPWNFPLLMFAWKV
GPALACGNTVVLKPAEQTPLSALYVSKLFH
EVGLPPGVLNVIPGSGSAGADLASHMDVDKIAFTGSTETGKAVVGAAAKSNLKPVTLELGGKSPFIICED
ADVDKAVELAHSAVFFNQGQCCCAGSRTFV
HERVYDEFVEKAKARALKRIVGDPFKKGVEQGPQIDTEQFEKILKYIKSGTESGATLESGGEKLGSKGFY
VQPTVFSNVQDNMLIARDEIFGPVQSLLKF
KDVEEVIRRANSSHYGLAAGVFTQNIDAANTISRALRVGTVWVNCFNIFDAAIPFGGYKMSGHGREKGVY
SLSNYLQVKAIVTPLKNPAWL
>SlALDH2B3
MAARVFLSRSVHLLSKGKRSHLGKIAAYKYSTAAAFEEPVKPTVNVDHTKLLINGQFVDSASGKTFPTLD
PRTGEVIAHIAE
GDAEDINRAVAAARKAFDEGPWPRMTAYERSKILLRLADLIEKHNDQIATLETWDTGKPYAQAAKIEVPM
VVRLLRYYAGWADKIHGMTIPADGPYHVQT
LHEPIGVAGQIIPWNFPLLMFSWKIGPALACGNTIVLKTAEQTPLSALYVASLFQEAGLPEGVLNIISGY
GATAGASLCSHMDVDKLAFTGSTETGKTIL
ELAAKSNLKPVTLELGGKSPFIVCEDADIDTAVEQAHFALFFNQGQCCCAGSRTYVHEKVYDEFLEKAKA
RALKRVVGDPFKSGTEQGPQIDSKQFDKIM
KYIRSGVDSGATLETGGEQFGKKGYYIRPTVFSNVKDDMLIAQDEIFGPVQSILKFKDLDEVVRRANSSR
YGLAAGVFSQNIDTANTLARALRVGTVWIN
CFDTFDATIPFGGYKMSGQGREKGEYGLKNYLQVKAVVTPLKNPAWL
>SlALDH2B4
MAFRLITSRLSHSSSSSLASLFQGRNSRVAATAALRYTTAAQDPIKPSVNVEYTKLFINGQFVDSASGKT
FPTLDPRTGEVI
AHVAEGDVEDINRAVVAARNAFDEGPWPKMSAYERSKILFRIADLIEKHNDEIATLETWDSGKLYQQVAT
IEIPMIVRLLRYYAGWADKIHGMTVPADGP
YHVQTLHEPIGVVGQIIPWNFPLLMFAWKIGPALACGNTVVLKTAEQTPLSALYVSKLLQEAGLPEGVLN
VISGFGPTAGAALSSHMDVDKLAFTGSTDT
GKTIMSLAANSNLKPVTLELGGKSPFIVCEDADVDQAVEFAHFALFFNQGQCCCAGSRTYVHESIYDEFV
EKAKARALKRTVGDPFDSSNEQGPQISSEQ
FEKVLKYIRSGIESGATLETGGDRLGTQGYYIKPTVFSNVKDDMLIATDEIFGPVQSILKFKDHDEVIRR
ANATKYGLAAGVFTKNIDTANTFMRALRVG
TIWINCFDIFDAAIPFGGYKMSGQGREKGEYSLKQYLQVKAVVTSLKNPAWL
>SlALDH2B7a
MAARRISSLLSRSLNLPSVSASLGRSHGVARHINRFSTAAAVEEIITPPVQINHTKLLINGQFVDSASGK
TFPTLDPRTGEVIANVAEGDLEDVNRAVAAARKAFDEGPWPKMSAYERSRIMLKFADLVEKHNDEIAALE
TWDNGKPYLQAAQAEVPSFVRLFRYYAGWADKIHGLTVPADGPHHVQILHEPIGVAGQIIPWNFPLLMMA
WKVGPALACGNTIVLKTAEQTPLTALYVANLLHEAGLPPGVLNIVSGFGPTAGAALASHMDVDKLAFTGS
TETGQTVLQLAAKSNLKPVTLELGGKSPFIICEDADIDHAVELAHFALFFNQGQCCCAGSRTYVHERVYD
EFVEKAKARAMRRVVGDPFKKGVEQGPQIDSEQFQKILRYIREGRDSSATLECGGDRIGSKGYFIQPTVF
SNVKEDMSIAQDEIFGPVQCVFKFKDIGEVIKRANNTRYGLAAGVFTKNIDTANTLTRGLRAGTVWVNCY
DIFDAGIPFGGYKMSGMGREKGIYSLNNYLQVKAVVTPLKNPAWI
>SlALDH2B7b
MNTHTRRIISQLRTSIRYSNYWRKGIRRFCSSAVVHEEPITPPVEVKYNQLLINGQFVDAASGKTFPTFD
PRTGEAITTVAEADTEDVNRAVFAARKAFDEGPWPKMTCAERSCIMLQFADLLERHSDELAALETWDKGK
PYEQAANEEIPMLIRLFRYYAGWADKIHGLTAPADSLHHVQTLHEPIGVAGQIIPWNFPLLMFAWKVGPA
LACGNTVVLKPAEQTPLSALYVSKLFHEVGLPPGVLNVIPGSGSAGADLASHMDVDKIAFTGSTETGKAV
VGAAAKSNLKPVTLELGGKSPFIICEDADVDKAVELAHSAVFFNQGQCCCAGSRTFVHERVYDEFVEKAK
ARALKRIVGDPFKKGVEQGPQIDTEQFEKILKYIKSGTESGATLESGGEKLGSKGFYVQPTVFSNVQDNM

LIARDEIFGPVQSLLKFKDVEEVIRRANSSHYGLAAGVFTQNIDAANTISRALRVGTVWVNCFNIFDAAI
PFGGYKMSGHGREKGVYSLSNYLQVKAIVTPLKNPAWL
>SlALDH2B7c
MAFRLITSRLSHSSSSSLASLFQGRNSRVAATAALRYTTAAQDPIKPSVNVEYTKLFINGQFVDSASGKT
FPTLDPRTGEVIAHVAEGDVEDINRAVVAARNAFDEGPWPKMSAYERSKILFRIADLIEKHNDEIATLET
WDSGKLYQQVATIEIPMIVRLLRYYAGWADKIHGMTVPADGPYHVQTLHEPIGVVGQIIPWNFPLLMFAW
KIGPALACGNTVVLKTAEQTPLSALYVSKLLQEAGLPEGVLNVISGFGPTAGAALSSHMDVDKLAFTGST
DTGKTIMSLAANSNLKPVTLELGGKSPFIVCEDADVDQAVEFAHFALFFNQGQCCCAGSRTYVHESIYDE
FVEKAKARALKRTVGDPFDSSNEQGPQISSEQFEKVLKYIRSGIESGATLETGGDRLGTQGYYIKPTVFS
NVKDDMLIATDEIFGPVQSILKFKDHDEVIRRANATKYGLAAGVFTKNIDTANTFMRALRVGTIWINCFD
IFDAAIPFGGYKMSGQGREKGEYSLKQYLQVKAVVTSLKNPAWL
>SlALDH2B7d
MAARVFLSRSVHLLSKGKRSHLGKIAAYKYSTAAAFEEPVKPTVNVDHTKLLINGQFVDSASGKTFPTLD
PRTGEVIAHIAEGDAEDINRAVAAARKAFDEGPWPRMTAYERSKILLRLADLIEKHNDQIATLETWDTGK
PYAQAAKIEVPMVVRLLRYYAGWADKIHGMTIPADGPYHVQTLHEPIGVAGQIIPWNFPLLMFSWKIGPA
LACGNTIVLKTAEQTPLSALYVASLFQEAGLPEGVLNIISGYGATAGASLCSHMDVDKLAFTGSTETGKT
ILELAAKSNLKPVTLELGGKSPFIVCEDADIDTAVEQAHFALFFNQGQCCCAGSRTYVHEKVYDEFLEKA
KARALKRVVGDPFKSGTEQGPQIDSKQFDKIMKYIRSGVDSGATLETGGEQFGKKGYYIRPTVFSNVKDD
MLIAQDEIFGPVQSILKFKDLDEVVRRANSSRYGLAAGVFSQNIDTANTLARALRVGTVWINCFDTFDAT
IPFGGYKMSGQGREKGEYGLKNYLQVKAVVTPLKNPAWL
>SlALDH2C4
MAEMNGNSETQFQIPKIKFTKLFINGEFVDSVSGNTFETIDPRNEEVIARISEGDKEDIDLAVKAAREAF
DNGPWPRLSAAERRRIMLKFADLIIENAEEIAALDAMDAGKLFVPVKNMDIPAAAEIIRYYAGAADKIHG
TTLKMSREMQGYTLLEPIGVVGHIIPWNFPTQMFLMKVGPALAAGCTMIVKPAEQTPLSALYYAQLAKQA
GVPDGVINVVTGFGSTAGAALCSHMDVDKISFTGSTEVGRLVMQAAALSNLKPVSLELGGKSPFIVFDDV
DVDKVAPLALVGILFNKGEICVAGSRLFIQEGIYDKFVKKLEQMVKTWVVGDPFDPNSHQGPQVDKKQYE
RVLSYIEHGKREGAKLLTGGNALDRKGYFIEPTIFIDVEDDMKIAKEEIFGPVLAVMKFKTVEEVIKRAN
CTNYGLAAGVMTNNLNIANTVSRSIRAGVIWINCYFAFDPDCPYGGYKCSGFERDLGMEGLHKYLQVKSV
ATPIYNSPWL
>SlALDH3F1a
MSTTTMTKLCNSYPTTPQIMLECEKELEVLKETFKSGKTKEESWRRSQLKNLLKLLEEKENDIFKALKQD
LGKHKVEAYRDEVGTLVKSVHYALDGLKQWMSPKKAKLPIAAFPSSAELLPEPLGLVLIISSWNFPFSLS
LEPLIGAIAAGNVVLLKPSDQAPASSSVLAKIIPNYLDNKAIKVIEGDYTVGDKLLQQKWDKIFFTGSPK
VAQIVMGAAAKHLTPVTLELGGKCPAIIDSLSSSWDKKIAMKRILSGKFGSCAGQACIGIDYILVDNTFV
NELVKLIKLGIPKMLGENPKESHSISRIVNKNQFLRLKNLLDEPMVKKSIIYGGSSDEDNLYIEPTVLLD
PPLQSTIMTDEIFGPLLPIITLDKIEDSIEFINARPKPLTIYAFTKNEEFKRKITKGTSSGSLVFNDTII
QYAADTLPFGGVGQSGFGRYHGKFSFDTFSHEKAIARRSFLTDIWFRYPPWSDHTLQLFRSAFIYDYLSV
VLITLGLKRA
>SlALDH3F1b
MTGLSLEPLIGAIAYENVALLKPSDQAPASSSVLAKIIPNYLDNKAIKVIEGDYTVGDKLLQQKWDKIFF
TGSPKVAQIVMGAAAKHLNPVTLELGGKCPAIIDSLSSSWDKKIAMRRILSGKFGSCAGQACSGIDYILV
DNIFVNELVKLIKLGIPKMLGENPKESHSISRIVIKNQFLRLKNLLDEPMNQRYIEPTVLLYPPVQSTIM
IDEIFGPLSPIITLDKIEDNIEFINARPKPLTIYAFTKNEEFKGKITKGTCSGSLVFSDTIIQDLEQFLP
>SlALDH3F1c
MRTEKANHLKDKMEELSLNSSNANLVESSGTVVKDRKVGHRAARCYQRKGQDSKKEGQSDVQANLVEGNE
VVVVVVVEANLQANKIGRVLETGASRHFSANKELLHDFEESTDRECIYIGDSTTDVVMGLSLEPLIGAIA
AGNVALLKPLDQAPASSSVLAKIIPNYLDNKAIKVIEGDYTVKLIKLGIPIMLGENPNESHSISRIVNKN
QFLRLKNLLDEPMVKKSIIYGGSSDEDNLDIEPTVLLDPPVQSTIMADEIFGPLSPIITLDKIEDSIEFI

NARPKPLTIYAFTKNEEFKRKITKRTSSGSLVFNDTIIQYATDTLPFGGVGQSGFGRYHGKFSFDTFSHE
KAIAKRSFLTDIWFRYSPWSDHTLQLFRSAFIYDYISVVLITLGLKRA
>SlALDH3F1d
MDVVEEDVLGEVTTAFRSRRTRSVAWRKAQLQAILKLLDENEEEIFEALRQDLGKHPVESYRDEVGVVRK
SATNALRCVEKWMAPQKAPIPLVLFPARGAVVSEPLGVVLIFVSWNFPISLALDPVIGAISAGNAIVLKP
SELAPKCSSVLANTIPRYLDPEAIKVVEGGHDVSEQLLQLKWDKIFFTGSPRVGRLIMSAAAKHLTPVTL
ELGGKCPTILDRLSNFSDLQVAVKRIVGGKWGPCNGQACIGIDYVLVETQFAPVLIELLEKSIKTFYGEN
LKTLANLARIVNKHHFDRVHNLLKDPKVAASVVYGGSVDEENMAIEPTILLNPPLDADIMNEEIFGPLLP
IITLKNIEESIPFINSRPKPLAIYAFTKNDSLKEKILQETSSGSLTFNDAMIQFLCDTLPFGGVGQSGYG
RYHGKFTFDTFSHEKAVLHRSFLVELESRYPPWNDFKMEFVRLAYNYDYLGMILLLLGLRGLFRTNRRQ
>SlALDH3H1
MDAEAIVKELRGTYGSGKTKSYEWRVSQLKALLKIAENHEKEITDALYSDLSKPELEAFIHEVSMMKTAC
KLALKELKWWMKPEKVKTSLTSFPSSAEIVPEPLGVVLVISAWNYPFLLSLDPVIGAIAAGNAVVLKPSE
IAPATSSVLAKLLGQYMDVSAIRVVEGAVPETTALLEQKWDKIFYTGNGKVGRIVLAAAAKHLTPVVLEL
GGKSPVVVDSNIDYKIAVRRIIAGKWGCNNGQACISPDYIITTKESVPKLLDAMKQELEKFYGKDPLKSG
DLSRIVNANHFQRLSKLLDDNKVVDKVVHGGQRDENNLKISPTILLDVPEDSLIMKEEIFGPLLPIITVN
KVEDSIQFIKAREKPLAAYLFTSNKKLEEEFVMNISAGGLLINDTTLQVALSTLPFGGVGESGMGSCHGK
FSFDTFSHKKAVLRRSFAGDVPARYPPYTAGKARFLKALLNGDIIGLIRALIGW
>SlALDH5F1a
MQMIRVRTRMALSACAMLYRSSISGPVRLMTTDTQSVAAKLSSSGLLRSQALIGGKWVDAYDGKTIKVHN
PATGEVITDVPCMGGRETNDAISSAYDAFSSWSKLTAAERSRYLRKWYDLIMAHKEELGQLMTLEQGKPL
KEAIGEVSYGAGFIEFSAEEGKRIYGDIIPSPLADRRLFVLKQPVGVVGAITPWNFPLAMITRKVGPALA
CGCTVVIKPSELTPLTALAAAELSIQAGIPPGVVNVVMGNAPDIGDALLASPQVRKITFTGSTKVGKKLM
EGAAATVKKVSLELGGNAPCIIFDDADLEVALKGALATKFRNTGQTCVCANRILVQEGIYDKFANAFAKA
VQNMKVGDGFTEGVEQGPLINEAAVQKVEYFVDEATSKGAKVLVGGKRHSLGMTFYEPTVVTGVNSEMLL
AKEEVFGPVAPLLKFKTDEEAIQMANDTNAGLAAYIFSTNIKRAWRVTEALEYGIVGVNEGLVSTEVAPF
GGVKQSGLGREGSKYGMDEYLEMKYVCLGSMS
>SlALDH5F1b
MQMIRVRTRMALSACAMLYRSSISGPVRLMTTDTQSVAAKLSSSGLLRSQALIGGKWVDAYDGKTIKVHN
PATGEVITDVPCMGGRETNDAISSAYDAFSSWSKLTAAERSRYLRKWYDLIMAHKEELGQLMTLEQGKPL
KEAIGEVSYGAGFIEFSAEEGKRIYGDIIPSPLADRRLFVLKQPVGVVGAITPWNFPLAMITRKVGPALA
CGCTVVIKPSELTPLTALAAAELSIQAGIPPGVVNVVMGNAPDIGDALLASPQVRKITFTGSTKVGKKLM
EGAAATVKKVSLELGGNAPCIIFDDADLEVALKGALATKFRNTGQTCVCANRILVQEGIYDKFANAFAKA
VQNMKVGDGFTEGVEQGPLINEAAVQKVEYFVDEATSKGAKVLVGGKRHSLGMTFYEPTVVTGVNSEMLL
AKEEVFGPVAPLLKFKTDEEAIQMANDTNAGLAAYIFSTNIKRAWRVTEALEYGIVGVNEGLVSTEVAPF
GGVKQSGLGREGSKYGMDEYLEMKYVCLGSMS
>SlALDH6B2
MMQFSVHRVKKLRSLTPGIFAVANHHFSVATESSWKHRTSLRVPNLIGGSFVDSQSSEFVDVINPATQEV
VSQIPLTTDKEFKSAVSAAKEAFPSWKNTPITTRQRVMLKFQELIRKNMDKLAFNVTTEQGKTLKDAQGD
VFRGLEVVEHACGMATLQMGEYGSNVSNGIDTYSLREPLGVCAGICPFNFPAMIPLWMFPVAATCGNTFI
LKPSEKDPGASMMLAELAMEAGLPDGVLNIVHGTHDVVNAICDDDDIRAVSFVGSNQAGMHIYSRASAKG
KRVQSNMGAKNHGVVMPDANIDSTVNALVGAGFGAAGQRCMALSTVVFVGDSKPWEEKLLERAKTLKVNA
GTEPDADLGPVISKQAKERVCRLVQSGVDSGAKLLLDGRDIVVPGYEKGNFVGPTILCGVTPDMECYKEE
IFGPVLLCMQANSLDEAINIVNQNMYGNGAAIFTTSGVAARKFQTEIESGQIGINVPIPVPLPFFSFTGS
KASFVGDLNFYGKAGVQFYTQIKTVTQQWKDLSSGSGNSLAMPTSQK
>SlALDH7B4a
MTNFTMEEYEFLKELGIGPQNLGCYVNGTWKATGPVISTVNPASNQIIAEVYEASARDYEEGMSACAEAA
KIWVQVPAPKRGEIVRQIGDALRANLQQFGRLVSLEMGKILPEGIGEVQEVIDMCDFAVGLSRQLNGSII

PSERPNHMMLETWNPLGIVGVITAFNFPCAVLGWNACIALVCGNCVVWKGAPTTPLVTIAMTKIVASVLE
KNNLPGSIFTAFCGGAAVGQAIAMDTRIPLVSFTGSSKVGLAVQQTVSQRFGKCLLELSGNNAIIIMDDA
DIKLAVRSVLFAAVGTAGQRCTTCRRLLVHESIYDKVLEPLVDVYKQVKIGDPLEKGTLLGPLHTRTSRE
NFEKGIHNIKSQGGKILTGGSVVESEGNFVRPTIVEISSKAEIVKEELFAPVLYVMKFKTFEEAVEINNS
VPQGLSSSIFTRNPENIFKWIGPQGSDCGIVNVNIPTNGAEIGGAFGGEKGTGGGREAGSDSWKQYMRRS
TCTINYGSELPLAQGINFG
>SlALDH7B4b
MTNFTMEEYEFLKELGIGPQNLGCYVNGTWKATGPVISTVNPASNQIIAEVYEASARDYEEGMSACAEAA
KIWVQVPAPKRGEIVRQIGDALRANLQQFGRLVSLEMGKILPEGIGEVQEVIDMCDFAVGLSRQLNGSII
PSERPNHMMLETWNPLGIVGVITAFNFPCAVLGWNACIALVCGNCVVWKGAPTTPLVTIAMTKIVASVLE
KNNLPGSIFTAFCGGAAVGQAIAMDTRIPLVSFTGSSKVGLAVQQTVSQRFGKCLLELSGNNAIIIMDDA
DIKLAVRSVLFAAVGTAGQRCTTCRRLLVHESIYDKVLEPLVDVYKQVKIGDPLEKGTLLGPLHTRTSRE
NFEKGIHNIKSQGGKILTGGSVVESEGNFVRPTIVEISSKAEIVKEELFAPVLYVMKFKTFEEAVEINNS
VPQGLSSSIFTRNPENIFKWIGPQGSDCGIVNVNIPTNGAEIGGAFGGEKGTGGGREAGSDSWKQYMRRS
TCTINYGSELPLAQGINFG
>SlALDH10A8
MAIPNIRIPCRQLFIDGEWREPLKKNRLPIINPANEEIIGYIPAATEEDVDMAVKAARSALRRDDWGSTT
GAQRAKYLRAIAAKVLEKKPELATLETIDNGKPWFEAASDIDDVVACFEYYADLAEALDSKKQTEVKLHL
DSFKTHVLREPLGVVGLITPWNYPLLMTTWKVAPALAAGCAAILKPSELASITSLELGEICREVGLPPGA
LSILTGLGHEAGSPLVSHPDVDKIAFTGSGPTGVKIMTAAAQLVKPVTLELGGKSPIVVFDDIHNLDTAV
EWTLFGCFWTNGQICSATSRLIIQETIAPQFLARLLEWTKNIKISDPLEEDCKLGPVISRGQYEKILKFI
STAKDEGATILYGGDRPEHLKKGYYIQPTIITDVDTSMEIWKEEVFGPVLCVKTFKIEEEAIELANDTKF
GLGAAILSKDLERCERFTKAFQSGIVWINCSQPCFWQPPWGGKKRSGFGRELGEWSLENYLNIKQVTQYV
TPDEPWAFYKSPSKL
>SlALDH10A9
MANRNVPIPRRQLYIGGEWREPVKKNRIPIINPATEEIIGDIPAATAEDVDIAVEAARKAIARDDWGSTT
GAQRAKYLRAIAAKVLEKKSVLATLESLDSGKTLYESAADMDDVAGCFEYYAGLAEALDSRRMTPVNLNS
DSYKSYVLREPLGVVGLITPWNYPLLMAIWKVAPALAAGCAAILKPSELASITCLELGEICREIGLPSGA
LNILTGLGPEAGGPLASHPHVDKISFTGSGPTGSKIMTAAAQLVKPVSLELGGKSPIVVFDDIDNLDIAA
EWTLFGIFANTGQVCSATSRLIVQENIASAFMDRLLKWTKNIKISDPLEEDCKLGPVVSAGQYEKVLKFI
SNAKSEGATILCGGERPQHLKKGYYVQPTIITDVNTSMEIWKEEVFGPVLCVKTFKTEEQAIELANDTKY
GLGAAVMSKDVKRCERFTKAFQTGIIWINCSQPTFNELPWGGKKRSGFGRDLGKWGLENFLNIKQVTEYT
SAEPLAFYKSPSKN
>SlALDH11A3a
MAGNGVFAEIIDGEVYKYYCEGEWKKSASGKSVAIINPTTRKTQYKVQACTQEEVNKVMEIAKAAQKSWA
KTPLWKRAELLHKAAAILKEHKAPIAECLVKEIAKPAKDAVTEVVRSGDLVSYTAEEGVRILGEGKFLVS
DSFPGNERTKYCLTSKIPLGVILAIPPFNYPVNLAVSKIAPALIAGNSLVLKPPTQGAVAALHMVHCFHL
AGFPKGLISCVTGKGSEIGDFLTMHPGVNCISFTGGDTGVAISKKAGMVPLQMELGGKDACIVLEDADLD
LAAGNIVKGGFSYSGQRCTAVKVVLVMESVADILVEKVNAKVAKLTVGPPEDNCDITPVVSESSANFIEG
LVMDAKEKDATFCQPYKREGNLIWPLLLDNVRPDMRIAWEEPFGPVLPVIRINSVEEGIHHCNASNFGLQ
GCVFTKDINKAILISDAMETGTVQINSAPARGPDHFPFQGIKDSGIGSQGITNSINMMTKVKTTVINLPT
PSYTMGKL
>SlALDH11A3b
MAGNGVFAEIIDGEVYKYYCEGEWKKSASGKSVAIINPTTRKTQYKVQACTQEEVNKVMEIAKAAQKSWA
KTPLWKRAELLHKAAAILKEHKAPIAECLVKEIAKPAKDAVTEVVRSGDLVSYTAEEGVRILGEGKFLVS
DSFPGNERTKYCLTSKIPLGVILAIPPFNYPVNLAVSKIAPALIAGNSLVLKPPTQGAVAALHMVHCFHL
AGFPKGLISCVTGKGSEIGDFLTMHPGVNCISFTGGDTGVAISKKAGMVPLQMELGGKDACIVLEDADLD
LAAGNIVKGGFSYSGQRCTAVKVVLVMESVADILVEKVNAKVAKLTVGPPEDNCDITPVVSESSANFIEG

LVMDAKEKDATFCQPYKREGNLIWPLLLDNVRPDMRIAWEEPFGPVLPVIRINSVEEGIHHCNASNFGLQ
GCVFTKDINKAILISDAMETGTVQINSAPARGPDHFPFQGIKDSGIGSQGITNSINMMTKVKTTVINLPT
PSYTMG
>SlALDH11A4a
MALRMWASSTANALRVSSTVSRTNFSLSRCFSTVLEGLKYASSHEWVKHEGSVATIGITDHAQDHLGEVV
FVDLPDSGTSVSHGSSFGAVESVKATSDINSPISGEIVEVNTKLSETPGLINSSPYEDGWMIKVKPSNPS
ELESLMGAKEYTKLCDEEEIH
>SlALDH11A4b
MALRMWASSTANALRVSSTVSRTNFSLSRCFSTVLEGLKYASSHEWVKHEGSVATIGITDHAQDHLGEVV
FVDLPDSGTSVSHGSSFGAVESVKATSDINSPISGEIVEVNTKLSETPGLVKCLLTHISTRALMKTDG
>SlALDH12A1
MMYRLSAYRQLQKRASSSHLNWITLFDSRRSNHTLSFATVKAEEVSGSQPAEVHNLVQGKWTKSSSWNTI
LDPLNGQPFIKVAEVNESELQPFVESLSKCPKHGLHNPFKAPERYLMLGDVSTKAAHALGLPEVSDFFAK
LIQRVSPKSYQQALVEVLVTQKFLENFCGDQVRFLARSFAVPGNHLGQQSHGFRWPYGPVAVIAPFNFPL
EIPLLQLMGALYMGNKPVLKVDSKVCIVMEQMLRLLHECGLPVDDVDFINSDGKTMNKLLVEAKPRMTLF
TGSSRVAEKLADDLSGRVKLEDAGFDWKILGPDVNEVDYVAWVCDQDAYACSGQKCSAESILFMHENWSK
SSLLDKMTELAARRKLDDLTIGPVLTVTTETMLDHAKKLLQIPGSRLLFGGEALQNHSIPKIYGAIKPTA
IFVPLEEILKDEHYPLVTKEIFGPFQVVTEYKDNQLPLVLDALEKMHAHLTAAVVSNDILFLQKVIGNSV
NGTTYAGLRARTTGAPQNHWFGPAGDPRGAGIGTPEAIKLVWSCHREIIYDVGPMPLGWKVPAST
>SlALDH18B1
MDSADPARAFVKDVKRIIIKVGTAVVTRGDGRLALGRMGSLCEQIRELTSQGFEVILVTSGAVGVGRQRL
RYRKLINSSFADLQKPQGDLDGKACAAVGQNGLMALYDTLFSQLDVTSAQLMVTDNDFRDPDFRRQLNET
VNSLLCLKVVPIFNENDAISTRKAPYEDSSGIFWDNDSLAALLALELKADLLVLLSDVEGLYTGPPTDPQ
SELIHTYVKEKHEGLITFGDKSRVGRGGMTAKVKAAVYAAYAGIPVVITSGFANNNIIKALDGQRVGTLF
HREAIKWASIGDFDAREMAVSARECARRLQTLSSQERSKILLDIADALEAKEEEILAENEADVAAAQQAG
YENALISRLAMKPGKISSLANSVRVLANMDEPVGRILKRTELADGIILEKTSSPLGVLLIIFESRPDALV
QIASLAVRSGNGLLLKGGKEAKRSNAILHKVITSSIPPIVGERLIGLVTSREEIPELLKLDDVIDLVIPR
GSNKLVSQIKAATKIPVLGHADGICHVFIDKSADLDMAKRIVLDAKTDYPAACNAMETLLVHEDLVQTGG
LNDLILELQEKGVSLFGGPKASSVLNIPEANSFHHEYGALACTVEIVEDVNTAIEHIHRHGSAHTDSIIT
EDKEVAELFLRQVDSAAVLHNASTRFSDGFRFGLGAEVGISTSRIHARGPVGVEGLLTTRWLARGSGQVV
DGDKEIVYTHKDLNLEA
>SlALDH18B2
MDSADPARAFVKDVKRIIIKVGTAVVTRGDGRLALGRMGSLCEQIRELTSQGFEVILVTSGAVGVGRQRL
RYRKLINSSFADLQKPQGDLDGKACAAVGQNGLMALYDTLFSQLDVTSAQLMVTDNDFRDPDFRRQLNET
VNSLLCLKVVPIFNENDAISTRKAPYELSLQDSSGIFWDNDSLAALLALELKADLLVLLSDVEGLYTGPP
TDPQSELIHTYVKEKHEGLITFGDKSRVGRGGMTAKVKAAVYAAYAGIPVVITSGFANNNIIKALDGQRV
GTLFHREAIKWASIGDFDAREMAVSARECARRLQTLSSQERSKILLDIADALEAKEEEILAENEADVAAA
QQAGYENALISRLAMKPGKISSLANSVRVLANMDEPVGRILKRTELADGIILEKTSSPLGVLLIIFESRP
DALVQIASLAVRSGNGLLLKGGKEAKRSNAILHKVITSSIPPIVGERLIGLVTSREEIPELLKLDDVIDL
VIPRGSNKLVSQIKAATKIPVLGHADGICHVFIDKSADLDMAKRIVLDAKTDYPAACNAMETLLVHEDLV
QTGGLNDLILELQEKGVSLFGGPKASSVLNIPEANSFHHEYGALACTVEIVEDVNTAIEHIHRHGSAHTD
SIITEDKEVAELFLRQVDSAAVLHNASTRFSDGFRFGLGAEVGISTSRIHARGPVGVEGLLTTRWLARGS
GQVVDGDKEIVYTHKDLNLEA
>SlALDH19
MALSVQEMGQRAKKATAQVAGLSLATRNTLLKNMGAALLMRQDEIIAANQQDLVAYGASLSRPMQKRLTL
DSDALTAIAESLAAVATLPDPLAGPYDTWENHAGLKIVKKIVPLGVVAMIYEARPNVTVDAAALALKSGN
AVILRGGKEAIHSNTVLATILRDVLIDQNLNPDIIQLITDTTHESVNTLLHMREAIDVLIPRGSAAFIDY
VVANATVPVIETGAGNTHIFVDASADQAAALRIIHNAKTQKPAVCNAAEKLLIHEAIAQEFLPKIADRLI

AARVALRGDQASLGIDGRLTPASDADWDTEYNDLVMGIKIVPDVTAAIDWINTHTTHHSETIISQDPDNI
AAFMNQVDAAVVYQNASSRFTDGFEFGFGAEIGISTQKLHARGPMGLPALTTIKYEALAMAIRA
>SlALDH22A1
MAFWWPLIVIAIAFAICKLLLMLIPDNVPSIDVDTSDVLDDGNQTKDNSFIYIPSRRHTDKVQCYEPATM
KYLGYFPALKPDEVKERVVQARKAQKIWAKSSFKQRRLFLRILLKYIIEHQDLICNISSRDTGKTMVDAS
LGEIMTTCEKIHWLLSEGEKWLKPEYRSCGRSMLHKVAKVEFSPLGVVGAIVSWNYPFHNIFNPMLAAVF
SGNSIVIKVSEHASWSGCFYLRIIQTALAAVGAPENLVEVITGFAETGEALVSSVDKIIFVGSPGVGKKI
MRSASDTLIPVTLELGGKDAFIVCEDVDVPHVAQIAARGALQSSGQNCAGAERFYVHKDVYSSFVAEVVK
IVKSVTAGPPLSGKYDMGAICMQEHSERLQYLVNDALDKGAEIVARGSVGNIGEGAVDQYFPPTVIVNVN
HTMKLMQEEAFGPILPIMKFSSDEEVVQLANDSSYGLGCAVFSGSQRRARQIASQLHCGVAAVNDFASNY
MCQSLPFGGVKDSGFGRFAGIEGLRACCLVKSVVEDRWWPFIKTKIPKPIQYPIAENGFEFQESLVHTLY
GLNIWDRLRALVNVLKILSEQPPAPTSNRRRND
>PtALDH2B3
MAARRISSLLSRSLSASASFLLSRGKNPSRGRSIYRFITAKALEEPITPPVQISYTQHFINGKFVDAASG
KTFPAYDPRTGEVIAHVAEGDNEDVNRAVAAARKAFDEGPWPKMSAYERSLIMLRFADLVDKHRDELAAL
ESWNSGKPYEQSAKSELPSFARLFRYYAGWADKIHGLTVPADSNHYVQTLHEPIGVAGQIIPWNFPLIML
AWKVGPALACGNTIVLKSAEQTPLTALHAAKLFQEAGLPPGVLNVVSGYGPSAGAALASHMNVDKLAFTG
STETGKIILELAAKSNLKSVTLELGGKSPFIVCEDADVDKAVELAHHALFFNQGQCCCAGSRTYVHERVY
DEFIEKAKARALRRVVGDPFKKGVEQGPQIDSDQFEKVLRYIRSGVESNATLECGGQRFGSKGYFIQPTV
FSNVEDDMLIAQDEIFGPVQSILKFKNVDEVIRRSNSTRYGLAAGIFTKNVDTANTLSRALRVGTVWVNC
FDVFDAAIPFGGYKMSGIGREKGIYSLNNYLQVKAVVTPLKNPAWL
>PtALDH2B4
MAARRISSLLSRSLSAPSAPSASTPLLLSRGKNPGRGRGVCSYTQHLINGQFVDAASGKTFPTHDPRTGE
VIAHVAEGDAEDVNRAVAAARKAFDEGPWPKMSAYERSLIMLRFADLVDKHRGELAALESWNSGKPYEQS
AKSELPSFARLFRYYAGWADKIHGLTVPADGNHHVQTLHEPIGVAGQIIPWNFPLIMFAWKVGPALACGN
TIVLKSAEQTPLTALYAAKLFQEAGLPPGVLNVVSGYGPSAGAALACHMDVDKIAFTGSTETGKIILELA
AKSNLKAVTLELGGKSPFIVCEDADVDKAVELAHFALFFNQGQCCCAGSRTYVHERVYDEFVEKAKARAL
RRVVGDPFKKGVEQGPQIDSEQFEKILRYIKSGVESNATLECGGQRFGSKGYFIQPTVFSNVQDDMLIAK
DEIFGPVQSILKFKNIDEVIQRANTTRYGLAAGIFTKNVDTANTLSRALRVGSVWVNCFDVFDAAIPFGG
YKMSGIGREKGIYSLHNYLQVKAVVTPLKNPAWL
>PtALDH2B7
MAAKKISTVLSRSFSSAAAPPCFFSRVRGGGGQSRLISRYNTTLAAVEDPITPPVSVKYNQLLINGQFVD
AASGKTFPTLDPRTGEVIAHVAEGDVEDVNRAVSAARKAFDEGPWPRMTAYERSRIIWRFADLLEKHTDE
IAALETWDNGKPYEQSAKIEIPMTVRIFRYYAGWADKIHGLTVPADGPYHVQTLHEPIGVAGQIIPWNFP
MLMFSWKVGPALACGNTVVIKTAEQTPLSAVYAAKLFHEAGLPDGVLNVVSGFGPTAGAALASHMDVDKL
AFTGSTDTGKIVLELASKSNLKPVTLELGGKSPFIVCEDADVDQAVELSHSAVFFNQGQCCCAGSRTFVH
ERVYDEFVEKAKARANQRAVGDPFKEGIEQGPQVDSDQFEKILRIIRSGVESGANLKAGGDRFGTTGYYI
QPTVFSDVQDDMLIAKEEIFGPVQSIFKFKDLDEVIQRSNNSRYGLAAGIFTHNLDTANTLSRALKVGTV
WINCYDVFDAAIPFGGYKMSGNGREKGIYSLNNYLQVKAVVTSLKNPAWL
>PtALDH2C4
MMKYADLIDEHIEELAALDAIDAGKLFSGGKAVDIPNVARLLRYYAGAADKIHGEVLKMSRELHGYTLRE
PIGVSGHIIPWNFPSSMFFMMSAPALAAGCTMIVKPAEQTPLSALFYGHLAKQAGMPDGVINVITGYGPT
AGAAIASHMDVDKVCFTGSTEVGRKIMQAAATSNLKQVSLELGGKSPLLIFDDADVDKAADLALLGILYN
KGEICVASSRVFVQEGIYDEFVKKLKEKAKDWVVGDPFDPRSRLGPQVDKQQFDKILSYIEHGKREGASL
LTGGKPVGKKGYFIEPTVFTDVKEDMMIATDEIFGPVMSLMKFKTIDEAIKKANNTKYGLAAGIVTKNLD
VANTVSRSIRAGTIWINCYFAFDNDCSYGGYKMSGFGRHLGMEALHKFLQVKSVVTPIYNSPWL
>PtALDH3F1

MEGLEGTLAELRDTFKSGRTRSVAWRKSQLRAMIEFVQDNEEEMFKVLDQDLGKHPVEAYRDEVGVVAKS
AKLSLSCVEKWMAPKKGNLPLAFFPASAEVMPEPFGVVLIMGSWNFPISLTLDPLIGAISAGNVVVLKPS
ELSPACSSFLAEAIPKYLDPKSIKVIEGGIDVCEQLLQQNWDKIFFTGSQRVGRIVMTAAAQHLTPVTLE
LGGKSPAILDSSSNPTNMKVIAKRIVAAKWGSCSGQACIAIDYMLVEEKFASYLIDLLEKTIKQFFGENP
RESKSLCKILNKNNFMRLLDLLKDPLIRASVVYGGSVDEETMYIEPTILLNPPLDSQIMTEEIFGPLLPI
ITLNNIHDSIEFISSRPKPLAIYAFTRDETFKKQILSKTSSGSVTFNDTLLQFVCDSLPFGGVGQSGFGR
YHGKYSFDTFSHEKAILQRRFFPELEPRYPPWNNLKFQFIKLLYAFNYIGLLLLLLGLKK
>PtALDH3F2
MSSKKAKLPRVALLSSAELVPEPLGFVLIISSWNFPFGLSLEPMIGAIAAGNTMVLKPSELAPASASLLA
NVLPTYLDNSAVKVIQGGPAVGERLLQQKWDKIFFTGSARVGRIIMSAAVKHLTPVALELGGKCPAVVDS
VSSSWDTKVTVNRILVSKFGACAGQACIAIDYILVEKRFASILVELMKVMIKKMFGENPRETNTVARIVN
EQHFLRLKNLLSDSAVQNSIVYGGSMDEKNLFVEPTILVDPPLDAAIMTEEIFGPLLPIITLDKVEDSIA
FINSKPKPLAIYAFTNNEKFRRRMLSETSSGSLVFNDAVIQYAADALPFGGIGESGIGKYHGKFSFDTFS
HYKAVTRRSFLTDFWFRFPPWNDYKLLLLEATYNYDYLGMLLVILGLKRRR
>PtALDH3H1
MATEEEKQMVFDVEAANMLTKELRDVFASGKTRSYEWRISQLKSMIKMCDEHEEDIVDALHQDLSKPKLE
SIVYEITMLKNSCTLAIKELKQWMMPEKAKTSLLTFPSSAEIVPEPLGVVLIISAWNYPFLLSLDPLVGA
IAAGNAMVLKPSEFSPATSSLLAKLLPEYLDISSIKVVEGAVSETSALLEQKWDKIFYTGNGIVGRIVMA
AAAKHLTPVVLELGGKSPAVVDSAIDLQIATRRLIAGKWGCNNGQACVSPDYIITTKDCADKLVDSLKKE
LETFYGKNPLESKDLSRIVNSKHFSRLTKLLDEDKVSRKIVYGGERDEANLKISPTILVDVPCDSLIMKE
EIFGPLLPILIVSKIEDSFDMINSGTKPLAAYLFTNNKKLKEQFVMSVSAGGVVINDIAMHLAIHTLPFG
GVGESGTGSYHGKFSFDAFSHKKAVLYRSFMGDAALRYPPYTRGKLRLMKAFMTSNFWTILRALFGRS
>PtALDH3H2
MATKEEENTVFDVEAANVLTKELRDVFASGKTRSYEWRISQLKSIVKMCDEHEEDIVDALRQDLSKPQLE
SIVYELTMVKNSCTLAIKELKHWMMPEKAKTSLTTFPSSAEIVSEPLGAVLIISAWNYPFLLSMDPLIGA
IAAGNAMVLKPSEVAPATSSLLAKLLPEYLDCSSIKVVEGAVSETSALLEQKWDKIFYTGNGRVGRIVMA
AAAKHLTPVVLELGGKSPVVVDSGIDIQIATRRIIVGKWGCNNGQACISPDYIITTKDCAEKLVDSLKKE
LEAFYGKNPLESKDLSRIVNSNHFSRLTKLLDEDKVSGKIVYGGERDEANLRIAPTILLGVPQNSLIMKE
EIFGPLLPILTVSKIEDSFDIIKSGTKPLAAYLFTNNKKLKEQFLMSVSAGGVVINDTTLHLAVHSVPFG
GVGESGMGSYHGKFSFDAFTHKKAVVYRSFVGDASVRYPPYTLGKLRLMKALITGNVWTILRTLLGMS
>PtALDH3I1
MRSLCVEPFQNLSVVDTGARRAFTHCSPWKTNHKHEAVLSFPLSSPIRKSLCICLSSSANLPVMMEEKKQ
TFDANEAAWLVKELNESFRTGKTKSYEWRVSQLKGIEKMVVEREKDICEALYKDLSKPEYEAFVSEIAMV
KSSCEEALKELKQWMKPEKAKTSMATYPSSAEIVSEPLGAVLVISTWNYPFSLSVKPVIGAITAGNAVVL
KPSEIAPATSSLLSKLFEEYLDRSAVRVLEGGVLETTALLDQKWDKIFYTGSPRVGRIVMTAAAKHLTPV
VLELGGKCPAVIDSDVDLQVTVRRIIAGKWQLNNGQACISVDYIITTKEFAPKLIDALRKGIEEFFGTDP
MESKDISCIVSSNHFSRLESLMDDYKVFNKIVVGGQRNQKKLKIAPTIFLDVPGDSQLMQEEIFGPLLPI
ITVENVKDSIDLINSKPKPLTAYLFTNNEKLKNNFVQSVSSGGMVINDTVLHVTVSSLPFGGVGESGMGS
YHGKFSFDAFSHKKAVLYRSFSGDASVRYPPYTPEKQKLIRAVMNGGIFDIIILALMGF
>PtALDH3I2
MTEGKKQPFDANEAPSLVKELKESFRTGRTRSYEWRVSQLKGIEKMVEEREKDISEALYKDLSKPEFEAF
VSEIAAVKSSCEEALKELKQWMKPEKAKTSMTAYPSSAEIVSEPLGAVLVISTWNYPFLLSIDPVIGAIA
AGNAVVLKPSEIAPVTSSLLSELFEEYLDSSAVRVVEGAVPETAALLEQKWDKIFYTGSPRVGRIVMTAA
AKHLTPVVLELGGKCPVVVDSDVDLQVTARRIIAGKWQLNNGQACISVDYIIATKDFAPKLIDALRNGIE
EFFGADPMESKYISRIVSSNHFSRLERLLDEYKVFNKIVVGGQRNQKKLKIAPTIFLDVPEDSQLMQEEI
FGPLLPIITVENVKDSIDLINSKPEPLVAYLFTNNQKLRNDFVQNVSCGGMVINDTVLHVTVSSLPFGGV
GESGMGSYHGKFSFDAFSHKKAVLYRSFSGDSPVRYPPYTPENKKLMRAVMNGGIFDIIILALMGWSRD
>PtALDH5F1

MTLGRIALARIPATRCKIHSLFSAPHSSSSTNRTSPPLSRHMSMKSENLVSKLTSSGLLKTQGLIDGKWV
DANDGDTIKVLNPATGEVVAIVPCMGQSETNNAISSAYDAFRSWSKLTASERSQRIRKWYDLLIAHKEEL
GQLITLEQGKPLKEAMGEVSYGASFIEFYAEEAKRVYGDIIPATLGDRRLFVLKQPVGVVGAITPWNFPL
AMITRKVGPALACGCTVVLKPSELTPLTALAAAELALQAGIPPGVLNVVMGKAPDIGDALLASHEVRKIT
FTGSTAVGKKLMAGAAGTVKRLSLELGGNAPCIVFDDADLDVAVKGSLAAKFRNSGQTCVCANRIIVQEG
IYDKFADSFSKAVQSMQVGDGFSEGVTQGPLINEAAVQKVESFVQDAIFKGAKVLLGGKRHSLGMNFYEP
TIISNVTEAMLLSREEVFGPVAPLLRFKTEEEAILMANNTKAGLAAYIFTNNVQRSWRVTEALEYGLVGV
NEGLISTEVAPFGGVKQSGLGREGSKYGMDEYLEMKYVCLGDMNRK
>PtALDH6B1
MLLLRSSIQRARNLKALKPSIFALRSSYCFSTGAAEPSSSLPSPPRVPNLIGGKFVDSQSSSTIDVINPA
TQEAVSRVPFTTNEEFRAAVSAAKQAFPAWRNTPITTRQRVMLKLQELIRRDIDKLAMNITTEQGKTLKD
AHGDVFRGLEVVEHACGMATLQMGEYVPNVSNGIDTFSIREPLGVCAGICPFNFPAMIPLWMFPVAVTCG
NTFILKPSEKDPGASIILAELAMEAGLPDGVLNIVHGTNDVVNAICDDDDIRAISFVGSNTAGMHIYSRA
SAKGKRVQSNMGAKNHAIVLPDANVDATLNALVAAGFGAAGQRCMALSTVVFVGDPESWENKLVERAKSL
KVNSGMEPDADLGPVISKQAKERVCRLIQSGVESGARLLLDGRNIVVPGFEHGNFIGPTILSGVTADMEC
YKEEIFGPVLLCMEAGSVEEAINILNRNKYGNGAAIFTASGAAARKFQTEIEAGQVGINVPIPVPLPFFS
FTGSKASFAGDLNFYGKAGVNFYTQIKTITQQWKDLPGGSGVSLAMPTSQKL
>PtALDH6B2
MLLLRSSIRRARNLKALKPSIFALRSSYCFSTGAAEPSSSLPSPPRVPNLIGGKFVDSQSSSTIDVINPA
TQEAVSPVPLTTNEEFRAAVSAAKQAFPAWRNTPITTRQRVMLKLQELIRRDIDKLAMNITTEQGKTLKD
AHGDVFRGLEVVEHACGMATLQMGEYVPNVSNGIDTFSIREPLGVCAGICPFNFPAMIPLWMFPVAVTCG
NTFILKPSEKDPGKDYLVYILCIIFGALSLQGTSVC
>PtALDH6B3
MLPLKISIQRARNLKALKPSIFALRSSYYFSTGVVEPSSSLRSPPRVPNLIGGKFVDSQSSSTIDVINPA
TQEVVSQIPLTTNEEFKAAVSAAKHAFPAWRNTPITTRQRVMLKLQELIRRDTDKLAMNITTEQGKTLKD
AHGDVFRGLEVVEHACGMATLQMGEYVPNVANGIDTFSIREPLGVCAGICPFNFPAMIPLWMFPVAVTCG
NTFVLKPSEKDPGASIILAELAMEAGLPNGVLNIVHGTNDIVNAICDDDDIRAISFVGSNTAGMHIYSRA
SAKGKRVQSNMGAKNHAIVLPDANTDATLNALVAAGFGAAGQRCMALSTVVFVGDSQSWENKLVECAKSL
KVNAGTEPDADLGPVISKQAKERVCKLIESGVESGARLLLDGRNIVVPGYEDGNFIGPTILSGVTADMDC
YKEEIFGPVLLCKEADSFEEAIHFVNRNKYGNGAAIFTTSGAAARKFQTEIEAGQVGINVPIPVPLPFFS
FTGSKASFAGDLNFYGKAGVNFYTQIKTITQQWKDLPGGSGVSLAMPTSQKL
>PtALDH6B4
MTDIQSSSGSGLDEAQMQMQPPPPGTFVDREELIQHVGDFAVSQGYVVTIKQSKRERVVVLGCDRGGVYR
NRKKADEETSAERKRRKRSGSRLTNCPFEAVGKKDDGLWVLTIKNGTHNHEPLKDITEHPSARRFSESEI
VLIKEMTEAGLKPRQILKRLRQSNPELLSTPKHVYNVKAKLRQGNMTGRNFKSLRPEKSAGRDKHLSIAE
PSWRQRYPMRVPNFIGGRLVNSQSFASIDVINPATQQVVSQVPLTTNEEFRAAVFAAKRAFPQWRDTPIT
TRQRIMFKFQELIRRDIDKLAMSITTEHGKTLKDAHGDVLRGLEVVEHACGLASLQIGEFVSNISSGIDT
YSIREPLGVCAGICPFEFPAMIPLWIFPIAVTCGNTFILKPSEKDPGASVMLAELAMEAGLPNGVLNIVH
GTNEIINGICDDDDIKAISFVGPNAVGAYVYARASAKGKRTQSNIGAKNHAVVMPDASVGATINALVAAG
FGGAGQKCMALNMAVFVGGLGPWEEKLVEHAKALKVTSGTEPDAELGPVISKQEKERIITLIQTGVESGA
KLVLDGRNIVVAGYENGNFIGPTILSDVTVNMECYKEDIFGPVLLCMQADSIEEAINIVNGNKYSNGASI
FTTSGVAARKFQTEVEVGQVGINVPISVPLPFSSFISAKPSFAGDVSFDGKAGIQFYTQVKTVTQQWRDL
VSDDSSSHQLPSS
>PtALDH6B5
MWVLNIKNGEHNHEPLKDMSEHPYSRRFSEEEVRQIRMMTEAGVKPRQVLKALKQSNPELQSTPRHLYNL
KAKIRQGGLSDRSLKSWRPNRSVLVNTSASSTGESLKEDRQPMKVPNFIGGKFVVSQGCTIIDVLNPATQ
EVVSHLPLTTYEEFKDAVIAAKRAFPSWKNMPIATRQRVMFRFQELIRRDMDKLATSITSEQGKTLKGAL
GDVLCGLEAVEHACAMATLQMGEFVPNASNGIDTYCIREPLGVCAGICPFNFPAMIPLWMFPIAVTCGNT

FVLKPCEKNPGASMILAALAVEAGFPDGVLNVIHGTNDIVNYICDDDDVKAISFIGSDLAGLHIYARAAA
RGKRVQSNIGGKNHAIILPDASIDDTLNALVAAGFGAAGQRCMALSTAVFVGGSSAWEHELVEHAKALKV
NAGTDPSADLGPVISKEVKDRICRLVQSGVDSGARLLLDGRNIVVPGYENGSFVGPTILCDVTISMECYK
EEILGPVLLCMQADSLEEAITIVNRNRYGNGASIFTTSGVAARKFQNDIDAVLVGINVSVPVPLPCSSFH
EAKVSFAGNLNFCGKTGVQFYTQIKTVAQQWRELPSIGVSLSMHTSNEMEMTSRGVCSALPPSERDSPGK
TVSPAMSLAPERDPQKHRELLCENLPKSGGSSVPSITDKDLHNQEASLVLPPTAEKDLQAKIPPTIPHAS
EIKLSSQEISLTTCQTSEGMYIPVPSQWNETPTLTSQRTESISQISQRIYLPTSQRRNNAAPSLKRIDAA
MDLTSECVYMATPRQNDNTGPALLKDSSPSPTSRPTDTAAHPASERLHDITTSHLSDSMVQSFQRNDHMF
PTERKYTSAAAHRNDHIGLTSQRPDVASYPSSERVYSSATSQRTDNMIPASQRAEAMPPTTKTMYMPPIV
QRNNGPQKTSERLFMYQSERMYSESTLISIDGFSSQGVSMTLATSQRM
>PtALDH7B3
MSFARKEYEFLSEIGLSSRNLGCYVDGTWKANGPVVTSVNPANNQAIAEVVEGSVEDYEEGMRACSEAAK
IWMQVPSPKRGEIVRQIGDALRTKLQELGRLVSLEMGKILPEGIGEVQEIIDMCDFCVGLSRQLNGSVIP
SERPNHAMLEMWNPLGIVGVITAFNFPCAVLGWNACIALVCGNCVVWKGAPTTPLITIAMTRLVAGVLEK
NNLPPAIFTSFCGGADIGQAIAKDTRISLVSFTGSSKVGLMLQQTVNQRFGKCLLELSGNNAIIIMDDAD
IQLAVHSVLFAAVGTAGQRCTTCRRLLLHESIYQRVLDQLLDVYKQVKIGNPLEKGNLLGPLHTSESRKS
FERGIEIIKSQACKILIGGSVIESEGNFVQPTIVEISPNADVVKEELFAPVLYVMKFQTLQEAIEINNSV
PQGLSSSIFTRKPEIIFKWIGPLGSDCGIVNVNIPTNGAEIGGAFGGEKATGGGREAGSDSWKQYMRRST
CTINYGNELPLAQGINFG
>PtALDH7B4
MGFARKEYEFLSEIGLSSRNLGCYVDGTWKANGPVVTSVNPANNQAIAEVVEGSIEDYEEGMRACSEAAK
IWMQVPSPKRGEIVRQIGDALRTKLQQLGRLVSLEMGKILPEGIGEVQEIIDMCDFSVGLSRQLNGSVIP
SERPNHAMLEMWNPLGIVGVITAFNFPCAVLGWNACIALVCGNCVVWKGAPTTPLITIAMTRLVAGVLEK
NNLPPAIFTSFCGGADIGQAVAKDTRIPLVSFTGSSKVGLMVQQIVNQRFGKCLLELSGNNAIIVMDDAN
IQLAVRSVMFAAVGTAGQRCTTCRRLLLHESIYQRVLDQLLDVYKQVKIGDPLEKGTLLGPLHTSESRKS
FEKGIEIIKSQGGKIITGGSVIESEGNFVQPTIVEISPNADVVKEELFAPVLYVMKFQTLQEAIEINNSV
PQGLSSSIFTRQPGVIFKWIGPQGSDCGIVNVNIPTNGAEIGGAFGGEKATGGGREAGSDSWKQYMRRST
CTINYGNELPLAQGINFG
>PtALDH10A8
MAIHLPNRQLFIDGEWRETVLKKRIPVINPATEQIIGDIPAATAEDVEIAVEAAKKAFSRNKGKDWSSAS
GAYRARYLRAIAAKITERKSELGKLEAIDSGKPLDEALWDMDDVAGCFEYYADLAEGLDTKQKAPVSLPM
ETFKSFVLKEPLGVVALITPWNYPLLLATWKVAPALAAGCTAILKPSELASVTCLELGEVCREVGLPPGV
LNILTGLGTEAGAPLASHPHVDKVAFTGSTATGSRIMASAAQMVKPVSMELGGKSPIIVFEDVDLDKAAE
WTLFGCFWTNGQICSATSRLLVHESIASEFLDKLVKWTKKIKISDPFEEGCRLGPLVSGEQYDKILKFIA
TAKSEGATILSGGDRPKHLNKGFFVEPTIIIDVTTSMQIWREEVFGPVLCVKTFSTEDEAIDLANDTHYG
LGAAVISNDPERCDRVAKAFRAGIVWINCSQPCFCQAPWGGIKRSGFGRELGEWGLENYLSVKQVTRYIS
EEPWGWYQAPSKL
>PtALDH10A9
MAIHLPIRQLFIDGEWREPVLKKRIPVINPATEQIVGDIPAATAEDVEIAVEAARKAFSRNKGQDWPSTS
GAYRAKYLRAIAAKITEKKSELGKLEVIDCGKPLDEALWDMDDVAGCFEYYADLAEGLDAKQKAPVSLPM
ETFKSYVLKEPLGVVALITPWNYPLLMGAWKVAPALAAGCTAILKPSELASVTCLELAEVCREVGLPPGV
LNILTGLGTEAGAPLASHPHVDKVAFTGSSATGSKIMASAAQMVKPVSMELGGKSPIIVFEDVDLDKAVE
WTLFGCFWTNGQICSATSRLLVHESIASEFLDRLVKWTKKIKISDPFEEGCRLGPVVSGGQYEKVLEFIA
TARSEGATILSGGDRPKHFTKGFFVEPTIITDVTTSMQIWREEVFGPVLCVKTFSTEDEAIELANDTHYG
LGAAVISNDLERCDRVTKVRHELTSNLVQITHQNLLIGIVYLLRQYFSFAPVLQAFRAGIVWINCSQPCF
CQAPWGGIKRSGFGRELGEWGLENYLSVKQVTQYISDEPWGWYQSPAKL
>PtALDH11A1

MAGTGMFSEILDGDLYKYYSDGEWKKSSSGKTVSIVNPTTRKTQYKVQACTQEEVNKVMESAKSAQKAWA
KTPLWKRAELLHKAAAILKEHKAPIAECLIKEIAKPAKDSVTEVVRSGDLISYTAEEGVRILGEGKFLVS
DSFPGNDRTKYCLTSKIPLGVVLAIPPFNYPVNLAVSKIGPALIAGNSLVLKPPTQGAVSCLHMVHCFHL
AGFPKGLISCVTGKGSEIGDFLTMHPGVNCISFTGGDTGIAISKKAGMIPLQMELGGKDACIVLEDADLD
LVAANIIKGGFSYSGQRCTAIKVVLVMESVADALVEKVKARVAKLRVGPPENDCDITPVVTESSANFIEG
LVMDAKEKGATFCQQYKREGNLIWPLLLDNVRPDMRIAWEEPFGPILPVVRINSVEEGIYHCNASNFGLQ
GCVFTKDINRAMLISDAMETGTVQINSAPARGPDHFPFQGLKDSGIGSQGITNSINMMTKVKTTVINLPS
PSYTMGYC
>PtALDH11A2
MAGTGVFSEILDGDAYKYYSDGEWKKSSSGKTVAIVNPTTRKTQYKVQACNQEEVNKVMELAKSAQKTWA
KTPLWKRAELLHKAAAILKEHKAPIAECLIKEIAKPAKDSVTEVVRSGDLISYTAEEGVRILGEGKFLVS
DSFPGNERTKYCLTSKIPLGVVLAIPPFNYPVNLAVSKIGPALIAGNSLVLKPPTQGAVSCLHMVHCFHL
AGFPKGLISCVTGKGSEIGDFLTMHPGVNCISFTGGDTGISISKKAGMIPLQMELGGKDACIVLEDADLD
LVAANIIKGGFSYSGQRCTAIKVVLVMESVADALVEKVKARVAKLRVGPPEDDCDITPVVTESSANFIEG
LVTDAKEKGATFCQQYKREGNLIWPLLLDNVRPDMRIAWEEPFGPILPVIRINSVEEGIHHCNASNFGLQ
GCVFTKDINKAVLISDAMETGTVQINSAPARGPDHFPFQGLKDSGIGSQGITNSIDMMTKVKTTVINLPS
PSYSMGSSGSSIRSRI
>PtALDH11A3
MAGTSVFAELVDEDTTVFKFYSDGEWKKSTSGKLVSIINPTTRKTQYKVQACTQEEVNKIIEAAKTAQKS
WAKTPLWKRAELLHKAAAILKEHRAPIAECLVKEIAKPAKDAVTEVVRSGDLVSYCAEEGVRILGEGKFL
VSDSFPGNERTKYCLTSKIPLGVVLAIPPFNYPVNLAVSKIAPALIAGNSIVLKPPTQGAVAALHMVHCF
HLAGFPKGLVSCVTGKGSEIGDFLTMHPGVSCISFTGGDTGIAISKKAGMIPLQMELGGKDACIILEDGD
LDLAAANIIKGGFSYSGQRCTAVKVILIMESVADTLVEKVKAKVAKLTVGPPEDDCDITPVVTESSANFI
EGLVMDAKQKGATFCQEYKREGNLIWPLLLDNVRPDMRIAWEEPFGPVLPVIRINSIEEAIYHSNASNFG
LQGCIFTRDINKAILISDAMETGTVQINSAPARGPDHFPFQGLKDSGIGSQGITNSINMMTKIKSTVINL
PAPSYAMG
>PtALDH12A1
MMYGFLVCRASQKATRNWLSSFNLSRSVHSLPFATVDAEGISGSQPAKVHNLVQGKWIGSSTWNTIVDPL
NGEPFIKIAEVDETGTQPFVESLSKCPKHGLHNPFKSPERYLLYGDITAKAAHMLAVPKVSDFFTRLIQR
VAPKSYQQALGEVQVTQKFLENFSGDQVRFLARSFAVPGNHLGQQSHGFRWPYGPVAIITPFNFPLEIPL
LQLMGALYMGNKPILKVDSKVCIVMEQMIRLLHHCGMPLSDVDFINSDGKTMNKLLLEANPQMTLFTGSS
KVAEKLAVDLKGRIKLEDAGFDWKILGPDVNEVDYIAWVCDQDAYACSGQKCSAQSILFMHENWSATSLI
SKMKDLAERRKLEDLTIGPVLTLTTEAMLDHMNKLLQIPGSKLLFGGKPLENHSIPSIYGALKPTAIYVP
LEEILRAKNYELVTREIFGPFQVITEYKKDQLPMVLDALERMHAHLTAAVVSNDVLFLQARLENIFASHP
TPLPIEVIGKTVNGTTYAGLRARTTGAPQNHWFGPAGDPRGAGIGTPEAIKLVWSCHREVIYDFGPLPKL
WEIPPST
>PtALDH18B1
MSDLDRSRAFFNDVKRLIIKVGTAVVTRADGRLALGRLGALCEQIKDLSSLGYEVIVVTSGAVGLGRQRL
KYRRFVNSSFSDLQKPQVDLDGKACAAVGQNSLMALYDTMFSQLDVTSAQLLVTDRDFKNKDFRKQLDET
VQSLLALRVIPIFNENDAVSTRKAPYEDSSGIFWDNDSLAALLALELKADLLVLLSDVEGLYSGPPSDPR
SKLIHTYIKEIHQSEITFGDKSRVGRGGMTAKVKAAVNAAYAGIPVVITSGYAPENIIKVLQGERVGTLF
HQDAHLWALDKEVGGREMAVAARESSRRLQALSSQDRNKILLDVADALEANEKLINIENEADVVAAQEAG
LEKSLISRLALKPGKIKSLANTIRVLANMDDPIGCILKRNELADGLVLEKTSSPLGVLLIIFESRPDALV
QIASLAIRSGNGLLLKGGKEAKRSNAILHKVITTAIPDTVGGKLIGLVTSIDEIPDLLKLDDVIDLVIPR
GSSKLVSKIKSSTKIPVLGHADGICHVYVDKSANIEMAKRVVLDAKVDYPAACNAMETLLVHQDLVHSGG
LNELIADLRTEGVTLFGGQRACKELNIPEAHTFHHEYNSMACTVEIVDDEHAAIDHIHQHGSAHTDCIVA
EDHDVAEVFLRQVDSAAVFHNASTRFCDGARFGLGAEVGISTSRIHARGPVGVEGLLTTRWILRGCGQVV
NADQGVIYTHKDITM

>PtALDH18B2
MNGTDPSRGFFKDVKRLIIKVGTAVVTRTDGRLALGRLGALCEQIKDLNSLGYEVIVVTSGAVGLGRQRL
KYRRLVNSSFADLQKPQVDFDGKACAAVGQNNLMALYDTLFSQLDVTSAQLLVTDSDFRDKGFRKQLDQT
VKSLLALRVIPIFNENDAVSTRRAPYEDSSGIFWDNDSLAALLALELKADLLVLLSDVEGLYSGPPSDPQ
SKLIHTYIKEIHQSEITFGDKSRVGRGGMTAKVKAAVNAAYAGIPVVITSGYAPENIMKVLQGERVGTLF
HQDAHLWVPVKEVSGREMAVAARESSRRLQALSSQDRKKILLGVADALEANEKLIKIENEADVAAAQQAG
LEKSLISRLALKPGKIESLANSIRVLANMEDPIGRVLKRTELADGLLLEKTSSPLGVLLIVFESRPDALV
QIASLAIRSGNGLLLKGGKEAKRSNAILHKVITTAIPDTVGGRLIGLVTSRDEIPDLLKLDDVIDLVIPR
GSNKLVSQIKSSTKIPVLGHADGICHVYMDKSANMEMAKRVVLDAKIDYPAACNAMETLLVHQDLVQTAG
LNELIVDLRTEGVTLFGGQRACKELNLPEAHSLHHEYNSMACTVEIVDDVHAAINHIHQHGSAHTDCIIA
EDQDVAEVFLCQVDSAAVFHNASTRFCDGARFGLGAEVGISTSRIHARGPVGVEGLLTTKWILRGSGQVV
NGDKGVIYTHKDMTLQSVD
>PtALDH22A1
MAFWWPLIVAASAYAICRFLLMLIPFNVPSIDVDASDVTEGNQTQENSFIYIPPRGRAQQSDKKVQCYEP
ATMKYLGFFPALSPAEVHDRVAQARKAQKIWAESSFKQRRQFLRILLKYIIEHQELICEVSSRDTGKTMV
DASLGEIMTTCEKITWLLSEGEKWLKPEYRCSGRAMFYKKSRVEFHPLGVIGAIVSWNYPFHNIFNPMLA
AVFSGNSIVIKVSENASWSGLFYFRIIQAALAAVGAPENLVDVITGFAETGEALVSSVDKIIFVGSPGVG
KMIMRNASDTLIPVTLELGGKDPFIVCEDADVSHVAQIAVRAVLQSSGQNCAGAERFYVHRDIYSSFVSE
VTKIVKSVSVGPPLAGRYDMGAICLQEHSDKLQILVNDALEKGAEIVVRGSFGHLGEGAVDQFYPPTVLV
NVDHTMKLMQEETFGPIMPIMKFSTDEEAVKLANDSRYGLGCAVFSGSQRRAREIASQIHCGVAAVNDFA
SNYMCQSLPFGGVKHSGFGRFAGVEGLRACCLVKSVVEDRLWPYIKTKIPKPIQYPVGENSFEFQQSLVE
ALYGLNIRDKLRAGVNVLKIMSEQNSSNSKSRNE
>ZmALDH2B2
MARRAASSLVSRCLLARAPAGAPPAAPSAPRRTVPADGMHRLLPGVLQRFSTAAAVEEPITPSVHVNYTK
LLINGNFVDSASG
KTFPTLDPRTGEVIAHVAEGDAEDINRAVAAARKAFDEGPWPKMTAYERSRILLRFADLIEKHNDELAAL
ETWDNGKPYEQAAQIEVPMVARLMRYYAGW
ADKIHGLIVPADGPHHVQILHEPIGVAGQIIPWNFPLLMYAWKVGPALACGNTLVLKTAEQTPLSALYIS
KLLHEAGLPEGVVNVVSGFGPTAGAALASH
MDVDKIAFTGSTDTGKIILELAAKSNLKTVTLELGGKSPFIIMDDADVDHAVELAHFALFFNQGQCCCAG
SRTFVHERVYDEFVEKAKARALKRVVGDPF
RKGVEQGPQIDDEQFNKILRYIRYGVDGGATLVTGGDRLGDKGFYIQPTIFSDVQDGMKIAQEEIFGPVQ
SILKFKDLNEVIKRANASQYGLAAGVFTNS
LDTANTLTRALRAGTVWVNCFDVFDAAIPFGGYKMSGIGREKGVDSLKNYLQVKAVVTPIKNAAWL
>ZmALDH2B5
MAATVRRAASSVLSRFLLTKPSPSPASAAGNKSALLGAGAAALHRFSTAPASAAAAAEEPIQPAVEVKHT
QLLINGNFVDAAS
GKTFPTLDPRTGEVIARVAEGDSEDIDRAVAAARRAFDEGPWPRMTAYERCRVLLRFADLIERHAEEVAA
LETWDNGKTLAQAAGAEVPMVARCVRYYAG
WADKIHGLVAPADGAHHVQVLHEPVGVAGQIIPWNFPLLMFAWKVGPALACGNTVVLKTAEQTPLSALYV
ANLLHEAGLPEGVLNVVSGFGPTAGAALCS
HMGVDKLAFTGSTGTGQIVLELAARSNLKPVTLELGGKSPFIVMDDADVDQAVELAHQAVFFNQGQCCCA
GSRTFVHERVYDEFVEKSKARALKRVVGDP
FRDGVEQGPQIDGEQFNKILRYVQSGVDSGATLVAGGDRVGDRGFYIQPTVFADAKDEMKIAREEIFGPV
QTILKFSGVEEVIRRANATPYGLAAGVFTR
SLDAANTLSRALRAGTVWVNCYDVFDATIPFGGYKMSGVGREKGIYALRNYLQTKAVVTPIKNPAWL
>ZmALDH2C1

MATANGSSKGPFEVPKVEVRFTKLFIDGKFVDAVSGKTFETRDPRTGEVIASIAEGGKADVDLAVKAARE
AFDNGPWPRMTGY
ERGRILHRFADLIDEHVEELAALDTVDAGKLFAVGKARDIPGAAHLLRYYAGAADKVHGATLKMAQRMHG
YTLKEPVGVVGHIVPWNYPTTMFFFKVGPA
LAAGCAVVVKPAEQTPLSALFYAHLAREAGVPAGVLNVVPGFGPTAGAAVAAHMDVDKVSFTGSTEVGRL
VMRAAAESNLKPVSLELGGKSPVIVFDDAD
LDMAVNLVNFATYTNKGEICVAGTRIYVQEGIYDEFVKKAAELASKSVVGDPFNPSVSQGPQVDKDQYEK
VLRYIDIGKREGATLVTGGKPCGDKGYYIE
PTIFTDVKDDMTIAQDEIFGPVMALMKFKTVEEVIQKANNTRYGLAAGIVTKNIDVANTVSRSIRAGAIW
INCYFAFDPDAPFGGYKMSGFGKDMGMDAL
DKYLQTKTVVTPLYNTPWL
>ZmALDH2C2
MASNGCNGNGNGNGNGKAAPAGVVVPEIKFTKLFINGEFVDAASGKTFDTRDPRTGDVLAHVAEADKADV
DLAVKSARDAFEH
GKWPRMSGYERGRIMSKLADLVEQHTEELAALDGADAGKLLLLGKIIDIPAATQMLRYYAGAADKIHGDV
LRVSGRYQGYTLKEPIGVVGVIIPWNFPTM
MFFLKVSPALAAGCTVVVKPAEQTPLSALYYAHLAKMAGVPDGVINVVPGFGPTAGAALASHMDVDSVAF
TGSTEVGRLIMESAARSNLKTVSLELGGKS
PLIIFDDADVDMAVNLSRLAVFFNKGEVCVAGSRVYVQEGIYDEFVKKAVEAARSWKVGDPFDVTSNMGP
QVDKDQFERVLKYIEHGKSEGATLLTGGKP
AADKGYYIEPTIFVDVTEDMKIAQEEIFGPVMSLMKFKTVDEVIEKANCTRYGLAAGIVTKSLDVANRVS
RSVRAGTVWVNCYFAFDPDAPFGGYKMSGF
GRDQGLAAMDKYLQVKSVITALPDSPWY
>ZmALDH2C4
MASNGNGDGTARVVVPEIKFTKLFINGEFVDAASGKTFETRDPRTGDVLAHVAEADQADVDLAVKSARDA
FDHGKWPRMSGYE
RGRVMSKLADLVEQHTEELAALDGADAGKLLLLGKMIDIPAATQMLRYYAGAADKIHGDVLRVSGKYQGY
TLKEPIGVRRYLSS
>ZmALDH2C5
MVSESNRGGADRTTAAGEERGQLLFDVPEIRFTKLFINGSFVDAVSGRTFETRDPRTGGVIASVAEADKE
DVDLAVRAARAAF
DHGEWPRMSGSERGRIMARLADLVEERADELAALESLDAGKHPAVTRAVDVGNAAGSLRYFAGAADKIHG
ETLKMPGQFQGHTLREPLGVAGVIIPWNFP
STMFAVKVAPALAAGCALVVKPAEQTPLSALYLAQLAKQAGVPDGVINVVPGFGPTAGAALASHMDVDMV
SFTGSTEVGRLIMKASAESNLKPVYLELGG
KSPLIVFDDADLDMAVELAVGASFFNKGEACVAASRVYVQERVYDRFEERLAERMRSWVVGDPFSDPSAD
QGPQVDKAQYERVLSYIDHGKREGATLLTG
GRPCGPEGKGYYIEPTVFTNVKEDMIIAKEEIFGPVMCLMKFKTVEEAIARANDTRYGLGAGVVTRDLDV
ANRVVRSVRAGVVWVNCYFAMGSDCPFGGR
KMSGFGKDEGMHALDKYLAVKSVVTPLRASPWI
>ZmALDH3E1
MGSVPEEKAKLGFGGLVGDLREVYESGRTQGLEWRQSQLRGLVRLLEEKEEEIFDVLHEDLGKHRGEAFR
DEVGVLKKSVVDK
LQNLKNWAAPEKAHTPLVAFPATALVVPEPLGVVLVFSCWNLPIGLALEPLSGALAAGNAVVVKPSELAP
ATSAFLAANIPKYLDSKAVKVVEGGPEVGE
KLMEHRWDKVLFTGSSRVGRLIMAQAAKHLTPVALELGSKCPCIVDWLDSDRDSQVAVNRIIGAKWSTCS
GQACIAIDYLLVEEEFAPILIEMLKSTLER

FFTKPEYMARILNEKQFQRLSGFLADRRVASSVVHGGHFNPKTLSMEPTLLLNPPLDSDIMTEEIFGPLL
PIITVKKIEDSIKFLRSKPKPLAIYAFTRN
EKLKQRIIDETSSGSITFNDAIVQYGLDSIPFGGVGHSGFGQYHGKYSFDMFSHKKAVLKRSFLVEFMFR
YPPWDETKIGMLRRVYRFDYVSLFLALIGL
RR
>ZmALDH3E2
MGRTEAADDGAESGGLGLGVGVGVGETVRELREAYESGRTRSLAWRQAQLRGLLRLLEEKEVEAFQALHK
DLGKHHAEAYRDE
VGVLIKSANGALQQLGKWMAPEKVRVPLIAWPATAQVVPEPLGVVLVFSCWNVPLGLSLEPLIGAIAAGN
AVALKPSELSPCTARFLGDNIGRYMDSSAV
KVVQGGPDVGVQLMEHRWDKVLFTGSPRIARAVMAAASRHLTPVALELGGKCPCIFDAMGSARDLQISVN
RMIAGKWSSCAGQACIAIDYVLVEERFAPI
LIKVLKSTLKRFFPEADHMARIVNERHFERLSNLLKDRSVAPSVLHGGSMDSKNLYIEPTILLNPPLDSA
IMTEEIFGPLLPIITVKNIEDSIAFVKAMP
KPLAIYAFTRDAALRRRIVDETSSGSVTFNDAVVQYAIDGLPFGGVGQSGFGQYHGKYSFEMFSHKKAVM
KRGYLVELTLRYPPWDESKVTLMRYLYRFN
YFAFVLSFLGLRR
>ZmALDH3H1
MDAEAAAAAAATAVEERERLRRSFASGRTRPAAWREAQLRGLLRMATEREDDICAALHADLAKPLTECYV
HEISLVISSCKFA
LKNLKKWMKPRKVPGGLLTFPSAASVAAEPLGVVLVISAWNYPFLLAIDPVVGAFAAGNAVALKPSEVAP
ATSLLLADLLPRYVDPSCVRVVQGGIAETT
ALLELQWDKIFYTGNSRVGRIVMSYAAKHLTPVVLELGGKCPVVVDSDVNLHVAAKRIAAGKWGCNSGQA
CVSPDYVVTTKSFAPKLLESLKRVLFEFYG
EEPLRSPDLSRVVNSNHFNRLMALMDDYSVSGNVAFGGQIDERRLRIAPTLLLDVPLDSAMMKEEIFGPL
LPIITVDKIGESFAVINSMPKPLAAYLFSN
DGQLKQQFERTVSAGGIMFNDTGIHLTNPNLPFGGVGESGMGAYHGAFSFDAFSHRKAVLDRSFLGEARA
RYPPYTPAKLAILRGVLNGSPLATVQAAAG
CTGGASAD
>ZmALDH3H2
MAEETVRELRASFAAGQTRPAEWRAAQLKGLIRMIDEKEAEISAALHEDLAKPHMESFLHEISLTKSSCK
FALKGLKNWMKPE
KVPAAITTFPSSAQIVPEPLGVVLIISAWNYPFILSIDPVIGAIAAGNAVVLKPSEIAPATSSLLAKLLP
EYVDNSCIKVVEGSVPETTALLEQRWDKIF
YTGNGTVGRIVMAAAAKHLTPVALELGGKSPVIVDSNVDLHVAAKRIVVGKWGCNNGQACIAPDYIITTK
SFAPELVASFKRVLERFYGEDPLESADLSR
IVNSKQFKRLTNLIEEKRVADKIVYGGKADEKQLKISPTLLLDVPEDSEIMTGEIFGPLLPIVTVEKIEE
SFDLINAKPKPLAAYLFTKNRKLQEEFVAS
VPAGGMLVNDTALHLTNPYMPFGGVGDSGMGCYHGKFGFDCFSHKKGVLIRGFGGEANARYPPYTTEKQK
ILRGLINGSFIALILALLGFPREKR
>ZmALDH3H3
MAEETVQELRASFASGRTRRAEWRAEQLKGLIRMIDEKEAEISAALHEDLAKPHMESYLHEISITRSSCK
FALDGLKSWMKPE
KIPAALTTFPSSAQIVPEPLGVVLIISAWNYPFILSIDPVIGAIAAGNAVVLKPSEIAPATSSLLAKLLP
EYVDNSCIKVVEGGVAETTSLLEQRWDKIF
YTGNGTVGRIVMAAAAKHLTPVALELGGKSPVVVDSNVDLHVAVKRIVVGKWGCNNGQACIAPDYIITTK
SFAPELVASLKRVLERFYGEDPLQSADLSR

IVNSKHFRRLTELIEEKSVADKIVYGGEVDEKQLKIAPTLLLDVPQDSAIMTGEIFGPLLPIVTVEKIEE
SFDLINARPKPLAAYLFTKNKKLQEEFVAD
VPAGGMLVNDTVLHLANPYMPFGGVGDSGMGCYHGKFGFDCFSHKKGVLVRGFGGEANARYPPYTTEKQK
ILRGLINGSFIALILALLGFPREKR
>ZmALDH5F1
MATAMMTMRRAAALGARHIPPAAAFSRHMSADASAAMEKIRAAGLLKTQGLIAGQWVDAYDGKTIEVQNP
ATGEVLANVSFMG
SRETSDAIASAHSTFYSWSKLTASERGKALRKWYDLIISHKEELALLMTLEQGKPMKEALGEVNYGASFI
EYFAEEAKRIYGDIIPPTLSDRRLLVLKQP
VGVVGAITPWNFPLAMITRKVGPALACGCTVVVKPSEFTPLTSLAAADLALQAGIPAGALNVVMGNAPEI
GDALLQSTQVRKITFTGSTAVGKKLMAESA
NTVKKVSLELGGNAPCIVFDDADIDVAVKGSLAAKFRNSGQTCVCANRILVQEGIYEKFASAFIKAVQSL
KVGNGLEESTSQGPLINEAAVQKVEKFIND
ATSKGANVMLGGKRHSLGMSFYEPTVVGNVSNDMLLFREEVFGPVAPLIPFKTEEEAVHMANDTNAGLAA
YIFTKSIPRSWRVSESLEYGLVGVNEGIIS
TEVAPFGGVKQSGLGREGSKYGVDEYLELKYICMGNLG
>ZmALDH5F2
MAMAMMAMRRAVALGARHIPAAAASSFRVVSLRHMSADAGAAMEKIRAAGLLRTQGLIAGQWVDAYDGKT
IEVQNPATGEVLA
NVSCMGSRETSDAIASAHSTFYSWSKLTASERSKALRKWYDLIISHKEELALLMTLEQGKPMKEALGEVN
YGASFIEYFAEEAKRIYGDIIPPTLSDRRL
LVLKQPVGVVGAITPWNFPLAMITRKVGPALACGCTVVVKPSEFTPLTALAAADLALQAGIPAGALNVVM
GNAAEIGDALLQSTQVRKITFTGSTAVGKK
LMAGSANTVKKVSLELGGNAPCIVFDDADIDVAVKGSLAAKFRNSGQTCVCANRILVQEGIYEKFAKAFI
QAVQSLKVGNGLEESTSQGPLINEAAVQKV
EKFINDATSKGANVMLGGKRHSLGMSFYEPTVVGNVSNDMLLFREEVFGPVAPLIPFKTEEEAVHMANDT
NAGLAAYIFTKSIPRSWRVSESLEYGLVGV
NEGIISTEVAPFGGVKQSGLGREGSKYGIDEYLELKYICMGNLG
>ZmALDH6B1
MLRSALFRSAPGLRRSPATAHLSTAAAAAAAWLSNGPASAPSRVRLLIGGEFVESRADEHVDVTNPATQE
VVSRIPLTTADEF
KAAVDAARTAFPGWRNTPVTTRQRVMFKFQELIRANMDKLAENITTEQGKTLKDAWGDVFRGLEVVEHAC
GMGTLQMGEYVSNVSNGIDTFSIREPLGVC
AGICPFNFPAMIPLWMFPIAVTCGNTFVLKPSEKDPGAAMMLAELAMEAGLPKGVLNIVHGTNDVVNNIC
DDEDIKAVSFVGSNTAGMHIYSRASAAGKR
VQCNMGAKNHAIILPDADRDATLNALIAAGFGAAGQRCMALSTAVFVGGSESWEDELVKRASGLVVSSGM
VNDADLGPVISRQAKDRICKLVQSGVDLCA
RILLDGRKIVVPYPVVY
>ZmALDH7B6
MGAFAKEEHQFLAELGLAQRNPGAFACGAWGGSGPTVTSTSPTNNQVIAEVVEASVHDYEEGMRACFDAA
KTWMAIPAPKRGE
IVRQIGDALRAKLHHLGRLVSLEMGKILPEGIGEVQEIIDMCDYAVGLSRQLNGSIIPSERPNHMMMEVW
NPLGVVGVITAFNFPCAVLGWNACIALVCG
NCVVWKGAPTTPLITIAMTKIVASVLEKNNLPGAIFTSFCGGTEIGQAIALDIRIPLVSFTGSTRAGLMV
QQQVSARFGKCLLELSGNNAIIVMDDADIQ
LAVRSVLFAAVGTAGQRCTTCRRLILHENIYQTFLDQLVEVYKQVRIGDPLEKGTLLGPLHTPASKENFL
KGIQTIKSQGGKILFGGSAIESEGNFVQPT

IVEITPSAPVVKEELFGPVLYVMKFQSLKEAIEINNSVPQGLSSSIFTKRPDIIFKWLGPHGSDCGIVNV
NIPTNGAEIGGAFGGEKATGGGREAGSDSW
KQYMRRATCTINYGSELPLAQGINFG
>ZmALDH10A5
MAPPQTIPRRGLFIGGAWREPCLGRRLPVVNPATEATIGDIPAGTAEDVEIAVAAARDAFSRDGGRHWSR
APGAVRANFLRAI
AAKIKDRKSELALLETLDSGKPLDEASGDMDDVAACFEYYADLAEALDGKQQSPISLPMENFKSYVLKEP
IGVVGLITPWNYPLLMATWKVAPALAAGCT
TILKPSELASVSCLELGAICMEIGLPPGVLNIITGLGPEAGAPLSSHSHVDKVAFTGSTETGKRIMISAA
QMVKPVSLELGGKSPLIVFDDIGDIDKAVE
WTMFGIFANAGQVCSATSRLLLHEKIAKKFLDRLVAWAKNIKVSDPLEEGCRLGSVISEGQYEKIKKFIS
TARSEGATILYGGGRPQHLRRGFFLEPTII
TDVSTSMQIWQEEVFGPVICVKEFRTESEAVELANDTHYGLAGAVISNDQERCERISKALHSGIIWINCS
QPCFVQAPWGGNKRSGFGRELGEWGLDNYL
TVKQVTKYCSDEPWGWYQPPSKL
>ZmALDH10A8
MASPAMVPLRQLFVDGEWRPPAQGRRLPVVNPTTEAHIGEIPAGTAEDVDAAVAAARAALKRNRGRDWAR
APGAVRAKYLRAIAAKVIERKPELAKLEALDCGKPYDEAAWDMDDVAGCFEYFADQAEALDKRQNSPVSL
PMETFKCHLRREPIGVVGLITPWNYPLLMATWKIAPALAAGCTAVLKPSELASVTCLELADICKEVGLPS
GVLNIVTGLGPDAGAPLSAHPDVDKVAFTGSFETGKKIMASAAPMVKPVTLELGGKSPIVVFDDVDIDKA
VEWTLFGCFWTNGQICSATSRLLIHTKIAKKFNERMVAWAKNIKVSDPLEEGCRLGPVVSEGQYEKIKKF
ISNAKSQGATILTGGVRPAHLEKGFFIEPTIITDITTSMEIWREEVFGPVLCVKEFSTEDEAIELANDTQ
YGLAGAVISGDRERCQRLSEEIDAGCIWVNCSQPCFCQAPWGGNKRSGFGRELGEGGIDNYLSVKQVTEY
ISDEPWGWYQSPSKL
>ZmALDH10A9
MMASQAMVPLRQLFVDGEWRPPAQGRRLPVVNPTTEAHIGEIPAGTAEDVDAAVAAARAALKRNRGRDWA
RAPGAVRAKYLRA
IAAKVIERKQELAKLEALDCGKPYDEAAWDMDDVAGCFEYFADQAEALDKRQNSPVSLPMETFKCHLRRE
PIGVVGLITPWNYPLLMATWKVAPALAAGC
AAVLKPSELASVTCLELADICKEVGLPPGVLNIVTGLGPDAGAPLSAHPDVDKVAFTGSFETGKKIMAAA
APMVKPVTLELGGKSPIVVFDDVDIDKAVE
WTLFGCFWTNGQICSATSRLLVHTKIAKEFNEKMVAWAKNIKVSDPLEEGCRLGPVVSEGQYEKIKKFIL
NAKSEGATILTGGVRPAHLEKGFFIEPTII
TDITTSMEIWREEVFGPVLCVKEFSTEDEAIELANDTQYGLAGAVISGDRERCQRLSEEIDAGIIWVNCS
QPCFCQAPWGGNKRSGFGRELGEGGIDNYL
SVKQVTEYISDEPWGWYRSPSKL
>ZmALDH11A3
MALAGTGVFAEILDSEVYRYYADGEWRSSASGKSVAIVNPTTRKTQYRVQACTQEEVNKAMDAAKVAQKA
WARTPLWKRAELL
HKAAAILKEHKAPIAECLVKEIAKPAKDAVSEVVRSGDLVSYTAEEGVRILGEGKLLVSDSFPGNERNKY
CLSSKIPLGVVLAIPPFNYPVNLAVSKIGP
ALIAGNALVLKPPTQGAVAALHMVHCFHLAGFPKGLISCVTGKGSEIGDFLTMHPGVNCISFTGGDTGIA
ISKKAGMVPLQMELGGKDACIVLEDADLDL
VSANIVKGGFSYSGQRCTAVKVVLIMESIADAVVQKVNAKLAKLKVGPPEDDSDITPVVTESSANFIEGL
VMDAKEKGATFCQEYRREGNLIWPLLLDHV
RPDMRIAWEEPFGPVLPVIRINSVEEGIHHCNASNFGLQGCIFTRDINKAILISDAMETGTVQINSAPAR
GPDHFPFQGLKDSGIGSQGITNSINMMTKV
KSTVINLPSPSYTMG

>ZmALDH12A1
MSRLLSRQHLAAVRRSAPFACVSRWLHTPSFATVSPQEVSGSSPAEVQNFVQGSWTASANWNWIVDPLNG
DKFIKVAEVQGTE
IKPFVESLSKCPKHGLHNPLKAPERYLMYGDISAKAAHMLGQPAVLDFFAKLIQRVSPKSYQQALAEVQV
SQKFLENFCGDQVRFLARSFAVPGNHLGQR
SNGYRWPYGPVAIITPFNFPLEIPLLQLMGALYMGNKPVLKVDSKVSIVMEQMIRLLHDCGLPAEDMDFI
NSDGAVMNKLLLEANPKMTLFTGSSRVAEK
LAADLKGRVKLEDAGFDWKILGPDVQEVDYVAWVCDQDAYACSGQKCSAQSVLFMHKNWSSSGLLEKMKK
LSERRKLEDLTIGPVLTVTTEAMIEHMNNL
LKIRGSKVLFGGEPLANHSIPKIYGAMKPTAVFVPLEEILKSGNFELVTKEIFGPFQVVTEYSEDQLELV
LEACERMNAHLTAAVVSNDPLFLQDVLGRS
VNGTTYAGIRARTTGAPQNHWFGPAGDPRGAGIGTPEAIKLVWSCHREVIYDVGPVPESWALPSAT
>ZmALDH18B2
MGRGGIGGAAAMAMAMETADPARAFVKDVKRIIIKVGTAVVTGMNGRLAMGRLGSLCEQVKQLNFQGYEV
ILVTSGAVGVGRQ
RLQYRKLIHSSFADLQNPQMNFDGKACAAVGQSVLMAIYDTLFSQLDVTSSQLLVTDRDFKDPSFGDQLR
ETVFSLLDLKVVPLFNENDAISTRRQPYED
SSGIFWDNDSLAALLAAELNADLLIMLSDVEGLYSGPPSDPQSKIIHTYVNEKHGKLISFGEKSSVGRGG
MQAKVSAAANAASKGVPVVIASGFATDSII
TVLKGEKIGTLFHNEANLWACSKEATAREMAVAARDCSRRLQKLSSEERKQILLDIADALEANEDAIRSE
NDADVEAAQVAGYEKSLVARMTLKPGKITN
LARSIRKTADMEDPISHTLKRTEVAKDLVFEKAYCPLGVLLIIFESRPDALVQIASLAIRSGNGLLLKGG
KEVMRSNAILHKVITGVIPDTVGKKLIGLV
TSKEEIADLLALDDVIDLVIPRGSKSLVSQIKATTKIPVLGHADGICHVYIDKSADMDMAKRIVLDAKID
YPAACNAMETLLVHKDLNKSEGLDDLLVEL
EKEGVVIYGGPVAHDKLKVPKVDSFRHEYSSMACTVEFVDDVQSAIDHINRYGSAHTDCIITTDRSAAEA
FLQQVDSAAVFHNASTRFCDGTRFGLGAEV
GISTERIHARGPVGVDGLLTTRCILRGSGQVVNGDKGVVYTHKDLPLQ
>ZmALDH18B1
MATADRTRTFMKDVKRVIIKVGTAVVTRGDDGRLAVGRLGCLCEQVKELNVLGYEVILVTSGAVGVGKQR
LKYRKLVNSSFAD
LQKPQMELDGKACAAVGQSGLMALYDMLFTQLDVSSSQLLVTDSDFENPNFRERLCETVESLLDLKVVPI
FNENDAISTRKAPYEDSSGIFWDNDSLAGL
LAIELKADLLVLLSDVDGLYSGPPSEPGSKIIHTYIKDKHYSGITFGDKSRVGRGGMTAKVKAAFVASNS
GTPVVITSGFASQSIVRVLQGEKIGTLFHK
DASLWEPSKDVSAREMAVAARECSRRLQNLSSDERKKILLDIADALEQNEDLIRTENEADVSAAQDAGYQ
KSLVDRLTLKPEKIASLAKSIRTLANMEDP
INQILKRTEVAEDLVLEKTSCPLGVLLIVFESRPDALVQIASLAVRSGNGLLLKGGKEAMRSNTVLHKVI
TGAIPDNVGQKLIGLVTSRDEIADLLKLDD
VIDLVIPRGSNKLVSQIKASTKIPVLGHADGICHVYIDKSADMNMAKRIVMDAKTDYPAACNAMETLLVH
KDLIKAPGLDDILLSLKTEGVAIYGGPVAH
EVLCIPKADSFHHEYSSMACTIEFVDDVQSAINHIHRYGSAHTDCIITTDDKVAETFLRQVDSAAVFHNA
STRFSDGARFGLGAEVGISTGRIHARGPVG
VEGLLTTRWIMRGSGQVVNGDKNVAYTHKNLPLQ
>ZmALDH22A1
MAFWWPLLVLAAAYALCRLLLFLIPPTVPSIDVDASDVLAKEDSFIYIPRRGKSTQTDKVQCYEPATMKY
LGYFPVVTPDEVK

EHVAQSRKAQRIWAKSSFKQRRQFLRILLKYILEHQDLICEVSSRDTGKTMVDASLGEIMTTCEKITWLL
DEGEKWLKPEYRSTGRSMLHKRAKVEFYPL
GVIGAIVSWNYPFHNVFNPVLAAVFSGNAAVIKVSEHATWSGCFYFRIIQAALSAVGAPENLVHIITGFA
ETGQALVSSVDKIIFVGSPGVGKMIMKRAS
ETLIPVTLELGGKDSFIVCEDVDLPSVVQVATRAALQSSGQNCAGAERFYVHDDIYSAFVSQIVKTVKSI
SVGPPLSGRYDMGAICMIEHSEKLQNLVND
ALDKGAEIAVRGSFGNLGEDAVDQFFPPTVLVNVDHTMKIMQEETFGPIIPIMKFSSDEEAIKLANDSKY
GLGCAVFSGNQKRAIRIASQLHCGVAAIND
FASSYMCQSLPFGGVKDSGFGRFAGVEGLRACCLVKSVVEDRLWPYIRTVIPKPIQYPVSEHGFEFQQLL
VETLYGYSVWDRLRSLVNLIKMVTEQNFAP
TSNATTKKRR
>HsALDH1A1
MSSSGTPDLPVLLTDLKIQYTKIFINNEWHDSVSGKKFPVFNPATEEELCQVEEGDKEDVDKAVKAARQA
FQIGSPWRTMDASERGRLLYKLADLIERDRLLLATMESMNGGKLYSNAYLNDLAGCIKTLRYCAGWADKI
QGRTIPIDGNFFTYTRHEPIGVCGQIIPWNFPLVMLIWKIGPALSCGNTVVVKPAEQTPLTALHVASLIK
EAGFPPGVVNIVPGYGPTAGAAISSHMDIDKVAFTGSTEVGKLIKEAAGKSNLKRVTLELGGKSPCIVLA
DADLDNAVEFAHHGVFYHQGQCCIAASRIFVEESIYDEFVRRSVERAKKYILGNPLTPGVTQGPQIDKEQ
YDKILDLIESGKKEGAKLECGGGPWGNKGYFVQPTVFSNVTDEMRIAKEEIFGPVQQIMKFKSLDDVIKR
ANNTFYGLSAGVFTKDIDKAITISSALQAGTVWVNCYGVVSAQCPFGGFKMSGNGRELGEYGFHEYTEVK
TVTVKISQKNS
>HsALDH1A2
MTSSKIEMPGEVKADPAALMASLHLLPSPTPNLEIKYTKIFINNEWQNSESGRVFPVYNPATGEQVCEVQ
EADKADIDKAVQAARLAFSLGSVWRRMDASERGRLLDKLADLVERDRAVLATMESLNGGKPFLQAFYVDL
QGVIKTFRYYAGWADKIHGMTIPVDGDYFTFTRHEPIGVCGQIIPWNFPLLMFAWKIAPALCCGNTVVIK
PAEQTPLSALYMGALIKEAGFPPGVINILPGYGPTAGAAIASHIGIDKIAFTGSTEVGKLIQEAAGRSNL
KRVTLELGGKSPNIIFADADLDYAVEQAHQGVFFNQGQCCTAGSRIFVEESIYEEFVRRSVERAKRRVVG
SPFDPTTEQGPQIDKKQYNKILELIQSGVAEGAKLECGGKGLGRKGFFIEPTVFSNVTDDMRIAKEEIFG
PVQEILRFKTMDEVIERANNSDFGLVAAVFTNDINKALTVSSAMQAGTVWINCYNALNAQSPFGGFKMSG
NGREMGEFGLREYSEVKTVTVKIPQKNS
>HsALDH1A3
MATANGAVENGQPDRKPPALPRPIRNLEVKFTKIFINNEWHESKSGKKFATCNPSTREQICEVEEGDKPD
VDKAVEAAQVAFQRGSPWRRLDALSRGRLLHQLADLVERDRATLAALETMDTGKPFLHAFFIDLEGCIRT
LRYFAGWADKIQGKTIPTDDNVVCFTRHEPIGVCGAITPWNFPLLMLVWKLAPALCCGNTMVLKPAEQTP
LTALYLGSLIKEAGFPPGVVNIVPGFGPTVGAAISSHPQINKIAFTGSTEVGKLVKEAASRSNLKRVTLE
LGGKNPCIVCADADLDLAVECAHQGVFFNQGQCCTAASRVFVEEQVYSEFVRRSVEYAKKRPVGDPFDVK
TEQGPQIDQKQFDKILELIESGKKEGAKLECGGSAMEDKGLFIKPTVFSEVTDNMRIAKEEIFGPVQPIL
KFKSIEEVIKRANSTDYGLTAAVFTKNLDKALKLASALESGTVWINCYNALYAQAPFGGFKMSGNGRELG
EYALAEYTEVKTVTIKLGDKNP
>HsALDH1B1
MLRFLAPRLLSLQGRTARYSSAAALPSPILNPDIPYNQLFINNEWQDAVSKKTFPTVNPTTGEVIGHVAE
GDRADVDRAVKAAREAFRLGSPWRRMDASERGRLLNRLADLVERDRVYLASLETLDNGKPFQESYALDLD
EVIKVYRYFAGWADKWHGKTIPMDGQHFCFTRHEPVGVCGQIIPWNFPLVMQGWKLAPALATGNTVVMKV
AEQTPLSALYLASLIKEAGFPPGVVNIITGYGPTAGAAIAQHVDVDKVAFTGSTEVGHLIQKAAGDSNLK
RVTLELGGKSPSIVLADADMEHAVEQCHEALFFNMGQCCCAGSRTFVEESIYNEFLERTVEKAKQRKVGN
PFELDTQQGPQVDKEQFERVLGYIQLGQKEGAKLLCGGERFGERGFFIKPTVFGGVQDDMRIAKEEIFGP
VQPLFKFKKIEEVVERANNTRYGLAAAVFTRDLDKAMYFTQALQAGTVWVNTYNIVTCHTPFGGFKESGN
GRELGEDGLKAYTEVKTVTIKVPQKNS
>HsALDH1L1

MKIAVIGQSLFGQEVYCHLRKEGHEVVGVFTVPDKDGKADPLGLEAEKDGVPVFKYSRWRAKGQALPDVV
AKYQALGAELNVLPFCSQFIPMEIISAPRHGSIIYHPSLLPRHRGASAINWTLIHGDKKGGFSIFWADDG
LDTGDLLLQKECEVLPDDTVSTLYNRFLFPEGIKGMVQAVRLIAEGKAPRLPQPEEGATYEGIQKKETAK
INWDQPAEAIHNWIRGNDKVPGAWTEACEQKLTFFNSTLNTSGLVPEGDALPIPGAHRPGVVTKAGLILF
GNDDKMLLVKNIQLEDGKMILASNFFKGAASSVLELTEAELVTAEAVRSVWQRILPKVLEVEDSTDFFKS
GAASVDVVRLVEEVKELCDGLELENEDVYMASTFGDFIQLLVRKLRGDDEEGECSIDYVEMAVNKRTVRM
PHQLFIGGEFVDAEGAKTSETINPTDGSVICQVSLAQVTDVDKAVAAAKDAFENGRWGKISARDRGRLMY
RLADLMEQHQEELATIEALDAGAVYTLALKTHVGMSIQTFRYFAGWCDKIQGSTIPINQARPNRNLTLTR
KEPVGVCGIIIPWNYPLMMLSWKTAACLAAGNTVVIKPAQVTPLTALKFAELTLKAGIPKGVVNVLPGSG
SLVGQRLSDHPDVRKIGFTGSTEVGKHIMKSCAISNVKKVSLELGGKSPLIIFADCDLNKAVQMGMSSVF
FNKGENCIAAGRLFVEDSIHDEFVRRVVEEVRKMKVGNPLDRDTDHGPQNHHAHLVKLMEYCQHGVKEGA
TLVCGGNQVPRPGFFFEPTVFTDVEDHMFIAKEESFGPVMIISRFADGDLDAVLSRANATEFGLASGVFT
RDINKALYVSDKLQAGTVFVNTYNKTDVAAPFGGFKQSGFGKDLGEAALNEYLRVKTVTFEY
>HsALDH1L2
MLRRGSQALRRFSTGRVYFKNKLKLALIGQSLFGQEVYSHLRKEGHRVVGVFTVPDKDGKADPLALAAEK
DGTPVFKLPKWRVKGKTIKEVAEAYRSVGAELNVLPFCTQFIPMDIIDSPKHGSIIYHPSILPRHRGASA
INWTLIMGDKKAGFSVFWADDGLDTGPILLQRSCDVEPNDTVDALYNRFLFPEGIKAMVEAVQLIADGKA
PRIPQPEEGATYEGIQKKENAEISWDQSAEVLHNWIRGHDKVPGAWTEINGQMVTFYGSTLLNSSVPPGE
PLEIKGAKKPGLVTKNGLVLFGNDGKALTVRNLQFEDGKMIPASQYFSTGETSVVELTAEEVKVAETIKV
IWAGILSNVPIIEDSTDFFKSGASSMDVARLVEEIRQKCGGLQLQNEDVYMATKFEGFIQKVVRKLRGED
QEVELVVDYISKEVNEIMVKMPYQCFINGQFTDADDGKTYDTINPTDGSTICKVSYASLADVDKAVAAAK
DAFENGEWGRMNARERGRLMYRLADLLEENQEELATIEALDSGAVYTLALKTHIGMSVQTFRYFAGWCDK
IQGSTIPINQARPNRNLTFTKKEPLGVCAIIIPWNYPLMMLAWKSAACLAAGNTLVLKPAQVTPLTALKF
AELSVKAGFPKGVINIIPGSGGIAGQRLSEHPDIRKLGFTGSTPIGKQIMKSCAVSNLKKVSLELGGKSP
LIIFNDCELDKAVRMGMGAVFFNKGENCIAAGRLFVEESIHDEFVTRVVEEIKKMKIGDPLDRSTDHGPQ
NHKAHLEKLLQYCETGVKEGATLVYGGRQVQRPGFFMEPTVFTDVEDYMYLAKEESFGPIMVISKFQNGD
IDGVLQRANSTEYGLASGVFTRDINKAMYVSEKLEAGTVFINTYNKTDVAAPFGGVKQSGFGKDLGEEAL
NEYLKTKTVTLEY
>HsALDH2
MLRAAARFGPRLGRRLLSAAATQAVPAPNQQPEVFCNQIFINNEWHDAVSRKTFPTVNPSTGEVICQVAE
GDKEDVDKAVKAARAAFQLGSPWRRMDASHRGRLLNRLADLIERDRTYLAALETLDNGKPYVISYLVDLD
MVLKCLRYYAGWADKYHGKTIPIDGDFFSYTRHEPVGVCGQIIPWNFPLLMQAWKLGPALATGNVVVMKV
AEQTPLTALYVANLIKEAGFPPGVVNIVPGFGPTAGAAIASHEDVDKVAFTGSTEIGRVIQVAAGSSNLK
RVTLELGGKSPNIIMSDADMDWAVEQAHFALFFNQGQCCCAGSRTFVQEDIYDEFVERSVARAKSRVVGN
PFDSKTEQGPQVDETQFKKILGYINTGKQEGAKLLCGGGIAADRGYFIQPTVFGDVQDGMTIAKEEIFGP
VMQILKFKTIEEVVGRANNSTYGLAAAVFTKDLDKANYLSQALQAGTVWVNCYDVFGAQSPFGGYKMSGS
GRELGEYGLQAYTEVKTVTVKVPQKNS
>HsALDH3A1
MSKISEAVKRARAAFSSGRTRPLQFRIQQLEALQRLIQEQEQELVGALAADLHKNEWNAYYEEVVYVLEE
IEYMIQKLPEWAADEPVEKTPQTQQDELYIHSEPLGVVLVIGTWNYPFNLTIQPMVGAIAAGNSVVLKPS
ELSENMASLLATIIPQYLDKDLYPVINGGVPETTELLKERFDHILYTGSTGVGKIIMTAAAKHLTPVTLE
LGGKSPCYVDKNCDLDVACRRIAWGKFMNSGQTCVAPDYILCDPSIQNQIVEKLKKSLKEFYGEDAKKSR
DYGRIISARHFQRVMGLIEGQKVAYGGTGDAATRYIAPTILTDVDPQSPVMQEEIFGPVLPIVCVRSLEE
AIQFINQREKPLALYMFSSNDKVIKKMIAETSSGGVAANDVIVHITLHSLPFGGVGNSGMGSYHGKKSFE
TFSHRRSCLVRPLMNDEGLKVRYPPSPAKMTQH
>HsALDH3A2
MELEVRRVRQAFLSGRSRPLRFRLQQLEALRRMVQEREKDILTAIAADLCKSEFNVYSQEVITVLGEIDF
MLENLPEWVTAKPVKKNVLTMLDEAYIQPQPLGVVLIIGAWNYPFVLTIQPLIGAIAAGNAVIIKPSELS

ENTAKILAKLLPQYLDQDLYIVINGGVEETTELLKQRFDHIFYTGNTAVGKIVMEAAAKHLTPVTLELGG
KSPCYIDKDCDLDIVCRRITWGKYMNCGQTCIAPDYILCEASLQNQIVWKIKETVKEFYGENIKESPDYE
RIINLRHFKRILSLLEGQKIAFGGETDEATRYIAPTVLTDVDPKTKVMQEEIFGPILPIVPVKNVDEAIN
FINEREKPLALYVFSHNHKLIKRMIDETSSGGVTGNDVIMHFTLNSFPFGGVGSSGMGAYHGKHSFDTFS
HQRPCLLKSLKREGANKLRYPPNSQSKVDWGKFFLLKRFNKEKLGLLLLTFLGIVAAVLVKAEYY
>HsALDH3B1
MDPLGDTLRRLREAFHAGRTRPAEFRAAQLQGLGRFLQENKQLLHDALAQDLHKSAFESEVSEVAISQGE
VTLALRNLRAWMKDERVPKNLATQLDSAFIRKEPFGLVLIIAPWNYPLNLTLVPLVGALAAGNCVVLKPS
EISKNVEKILAEVLPQYVDQSCFAVVLGGPQETGQLLEHRFDYIFFTGSPRVGKIVMTAAAKHLTPVTLE
LGGKNPCYVDDNCDPQTVANRVAWFRYFNAGQTCVAPDYVLCSPEMQERLLPALQSTITRFYGDDPQSSP
NLGRIINQKQFQRLRALLGCGRVAIGGQSDESDRYIAPTVLVDVQEMEPVMQEEIFGPILPIVNVQSLDE
AIEFINRREKPLALYAFSNSSQVVKRVLTQTSSGGFCGNDGFMHMTLASLPFGGVGASGMGRYHGKFSFD
TFSHHRACLLRSPGMEKLNALRYPPQSPRRLRMLLVAMEAQGCSCTLL
>HsALDH3B2
MKDEPRSTNLFMKLDSVFIWKEPFGLVLIIAPWNYPLNLTLVLLVGALAAGSCVVLKPSEISQGTEKVLA
EVLPQYLDQSCFAVVLGGPQETGQLLEHKLDYIFFTGSPRVGKIVMTAATKHLTPVTLELGGKNPCYVDD
NCDPQTVANRVAWFCYFNAGQTCVAPDYVLCSPEMQERLLPALQSTITRFYGDDPQSSPNLGRIINQKQF
QRLRALLGCGRVAIGGQSNESDRYIAPTVLVDVQETEPVMQEEIFGPILPIVNVQSVDEAIKFINRQEKP
LALYAFSNSSQVVNQMLERTSSGSFGGNEGFTYISLLSVPFGGVGHSGMGRYHGKFTFDTFSHHRTCLLA
PSGLEKLKEIHYPPYTDWNQQLLRWGMGSQSCTLL
>HsALDH4A1
MLLPAPALRRALLSRPWTGAGLRWKHTSSLKVANEPVLAFTQGSPERDALQKALKDLKGRMEAIPCVVGD
EEVWTSDVQYQVSPFNHGHKVAKFCYADKSLLNKAIEAALAARKEWDLKPIADRAQIFLKAADMLSGPRR
AEILAKTMVGQGKTVIQAEIDAAAELIDFFRFNAKYAVELEGQQPISVPPSTNSTVYRGLEGFVAAISPF
NFTAIGGNLAGAPALMGNVVLWKPSDTAMLASYAVYRILREAGLPPNIIQFVPADGPLFGDTVTSSEHLC
GINFTGSVPTFKHLWKQVAQNLDRFHTFPRLAGECGGKNFHFVHRSADVESVVSGTLRSAFEYGGQKCSA
CSRLYVPHSLWPQIKGRLLEEHSRIKVGDPAEDFGTFFSAVIDAKSFARIKKWLEHARSSPSLTILAGGK
CDDSVGYFVEPCIVESKDPQEPIMKEEIFGPVLSVYVYPDDKYKETLQLVDSTTSYGLTGAVFSQDKDVV
QEATKVLRNAAGNFYINDKSTGSIVGQQPFGGARASGTNDKPGGPHYILRWTSPQVIKETHKPLGDWSYA
YMQ
>HsALDH5A1
MATCIWLRSCGARRLGSTFPGCRLRPRAGGLVPASGPAPGPAQLRCYAGRLAGLSAALLRTDSFVGGRWL
PAAATFPVQDPASGAALGMVADCGVREARAAVRAAYEAFCRWREVSAKERSSLLRKWYNLMIQNKDDLAR
IITAESGKPLKEAHGEILYSAFFLEWFSEEARRVYGDIIHTPAKDRRALVLKQPIGVAAVITPWNFPSAM
ITRKVGAALAAGCTVVVKPAEDTPFSALALAELASQAGIPSGVYNVIPCSRKNAKEVGEAICTDPLVSKI
SFTGSTTTGKILLHHAANSVKRVSMELGGLAPFIVFDSANVDQAVAGAMASKFRNTGQTCVCSNQFLVQR
GIHDAFVKAFAEAMKKNLRVGNGFEEGTTQGPLINEKAVEKVEKQVNDAVSKGATVVTGGKRHQLGKNFF
EPTLLCNVTQDMLCTHEETFGPLAPVIKFDTEEEAIAIANAADVGLAGYFYSQDPAQIWRVAEQLEVGMV
GVNEGLISSVECPFGGVKQSGLGREGSKYGIDEYLELKYVCYGGL
>HsALDH6A1
MAALLAAAAVRARILQVSSKVKSSPTWYSASSFSSSVPTVKLFIGGKFVESKSDKWIDIHNPATNEVIGR
VPQATKAEMDAAIASCKRAFPAWADTSVLSRQQVLLRYQQLIKENLKEIAKLITLEQGKTLADAEGDVFR
GLQVVEHACSVTSLMMGETMPSITKDMDLYSYRLPLGVCAGIAPFNFPAMIPLWMFPMAMVCGNTFLMKP
SERVPGATMLLAKLLQDSGAPDGTLNIIHGQHEAVNFICDHPDIKAISFVGSNKAGEYIFERGSRHGKRV
QANMGAKNHGVVMPDANKENTLNQLVGAAFGAAGQRCMALSTAVLVGEAKKWLPELVEHAKNLRVNAGDQ
PGADLGPLITPQAKERVCNLIDSGTKEGASILLDGRKIKVKGYENGNFVGPTIISNVKPNMTCYKEEIFG
PVLVVLETETLDEAIQIVNNNPYGNGTAIFTTNGATARKYAHLVDVGQVGVNVPIPVPLPMFSFTGSRSS
FRGDTNFYGKQGIQFYTQLKTITSQWKEEDATLSSPAVVMPTMGR

>HsALDH7A1
MWRLPRALCVHAAKTSKLSGPWSRPAAFMSTLLINQPQYAWLKELGLREENEGVYNGSWGGRGEVITTYC
PANNEPIARVRQASVADYEETVKKAREAWKIWADIPAPKRGEIVRQIGDALREKIQVLGSLVSLEMGKIL
VEGVGEVQEYVDICDYAVGLSRMIGGPILPSERSGHALIEQWNPVGLVGIITAFNFPVAVYGWNNAIAMI
CGNVCLWKGAPTTSLISVAVTKIIAKVLEDNKLPGAICSLTCGGADIGTAMAKDERVNLLSFTGSTQVGK
QVGLMVQERFGRSLLELGGNNAIIAFEDADLSLVVPSALFAAVGTAGQRCTTARRLFIHESIHDEVVNRL
KKAYAQIRVGNPWDPNVLYGPLHTKQAVSMFLGAVEEAKKEGGTVVYGGKVMDRPGNYVEPTIVTGLGHD
ASIAHTETFAPILYVFKFKNEEEVFAWNNEVKQGLSSSIFTKDLGRIFRWLGPKGSDCGIVNVNIPTSGA
EIGGAFGGEKHTGGGRESGSDAWKQYMRRSTCTINYSKDLPLAQGIKFQ
>HsALDH8A1
MAGTNALLMLENFIDGKFLPCSSYIDSYDPSTGEVYCRVPNSGKDEIEAAVKAAREAFPSWSSRSPQERS
RVLNQVADLLEQSLEEFAQAESKDQGKTLALARTMDIPRSVQNFRFFASSSLHHTSECTQMDHLGCMHYT
VRAPVGVAGLISPWNLPLYLLTWKIAPAMAAGNTVIAKPSELTSVTAWMLCKLLDKAGVPPGVVNIVFGT
GPRVGEALVSHPEVPLISFTGSQPTAERITQLSAPHCKKLSLELGGKNPAIIFEDANLDECIPATVRSSF
ANQGEICLCTSRIFVQKSIYSEFLKRFVEATRKWKVGIPSDPLVSIGALISKAHLEKVRSYVKRALAEGA
QIWCGEGVDKLSLPARNQAGYFMLPTVITDIKDESCCMTEEIFGPVTCVVPFDSEEEVIERANNVKYGLA
ATVWSSNVGRVHRVAKKLQSGLVWTNCWLIRELNLPFGGMKSSGIGREGAKDSYDFFTEIKTITVKH
>HsALDH9A1
MFLRAGLAALSPLLRSLRPSPVAAMSTGTFVVSQPLNYRGGARVEPADASGTEKAFEPATGRVIATFTCS
GEKEVNLAVQNAKAAFKIWSQKSGMERCRILLEAARIIREREDEIATMECINNGKSIFEARLDIDISWQC
LEYYAGLAASMAGEHIQLPGGSFGYTRREPLGVCVGIGAWNYPFQIASWKSAPALACGNAMVFKPSPFTP
VSALLLAEIYSEAGVPPGLFNVVQGGAATGQFLCQHPDVAKVSFTGSVPTGMKIMEMSAKGIKPVTLELG
GKSPLIIFSDCDMNNAVKGALMANFLTQGQVCCNGTRVFVQKEILDKFTEEVVKQTQRIKIGDPLLEDTR
MGPLINRPHLERVLGFVKVAKEQGAKVLCGGDIYVPEDPKLKDGYYMRPCVLTNCRDDMTCVKEEIFGPV
MSILSFDTEAEVLERANDTTFGLAAGVFTRDIQRAHRVVAELQAGTCFINNYNVSPVELPFGGYKKSGFG
RENGRVTIEYYSQLKTVCVEMGDVESAF
>HsALDH16A1
MAATRAGPRAREIFTSLEYGPVPESHACALAWLDTQDRCLGHYVNGKWLKPEHRNSVPCQDPITGENLAS
CLQAQAEDVAAAVEAARMAFKGWSAHPGVVRAQHLTRLAEVIQKHQRLLWTLESLVTGRAVREVRDGDVQ
LAQQLLHYHAIQASTQEEALAGWEPMGVIGLILPPTFSFLEMMWRICPALAVGCTVVALVPPASPAPLLL
AQLAGELGPFPGILNVLSGPASLVPILASQPGIRKVAFCGAPEEGRALRRSLAGECAELGLALGTESLLL
LTDTADVDSAVEGVVDAAWSDRGPGGLRLLIQESVWDEAMRRLQERMGRLRSGRGLDGAVDMGARGAAAC
DLVQRFVREAQSQGAQVFQAGDVPSERPFYPPTLVSNLPPASPCAQVEVPWPVVVASPFRTAKEALLVAN
GTPRGGSASVWSERLGQALELGYGLQVGTVWINAHGLRDPSVPTGGCKESGCSWHGGPDGLYEYLRPSGT
PARLSCLSKNLNYDTFGLAVPSTLPAGPEIGPSPAPPYGLFVGGRFQAPGARSSRPIRDSSGNLHGYVAE
GGAKDIRGAVEAAHQAFPGWAGQSPGARAALLWALAAALERRKSTLASRLERQGAELKAAEAEVELSARR
LRAWGARVQAQGHTLQVAGLRGPVLRLREPLGVLAVVCPDEWPLLAFVSLLAPALAYGNTVVMVPSAACP
LLALEVCQDMATVFPAGLANVVTGDRDHLTRCLALHQDVQAMWYFGSAQGSQFVEWASAGNLKPVWASRG
CPRAWDQEAEGAGPELGLRVARTKALWLPMGD
>HsALDH18A1
MLSQVYRCGFQPFNQHLLPWVKCTTVFRSHCIQPSVIRHVRSWSNIPFITVPLSRTHGKSFAHRSELKHA
KRIVVKLGSAVVTRGDECGLALGRLASIVEQVSVLQNQGREMMLVTSGAVAFGKQRLRHEILLSQSVRQA
LHSGQNQLKEMAIPVLEARACAAAGQSGLMALYEAMFTQYSICAAQILVTNLDFHDEQKRRNLNGTLHEL
LRMNIVPIVNTNDAVVPPAEPNSDLQGVNVISVKDNDSLAARLAVEMKTDLLIVLSDVEGLFDSPPGSDD
AKLIDIFYPGDQQSVTFGTKSRVGMGGMEAKVKAALWALQGGTSVVIANGTHPKVSGHVITDIVEGKKVG
TFFSEVKPAGPTVEQQGEMARSGGRMLATLEPEQRAEIIHHLADLLTDQRDEILLANKKDLEEAEGRLAA
PLLKRLSLSTSKLNSLAIGLRQIAASSQDSVGRVLRRTRIAKNLELEQVTVPIGVLLVIFESRPDCLPQV
AALAIASGNGLLLKGGKEAAHSNRILHLLTQEALSIHGVKEAVQLVNTREEVEDLCRLDKMIDLIIPRGS

SQLVRDIQKAAKGIPVMGHSEGICHMYVDSEASVDKVTRLVRDSKCEYPAACNALETLLIHRDLLRTPLF
DQIIDMLRVEQVKIHAGPKFASYLTFSPSEVKSLRTEYGDLELCIEVVDNVQDAIDHIHKYGSSHTDVIV
TEDENTAEFFLQHVDSACVFWNASTRFSDGYRFGLGAEVGISTSRIHARGPVGLEGLLTTKWLLRGKDHV
VSDFSEHGSLKYLHENLPIPQRNTN
>CrALDH2E1
MAMSTLRAALGHAARVAHEGSLVGLLSRGMASAAQPAVDHAHHEKSRSALSYAQKLPNQLFIDGKWVDAL
SRKTMPVVDPRTEEVVVEVAEGDAADVDRAVEAARRAFDTGPWPRMTAKERGRLLYRLADAMEAHVDELA
QLETLDNGKPFFYSRHVDVPFAIDHLRYYAGWADKIHGKTIPVDGPYLAYTFHEPLGVVGQIIPWNFPIL
MAAWKLGPALAAGNTVVLKPAEQTPMTALKVAQLAKEVGLPDGVLNVVTGYGPTAGNRVASHPGVDKTAF
TGSTEVGRLVAKAAAEQLKPCTLELGGKSPIIVCPDVDVDKAVADAHMALFFNHGQCCAAGSRVYVHEAV
YDEFVRKSTEAAATRKVGDPFSSVEQGPQVDDDQFKKILSYIDSGKRQGAKLMTGGGRKGDRGYYVEPTV
FADVKDDMKIAREEIFGPVQSIMKWKSLDDVIARANNSPYGLAAGVFSNNIDTVNTLTRALKSGTVWVNC
YNLYDNAVPFGGYKESGIGREKGEYALSNYTQVKAVYQPLSNPAWR
>CrALDH5G1
MPPRITCARTQVLNPATGAVIATLPRMRADETRAAIAAAHSVLPQWRATPARERAAILRRWHDLILQHQS
DIAALMTAECGKPTAEALAEIAGGAASVDWFAGEAVRVAGDVLEPPSRDRRMVVLKQPVGVVGAITPWNF
PMSMITRKVAPALAAGCTALVRPATTTPHIRLPPPAHTPKRAGLPDGVLNLVLGDAAAIGHELVHSDTVR
KIGFTGSTAVGKMLAAGAGAGVKRVSLELGGNAPVLVFEDADLELAARGIVASALRNAGQTCICANRVFV
HTAVYDKLAEAVVGRVRKLKVGDGAEPGVHVGPLITPAALDKAIVTAHVHDAVAKGGKLLLAGGGGGGGG
GAAAAAAGNFYLPTVIGEATIDMRCFKEETFGPLIPLFRFTSDEEAVLLANTTEYGLAAYFYTRDLGRAW
RVAEELEFGMVGASMGWVGGEVAPFGGVKQSGLGREQSKYGIAEFMDIKYVCMGLG
>CrALDH6B1
MLRGQRSSPVLRAGHRFFASAAAPSPVAAAAAPPKVKLLIDGQFVDSTTENWLDVVNPANQDVLGKLPLT
TKSEFNAAVKAASDAFPKWRATPVPTRVRVMFKFQELIRANMEELARSVTMEQGKTLADARGDVFRGLEV
VETACGIAPYMTGEMVENVAGGIDCYSIRQPLGVVAGICPFNFPAMVPLWMFPLAITAGNTFVLKPSERD
PGAAVMLADLAQQAGLPKGVLNIVQGSRDVVNWICDDPAIRAISFVGSDSAGKYIYARGCAAGKRVQANL
GAKNHAVVMPDADVDSTVKALAGAAFGAAGQRCMAISAAVFVGGFTDKWREPLLEAARGLKLNAGWEKDA
DVGPMISPEAKARAERLIASGAAAGAQVLLDGRGVSVPGYERGNFLGPTLLAGVTPDMEAYREEIFGPVL
SCMDAATLDDALAIVNGNEHGNGTAIFTRSGAAARRFQNEVDVGMVGINVPIPVPLPFFSFTGWRGSFAG
DLHMYGRAGVQFYTQTKTVTAKWPAEDIRGISAPAATTSSSSGSKSSCGPKERLPGLDRVGAS
>CrALDH10A1
MASPVPPRLLYIGGEWVAPVKGGSLPVINPATEKEFARIPNATSEDVDAAVAAATAAFKSGHWSKTTGAY
RAKYLKAIATKLREHKAVLAKAETMDCGKPIDEASWDMDDVATCFDYYAGQAEALDGRNGAAPAIDVGMS
EFDVRVRREALGVVGLITPWNYPLLMAAWKVAPALAAGCTAVLKPSELASLTCLELAAIAAEVGLPPGVL
NVITGTGQDAGAPLSAHKGLAKVAFTGSAATGRLVAQAAAANIRPASMELGGKSALIVFEDADIEKAVEW
AMFGCFWTNGQICSSTSRLLVQEAVAPAFLQQLKKRAEAINVCDPLTEGCRLGPLVSEGQYRKVLSYVEA
GKAEGAQLLTGGGRPAGAPGTAGYWLAPTVFAGVKPHMRIWREEIFGPVLSVGTFSTEAEAVAAANDSEY
GLAGAVISADPDRCKRVAEALECGIVWINCSQPCFCYAPWGGIKNSGHGRELGEWGLDNFLSVKQITKYV
SPDIWGWYNPPSKL
>CrALDH11A1
MSAQEFYAPILASTGVYKFYIDGQWKESVSGKSVAISNPSTRQTAYQVQACTQDEVNKMFESAKVAQKAW
ARTPLYKRAEVLHKVASLMRQYAQPIADCLVKEVAKPSKDSLTEVVRSADLIDYTAEEGVRYLGEGQLLN
SDSFPGNARNKLCLVSKVPLGVVLAIPPFNYPVNLAVSKLAPALMAGNAVRGSARVSAAVCPLVLLSSCF
HAAGLPAGLLSVATGRGAEIGDFLTTHPDVNCISFTGGDTGISIAKKAGMVPLQMELGGKDVCIVCEDAD
LDLAAKHIIKGGFSYSGQRCTAVKLVLVAAPVADRLVAAVAAGVAKLSVGRPEDDCDITPVVSESSANFI
EGLAMDAKAKGATFVTGEWRREGNLIWPVLLDHVTADMRLAWEEPFGPVLPVMRVSSVEAAVEHCNKSKY
GLQGCVFTRDINAAIRISDAMETGTVQVNSAPARGPDHFPFQGFRDSGIGSQGIRNSLAMMIKTKSTVIN
LDKESYTLG

>CrALDH12A1
MLGLAPRGAALGSAAPVLPSLLHLARQFTEWATVDPKKLSGAAPASCQNLVAGRWTGSRESRQLPDPLNG
EPFISVPHTQVDEITPFVESLRAVPKSGLHNPLKNPQRYLLYGDVSFRVAAEMRKPAVEDFFARLIQRVA
PKSYDQALGEVRVTRKFFENFTGDQVRFLARGFTNPGDHAGQTSSGTRWPYGPVALITPFNFPLEIPALQ
LMGALYMGNKPLLHVDQRVSVVAEQLVRLLAHCGMPASDLDLLHGPGATVGEVIKRAEPRSTLFTGSQRV
AERLAVETHGKVFLEDAGFDWKIMGPDVSNVDYVAWQCDQDAYACSGQKCSAQSILFAHSNWVQAGLLNK
MAAAAAQRQLSDLTIGPVLTWTTEAILAHTNKLLQIPGAKVLFGGKPLTGHSIPAVYGAVQPTAVFVPLV
EALKPEHFGTVTTEVFGPFQVVTEYGDGQLPLVLEACERMTHHLTAAIVSNDINFIQHVLAHTVNGTTYA
GIRARTTGAPQNHWFGPAGDPRGAGIGTPEAIRMVWSCHREIITDFGPVPPASGLKQS
>CrALDH18D1
MQRLVLKPAKIAQLAEGIRAIAAQEEPLGRLLRKVEVAEGLILDKVTVPIGVLLVIFEARPDALPQIASL
AIRSGNGLLLKGGKEATHSNAALHKVIVEALGPMGSDLIALVTSREEIESLLALDDVVDLVIPRGSNALV
SHIKRNTRIPVLGHADGICHVYVDAAADLDSAIKIVLDAKTDYPAACNAVEKVLIHKDWVGKGGVKAIYE
ALHQAGVEVHAGDAVKPLLPELPPPPAPRHEYSALAVTLELVDNMEVAIDHIHKYGSAHTDCIVTTDGGR
AEAFLRGVDSACVFHNASTRFADGFRFGLGAEVGISTSRIHARGPVGVEGLLTTKWVLRGEGHVVAKDQG
VRFTHKVLAGGSEAGGSEAGGAGAGAGGRQGSTGRRRGCVVM
>CrALDH22B1
GQPVDKDVVPCYDPSTMQLLGHLPAMSASEVRSRIARCKAAQKEWRTSSFAQRRKLLRILLKFIIENVET
ICRVSARDSGKPMLDAILGEVVVTCEKIHWLSREGEAVLRPERRSAGILSFYKSARVEFHPVGVVGAIVP
WNYPFHNVLNPLTAALFAGDGLVIKVSEHASWSTGYYGRMISAALAAAGAPADLVQIVTGYGEAGSALVT
GGVDKVIFVGSTQVGKMVMRAAADTLTPVVLELGGKDAVIVTEDADLDNLVQVVLKAAFLNCGQNCAGGE
RFFVHEKIYDKFLERLTPLVAGLRQGNPLGDAPVDCGAMCMPGLAEKVHGLVTEAVSRGARLLAGGVLVP
SGERGGQFYPPTLLADVRPGMKIWEEEVFGPVMSVIKWSTDDEVVALANDCDFGLGSNVFAGSQARARSI
ASRLEAGMSSINDFATTYMCQSLPFGGVKHSGFDRFAGVEGLRGLCVPKAVAEDRFPLLMRSSIPPAWQL
PLPPHAVAFGVSLVTMFYG
>CrALDH24A1
MCGAMCQLLFLTKVLKAYGGCGLQLPPPSPAAAGLLEMLAGRLTNTAQPPPRVAASDNAEQLCGSTGSGG
SACLRVDDAASVGRAAAEAQRAQRTWCAASSPAARLAVMRGFASRLESGDGAVIAELLCSETGKPLRQAR
AEVRLAAARVRQACGLLQQLPFTLAVGDRVTTGLGPGEGLASSFASKPQRQRTEFVEWEPAGVVASITAW
NFPLVLACDVAVPALCLGNAVLAKPSEHAVLTGLALQEVWHRAGLPQASGNARGYTLVLFVSTHAHLLVE
VIGGPVNKAAADAFARLAAARVARLRAGDPAEASTELGPLTLGRAAAAGLQALVREAVEGGAKDWVAEQV
ADPAAATAATATWAVAEVESGGSFLAPAVLTGVKPGMRVLEDETFGPVLCVVAVSSPEEAAALMSRSRYG
LTAACYSRDEGVARRLLRAADVGTVFWNGCGEMPLALPWSGRRRSGLGFQLGGPEGYRAFLRPKSHVFTR
MFA
>PpALDH2B1
MHCRAVLATEQVEAPIKSPFPVQYTKLFINNSFVDSVSGKTFPSIDPRSEEVAVEVAQAAAEDVDRAVKA
ARKAFEEGPWPRMPGCERAGIMNRIADLLDEHKDELSALDTLNMGKVYDMARLGEAPLAIGLFRYYAGRW
CDKAQGMTLPTNGPFHAYTLHEPIGVVGSILPWNAPFYLLAMKVAPALACGNTIVLKPAQQSPLSALLIA
KLAAEAGLPDGVLNVVTGYGDTGMHIASHMDVDKVAFTGSTQVGRQIMQAAAQSNLKPVNLELGGKSPFI
IFGDADMDAAVESAHQAIFYNQGQMCVAGSRTFVHESVYDEYLERAKARAEKRVVGDPFKPGVEQGPQQA
DEAQFNKVMSYIRAGKDEGARLITGGERVGSKGYYIQPTIFSDVQDDMKICREEIFGPVMSVIKFKTVEE
VIQRSNQSEYGLGATVMSKNVDIINTVTRSLKAGIVWVNTYGILTPSAPFGGYKSSGFGRENGAYALANY
QQVKSVIMPICNPPYL
>PpALDH2B2
MARGKARTLLSTALRATSAGPARLHRNFCAAAAAEELENPTIAPVEVKLTKLLIDGEFVDAASGKTFPTI
DPRSEQVIAHVAEGDVEDVNRAVRAARKAFDHGPWPKMPPFQRQRILLKYADLLDQHADELAALETMDSG
KPYEQARYAELPLMSRQFRYFAGWADKIFGTTGPSDGIHAVQTLHEPIGVVGQIIPWNFPLVMYCWKVAP
ALAAGNTIVLKTAEQTPLSAILAGKLALEAGIPPGVLNIVSGYGPTAGASIAEHMDIDKVAFTGSTEVGK

LVMAAAARSNLKPVTLELGGKSPMIICEDANVDEAVELAHFALFFNMGQCCCAGSRTFVHESIYDEFVEK
SKARALKRVVGDPFRKGVEQGPQVDKDQFHKVLGYVESGMEQGANLITGGGRLGSKGYYIKPTIFTDVKE
GMKIFDEEIFGPVQSIAKFKTLDEVVQRANNTVYGLAAGIFSNNINTVNTLSRALRAGTIWVNCFDVFDA
TIPFGGYKQSGIGREKGKYVLESYTQVKAVVTPLHNPAWL
>PpALDH3H1
MEEDLEVGGRGAVRGAAELPRLVTEVREAYRNGRTRPAAWRVQQLNGIIRMISERESEIVQTLYTDLGKP
SHESYVTEVSLVRSACKLAIKELKKWMAPLKVSGSITTFPSWGAIVAEPLGVALVISAWNFPFLLSVEPL
VGAISAGCAMVLKPSEVAPATAALLSKLVPLYLDSSVIRVVEGGVDETTVLLDQQWDKIFYTGSPRVGRI
VMAAASKHLTPVTLELGGKCPVYIDRTADLKVGLRRIALGKWGCNNGQACIAPDYLLIDEIIASEVVDTL
IDVIETFYGKDPKTSQDLSRIVNTKHYSRLAGFLDDPKISSKIVHGGARDDNKLYISPTLVCDVPMDSTL
MSEEIFGPILPIIKVKGVQEAIDIISDRPKPLVAYVFTKNKEVEKRIVASISSGGMVVNDTIVHFLNPGL
PFGGVGESGMSSYHGKFSFDAFSHKKAVLYRNNLGDVPARFPPFTTMKQNFRRAIMDGHYLSAVISLTGL
KK
>PpALDH3H2
MKVGGRSADHGDERVLQLVAEVREAYMTMRTKPAEWRVQQLKGLLRMVIESESEIVEALYADLGKPAHES
YMSEISLVKSSCKLAIKELKKWMAPQRLCIMHVMFGALMVSGSMITFPSSASIVAEPLGVTLVISAWNFP
FLLSVDPLIGAISAGCAVVLKPSEVVYATPALLAKLIPLYMDNSVIRVVEGGVAETTLLLEQKWDKIFYT
GNPKVGRIVMAAASKHLTPVTLELGGKCPVYFDRSANLKVCLRRIAQGKWGNNNGQACISPDYILVDESI
ASELVDNLKEIIETFYGKNPISSTNLSRIVNTKHYLRLISFLEDPQICSKIVHGGERDEKKLYIAPTLVC
DALMDSFLMSEEIFGPILPIIKVQGEQEAIDIINARPKPLAAYVFTTNKAVEERMVKNVSSGGMVVNDTV
MHFVNPGLPFGGVGESGMGSYHGKFSFDAFSHKKAVLYRTSLGDFPARYPPFTTKKQNFLRCVLDGDYIG
AILSLTGLKK
>PpALDH3K1
MTTIDAPQIVSELRATLRTARTRPAQWRLDQIRAVLKLVNENEDDIYAALHSDLHKSNYESFLTEVNVLV
SACKSTMKNLHKWMAPEKKPIPLAVWPANASVISEPLGVALVISPWNFPLLLALDPVVGAIAAGCTVCLK
TSEIAPATSALLARLLPEYVDTEAIKVVEGSIPEVTALLEQKWDKIFYTGNAKVGRIIMGAAAKHLTPVT
LELGGKCPLFIDDSVDLKVASKRIMVGKYGSNAGQACISPDYVLVEEHFAPTLIKQLQKTLLEFYGPDPS
ASVDLARIINKNHFQRLSSMLDDPSIADKIVHGGERDEKSLYIAPTLIDNPPLDSPVMVEEIFGPMLPII
TVRNVDHALNIINDKPKPLEVYVFSNNKDLFNRFRDETSSGGIVMNDCVLQFIIPELPFGGVGESGTGAY
HGKATFDAFSHRKAVLVKNMGGDVFARYPPFTVRKQSLIKALLTGTIIDIILAALGWRK
>PpALDH3K2
MTIIKPHSVLDAAKVASCLRETFRTGRTRPSNWRLEQLHAIVKLIEENEDEICRALFADLHKPRHEALTM
ESLVVTASAKHAIKNLNKWRAPVKKETPYIVWPASAFILPEPLGVAFVIAPWNFPFLLAVDPVIGAIAAG
CTVCLKTSEVTPATSELLSTLVPKYLDNDAIKVVEGGVPEVTALLEQKWDKIFYTGNPKVGRIVMGAAAK
HLTPVTLELGGKCPVFIDDTVDLQLASRRIMAGKYGSNSGQACISPDYILVEEHLAPKLIKQFRSTLVEF
FGEDPRTSKDLSRIVNKNHFQRLSRLLDDPATADRIVHGGERDEDSLYIAPTLIEDPALDSPIMADEIFG
PLLPIITVRNVNAAIDLINDKPKALVVHLFSTNKDYVKMFTEETSSGGLVMNDCIMQFIVPELPFGGVGE
SGTGAYHGKASFDTFSHFKSIFNKSQSGDAPIRYPPFTLWKQAMLRAFLEGRFFKLIQLLLGLQK
>PpALDH3K3
MSTMNPVPRLDTSQVASSLRAAFRTGRTRSVKWRLEQLHAIVKLLEENEEDIYWALDADLRKPRHEAFLS
EIYTITASARYSIKNLHKWMAPAKKGVPLLAWPASASIVPEPLGVVFIMSPWNFPFMLAVDPLIGAISAG
CAVCLKASEITPTTSALLARLIPKLSARGYGVAGTEMGQDLLHWITMRAAARHLIPVTLELGGKCPLFID
DTVDLQVASRRIMSGKFSSNNGQACIAPDYILVEEHLAPKLIKQLQSTLVQFYGEDPRSTKDLARIVNKN
HFQRLSRLLDHPSTAENIIHGGERDEESLYIAPTLIKDPPLSSPIMEEEIFGPLLPIITDHVDAAVDLIN
DKPKALEIYLFSTRKDYAMKFAEETSSGGLVMNDCIVQFAVSELPFGGVGESGTGAYHGVASFNTFSHYK
SIFNKSLGVDVAFRYPPFTTQKQGMMRAFLEGRFVNFILLALGLQK
>PpALDH5F1
MVQLNEAGLFKSQGLIGDKWVDAENGHTLPVNNPATGEILTSVPFMGKREAEKAIAAASQAFTSWSKRTA

NDRSKILRQWFNLLIKNKDDLGKLIVLEQGKPLAEAVGEVIVYGAAFVEYYAEEAKRVYGDIIPSPFPEK
RMLVMKQPVGVVAAIAPWNFPLAMITRKVPPALAAGCTVVIKPSELTPLTALAAAELALQAGIPPGVVNV
VMGDAKGIGDAMLDSTEVRKITFTGSTGVGKMLLAGAGKTVKKVSLELGGNAPCIVFDDANLDVAVKGVL
AGKYRNSGQTCVCINKIFVQDGIYDKFAEAFAKAVSGLRAGNGLEPGITQGPLINETALEKVERHVQDAV
SKGAKVLVGGKRHSLGRTFYEPTILGNASDEMLIFREEVFGPVAPLVRFNTDEEAIKLANNSEFGLAAYA
FTENITRGWRVAESLEFGMVGLNEGLISTEVAPFGGMKQSGLGREGSKYGLDEYLEMKYVCLGNMAQPVG
>PpALDH5F2
MGFAQVGAMVQLKNMGLFKTQGLINGEWANALDNRTLAVNNPATGDVLANVPFMGKIDAEKAIAAASVAF
LPWSKRTAFDRCKLLRKWFDLILENKDDLAKLITLENGKPLTEANGEVVYGGGFVEYYAEEGKRVFGDII
PSPFPTKRMLVMKQAVGVVGAITPWNFPLAMITRKVAPALAAGCTIVLKPAELTPLTALAAAELAVQAGI
PSGVLNVVMGDAVEIGAAMMDSNEVRKITFTGSTHVGKLLMAAASKTVKKISLELGGNAPLIIFNDADIE
IAVQGALYGKYRNAGQTCVCVNRILVQDGIYDKFSEAFTKAVQKLRVGHGLDPGVTQGPLINEASLKKVE
AHVQDAVSKGAKVLVGGKRHSLGGTFYQPTVLCGCNDEMLIFREEVFGPVAPLMRFQTDEEAIKMANDTE
YGLAAYAYTENIEHGWRIAEALDYGMVGLNETLISSEVAPFGGTKQSGLGREGSNYGIDEYLELKYLCLG
NIKQPFMC
>PpALDH6B1
MSGIVRQALRASKCRGYQHIGSQLLNGSKVCSRNAALFRSAVVGAARPDAVIASSRVPWDYSSFSTSAVK
AVKETPALKAKNYVGGKFVESQSTEHIDVLNPATQEVVSRVPLTTHEEFETAVAVAKEAYKTWRKTPVTA
RQRVMLKLQELIRRDMDKLAMSVTLEQGKTLADARGDVFRGLEVVEQACGMANQQMGEFVENVSSGIDTY
SIRQPLGVCAGICPFNFPAMIPLWMFPMAVTTGNTFVLKPSEKDPGAAMLLAELATEAGLPPGVLNIVHG
TYDVVNQICDHPDIKAVSFVGSDVAGMHIYSRASATGKRVQCNMGAKNHAVIMPDADPEATLNALVGAAF
GAAGQRCMAISTAVFVGDSKRWEEGLRERGVKLKVTGGTEPGADLGPVISKQSKERICRLVESGAKAGAR
IVLDGRGIKVPGYEQGNFVGPTILADVTEDMECYKEEIFGPVLLCMEAASLQDAIEIVNRNKYGNGTAIF
TKSGAAARAFQHEVDAGQVGINVPIPVPLPFFSFTGSRGSFAGDLNFYGKAGVHFFTQIKTVTSQWKEKD
LHGVAMAFPTSGKV
>PpALDH7B4
MGVEFEREDYLFLNELGISPENLGCYGGGVWRANGPTVTSVNPSDNKPIASVREASLEDYEDSMRACAEA
RRMWMLTPAPKRGEIVRQIGDGLRDKLPLLGKLVSLEMGKILAEGIGEVQEFIDMCDYAVGLSRQLSGSI
IPSERPNHAMMEVWNPLGIVGVITAFNFPCAVLGWNACIALVCGNCVVWKGAPTTPLVTLATTKVIAEVL
ERNKLPGGIFTCVCGGAEIGSAIAYDTRIPLVSFTGSTKVGLLVQSIVHARHGKTLLELSGNNAIIVMDD
AVLPLAVRAVLFAAVGTAGQRCTTCRRLIVHEKVYDDMLAGLLKAYKQVKTGNAVDETTLCGPLHSKHSK
ACFEEGIKKIKAQGGKILTGGSVIDRDGNFVEPTVVEISHDAEIVREELFGPVLYVFKIKSLEEAIELNN
SVPQGLSSSIFTRNPETIFTWIGPTGSDCGIVNVNIPTNGAEIGGAFGGEKATGGGREAGSDSWKQYMHR
ATCTINYGNDLPLAQGINFGG
>PpALDH10A1
MGLHAGVDVPRRGLFIDGEWVDPVLGKRIPIVNPTTEETVGDIPAATSEDVDAAVKAAKEAFYRNKGKDW
AKAGGKHRATFLRAIAKRVAERKSELAKLESIDCGKPIDEAEWDMDDVSGCFEYYADLAEKLDERQYAPL
ELPMEQFKCNILREAIGVVGLITPWNYPMLMATWKVAAALAAGCTAILKPSELASVTCLELAGIAKDVGL
PRGVLNVVTGYGHEAGAPLASHSGVDKIAFTGSTATGRSVMSAASQLIKPVTLELGGKSPIIVFDDADVE
KAVEWAMFGAFWTNGQICSATSRLLLQEGIADEFLKKLGIWASSIKVSDPLEKDCRLGPLVSEGQYEKVQ
KFVKVALDEGATLVCGGKRPDHLTTGYFLAPTVLSNVKPHMQIWTDEVFGPVLAVSTFKTEEEALALAND
TQYGLAGAVISKDDDRCKRVSEALEVGIVWINCSQPCFCQAPWGGNKRSGFGRELGEWGLENYLSVKQLT
RYISTDDWGWYPKPSKL
>PpALDH11A5
MFFSSAVGSETFKELLDGDTYKFYSNGEWQVSTSGKSIGVLNPTTLKLQFKVQACTPEEVNSCVEKAKVA
QKGWAKTPLWKRAEALHRFASILKEQKAPIAECLVAEVAKCLKDAITEVVRSGDLLAYAAEEGVRILGKG
DFLVSDSFPGNGRNKYCLASKIPLGVVLAIPPFNYPVNLAVSKLGPALVAANAVILKPPTQGGVSCLHMI
QCLHMAGFPKGLVSAITGKGSIIEDLLTTHPSISCIRHIPQLPQPFYICHFFTGGETGMAICRKAGMIPL

QMELGGKDTCLVLEDADLELAANNIIKGGYSYSGQRCTAVKVICVMESVADDLVKRVVEKMKKLTVGPPD
KDCDITPVISESSANFIQGLVDDARQQGAKFHQEWRREGNLIWPMLVDNVKPDMRIAWEEPFGPVIPVLR
IKSPEEGIHHCNANSFALQGCVFTRDIDKAILMSNAMEGPDHFPFQGLKDSGIGSQGITNSINMMTKTKS
TVINLPVESYTIG
>PpALDH11A1
MAGKGVFKEILDGDVFKYYVDGEWRRSCSGQFINVQNPSTRKPMFRVQACTQDEVNRCIDSAKAAQKIWA
RTPLWKRAEALHRCAAIMKEQKAPIADCLVKEVAKALKDAVVEVVRSGDLLSYTAEEGIRILSEGKFLVS
DPFPGNERNKYCLSSKIPLGVILAIPPFNYPVNLAVSKMGPALIAGNAVVLKPPTQGAVSCLHMVHTFHL
AGFPKGLISAVTGKGADIGDFLTMHPGINCIRPVSRPLFTGGCETGIAISKKAGMIPLQMELGGKDSCII
LEDADLELAATNIIKGGFSYSGQRCTAVKVVLAMECIADQLVCKVNAKLAKLTIGMPEDDCDITPVISET
SANFIEGLVHDARHKGARLHQEWRREGNLIWPILIDHVRPDMRIAWEEPFGPILPVIRIKTVEEGIHHCN
ANNFALQGCIFTRSFDKAIMISDAMESGTIQINSAPGRGPDHFPFQGLRDSGVGSQGITNSINMMCKIKS
TVMNFPAATYTMG
>PpALDH11A2
MAGQGFFQDIFDGEAFKYYADGEWKVSSSRASVSITNPSTLKTQFKVQACTQDEVNKAIESAESAQKLWA
KTPLWKRAEALHKFAGILKDQKNAIADVLVKEIAKPLKDAVTEVVRSGDLVSYSAEEGIRLLAEGKFLVS
DSFPGNGRNKYCLASKIPIGVVLAIPPFNYPINLAVSKIAPALIAGNAVVLKPPTQGAVAALHIVHCIHL
AGFPKGLVAAITGKGSEIGDLLTMHPGINCISFTGGDTGIAISRKAGMIPLQMELGGKDCCIVLEDADLE
LAANNVIKGGYSYSGQRCTAIKVICVMESVAEELVQKIVQRISKLKVGMPEDNCDITPVVSQSSANFIQG
LVEDAQKKGAKFHQEWKREGNLIWPILIDHVTPDMRIAWEEPFGPVIPVIRIKTVEEGIHHCNANNFALQ
GCIFTKDINKAILISNAMESGTIQINAAPARGPDHFPFQGLRDSGIGSQGITNSIIMMTKTKSTVINLPV
ESYTMG
>PpALDH11A3
MAGTGFYESILDNDVFKYYADGEWKVSSSGKSVGITNPSTLKVQYKVQACTQDEVNKAVESAQAAQKIWA
KTPLWKRAEALHRFAAILKDNKNEIAEALVKEIAKPHKDALTEVVRSGDLISYSAEEGIRILAEGKFLVS
DSFPGNGRNKYCLASKIPLGVVLAIPPFNYPVNLAVSKIAPALIAGNAVVLKPPTQGAVSALHMVHCLHM
AGFPKGLVSAITGKGSEIGDLMTMHPGINCISFTGGDTGIAISRKAGMVPLQMELGGKDCCLVLEDADLE
LAANNVIKGGYSYSGQRCTAVKVICVMESVAEELVSKIVQKMTKLTVGMPEDNCDITPVVSQSSANFIQG
LVEDAQAKGAKFHQEWKREGNLIWPLLIDNVTPDMRIAWEEPFGPVIPVIRIKTVEEGIHHCNANNFALQ
GCVFTKDINKAILVSDAMESGTIQINAAPARGPDHFPFQGLRDSGIGSQGITNSIQMMTKTKSTVINLPT
ESYTMG
>PpALDH11A4
MAGQGFFKDILDGDVFKYYADGEWKVSSSGRSVGITNPSTLKVQYKVQACTQEEVNKSVESAHAAQKIWA
KTPLWKRAEALHRFAGILKDQKNPIAEALVKEIAKPQKDAVTEVVRSGDLISYSAEEGIRILAEGKFLVS
DSFPGNGRNKYCLASKIPLGVVLAIPPFNYPVNLAVSKIAPALIAGNAVILKPPTQGAVSALHMVHCLHM
AGFPKGLVSAITGKGSEIGDLMTMHPGINCISFTGGDTGIAISRKAGMVPLQMELGGKDCCLVLEDADLE
LAANNVIKGGYSYSGQRCTAVKVICVMESVAEELVTKIVEKMTKLKVGMPEDNCDITPVVSQSSANFIQG
LVEDAKAKKAKFHQEWKREGNLIWPLLIDNVTADMRIAWEEPFGPVIPVIRIKTVEEGIHHCNANNFGLQ
GCVFTKDINKAILISDAMESGTIQINAAPARGPDHFPFQGLRDSGIGSQGITNSIQMMTKTKSTVINLPT
ESYTMG
>PpALDH12A1
MQRCVVKRVGAVYGRSRSVIGKASSECLHPSFTHRSSSTLLSDHRPQSSLSFASCDADKLSEAHQYQMHN
LVQGKWEQTSKSIELLDPLNGEKFISVPDTSIDEISPFVQSLRACSKSGLHNPLKNPERYLLYGDIMAKA
AHLLKQPQVETFFARLIQRVAPKSFAQAVGEVTVTQKFLENFSGDQVRFLARSFVVPGNYQGQQSNGMRW
PYGPVAIITPFNFPLEIPALQALGALFMGNKPILKVDSKVSIVMEQFIRLLHKCGMPPTDMDFINSDGPV
MNKLLLEAEPKTTLFTGSSKVAEKLALDLKGRVKLEDAGFDWKILGPDVQNEDYVAWVCDQDAYACSGQK
CSAQSILFMHENWANQNFLERLKKLASKRKLEDLTVGPVLTVTTERMLDHVKNLLAIPGARVEFGGKPLT
NHTIPDVYGALEPTAVFVPLKEILRNEENFALATTEIFGPFQILTEYKHEDLPLVFEACERMHAHLTAAV

VSNDVHFLQEVLSNTVNGTTYAGIRARTTGAPQNHWFGPAGDPRGAGIGTPEAIKLVWSCHREIIQDVGP
IPNGWSTPQCT
>PpALDH18B1
MDRSRIFIRDAKRVVIKIGTAVVTRHDGRLALGRLGAICEQVKELITDGIEVIFVTSGAVGVGRQKLRHQ
RMMNSRRVIFVDLQKPQVELDGKPCAAVGQSGLMALYDSLFSQLDVASSQLLVTDNDFKDPEFRQQLSET
VNSLLALRVVPIFNENDAISTRKSPYFDSTGIFWDNDSLAALLALELQADLLILLSDVEGLYTGPPSEPK
SQLIHTYLKEKHDDMVTFGEKSRVGRGGMTAKVYAAWQAASAGIPVVISSGCVADGLQRVMRGDHVGTLF
HRDAHQWVDLKETGARNMAVAAREGSRRLQGLTSEERKSILHAVADALLANEAIIKAENDADVELAQMTG
VSKALVGRLTIKPGKIAALASSLRTLADMKEPIGEVLKRTEVAEGLTLEKTSCPLGVVLVVFESRPDALV
QIASLAIRSGNGLLLKGGKEAARSNAILHKVITEALPKSVGPNLIGLVTSRDEIPDLLKLDDVIDLVIPR
GSNKLVAQIKATTKIPVLGHADGVCHVYVDTAADLEKAKNIVIDSKVDYPAACNALETLLVHEDLVATGG
LEMLAFALQSAGVTLYGGARASGILKLPRASSYHIEYSALSCTVEVVKDVQEAIDHIHEHGSAHTDCIVT
ENHITAETFLHHVDSAAVFHNASTRFSDGARFGLGAEVGISTGRIHARGPVGVEGLLTTRWLLRGSGQLV
NGDKGVQYTHKKLPIGEDEGLAALATANQSLENGNSAVKTTSNGAVPS
>PpALDH21A1
MTLGHMVQKAKESSGDVTPKKYNIFLASKPVDGDRKWLDVTNKYTNDVAAKVPQATHKDIDDAIDAAVAA
APAMAAMGAYERKAVLEKVVAELKNRFEEIAQTLTMESGKPIKDARGEVTRTIDTFQVAAEESVRIYGEH
IPLDISARNKGLQGIVKKFPIGPVSMVSPWNFPLNLVAHKVAPAIAVGCPFVLKPASRTPLSALILGEIL
HKIEELPLGAFSILPVSREDADMFTVDERFKLLTFTGSGPIGWDMKARAGKKKVVMELGGNAPCIVDDYV
PDLDYTIQRLINGGFYQGGQSCIHMQRLYVHERLYDEVKEGFVAAVKKLKMGNPFEEDTYLGPMISESAA
KGIEDWVKEAVAKGGKLLTGGNRKGAFIEPTVIEDVPIEANARKEEIFGPVVLLYKYSDFKEAVKECNNT
HYGLQSGIFTKDLNKAFYAFEHMEVGGVILNDSPALRVDSQPYGGLKDSGIQREGVKYAMDDMLETKVLV
MRNVGTL
>PpALDH23A1
MFDVVNPATGKVIGELLIESKDEVVSKFEALAAGQKKWRSVPLVERRAMLERFNELLRLNMPVLAKTLST
EMGKPIAQAKNEVRATVDRVRFYLENYEKVLKESCVLETSILKEKVVYEPLGVVANISAWNYPYFVSTNV
FAAALLTGNAVLYKPSEHATLTGMEITNLLYEAGVPKNVFAMTTGKGETGAAVASLKGLGGLFFTGSNKT
GLEIAKQAAPNLVKLQLELGGKDPVYVRADVADVGAAAASIADGAFYNCGQSCCSVERIYVDKRIYNEFL
SAFIKNVMAFKVGDPLKPDTYIGPVARQPHLPYLAAQVQDAISKGARASSHTHLESNQGGFYFPPTVLSD
VNHTMDVMKEESFGPLIGIQAVENDAEALALMNDTTYGLTASVYCKHNQDAENILRELDVGTGYWNCCDR
VSPRLPWSGRRGSGLGVTLGMDGLRSFVKPKGIVFQSPSNKD
>MmALDH1A1
MSSPAQPAVPAPLADLKIQHTKIFINNEWHNSVSGKKFPVLNPATEEVICHVEEGDKADV
DKAVKAARQAFQIGSPWRTMDASERGRLLNKLADLMERDRLLLATMEALNGGKVFANAYL
SDLGGCIKALKYCAGWADKIHGQTIPSDGDIFTYTRREPIGVCGQIIPWNFPMLMFIWKI
GPALSCGNTVVVKPAEQTPLTALHLASLIKEAGFPPGVVNIVPGYGPTAGAAISSHMDVD
KVAFTGSTQVGKLIKEAAGKSNLKRVTLELGGKSPCIVFADADLDIAVEFAHHGVFYHQG
QCCVAASRIFVEESVYDEFVKRSVERAKKYVLGNPLTPGINQGPQIDKEQHDKILDLIES
GKKEGAKLECGGGRWGNKGFFVQPTVFSNVTDEMRIAKEEIFGPVQQIMKFKSVDDVIKR
ANNTTYGLAAGLFTKDLDKAITVSSALQAGVVWVNCYMMLSAQCPFGGFKMSGNGRELGE
HGLYEYTELKTVAMKISQKNS
>MmALDH1A2
MTSSEIAMPGEVKADPAALMASLQLLPSPTPNLEIKYTKIFINNEWQNSESGRVFPVCNP
ATGEQVCEVQEADKVDIDKAVQAARLAFSLGSVWRRMDASERGRLLDKLADLVERDRATL
ATMESLNGGKPFLQAFYIDLQGVIKTLRYYAGWADKIHGMTIPVDGDYFTFTRHEPIGVC
GQIIPWNFPLLMFTWKIAPALCCGNTVVIKPAEQTPLSALYMGALIKEAGFPPGVVNILP
GYGPTAGAAIASHIGIDKIAFTGSTEVGKLIQEAAGRSNLKRVTLELGGKSPNIIFADAD
LDYAVEQAHQGVFFNQGQCCTAGSRIFVEESIYEEFVKRSVERAKRRIVGSPFDPTTEQG

PQIDKKQYNKVLELIQSGVAEGAKLECGGKGLGRKGFFIEPTVFSNVTDDMRIAKEEIFG
PVQEILRFKTMDEVIERANNSDFGLVAAVFTNDINKALMVSSAMQAGTVWINCYNALNAQ
SPFGGFKMSGNGREMGEFGLREYSEVKTVTVKIPQKNS
>MmALDH1A3
MATTNGAVENGQPDGKPPALPRPIRNLEVKFTKIFINNDWHESKSGRKFATYNPSTLEKI
CEVEEGDKPDVDKAVEAAQAAFQRGSPWRRLDALSRGQLLHQLADLVERDRAILATLETM
DTGKPFLHAFFVDLEGCIKTFRYFAGWADKIQGRTIPTDDNVVCFTRHEPIGVCGAITPW
NFPLLMLAWKLAPALCCGNTVVLKPAEQTPLTALYLASLIKEVGFPPGVVNIVPGFGPTV
GAAISSHPQINKIAFTGSTEVGKLVREAASRSNLKRVTLELGGKNPCIVCADADLDLAVE
CAHQGVFFNQGQCCTAASRVFVEEQVYGEFVRRSVEFAKKRPVGDPFDAKTEQGPQIDQK
QFDKILELIESGKKEGAKLECGGSAMEDRGLFIKPTVFSDVTDNMRIAKEEIFGPVQPIL
KFKNLEEVIKRANSTDYGLTAAVFTKNLDKALKLAAALESGTVWINCYNAFYAQAPFGGF
KMSGNGRELGEYALAEYTEVKTVTIKLEEKNP
>MmALDH1A7
MSSPAQPAVPAPLANLKIQHTKIFINNEWHDSVSSKKFPVLNPATEEVICHVEEGDKADV
DKAVKAARQAFQIGSPWRTMDASERGRLLNKLADLMERDRLLLATMESMNAGKVFAHAYL
LDVEISIKALQYFAGWADKIHGQTIPSDGNIFTYTRREPIGVCGQIIPWNGPLIIFTWKL
GPALSCGNTVVVKPAEQTPLTALHMASLIKEAGFPPGVVNIVPGYGPTAGGAISSHMDID
KVSFTGSTEVGKLIKEAAGKSNLKRVTLELGGKSPCIVFADADLDSAVEFAHQGVFFHQG
QICVAASRLFVEESIYDEFVRRSVERAKKYILGNPLNSGINQGPQIDKEQHNKILGLIES
GKKEGAKLECGGGRWGNKGFFVQPTVFSNVTDEMRIAKEEIFGPVQQIMKFKSMDDVIKR
ANNTTYGLAAGVFTKDLDKAITVSSALQAGMVWVNCYLAVPVQCPFGGFKMSGNGRELGE
HGLYEYTELKTVAMQISQKNS
>MmALDH1B1
MLTARLLLPRLLCLQGRTTSYSTAAALPNPIPNPEICYNKLFINNEWHDAVSKKTFPTVN
PTTGEVIGHVAEGDRADVDLAVKAAREAFRLGSPWRRMDASERGRLLNRLADLVERDRVY
LASLETLDNGKPFQESYVLDLDEVIKVYRYFAGWADKWHGKTIPMDGEHFCFTRHEPVGV
CGQIIPWNFPLVMQGWKLAPALATGNTVVMKVAEQTPLSALYLASLIKEAGFPPGVVNII
TGYGPTAGAAIAQHMDVDKVAFTGSTEVGHLIQKAAGESNLKRVTLELGGKSPSIVLADA
DMEHAVDQCHEALFFNMGQCCCAGSRTFVEESIYREFLERTVEKAKQRKVGNPFELDTQQ
GPQVDKEQFERILGYIRLGQKEGAKLLCGGERLGERGFFIKPTVFGDVQDGMRIAKEEIF
GPVQPLFKFKKIEEVIQRANNTRYGLAAAVFTRDLDKAIYFTQALQAGTVWVNTYNIVTC
HTPFGGFKESGNGRELGEDGLRAYTEVKTVTIKVPEKNS
>MmALDH1L1
MKIAVIGQSLFGQEVYCQLRKEGHEVVGVFTIPDKDGKADPLGLEAEKDGVPVFKFPRWR
ARGQALPEVVAKYQALGAELNVLPFCSQFIPMEVINAPRHGSIIYHPSLLPRHRGASAIN
WTLIHGDKKGGFTIFWADDGLDTGDLLLQKECDVLPDDTVSTLYNRFLFPEGIKGMVQAV
RLIAEGTAPRRPQPEEGATYEGIQKKETAMINWDQPAEAIHNWIRGNDKVPGAWTEACGQ
KLTFFNSTLNTSGLVAQGEALPIPGAHRPGLVTKAGLILFGNDDRMLLVKNIQLEDGKMM
PASQFFKGSASSALELTEEELATAEAVRSSWMRILPNVPEVEDSTDFFKSGAASVDVVRL
VEEVKELCDGLELENEDVYMATTFGDFIQLLVRKLRGEDGESECVINYVEKAVKKLTLQM
PYQLFIGGEFVDAEGAKTYSTINPTDGSVICQVSLAQVSDVDKAVAAAKEAFENGLWGKI
NARDRGRLLYRLADLMEQHQEELATIEALDAGAVYTLALKTHVGMSIQTFRYFAGWCDKI
QGATIPINQARPNRNLTLTKKEPVGVCGIVIPWNYPLMMLSWKTAACLAAGNTVVIKPAQ
VTPLTALKFAELTLKAGIPKGVVNILPGSGSLVGQRLSDHPDVRKIGFTGSTEVGKHIMK
SCALSNVKKVSLELGGKSPLIIFADCDLNKAVQMGMSSVFFNKGENCIAAGRLFVEDSIH
DQFVQKVVEEVGKMKIGNPLDRDTNHGPQNHEAHLRKLVEYCQRGVKEGATLVCGGNQVP
RPGFFFQPTVFTDVEDHMYIAKEESFGPIMIISRFADGDVDAVLSRANATEFGLASGVFT

RDINKALYVSDKLQAGTVFVNTYNKTDVAAPFGGFKQSGFGKDLGEAALNEYLRIKTVTF
EY
>MmALDH1L2
MLWRGSQALRHFSTSRVYFKNKLKLALIGQSLFGQEVYSQLLKEGHRVVGVFTVPDKDGK
ADPLALAAEKDGTPVFKFPRWRLKGKTIKEVAEAYQSVGAELNVLPFCTQFIPMDVIDSP
KHGSIIYHPSLLPRHRGASAINWTLIMGDKKAGFSVFWADDGLDTGPILLQRSCDVKPND
TVDSLYNRFLFPEGIKAMVEAVQLIADGKAPRTPQPEEGATYEGIQKKENAEVSWDQPAE
GLHNWIRGHDKVPGAWAEINGQMVTFYGSSLLTSSVPSGEPLDIRGAKKPGLVTKNGLVL
FGNDGKALMVRNLQFEDGKMIPASQYFSAGETSVVELTAEELKVAETIKVIWARILSNTP
VIEDSTDFFKSGASSMDVVRLVEEIRQSCGGLQLQNEDVYMATKFGDFIQKVVRRLRGED
EEAEMVVDYVSKEVNGMTVKIPYQCFINGQFVDAEDGETYATVNPTDGTTICRVSYASLA
DVDRAVAAAKDAFENGEWGRMNARDRGRLMYRLADLMEENQEELATIEALDSGAVYTLAL
KTHIGMSVQTFRYFAGWCDKIQGSTIPINQARPNYNLTFTKKEPLGACAIIIPWNYPLMM
LAWKSAACLAAGNTLVLKPAQVTPLTALKFAELTVKAGFPKGVINIIPGSGGVAGQRLSQ
HPDIRKLGFTGSTSVGKQIMKSCAVSNLKKVSLELGGKSPLIIFSDCDLEKAVRMGMGAV
FFNKGENCIAAGRLFVEEAIHDEFVTRVVEEIKKMKIGDPLDRSTDHGPQNHRAHLEKLL
QYCETGVQEGATLVYGGRQVQRPGFFMEPTVFTGVEDHMYLAKEESFGPIMVISKFQNGD
IDGVLQRANNTEYGLASGVFTRDINKAMYVSDKLEAGTVFINTYNKTDVAAPFGGMKQSG
FGKDLGEEALNEYLKIKTVTLEY
>MmALDH2
MLRAALTTVRRGPRLSRLLSAAATSAVPAPNHQPEVFCNQIFINNEWHDAVSRKTFPTVN
PSTGEVICQVAEGNKEDVDKAVKAARAAFQLGSPWRRMDASDRGRLLYRLADLIERDRTY
LAALETLDNGKPYVISYLVDLDMVLKCLRYYAGWADKYHGKTIPIDGDFFSYTRHEPVGV
CGQIIPWNFPLLMQAWKLGPALATGNVVVMKVAEQTPLTALYVANLIKEAGFPPGVVNIV
PGFGPTAGAAIASHEGVDKVAFTGSTEVGHLIQVAAGSSNLKRVTLELGGKSPNIIMSDA
DMDWAVEQAHFALFFNQGQCCCAGSRTFVQENVYDEFVERSVARAKSRVVGNPFDSRTEQ
GPQVDETQFKKILGYIKSGQQEGAKLLCGGGAAADRGYFIQPTVFGDVKDGMTIAKEEIF
GPVMQILKFKTIEEVVGRANDSKYGLAAAVFTKDLDKANYLSQALQAGTVWINCYDVFGA
QSPFGGYKMSGSGRELGEYGLQAYTEVKTVTVKVPQKNS
>MmALDH3A1
MSNISSIVNRARDAFNSGKTRPLQFRVEQLEALQRMINENLKGISKALASNLRKNEWTSY
YEEVAHVLDEIDFTIKGLSDWAEDEPVAKTRQTQEDDLYIHSEPLGVVLVIGAWNYPFNL
TIQPMVGAIAAGNAVVLKPSEVSDHMADLLSTLIPQYMDKDLYPVIKGGVPETTELLKEK
FDHIMYTGSTAVGKIVMAAAAKHLTPVTLELGGKSPCYVDKDCDLDVACRRIAWGKFMNS
GQTCVAPDYILCDPSIQNEIVEKLKKSLKDFYGEDAKQSHDYGRIINDRHFQRVINLIDS
KKVAHGGTWDQPSRYIAPTILVDVDPQSPVMQEEIFGPVMPIVCVRSLDEAIKFINQREK
PLALYVFSNNDKVIKKMIAETSSGGVTANDVIVHITVPTLPFGGVGNSGMGAYHGKKSFE
TFSHRRSCLVRSLRNEEANKARYPPSPAKMPRH
>MmALDH3A2
MERQVLRLRQAFRSGRSRPLRFRLQQLEALRRMVQEREKEILAAIAADLSKSELNAYSHE
VITILGEIDFMLGNLPELASARPAKKNLLTMMDEAYVQPEPLGVVLIIGAWNYPFVLTMQ
PLVGAIAAGNAAIVKPSELSENTAKILAELLPQYLDQDLYAIVNGGIPETTELLKQRFDH
ILYTGNTAVGKIVMEAAAKHLTPVTLELGGKSPCYIDRDCDLDVACRRIAWGKYMNCGQT
CIAPDYILCEASLQNQIVQKIKETVKDFYGENIKASPDYERIINLRHFKRLQSLLKGQKI
AFGGEMDEATRYLAPTILTDVDPNSKVMQEEIFGPILPIVSVKNVDEAINFINDREKPLA
LYVFSRNNKLIKRVIDETSSGGVTGNDVIMHFTVNSLPFGGVGASGMGAYHGKYSFDTFS
HQRPCLLKGLKGESVNKLRYPPNSESKVSWAKFFLLKQFNKGRLGMLLFVCLVAVAAVIV
KDQL

>MmALDH3B1
MDSFEDKLQQLREAFKEGRTRSAEFRAAQLQGLSHFLRDNKQQLQEALAQDLHKSAFEAE
VSEIAISQAEVDLALRNLRSWMKDEKVSKNLATQLDSAFIRKEPFGLVLIIVPWNYPINL
TLVPLVGAIAAGNCVVLKPSEISKATEKILAEVLPRYLDQSCFTVVLGGRQETGQLLEHK
FDYIFFTGNAYVGKIVMAAAAKHLTPITLELGGKNPCYVDDNCDPQIVANRVAWFRYFNA
GQTCVAPDYILCSQEMQERLVPALQNAITRFYGDNPQTSPNLGRIINQKHFKRLQGLLGC
GRVAIGGQSDEGERYIAPTVLVDVQETEPVMQEEIFGPILPLVTVRSLDEAIEFMNRREK
PLALYAFSKRSQVIKQVLARTSSGGFCGNDGFMHMTLSSLPFGGVGTSGMGRYHGKFSFD
TFSNQRACLLRSPGMEKINDLRYPPYSSRNLRVLLVAMEERCCSCTLL
>MmALDH3B2
MSAAETGSEPSQGAGPSEATLHSLREAFNAGRTRPTEFRTAQLRSLGRFLQENKELLQDA
LAKDVGKSGFESDMSEIILCENEVDLALKNLQTWMKDEPVSTNLLTKLSSAFIRKEPFGL
VLIIAPWNYPVNLMIIPLVGAIAAGNCVVLKPSEISKNTEKVLAELLPQYLDQSCFAVML
GGPEETRQLLEHKFDYIFFTGSPRVGKIVMTAAAKHLTPITLELGGKNPCYVDDNCDPQT
VANRVAWFRYFNAGQTCVAPDYILCSQEMQERLVPALQNSITRFYGDNPQTSPNLGRIIN
QKHFKRLQGLLGCGRVAIGGQSDEGERYIAPTVLVDVQETEPVMQEEIFGPILPLVTVRS
LDEAIEFINRREKPLALYAFSNNNQVVNQMLERTSSGGFGGNDGFLYLTLPALPLGGVGN
SGMGRYHGKFSFDTFSHHRACLLRSPGMEKLNDLRYPPYGPWNQQLISWAIGSRSCTLL
>MmALDH4A1
MLPLPSLRRSLLSHAWRGAGLRWKHTSSLKVTNEPILAFSQGSPERDALQKALKDLKGQM
EAIPCVVGDEEVWTSDIQYQLSPFNHAHKVAKFCYADKALLNRAIDAALAARKEWDLKPM
ADRAQVFLKAADMLSGPRRAEVLAKTMVGQGKTVIQAEIDAAAELIDFFRFNAKFAVELE
GEQPISVPPSTNHTVYRGLEGFVAAISPFNFTAIGGNLAGAPALMGNVVLWKPSDTAMLA
SYAVYRILREAGLPPNIIQFVPADGPTFGDTVTSSEHLCGINFTGSVPTFKHLWRQVAQN
LDRFRTFPRLAGECGGKNFHFVHSSADVDSVVSGTLRSAFEYGGQKCSACSRLYVPKSLW
PQIKGRLLEEHSRIKVGDPAEDFGTFFSAVIDAKAFARIKKWLEHARSSPSLSILAGGQC
NESVGYYVEPCIIESKDPQEPIMKEEIFGPVLTVYVYPDDKYRETLQLVDSTTSYGLTGA
VFAQDKAIVQEATRMLRNAAGNFYINDKSTGSVVGQQPFGGARASGTNDKPGGPHYILRW
TSPQVIKETHKPLGDWRYSYMQ
>MmALDH5A1
MATCFLLRSFWAARPALPPPGRFRPEPAGTPRRSYASGPGGLHADLLRGDSFVGGRWLPA
PATFPVYDPASGAKLGTVADCGVPEARAAVRAAYDAFNSWKGVSVKERSLLLRKWYDLMI
QNKDDLAKIITAESGKPLKEAQGEILYSALFLEWFSEEARRIYGDIIYTSAKDKRGLVLK
QPVGVAAIITPWNFPSAMITRKVGAALAAGCTVVVKPAEDTPYSALALAQLANQAGIPAG
VYNVIPCSRNKAKEVGEVLCTDPLVSKISFTGSTATGKILLHHAANSVKRVSMELGGLAP
FIVFDSANVDQAVAGAMASKFRNAGQTCVCSNRFLVQRGIHDSFVTKFAEAMKKSLRVGN
GFEEGTTQGPLINEKAVEKVEKQVNDAVAKGATVVTGGKRHQSGGNFFEPTLLSNVTRDM
LCITEETFGPLAPVIKFDKEEEAVAIANAAEVGLAGYFYSQDPAQIWRVAEQLEVGMVGV
NEGLISSVECPFGGVKQSGLGREGSKYGIDEYLEVKYVCYGGL
>MmALDH6A1
MAAAVAAAAAMRSRILQVSSKVNATWYPASSFSSSSVPTVKLFIDGKFVESKSDKWIDIH
NPATNEVVGRVPQSTKAEMDAAVESCKRAFPAWADTSILSRQQVLLRYQQLIKENLKEIA
RLITLEQGKTLADAEGDVFRGLQVVEHACSVTSLMLGETMPSITKDMDLYSYRLPLGVCA
GIAPFNFPAMIPLWMFPMAMVCGNTFLMKPSERVPGATMLLAKLLQDSGAPDGTLNIIHG
QHDAVNFICDHPDIKAISFVGSNQAGEYIFERGSRNGKRVQANMGAKNHGVVMPDANKEN
TLNQLVGAAFGAAGQRCMALSTAILVGEAKKWLPELVDRAKNLRVNAGDQPGADLGPLIT
PQAKERVCNLIDSGTKEGASILLDGRRIKVKGYENGNFVGPTIISNVKPSMTCYKEEIFG
PVLVVLETETLDEAIKIVNDNPYGNGTAIFTTNGATARKYAHMVDVGQVGVNVPIPVPLP

MFSFTGSRSSFRGDTNFYGKQGIQFYTQLKTITSQWKEEDATLSSPAVVMPTMGR
>MmALDH7A1
MWRVPRRLCVQSVKTSKLSGPWSRPAAHMSTLLIHHPQYAWLQDLGLREDNEGVYNGSWG
GRGEVITTYCPANNEPIARVRQASLKDYEETIGKAKKAWNIWADIPAPKRGEIVRKIGDA
FREKIQLLGRLVSLEMGKILVEGIGEVQEYVDVCDYAAGLSRMIGGPTLPSERPGHALIE
MWNPLGLVGIITAFNFPVAVFGWNNAIALITGNVCLWKGAPTTSLVSVAVTKIIAQVLED
NLLPGAICSLVCGGADIGTTMARDERVNLLSFTGSTQVGKEVALMVQERFGKSLLELGGN
NAIIAFEDADLSLVVPSVLFAAVGTAGQRCTTVRRLFLHESIHNEVVDRLRSAYSQIRVG
NPWDPNILYGPLHTKQAVSMFVRAVEEAKKQGGTVVYGGKVMDHPGNYVEPTIVTGLAHD
APIVHQETFAPILYVFKFQDEEEVFEWNNEVKQGLSSSIFTKDLGRIFRWLGPKGSDCGI
VNVNIPTSGAEIGGAFGGEKHTGGGRESGSDAWKQYMRRSTCTINYSTSLPLAQGIKFQ
>MmALDH8A1
MAGKRELLMLENFIGGKFLPCNSYIDSYDPSTGEVYCKVPNSGKEEIEAAVEAAREAFPA
WSSRSPQERSLVLNRLADVLEQSLEELAQAESKDQGKTLTLARTMDIPRSVLNFRFFASS
NLHHVSECTQMSHLGCMHYTVRTPVGIAGLISPWNLPLYLLTWKIAPAIAAGNTVIAKPS
EMTSVTAWMFCKLLDKAGVPPGVINIVFGTGPRVGEALVSHPEVPLISFTGSQPTAERIT
QLSAPHCKKLSLELGGKNPAIIFEDANLEECIPATVRSSFANQGEICLCTSRIFVQRSIY
SEFLKRFVEATRKWKVGVPSDPSANMGALISKAHLEKVRSYVLKAQTEGARILCGEGVDQ
LSLPLRNQAGYFMLPTVITDIKDESRCMTEEIFGPVTCVVPFDSEEEVITRANSVRYGLA
ATVWSKDVGRIHRVAKKLQSGLVWTNCWLIRELNLPFGGMKSSGIGREGAKDSYDFFTEI
KTITIKY
>MmALDH9A1
MSTGTFVVSQPLNYRGGARVEPVDASGTEKAFEPATGRVIATFACSGEKEVNLAVENAKA
AFKLWSKKSGLERCQVLLEAARIIKERKDEIATVETINNGKSIFEARLDVDTCWQCLEYY
AGLAASMAGEHIQLPGGSFGYTRREPLGVCVGIGAWNYPFQIACWKSAPALACGNAMIFK
PSPFTPVSALLLAEIYTKAGAPPGLFNVVQGGAATGQFLCHHREVAKISFTGSVPTGVKI
MEMSAKGVKPITLELGGKSPLIIFSDCNMENAVKGALMANFLTQGQVCCNGTRVFVQKEI
ADKFINEVVKQTQKIKLGDPLLEDTRMGPLINAPHLERVLGFVKLAKEQGATVLCGGEVY
VPEDPKLKHGYYMTPCILTNCRDDMTCVKEEIFGPVMSILTFGTEAEVLERANDTTFGLA
AGVFTRDIQRAHRVAAELQAGTCYINNYNVSPVELPFGGYKKSGFGRENGRVTIEYYSQL
KTVCVEMGDVESAF
>MmALDH16A1
MAATRVQPSTREIFTTLEYGPVPESHACALAWLDTHNRLLGHHVNGMWLKPEHRNPAPCQ
DPITGENLASCLQAEAEDIAAAVEAAKIAFKAWSQLPGAARGQHLTRLAKVVQKHQRLLW
TLESLVTGRAVREVRDGDVPLAQQLLQYHAVQAHAQGDALADWQPVGVIGLILPTPFSFL
DMMWRVCPALAMGCTVVALVPPAFPTPLLLAQLAGELGSFPGILNVVCGPASLGPVLASQ
PGVQKVAFCGAVEEGRVLRRTLAGRGAELGLALGTESLLLLTDSADVDSAVEGVVDAVWS
DRSLGGLRLLIQESVWDEAMRRLQARMAQIRSGRGLDGAVDMGARGAAARDLAQSFVDEA
QSQGGQVFQAGDVPSSSPFFSPALVSGLPPAAPCAQAEVPWPVVMASPFRTVKEALALAN
GTPRGGSASVWSERLGQALELGYGLQVGTVWINAHGLRDPAVPTGGCKESGSSWHGGPDG
LYEYLQPLGTPSQESFLCENINYDTFGLAASSILPSGPETGPSPAPPYGLFVGGRFQSPG
TQSSRPIQDSSGKVSSYVAEGGAKDIRGAVEAAHQAAPGWGAQSPRARAGLLWALAAALE
RRKPVLTSQLERHGAAPTVAKTEVELSVRRLQTWGTRVQDQGQTLQVTGLRGPVLRLREP
LGVLAVVCPDEWPLLAFVSLLAPALAHGNAVVLVPSGACPLLALEVCQDIAPLFPAGLVS
VVTGDRDHLTRCLALHQDVQALWYFGSAQGSQFVEWASAGNLKSVWVNRGFPRAWDVEVQ
GAGQELSLHAARTKALWLPMGD
>MmALDH18A1
MLRHMHRSGVQPFRQRLLPWVQSIAVPRSNRVQPSAIRHVRSWSNIPFITVPLSRAHGKP

FAHRSELKHAKRIVVKLGSAVVTRGDECGLALGRLASIVEQVSVLQNQGREMMLVTSGAV
AFGKQRLRHEILLSQSVRQALHSGQNHLKEMAIPVLEARACAAAGQSGLMALYEAMFTQY
SICAAQILVTNLDFHDEQKRRNLNGTLHELLRMNIVPIVNTNDAVVPPAEPNSDLQGVNV
ISVKDNDSLAARLAVEMKTDLLIVLSDVEGLFDSPPGSDDAKLIDIFYPGDQQSVTFGTK
SRVGLGGMEAKVKAALWALQGGTSVVIANGTHPKVSGHVITDIVEGKKVGTFFSEVKPAG
PTVEQQGEMARSGGRMLATLEPEQRAEIINHLADLLTDQREEILLANKKDLEEAEGRLAS
PLLKRLSLSTSKLNSLAIGLRQIAASSQESVGRVLRRTRIAKNLELEQVTVPIGVLLVIF
ESRPDCLPQVAALAIASGNGLLLKGGKEAAHSNRILHLLTQEALSIHGVKEAIQLVNTRE
EVEDLCRLDKIIDLIIPRGSSQLVRDIQKAAKGIPVMGHSEGICHMYVDSEASVDKVTRL
VRDSKCEYPAACNALETLLIHRDLLRTPLFDQIIDMLRVEQVKIHAGPKFASYLTFSPSE
VKSLRTEYGDLEVCIEVVDSVQEAIDHIHKYGSSHTDVIVTENEKTAEFFLQHVDSACVF
WNASTRFSDGYRFGLGAEVGISTSRIHARGPVGLEGLLTTKWLLRGQDHVVSDFSEHGSL
KYLHENLPVPQRNFS

>SmALDH2D1

MGSDAKDLVKHTKLFIDGRFVDAVSGKTFPTFNPSNSECIAQVAEGDAADVDLAVRAAREAFDHGPWPRL
AAAERGRILYKFADVIEEHLDELA
TLETLNNGMLIDLSKGIIAGSVASLRYNAGWADKLNGKTLRTDSTRMCYTLLEPIGVVGAIVPWNFPAHM
FLNKVGSALTCGNTIVVKVAEQTPLTGLLL
ASLSQEAGIPAGVLNVIPGYGPTAGAAISKHMSVDKVTFTGSTEVGRMIMESAARSNLKPVTLELGGKSP
FIICEDADLDSAVAVSQNAIFMHQGQVCVA
ASRVFVHESIHDEFIKRSVKLASERVIGDPFQSGVQNGPQINQEQLDKVLSYIESGKKEGASLLVGGKRI
GDKGFYIQPTIFGDVKQSMKIANEEIFGPV
LSVLKFKTLDEAVELANSTHYGLAAAVFSKNIDTVNLLTRSIKSGVVYVNSYLRAGPTVPFGGYKMSGIG
RENGYEGLLPYLQHKSILMPLENSPWN

>SmALDH2B2

MAPSTISDLPIGPAPVSVKYTKLFIDGQFVDAVSGRTFETLDPRNGEVISKVAEADKQDVDVAVKAARKA
FDHGPWPRLSGYARGRILLKFADL
LEHHFDELAALETLDNGKPLDLVKYVDLPMALRLLRSFAGFADKICGKTVKIDGPYHAYTLLEPIGVVGQ
IIPWNFPLIMFFLKISPALAAGNTIVLKTA
EQTPLSALFCASLLKEAGLPPGVLNILSGFGPTAGAAISSHNDVDKIAFTGSTDVGKLVMEAAAKSNLKA
VSLELGGKSPMIVLDDADVDVAVELAHLAL
FFNVGQCCVAGSRVFVQEGIYDEFLRKAADRAKRRVTGDSFQSGVDHGPVVDQQQFDRVLGYVEIGKREG
ARLVTGGCRIGSRGFYIEPTIFADVEDYMR
IAREEIFGPVMSVLKFRTIDEVIQRANDTAYGLAAGIVTKDLNSANRLTRSLRAGTVWINCYHVFDPALP
FGGYKMSGIGRENGKQVLYQYSQVKSVVTP
VESPWL

>SmALDH2B4

MEFGTILAYWWLNARIWILHQLAWCKTFPTIDPRSEEIIAQVAEGDEEDVNRAVKAARNAFEKGPWPRMT
AYERSKILFRYADLLEQHSDELVA
LDVLDNGKTIDQATFAEMPNVIRWFRYYAGWADKIHGMTLQADSPHHVHTLHEPIGVVGQIVPWNFPIIM
FSWKVAPALACGNTVVLKSAELTPLSAILA
GTLALEAGVPPGVLNIISGFGHTAGAAIASHMDIDKVAFTGSTEVGRSVMEAAARSNLKPVTLELGGKSP
FIVCGDADIDKALELSHLALFFNQGQTCCA
GSRTFVHESVYDEFVEKAKKKAENRVLGDPFQSGVEHGPQVDISQFNKVMKYIGYGKEQGATLLTGGERH
GDKGFYIQPTVFADVGDSMAISRDEIFGPV
QCISKFKTLEEVVERANNTQYGLAAGVFTQSLDTANFLSRALKVGTVWVNTYYAFDAAIPFGGYKMSGFG
REKGEYVLKNYLQVKAVVTPLKNPAWL

>SmALDH2B1
MDFLSRDKLKETIEHTQLFIDGQFVDSASGKKFAAFDPSTGETIADVAEGDERDVDLAVQAARKAFEEGP
WPRLAGAKRGKILAKLADLMEAKI
MDLSTLETLNNGMPLQATMFMTNAAIDVLRYYGGWADKIAGKTLKGDGDVHAYTLYEPIGVVGAIVPWNF
PVYLLVCKIAPALVCGNTMVVKPSEQAPLT
ALWIAKLALEAGVPAGVLNIVPGFGPTAGAAIARHMDIDKLTFTGSTNVGRLVMNDAASSNLKQVTLELG
GKSPFIICEDANLEVAAFFSHLAIFFHQGQ
VCLAGSRVFVHESVYDAFVEKAVAMAKRRVIGDPLKIEVEHGPQINQAQADKILSYIESAHAEGARLVTG
GKRIGDKGFYIEPTIFADVTQSMTIAKEEI
FGPVLSVLKFKTLDEAVKLANSTSYGLAAAIFAKDIDTVNFLSRSIKSGIVFVNSYFSAGPGIPFGGYKM
SGIGRENGYEGLLPYLQTKSVVMPLANSPW
L
>SmALDH2B3
MKSSILNSLSMGSLWTLPLVSETFPTVDPRTEEVLADVAKADVEDINRAVKAARKAFDHGPWPRMTAYER
SKILLKYADLLEKHNDELATLDSL
DSGKLYSQSQGVEIPHFVTRLFRYYAGWADKIHGKTLPADGPHQVLTFHEPIGVVGQIIPWNFPMVMFAW
KVAPALACGNTIVLKTAEQTPLSACLAAKL
AVEAGLPPGVLNVVSGFGETAGAAISSHMDIDKVAFTGSTETGKLVMQAAARSNLKPVTLELGGKSPFII
MPDADIDQAVELSHFALFFNQGQCCCAGSR
TFVHESIYDEYIEKAKARALKRVVGDPFKSGVEQGPQVDKAQFEKILSYIDVGRHEGANLVTGGARIGNK
GYYIQPTIFSDVKDDMAISRDEIFGPVQAV
TKFRTVQEAIERANNSPYGLAAGVFTKDIDTANTFSRALRVGSVWINCYDVFDAAIPFGGYKMSGQGREK
GEYVLHNYTQVKAVVTPLKNPAWL
>SmALDH2D2
MGSDAKDLVKHTKLFIDGRFVDAVSGKTFSTFNPSNSECIAQVAEGDAADVDLAVRAAREAFDHGPWPRL
AAAERGRILYKFADVIEEHLDELA
TLETLNNGMLIDLSKGIIAGSVASLRYNAGWADKLNGKTLRTDSTRMCYTLLEPIGVVGAIVPWNFPAYL
FLNKVGSALTCGNTIVVKVAEQTPLTGLLL
ASLSQEAGIPPGVLNVIPGYGPTAGAAISKHMRVDKVTFTGSTEVGRMIMESAARSNLKPVTLELGGKSP
FIICEDADLDSAVAVSQDAIFMHQGQVCVA
ASRVFVHESIHDEFIKRSVKLASERVIGDPFQSGVQNGPQINQEQLDRVLSYIESGKKEGASLLVGGKRI
GEKGFYIQPTIFGDVKQSMKIASEEIFGPV
LSVLKFKTLDEVVELANSTHYGLAAAVFSKNIDTVNLLTRSIKSGVVYVNSYLGDGPAVPFGGYKMSGIG
RENGYEGLLPYLQHKSILMPMENSPWN
>SmALDH3H1
MESAVIAAVCGEVRGDFKSGRTRSLDWRLAQLKSIVDLIKKHEEDITEAVAVDMGKPSYECFASEIFPVK
SACQLAIKNLKNWTAAVNVPPLLA
TLPASASMKPEPFGVVLIISAWNFPFLLSLDPMVGAIAAGNAVVLKPSEMAPATSALIARLLPLYLDKSA
IRVVEGGVPETTALLQQKWDKIFYTGSPRV
GRIVMAEAAKNLTPVTLELGGKSPVIVDSSSDLKVATRRIAVGKWGNNNGQACVSPDYVLVDSSCSTKFI
EAMKDTLKSFYGENPRESMDISRVVNINHF
NRLVGLLDDPNIASKIAHGGEKDETKLYIAPTLLEDVPLDSKVMSEEIFGPILPIISVRSIDEAIDIVNS
RPKPLALYLFTKKDKVKEKVIAETSAGGMV
VNDCCLHFLTTTLPFGGVGESGMGSYHGKFSFDAFSHHKAILTRPFWMDIMARYPPYSAHKKTFIRCLLE
ADFVGVILCLLGLKG
>SmALDH3H2
MDPLQTDRLVAELRDEFRSGRTRPMDWRAAQLRAMLRMIDEREGEIIEALDRDIGKPAYETYVAELSTIA
NSCTNALKHLRSWMAPEKVSTSMI

SFPSSGEIVPEPLGVALVISAWNFPFLLSLDPVIGAICAGNAVVLKPSELAPATAALLAKLVPLYLDKKA
IRVVEGGVPETTALLDQQWDKIFYTGSTRV
GKIVMAAAAKNLTPVVLELGGKSPVLVDSNVDVKVTARRIALGKWGNNNAQACISPDYILADESVVPKLI
TAIKECLLEFYGDDPSRSKDIARVVNGSHF
ERLTGLLDEDGVKDKIVFGGARDSNKLFIAPTVILDPPADSAVMTEEIFGPLLPVIPVDSMESAMSFVNT
RPKPLALYLFTRDKALEKKVVSETSAGGMV
VNDTVLHFVTETMPFGGVGHSGMGAYHGKFSFDAFSHRKAVLYRGFWADMASRYPPYTIAKQNFVRNFLQ
GNYLEAIKALLQNLRS
>SmALDH5F1
MATIASAGASQAVLDKINNAGLLRAKGLISGEWVSAEDGRTLPVYNPATGEFLTEVPLMGERETLSAIDS
AHNAFKSWSKKTCSERSKLLRRWY
ELILEKKEEIAQLMTLEQGKPLKEALGEVSYGAGFIELFAEEAKRTYGDIIPSPYPDRRLLVVKQPVGVV
GTITPWNFPLAMITRKVAPALAAGCTVVIK
PAELTPLTALAAAELSIQAGIPPGVINVVMGDAPQIGAALLESTKVRKISFTGSTQVGKKLMAGAASTVK
RLSLELGGNAPCIILDDADIEVAVKGALAS
KYRNSGQTCVCANRVLVQDGIYDKFAEAFIQAVSGLKVGNGLEEGVTQGPLINEAAVKKVEQHVEDALSK
GAMLLAGGHRHRLGAAFYEPTVLGDATEEM
LIMKEEVFGPVAPLIRFKTDEDAIRIANSTEAGLAAYLFSGSISRAWRVAEALEYGMVGWNEGVISTEVA
PFGGVKQSGLGREGSKYGIDEYLEMKYICI
GNMLP
>SmALDH6B1
MIRRVFCGVLARSSAAKGIPGDNRALVAGLNAVFSPRNFSSLPEKELNDSAIAFGQPKVKLLVGGEFIDS
KTSDWVDVVNPATQEIVSRLPLTT
PSEFDAAVDSAKAAFPKWRDTPVTTRQRVMLKLQELIRRDMDKLALNVTTEQGKTLGDARGDVFRGLEVV
EFACGAATLQMGEFVENVSTGIDTYSIRQP
LGICAGICPFNFPAMIPLWMFPVAVTCGNTFVLKPSEKDPGASLMLAELAAEAGLPPGVLNIVHGTNDVV
NRICDHPDIKAVSFVGSDKAGMHIYSRAAA
TGKRVQCNMGAKNHAVIMPDASPEATMNALTGAAFGAAGQRCMAISTAVFVGDSKPWEEGLKQRAMKLKI
GCGTEPGADLGPVISKQILAVQAKERICSL
VESGLKDGARVVLDGRNVEVPGFTSGNFVGPTILADVRPDMDCYKEEIFGPVLLCLKAETLEEAIEIVNS
NKYGNGTAIFTTSGPAARKFQHEIDVGQVG
INVPIPVPLPFFSFTGSRGSFAGDLNFYGKAGVHFFTHMKTVTTQWKDSPGGVTMAFPTSQKV
>SmALDH7B4
MDFGSRPELRFLEDLGLQATNQGCYGGGEWRAGGKSVSSLSPASNQPIATVIEGSLEDYERSLKACESAR
ESWMLTPAPKRGEIVRQIGDAFRK
NLENLGRLISLEMGKILVEGIGEVQEVIDMCDFAVGLSRQLSGLILPSERPNHMMMEVWNPLGIVGVITA
FNFPCAVLGWNACIALVCGNCVVWKGAPTT
PLVSIATTKIIAGVLERNGLPGAIFTCICGGAEIGEAIAKDSRIPLVSFTGSSKVGQIVQCHVNSRFGKC
LLELSGNNAIVVMDDADLSLAVRSVLFAAV
GTAGQRCTTCRRLFVHQAIYKDFIEKLVTAYGQLKVGDPLKHDSLVGPLHSSQSKQAFEQGLEAIASQGG
KFLTGGVGTTTSPGENYVTPSIVEISHDAD
VVKEELFGPLLYVFQFKTLDEAIAMNNSVPQGLSSSIFTKSHETIFKWIGPTGSDCGIVNVNIPTNGAEI
GGAFGGEKATGGGREAGSDSWKQYMHRATC
TINYGKTLPLAQGINFN
>SmALDH10A1
MAPAIPSRLLYIGGQWRAPDLGGQIAVINPATEDTIGYIPAATADDVDLAVKAAREAFTKDNGKYWARTT
GKYRAKFLRAIAAEVTARKSQLAE
LEAMDCGKPLDEAAWDMDDVAGCFDYYAGLAEGLDAKGHLSVELPMDTFKTNVLKEPIGVVGLITPWNYP

LLMATWKVAPALAAGCTAILKPSELASVTC
LELASIAAKVGLPAGVLNVVTGLGKDAGAPLSKHPGVDKVAFTGSTATGKSIMGAAAEIIKPVTLELGGK
SAIIVFDDVDIEKAVEWTMFGVFWTNGQIC
SATSRLLLQENIASVFLERLAEWTKTIKISNPLEPGCRLGPVVSDGQYKKVMKYISTAQEEGATLLCGGK
RPEHLSKGYFVEPTVFANVRPSSQIWKEEV
FGPVLAVRTFRTEEEAIKLANDSEYGLAGAVISTDEDRCQRVAELLQAGIIWINCAQPTFTQAPWGGTKR
SGFGRELGEWGLENYLSVKQVTKYISEEQW
GWYPRPSKM
>SmALDH11A1
MAGTGTFKEIVEGDVYKFYADGQWRLSSSGNISYVYNPTVAGKQAYKITACSKDEVNQAFQSAKAAQKIW
AKTPLYKRAEMLHKVAEIMRKNKGP
IAECLVKEVAKPAKDSVTEVVRSADLLSYTAEEGIRILSEGKFLVSDSFPGNPRNKLCLASNAPLGVILA
IPPFNYPVNLTCSKIGPALIAGNAVVVKPP
TQGSVSTLHMMHCFHLAGFPKGLLSCVPIKASELGDYVTTHPLVNCISFTGGCDTGMSISKKAGMVPLQM
ELGGKDAMIVLEDGDIELAATNIIKGGFSF
SGQRCTAVKVVLVMESVADKLVSSVNAKVAQLKVGPPENDCDIVPVVTEASANFIEGLVTDAKAKNAKLC
QAKFSRQEWKRQGNLIWPLLIDNVTTDMRI
AWEEPFGPVIPVMRIKTPEEGVKHCNSSKYGLQGCVFTSDINKAMLLSDAMESGTIQINSAPARGPDHFP
FQGIRESGVGSQGVTNSINLMTKIKTTVLN
LPTASYTMGTNKLPFSSLLSFLLSLVLGSGRRRVFADREKRTSVW
>SmALDH11A2
MSIGGDCRCCVDEEIDAFGEIVGGNGVFKYYADGAWKISSSEESLPVINPTSREPFVAITACTRREVNKA
FHSAKEAQKSWAKTPLFKRAEMLHK
VAGVMKEKKNMEAIAECLIKEVAKPDKDAYTEVVRSADLLSYTAEEGIRILSQGKFLVSDSFPGNERNKV
CISSKVPLGIILAIPPFNYPVNLTVSKIGP
ALIAGNAVVIKPPTQGAVSTLHMIHCFHMAGFPKGLISCVTIKASELGDYMTTHPLVNCISFTGGCETGI
SISQKAGMIPLQMELGGKDAMIVLEDADLD
LAATNIIKGGFSYSGQRCTAVKIVLAMEGIADALVSKVNAQIAKLKIGPPERIDTDIVDVVSEASADFIE
TLVADAIDSGAKLCQGWKRRGNKIWPLLID
NVTTEMRIAWEEPFGPVIPVMRIKSVEEGIQHCNSSKYGLQGCVFTKDINKAILVSDAMESGTIQINAAP
ARGPDHFPFQGIRDSGIGSQGVTNSINFMT
KIKTTVINLPSASYSLGKILFLELKGLAMERAFPCFQLWVIMSTRNRKGSWMPEILKL
>SmALDH11A3
MAGTGVFKACLDGDVYKYYADGDWKMSSSGKTVSVTNPTTGKPIYKVTACTQDEINKVFQSAKAAQKLWA
KTPLYKRAEMLHKVSVIMKEHKSP
LADCLVKEVAKPAKDAVTEVVRSGDLIDYTAEEGIRIMSEGKFLVSDPFPGNERNKLCLSSKVPLGVILA
IPPFNYPINLSVSKIGPALITGNSVVIKPP
TQGAVSTIHMMHCFHLAGFPKGLISCVTIKVSELGDFLTTHPAVNCISFTGGCDTGIAISKKANMIPLQM
ELGGKDTMLVLDDADVESAAVNIMKGGFAF
SGQRCTAVKVVLAMESIADALVSKVNAKVAKLSVGPPENDCDIVPVVSESSANFIEGLVMDAKQKGAKLC
QEWKREGNLIWPLLIDHVKPDMRIAWEEPF
GPVIPVLRIKTAEEGINHCNSSRYGLQGCVFTKDVNKAMLVSDAMETGTVQINAAPARGPDHFPFQGIRD
SGIGSQGVTNSINMMTKIKTTVINLPQPSY
TMA
>SmALDH11A4
LAGQGPFKSVLDGDVFCYYADGEWKTSSSGKSLAVLNPSNGKTQYKVTACTRDEVNKAFQSAKKAQKEWA
KVPLCKRAEYLYKAAAILKAHKTPI
AECLVREVAKPAKDSVTEVVRSADLIVYTAEEGIRILSQGNFLVSDSFPGNDRNKLCFTSKVPLGVVLCI

PPFNYPVNLSVSKLGPALIAGNAVVLKPPT
QGAVSAIHMMHCFHLAGFPKGLLSCVTIKVSELGDYLTTHPMVNCISFTGGCETGISISRKANMVPLQME
LGGKDAFIVLDDADLEAAATNVIKGGFAFS
GQRCTAVKVVLAQESIADTLVSKINAKVGKLTVGPPEQDCDIVPVVSDASANFIEGLVSDAKQKGAKLCQ
DWKRQGNLIWPILVDKVKPEMRIAWEEPFG
PIIPVIRIKTQEEGIKHCNASRYGLQGCVFTQSLDRAMQVSDAMETGTVQINAAPARGPDHFPFQGVRDS
GIGSQGITNSINMMVKIKTTVMNLPSNSYT
MA
>SmALDH11A5
GPFQDIVEEDGSFKYYADGEWQTSSSRRSVTISNPATREAQYKVQACTQEEVNRAIDSAKAAQRVWAKTP
LWKRAEKLHKAASLLKELKNPIAEC
LIKEIAKPAKDAMSEVVRSGDLISYTAEEGIRVLAEGKFLVSDSFTGNDRNKLCLASKIPLGVILAIPPF
NYPVNLAVSKIAPALIAGNAVVVKPPTQGA
VAGLHAIHCFHLAGFPKGLISCITGKGSEIGDFLTMHSGVNCISFTGGDTGIAISKKAGMIPLQMELGGK
DACIVLEDADIDLAATNIVKGGFSYRQVLD
GQRCTAVKLVLVMDSVADELVAKVNARISKLTVGSPEDNSDITAVVSEASANFIEDLVEDAREKGASFCQ
DYKRKGNLIWPLLVDRVTPDMRLAWEEPFG
PVIPVIRIKSVEEAIHHCNSSNLGLQGCVFTRDINKAMIMSDAMETGTVQINSAPARGPDHFPFQGLRDS
GIGSQGVTNTINMMTKTKTTVINLPGPSYS
MG
>SmALDH11A6
MAAGTGPFEEIIDGGVYKYYADGEWRTSSSGKSVTIYNPSTREAQYKVQACTQEEVNKAIDSAKAAQKLW
AKVPLWKRAEALHKAAALLKELKD
PIAECLVKEIAKPAKDAVSEVVRSGDLISYTAEEGVRILAEGKFLVSDSFPGNDRTKLCLTSKIPLGVVL
AIPPFNYPVNLAVSKIAPALIAGNAIVLKP
PTQGAVAGLHTVQCFHRAGFPKGLISCITGKGSEIGDFLTMHPGVHCISFTGGDTGIAISKKAGMIPLQM
ELGGKDACIVLDDADIDLAAANVVKGGFSY
SGQRCTAVKVVLVMESVADDLVSKVNAKIAKLKVGAPEDDCDITAVVTESSANFIEGLVKDAKEKGATFC
QEYKREGNLIWPLLLDHVRPDMRIAWEEPF
GPIVPVIRISSVEEGIHHCNASNFALQGCVFTRDVNKAMMISDAMETGTVQINSAPARGPDHFPFQGLRD
SGIGSQGVTNSIAMMTKIKTTVINLPAPSY
SMG
>SmALDH12A1
MDSSSALAFATVYPDDLSTAHPYQVENLVRGRWGKSLKSSKLPDPLNGGEFITVPEATGHELPEYIESLQ
SCPKSGLHNPLKNPERYLLYGDISA
RAASSLKHREVGHFFARLIQRVSPKSYQQAQAEVTVTQKFLENFSGDQVRFLAKSFAAPGNHLGQQSIGY
RWPYGPVAIITPFNFPLEIPVLQLMGALYM
GNKALVKVDSKVSVVMEQMLRLLHACGLPPNDTDFINCDGPVMNKLLVEAKPSMTLFTGSSRVAEKLALD
LKGRIKLEDAGFDWKILGPDVNEIDYVAWV
CDQDAYACSGQKCSAQSILFMHENWSSRNFVEKLKQLAAKRKLDDLTIGPVLTVTTKTMLDHMEKLLSIP
GSSVAFGGKPLENHSIPDVYGAIEPTAIFV
PLKEILKDEHFDLVTKEIFGPFQVITEFKQCELPLVLRACERMHAHLTAAVVSNDVEFLQEVLGETVNGT
TYAGIRARTTGAPQNHWFGPAGDPLGAGIG
TPEAIKLVWSCHREIIQDFGPVPKNWTSTTS
>SmALDH18B1
MRQQIDPSREFTRDVRRVIVKVGTSVVTRPDGKLAVGRLGALCEQVKDIMDDHIEVIVVTSGAVAVGRQK
LRQQRMMNSRQVRRVIHHFCFLLV
CLDLQKPQAELSGKACAAIGQAGLMALYDSIFSQLDVASSQLLVTDREFIDPEFRKQLCETVDTLLQLRV

VPIFNENDAMSTRRAPYKDSSGIFWDNDSL
AALLALELKVDLLILLSDVEGLYTGPPSDPSSKLISTYIPAQHETITFGEKSRYGRGGMTTKVTAAREVA
SAGIPVVITSGYTPDGFHRVLRGDSIGTLF
HKDAHIWTLTINNDARLMAVAARDSSRRLQSLTSGERQNILYDVADALERNEETIRSENQADVRLAEELG
IAKPLLSRLTLKPGKIADLAKAVRALADMR
EPIGSILQRTEVAENLTLDKTSCPLGVILVIFESRPDALVQIASLAIKSGNGLLLKGGKEAARSNAILHK
VITEALPKSVGKELIGLVTSRDEIPDLLKL
DDVIDLVIPRGSNKLVSQIKESTKIPVLGHADGICHVYVDKAARLDVATKIAIDSKVDYPAACNAMETLL
VHEDLVDTGGLETLASALKSAGVTLNGGER
ASKLLNIPKMTKFHHEYSGLACTVEVVKDVHAAINHIHEHGSAHTDCIVTEDKEVAELFLQQLDSAAVFH
NASTRFSDGTRFGLGAEVGISTSRIHARGP
VGVEGLLTTRWLLRGNGQVVQGDRGVVYTHKDVEVDSMEGWTSMGNGLPASVNGGSLKKSASPFLGSVLA
PSVDAAT
>SmALDH21A1
MKFPMYIASCARDQSANYLDVIDKHTGQAAAKVPLASPEDIEEAIQECVKAAPAMAALPSYERKAVLRKI
VFELEKRSEEIAQLITTESGKPIK
DARGEVQRSIDTFEVAAEEATRIYGEVLPLDISARNKGIEGIVKKFPIGPVSMVSPWNFPLNLVAHKVAP
AIAVGCPFVLKPSSRTPLSALFLGEILASC
DSLPKGAFSILPTTRNEADAFTTDDRFKLLTFTGSGMAGWNMKARAGKKKVVMELGGNAPCIVEDLVPDL
EGTIARLVHGGFYQSGQSCIHMQRLYVRGG
LYKEVKDALIAAVKKLKGGDPRQDDTSIGPMISESSAATVEKSVNEAVKAGAKLLVGGKRRGAFMEPTVL
EDAPFDTDARKEEIFGPVILLYSYNDFKEA
VKEANNTHYGLQAGVFTRDLNKAFYAFEHIEAGGVCLNDSPSMRVDSQPYGGIKDSGIQREGVKYAMDDM
LETKVLVMRNVGNASYF
>SmALDH22A1
MDVLWAVAILAILFYLCRLLMLLIPPVVPEIVVDTSDVMLGKGGDDDSYIYVARRRSGDEDTVHCYDPAT
MKYLGQLPALNFDEVSDHVARARE
AQKKWARSSFEKRRQLLRILLKYTIDHQELICEVSARDSGKTLVDAALGEILTTCEKITWLVGQGEQWLQ
PEYRLVKLFISAGRMMLHKTARVEYSPIGV
IGAIVPWNYPFHNILNPMVSAVFAGNAIVIKVSEHASWSAFFYSRIIKAALRAAGAPADLVHVITGYGET
GKALVSSLVDKLIFVGSTAVGKMVMEQAAKT
LTPVVLELGGKDPFIVCEDADVAQIAARAALQSSGQNCAGAERFYIHAQIYQQFVDEVVRIVRTVRMGPP
LEGLFDMGAVCIQEHTDRLQALVNDAVTKG
AEIAVRGDLVLPDFGNSVVGQFYPPTVLLNVNHSMRLMQEEIFGPIIPIMKFHSDDEAITLANDSNFGLG
CSVFSANKERAVAIASKIYCGMAAINDFAV
TYMCQSLPFGGVKNSGFGKFAGVEGLRGCCLVKSIAEDRFSFFKTYPVAENAFQFEEALVRMFYGLTVVE
KFQGLVNLVKIFTEQKDVKKTL
>SmALDH23B1
MASFCKIVKRFYRANKPIKPNTSWPGYRNVLDITNPATLKKVGELQEDTIPDIERKLEYLHTGLKRWKLT
HVDQRKAALEKFADALVTRKTTLA
KILTSETGKPISQARSEIAATVDRIHYFVENCEKVIGTQTVLESSRLKEKVQYEPLGVIANISAWNYPYF
LSANVFAPALLTGNCVLYKPSENASLTGQE
ITDMLHNAGIPEDVFVISSGGADTGSRIAGNKDIGGLFFTGSYDTGLEIAKKASPNLVKLQLELGGKDAA
YIRHDVPNILATATTVADGAFYNSGQSCCS
IRRIYVNKRVYVPFMEALKKISHSYRIGDPTLEDTYIGPLCTKKQVEKIGKLLAEAINRGAQVDVGGDTT
NSTAHKVGYFVPPTILTDVDHQMSIMRKET
FGPVVGVMCVEDDDEATTLMSDTEYGLTASVFSKHLEDAEAILNELKVGTGYWNCCDRVSPRVPWSGRKR
SGVGSTLGIDGLRAFVQPKGFFCHEPMAAI

>SmALDH23B2
MASFCKIVKRFYRANKPIKPNTSWPGYRNVLDITNPATLKKVGELQEDTIPDIERKLEYLHTGLKRWKLT
HVDQRKAALEKFADALVTRKTTLA
KILTSETGKPISQARSEIAATVDRIHYFVENCEKVIGTQTVLESSRLKEKVQYEPLGVIANISAWNYPYF
LSANVFAPALLTGNCVLYKPSENASLTGQE
ITDMLHNAGIPEDVFVISSGGADTGSRIAGNKDIGGLFFTGSYDTGLEIAKKASPNLLELGGKDAAYIRH
DVPNILATATTVADGAFYNSGQSCCSIRRI
YVNKRVYVPFMEALKKISHSYRIGDPTLEDTYIGPLCTKKQVEKIGKLVDVGGDTTNSTAHKVGYFVPPT
ILTDVDHQMSIMRKETFGPVVGVMCVEDDD
EATTLMSDTEYGLTASVFSKHLEDAEAILNELKVGTGYWNCCDRVSPRVPWSGRKRSGVGSTLGIDGLRA
FVQPKGFFCHEPMAAI
>OtALDH3
MRASFRSGHTLPMRKRKEQLGQLLKMLKEREDEILDALREDLSREHVEAFYYDFALPRAEIRAMLRNIRS
WTGRSLVKAFNVITWPSKQWMERQPLGCALVCSSWNFPFLLSLVPVAGAIAAGNAVVLKPSNDSKASTAL
LVKLVREYCDPRVVQCVGSEVPGNGVDVMQTVLKEKFDVIFFTGSSKVGKIVARAAAENLTPCILELGGK
NPVVVTDCADVDLAAKQCVWGRVINCGQQCISPEYVLCHESRCDEFERMCSKWAAKFVPDVTLNGAMARI
GGPDPESRMKAIAKLIDDAKAGVAGDTVVYGGTYDVKRRLVEPTVIKCGEKSPFMEAELFAPILCVHSYK
TLGQAVDTIQAQMKPLTMYVFSRSAKKTRFLLDNTHAGGVTVNGTLTHCAHDRLPFGGVGDSGYGRYHGR
YSVECFQREKPVLQKTRWGRCLGLGLLSDPSFLYSPQAEWKTKCVRAVASLM
>OtALDH11
MTTSSSSDGFYAELTNAEGGALRYAVNGAWRASSSDATVESVNPSRANARANAFQACTRAEVDEAFAGAR
AAQGPWARTPLHERASLLHRAATLMRENAGGMVSALMIEVAKGAKESATEVERXXXXXXXGNDRNKICMA
SNVPVGVVLCIPPFNYPVNLCVSKVAPALIAGNAVVVKPPTQGCTATLHMIHCFIKAGFPPGLIQAVTGR
GGEIGDYLTTHPLVNAISFTGGETGIRVAQKAGMVALQTELGGKDACIVLPDADLELAAKSIVKGGFSYS
GQRCTAVKIVAVFEEVADELIGKVNERIAKLSVGLPEDDATITAVVSKSSADFIQSLVVDAESKGATLCQ
EWKREDNLIWPLLIDNVTHDMKICWEEPFGPVLPVVRVKNENEALELVNKSRFGLQGCVFTRDIDRAIRL
SDAMQTGTVQINGPPARGPDHFPFQGVKDSGIGSQGITNSIKVMTKVKSTVINLAKPSYTIA
>OtALDH5
MSVRARASSSNASAASEVARALGLRRDDLLLDVVREREASTSGTMSVEVTNPATGTTLARCRATSANDVE
HILRRSKESQERWANEYTAHARAKIVRRWFELVEANAEDLARVCTAESGKPLAESRAEVAYAASFLEWFA
DEGRRVYGDVVPSSSTGSRIMAIKQPVGVTAAITPWNFPLAMITRKAGAALAAGCSMVVKPSEETPLSAF
ALGALAKEAGCPDGVLQFVVGDPVVIGELLCASPIVRKITFTGSTRVGKLLMKQSSDTVKRVSMELGGNA
PFIVCADADVDAAVRGAMASKFRNSGQTCVCAQRFIVHESVEDDFVRKLTAAANALKLGNGLEDESVTQG
PLINAAQVERVDAHVRDALSKGAVCHSGGKRADGTFYEPTVLSKCTDDMLVMQEETFGPVAAITTFVSDD
DALRMANSTNAGLASYVFTSDLKRSYTFSEKLEFGIVGVNTGVISTAQAPFGGVKESGVGREGGKYGMEE
YVETKYVCVGGLD
>OtALDH22
MSLVRVLTGHVRVALDATNAPEIAREIARVAVTTIEQRHAWTAVIAIAIVLRVLLVERTPKIVVALTEEE
ASCEGDALTFDPSGAWPTKTVPCYDPGTMRRLGPDVEAMSAEEVRARIRRASEAQKEWAKSSFATRRKLL
RVIQRFILEEQDSICRVSARDSGKPLVDAAFGEVLVTLEKIRWLCNEGEQWLKPESRSSGAMMFYKKARV
EYHPVGVMGAIVPWNYPFHNVFNPLVANLFAGNALVVKVSEYATWSSQYYGRVIDAALDAVGAPRDLVQI
ITGYGEAGNALVTGGCQKVVFVGSTGIGRKVMEAAAKTLTPVVLELGGKDPFIVCADADLKQCVPMALRG
AFQSCGQNCAGAERFYVHEKIHDKFLGKVVDSAKKLRQGPSVDKRAIMCMPKQAQYVQSLIDDAVARGAI
VHLGAQGGQFYPPTVISGITHDMRIAREEVFGPVLAIVKTKSDEESIALANDCDFGLGSNVFTRSTKRAE
FLGKQLEAGMTSINDFCSTYMAQSLPFGGVKESGFDRFAGIEGLRGCCVPKSVVVDRFPLLMKTNIPPPL
CYPVADNAFAFCKALARMFFGLSLAQRFGGLLALAKCFLLPSKTYTKYD
>OtALDH12
MSRAAGAARGANLIAGAWREITDESRATKIVDPMNKSETDAFIVLPSTDTKQEIDEVARSLASCPKSGLH

NAFKAPERYVMWGDVSMRLAQEFRKPEVEEYFARLIQRVAPKSHAQALAEVVVTRKFLENFAGDNVRFMA
RGFSVSGDHLGQQSHGLRWPYGPVAVITPFNFPLEIPVLQLMGALFMGNKALVKSDSKVSVVLEQFIRLM
IECGAPATDLDFIHSDGVTMNSILSAAKPKMTLFTGSQKVAHHLARELEGNVKLEDAGFDWKILGPDVGD
VDYVAHVCDQDAYACSGQKCSAQSILFMHKNWVDVGIESKLAALAGERNLEDLTVGPVLTLTTKTMLDHV
DRLAALPGARVAFGGKELKDGNHSIPSQYGAIEPTAVFVPLKTIMASEENFKLVTTEFFGPMQVLTSYDD
EELPLVLDACERMDAHLTAAVVSSDEMFSQRVLGSTVNGTTYAGRRARTTGAPQNHWFGPAGTPMAGGIG
TIEAIRLVWSCHREIIFDRGPVESDWKTPPRA
>OtALDH10
MDVRGGALGRAGALIDNAWTTTRRSLPVVNPHDGAVVGAIARGSVADVDDAVRSARKGFVTWSTRNGRER
AKTLRAVADGLRRRRETLARLETTDCGKPLDESAWDVDDAIGCFEYYADRCERVFGERAYAEEVVELPDE
DFAGRVRREPLGVIGLITPWNYPLLMATWKVAPALASGCAVVLKPSEEASLTCQVLGDVCVEAGLPPGAL
CVVTGRGDEAGAALCAHRGVDKISFTGSFRTGQTIMRACAQDVKPVSLELGGKSALVIFDDCDLEKAVEW
AMFGCFWTNGQICSATSRVLVHENIRERFLARLKEASEAIPVGDPLAEGCRLGPLASAAQYKKVTSMVNR
IKRTKIHLLTGGNRPRARGCEKGFYIEPTVFVDPPLDSEAWREEIFGPVMCVRSFRTEEEVIAITNDSEY
ALAAAVITDDVARRERMASAFDVGIVWIQCSQPAFTQLPWGGRRRSGFGRDLGVNGMDKYMHQKQIVEYT
SGAQFEWYPMFKKSKL
>MdALDH2B4
MAARRLSSLLSRSLSAASPSSSSGSAASLLRSGGRRVSRFSTAAATEELIIPPVQISHTQHLINGKFVDA
ASGKTFPAYDPRTGEVI
AHVAEGDAEDINRAVAAARKAFDEGPWPKMSAYERSRILLRFADLVEKNSEELAALETWNNGKTYEQALT
AELPMLARLFHYYAGWADKIHGLTVPADGP
YHVQTLHEPIGVAGQIIPWNFPLLMFAWKVGPALACGNTIVLKSAEQTPLTALYVAKLFQEAGLPPGVLN
VVSGDGPTAGAALASHMEVDKVAFTGSTDT
GKIILELAARSNLKPVTLELGGKSPFIICEDADINHAVELAHFALFFNQGQCCCAGSRTFVHERVYDEFI
EKAKARAVKRVVGDPFKKGVEQGPQIDNEQ
FEKVLRYIRSGIDSNATLECGGERLGSKGYFIQPTVFSNVEDDMLIAQDEIFGPVQSILKFKELDEVVRR
ANATRYGLAAGVFTKNINTANYLTRALRAG
TVWVNCFDVFDAAIPFGGYKMSGIGREKGIYSLHNYLQVKAVVTPLKNPAWL
>MdALDH2B12
MAARRLSSLLSRSLSAASSSSSSSSGSAASLLRSRGGSVSRFSSTAVATEELIIPPVQISHTQHLINGKFV
DAASGKKFPTYDPRTGE
VIAHVAEGDAEDINRAVAAARKAFDEGPWPKMGAYERSRILLRFADLVEKNSEELAALETWNNGKPYEQA
LTAEVPMLARLFHYYAGWADKIHGLTVPAD
GPYHVQTLHEPIGVAGQIIPWNFPLLMFAWKVGPALACGNTIVLKSAEQTPLTALYVAKLFQEAGLPPGV
LNVVSGDGPTAGAALASHMDVDKVAFTGST
DTGKIILELAARSNLKPVTLELGGKSPFIICEDADIDHAVELAHFALFFNQGQCCCAGSRTFVHERVYDE
FIEKAKARAVKRVVGDPFKKGVEQGPQASN
HCDNSQIDNEQFEKVLRYIRAGIDSXATLECGGGRLGSKGYFIQPTVFSNVKDDMLIAKDEIFGPVQSIL
KFKELDEVVRRANATRYGLAAGVFTKNIDT
ANYLTRALRAGTVWVNCFDVFDAAIPFGGYKMSGIGREKGIYSLHNYLQVKAVVTPLKNPAWL
>MdALDH2B13
MAARRLSSLLSRSLSAASSSSSSSSSGSAASLLRSRGGSVSRFSSTAVATEELIIPPVQISHTQHLINGKF
VDAASGKKFPTYDPRTG
EVIAHVAEGDAEDINRAVAAARKAFDEGPWPKMGAYERSRILLRFADLVEKNSEELAALETWNNGKPYEQ
ALTAEVPMLARLFHYYAGWADKIHGLTVPA
DGPYHVQTLHEPIGVAGQIIPWNFPLLMFAWKVGPALACGNTIVLKSAEQTPLTALYVAKLFQEAGLPPG
VLNVVSGDGPTAGAALASHMDVDKVAFTGS
TDTGKIILELAARSNLKPVTLELGGKSPFIICEDADIDHAVELAHFALFFNQGQCCCAGSRTFVHERVYD

EFIEKAKARAVKRVVGDPFKKGVEQGPQAS
NHCDNSQIDNEQFEKVLRYIRAGIDSXATLECGGGRLGSKGYFIQPTVFSNVKDDMLIAKDEIFGPVQSI
LKFKELDEVVRRANATRYGLAAGVFTKNID
TANYLTRALRAGTVWVNCFDVFDAAIPFGGYKMSGIGREKGIYSLHNYLQVKAVVTPLKNPAWL
>MdALDH2B14
MVTRRVGSLLNRSFTSASMFSKXRSSSVVRGIGKYSTDASIESPISPSVKVNYTQLLINGQFVDSASGKT
FPTLDPRTGEVIAHVAE
GDAEDVNRAVSAARKAFDEGPWPKMTAYERSRVLFRFADLIEKHNDEIAALETWDNGKPFEQAAKIEVPM
VARFFRYYAGFADKIHGLTVPADGEYHVQT
LHEPIGVAGQIIPWNFPLLMFAWKVAPALACGNTIVLKSAEQTPLSALYAATLLQEAGLPPGVLNVVSGF
GPTAGASLASHMDVDKVAFTGSTDTGKKIL
ELAAKSNLKTVTLELGGKSPFIVCEDADVDKAVEMAHFALFFNQVSKHCLQKKV
>MdALDH2B15
MATRRVGSLLNRSFTSASMFSKXRSSSVVRGIGKYSTDASIESPISPSVKVNYTQLLINGQFVDSASGKT
FPTLDPRTGEVIAHVAE
GDAEDVNRAVSAARKAFDEGPWPKMTAYERSRVLFRFADMIEKHNDEIAALETWDNGKPFEQAAKIEVPM
VVRFFRYYAGMDLFNTTTPHALWGLNKIYV
LILDTPQDLRTRFMVSXFQPMESIMCKPCMNLSVLQVAPALACGNTIVLKXAEQTPLSALYAATLLQEAG
LPPGVLNVVSGFGPTAGASLASHMDIDKFA
FTGSTDTGKKILELAAKSNLKTVTLELGGKSPFIVCEDADVDKAVEMAHFALFFNQGQCCCXGSRTFVHE
KVYDEFLEKAKXRAERRLVGDPFKGGIEQG
PQILKYIDYGVKAGAKLETGGERVGTKGFYIKPTVFSDVXDDMXIAXEEIFGPVQTILKYKDLNEVIRRA
NNSRYGLAAGVFTQNIDTANTLXRALRVGS
VFINCXXXFDASIPFGGYKMSGIGREKGIYGLSNYLQVKAVVTPLKNPAWL
>MdALDH2C4
MMKLADLIDQHLEELAILDTVDAGKLFSSCKTXDIPXVXEXVRYYAGAADKIHGEVLKMSRDLQAYTLLE
PIGVVGLIVPWNFPSTL
LFAKVSPALAAGCTMVIKPAEQTPLSALYXAHLAKLAGVPDGVFNVITGFGKTAGAAXSHHMDIDKVSFT
GSTEVGREVMQAAAKSNLKQVSLELGGKSP
LVIFDDADINMAADLALLGILFNKGEICVASSRVYVQEGIYDEFVKKLQEKXKDWVVGDPFDPXVHQGPQ
VTDSTASNGVDKKQFDKILTYIEHGKNGGA
TLLTGGKPVGNKGYYIEPTXFTDVKDDMLIAQDEIFGPVMALMKFKTIEEAIQRANNTRYGLAAGIITND
LNVANTVSRSIRAGIIWINCYFEFDRDCPY
GGYKMSGFGRDSGMQGLYEYLHTKSVVTPIYSSPWL
>MdALDH2C6
MGSDFNGSSAXFVKIPIIKFTQLFINGDFVDSVSGKTFETIDPRTGEVIARVAEGDTEDVDLAVKAARAA
FDHGPWPRLPGAERGRI
MMKLADLIDQHLEELAILDTVDAGKLFSSCKTVDIPQVAEMVRYYAGAADKIHGEVLKMSRDLQAYTLLE
PIGVVGLIVPWNFPSTLLFAKVSPALAAGC
TMVIKPAEQTPLSALYYAHLAKLAGVPDGVFNVITGFGKTAGAAISHHMDIDKVSFTGSTEVGREVMQAA
AKSNLKQVSLELGGKSPLVIFDDADINMAA
DLALLGILFNKGEICVASSRVYVQEGIYDEFVKKLQEKAKDWVVGDPFDPNVHQGPQVDKKQFDKILTYI
EHGKNGGATLLTGGKPVGNKGYYIEPTVFT
DVKDDMLIAQDEIFGPVMALMKFKTIEEAIQRANNTRYGLAAGIITNDLNVANTVSRSIRAGIIWINCYF
EFDRDCPYGGYKMSGFGRDSGMQGLYEYLH
TKSVVTPIYSSPWL
>MdALDH2C7
MGSDFNGSSASFVKIPAIKFTQLFINGGFVDSVSGKTFETIDPRTGEVITGVAEGDKEDVDLAVNAARAA

FDHGTWPRLPGAERGRI
MMKLADLIDQHVEELATLDTIDAGKLFSFGKTRDIPQVAEMVRYYAGAADKIHGEVLKMSRDLQAYTLLE
PIGVVGLIVPWNFPSTLLFAKVSPALAAGC
TMVIKPAEQTPLSALYYAHLAKLAGVPDGVFNVITGFGKTAGAAISHHMDIDKVSFTGSTEVGREVMQAA
AKSNLKQVSLELGGKSPLVIFDDADINMAA
DLALLGILFNKGEICVASSRVYVQEGIYDEFVKKLQEKXKDWVVGDPFDPXVHQGPQVTDSTASNGVDKK
QFDKILTYIEHGKNGGATLLTGGKPVGNKG
YYIEPTXFTDVKDDMLIAQDEIFGPVMALMKFKTIEEAIQRANNTRYGLAAGIITNDLNVANTVSRSIRA
GIIWINCYFEFDRDCPYGGYKMSGFGRDSG
MQGLYEYLHTKSVVTPIYSSPWL
>MdALDH2C8
MGSDFNGSSXSFVKIPAIKFTQLFINGGFVDSVSGKTFETIDPRTGEVITRVAEGDKEDVDLAVKAARAA
FDHGTWPRLPGAGRGRI
MMKFADLIDQHVEELATLDTIDAGKLFSFGKTRDIPQVAEMVRYYAGAADKIHGEVLKMSRDLQAYTLLE
PIGVVGLIVPWNFPSTLLFAKVSPALAAGC
TMVIKPAEQTPLSALYYAHLAKLAGVPDGVFNVITGFGKTAGAAISHHMDIDKVSFTGSTEVGREVMQAA
AKSNLKQVSLELGGKSPLVXFDDADINMAA
DLALLGILXNKGEICVASSRVYVQEGIYDEFVKKLQEKAKDWVVGDPFDPNVRQGPQVDKKQFDKILTYI
EHGKNEGATLLTGGKPLGNKGYYIEPTIFT
DVKDDMLIXQDEIFGPVMALMKFKTIEEXIQRANNTRYGLAAGIITKDLNVANTVSRSIRAGIIWINCYF
AFDRDCPYGGYKMSGFGRDFGMQALYKYLH
TKSVVTPIYNSPWL
>MdALDH2C9
MGSDFNGTSASDSFVKTPTIKFTQLFINGEFLDSVSGFSPFSPSSQMLRAGKTFETIDPRTGDVITRVAE
GDKEDVDLAVKAARAAF
DHGPWPRLPGAERGRIMMKFADLIDKHAEELAILDTVDAGKLFSVGKTMDIPQVAEMLRYYAGAADKIHG
EVLKMSRELHGYTLLEPIGVVGLIVPWNFP
STXLFGKVSPALAAGCTMVIKPAEQTPLSALYYAHLAKLAGVPDGVLNVITGFGKTAGAAISYHMDIDKV
SFTGSTEVGREVMQAAAKSNLKPVSLELGG
KSPLVIFDDADINMAADLALLGILYNKGEICVASSRVYVQEGIYDEFVKKLQEKAKDWVVGDPFDPNVRQ
GPQVDKKQFERILSYIEHGKKEGATLLTGG
KPVGNKGYYIEPTIFTDVKDDMLIAQDEIFGPVMALMKFKTIEEAIQRANNTKYGLAAGIITKDLNVANT
VSRSIRAGIIWINCYFAFDRDCPYGGYKMS
GFGRDFGMQGLYHYLHTKSVVTPLFNSPWL
>MdALDH2C10
MLTWLSRRRVLPSTMALGLACLALYERGRIMMKFADLIDKHAEELAILDTVDAGKLFSVGKTMDIPQVAE
MLRYYAGAADKIHGEVL
KMSRELHGYTLLEPIGVVGLIVPWNFPSTLLFGKVSPALAAGCTMVIKPAEQTPLSALYYAHLAKLAGVP
DGVLNVITGFGKTAGAAISYHMDIDKVSFT
GSTEVGREVMQAAAKSNLKPVSLELGGKSPLVIFDDADINMAADLALLGILYNKGEICVASSRVYVQEGI
YDEFVKKLQEKAKDWVVGDPFDPNVRQGPQ
VDKKQFERILSYIEHGKKEGATLLTGGKPVGNKGYYIEPTIFTDVKDDMLIAQDEIFGPVMALMKFKTIE
EAIQRANNTKYGLAAGIITKDLNVANTVSR
SIRAGIIWINCYFAFDRDCPYGGYKMSGFGRDFGMQGLYHYLHTKSVVTPLFNSPWL
>MdALDH2C11
MLKFADLIDQHVEELASLDTVNAGNLFGDGKIAGIPSVANSLRYYAGAADKIHGEVLKMSRDFHAYTLLE
PIGVVGHIIPWNFPSTL
FFTKVSPCLAAGCTMVVKPAEQTPLSAIYYAYLAKLAGVPDGVLNVITGFGQTAGAAISHHMDIDKVAFT

GSTEVGREVMQAAAKSNLKQVSLELGGKSP
LVIFDDADINMAVELALLGIFYNQGEICVASSRVYVQDGIYDEFVKKLKDEVKTWVVGDPFDPNVRQGPQ
VDKXQFXKVLTYIEXGKREGATLLTGGKPL
GDKGYYIEPTVFTDVKEXMLIAKDEIFGPVMALMKFKTIEEAIKKANNSXYGLAAGIITKDLNVANTVSR
SIRAGIIWINCYLAFDGDCPXGGYKMSGFG
RELGLQGLHKYLHTKSVVTPIYNSPWL
>MdALDH2C12
MLKFADLIDQHVEELASLDTVNAGNLFGDGKIAGIPSVANSLRYYAGAADKIHGEVLKMSRDFHAYTLLE
PIGVVGHIIPWNFPSTL
FFTKVSPCLAAGCTMVVKPAEQTPLSAIYYAYLAKLAGVPDGVLNVITGFGQTAGAAISHHMDIDKVXFT
GSTEVGREVMQAAAKSNLKQVSLELGGKSP
LVIFDDADINMAVELALLGIFYNQGEICVASSRVYVQDGIYDEFVKKLKDEVKTWVVGDPFDPNVRQGPQ
VDKXQFXKVLTYIEXGKREGATLLTGGKPL
GDKGYYIEPTVFTDVKEXMLIAKDEIFGPVMALMKFKTIEEAIKKANNSXYGLAAGIITKDLNVANTVSR
SIRAGIIWINCYLAFDGDCPYGGYKMSGFG
RELGLQGLHKYLHTKSVVTPIYNSPWL
>MdALDH3F1
MEAVREVEETLSKLRQTFKSGXTRSVAWRKKQLSALLELXNGNEEKIFKALDQDLGKHPVESYRDEVGVV
KKSLNHSLSNLEKWAAP
KKARLPLLLFPTSGEVLPEPLGVVLIFASWNFPISLALDPVIGAIAAGNVVVLKPSEQAPACSSFLAKTV
PQYLDGKAVEVIEGWVGRMVMSAAAKNLTP
VTLELGGKCPIVLDSLSNASDLKVAVKRIVGGKWGPCNGQACIGVDYMLVEEKFASMAIDLLKKTIKRFY
SESPKDSKCIARVVNKRHFERLRNLLKDPL
VADSIVYGGSLDEENLFIEPTILLDPPLNAAIMTEEIFGPLLPIITLKKIEESIEFINSKPKPLAIYAFT
KDENLRRRIVSETSSGTVVFNDILIQFVCD
SIPFGGVGQSGFGRYHGKYSFDTFSHEKAVMRANFIIEFGSRYPPWNDFKDKFIRFAYNFDYISLALLLL
GLKR
>MdALDH3F2
MEAVREVEETLSELRQTFKGGRTRSIAWRKKQLSSLLKLVNDNEENIFRALDQDLGKHPVESYRDEIGVV
KKSINHTLSNLEKWVAP
KKVGLPLPMFPTSGWLLPEPLGVVLVFASWNFPISLALDPMIGAIAAGNAVVLKPSEQAPASSSFLANTI
PQYMDSKAVKIVEGGAEISELLLQQKWDKI
FFTGSQQVGRIVMSAAAKNLTPVALELGGKCPIILDSISSPSDLKVAIQRIVGGKWGPCNGQACIGVDYM
LVEEKFAPTVIESLKKTIKRFYSESPKDSK
CLARVVNKRHFERLRNLFKDPPVAASIVHGGSLDEENLFMEPTILLDPPLNAAIMTEEIFGPLLPIITVK
KIEESIEFINSRPKPLAIXAFTKDANFRRR
VLSETSSGTVVFNDILIQFVCEALPFGGVGQSGFGRYHGKYSFDTFSHEKAVVQANFFIEFGSRYPPWND
FKNNIIRSAYNLDDLNLVLLLLGLKR
>MdALDH3H1
MATVEEQKRMYFDSKDALTMVEELRASYNSGKTRSYEWRESQLNNLLKVAELHEQEIVDALRSDLSKPEF
EAYVQEIAMLKNSCNLA
IKELRKWMKPEKVKTSIAVFPASAEIVSEPLGAILVISAWNYPFLLSLDPVVGAIAAGNVVVLKPSELAP
ATSSLLAKLVGEYMDSSCIRVVEGAIAETS
ALLEQKWDKICYTGNGRVGCIVMASAAKHLTPVLLELGGKSPVIVDSGINLQACVSPDYIVTTKDFAPKL
VESLKQELENFYGKNQLESKDLSRIVNPNH
FARLTKLLDEDKVSGKIVHGGEMDKTNLRIAPTILLNVPQDSLIMTEEIFGPLLPILTVDKLEDSFDLIN
SGAKPLAAYLFTNKKKLKEHFVNTVSAGGL

VINDTTVHLAVPSVPFGGVGESGMGAYHGKFSFDAFSHKKAVVYRGFVGDASIRYPPYTKGKLRILKALM
GGGILSIIRALFGWPEA
>MdALDH3H7
MGSEKPFDEEAASAVVKELRVTFASGKTRSYQWRVSQLECILKLLTENELEIVNALRSDLSKPELESKVY
EIAMLKNSCNLAIKELR
KWMKPEKVKTSIAVFPASAEIVSEPLGAILVISAWNYPFLLSLDPVVGAIAAGNVVVLKPSELAPATSSL
LAKLVGEYMDSSCIRVVEGAIAETSALLEQ
KWDKICYTGNGRVGCIVMASAAKHLTPVLLELGGKSPVIVDSGINLQACVSPDYIVTTKDFAPKLVESLK
QELENFYGKNQLESKDLSRIVNPNHFARLT
KLLDEDKVSGKIVHGGEMDKTNLRIAPTILLNVPQDSLIMTEEIFGPLLPILTVDKLEDSFDLINSGAKP
LAAYLFTNKKKLKEHFVNTVSAGGLVINDT
TVHLAVPSVPFGGVGESGMGAYHGKFSFDAFSHKKAVVYRGFVGDASIRYPPYTKGKLRILKALMGGGIL
SIIRALFGWPEA
>MdALDH3H8
MGSMAIEEEKQTAANPFDEEAASAVVKELRASFVSGKTRSYQWRVSQLKGIXKLITKKEREIVDALRSDL
SKPELESQVYVNLLVXV
VQYNTLLYADCSLSCFVTSLPXKNMHIWILLNKNKGCINDGVVALXYLIDILHIYYCGYGSLLTPYKMER
VGRNLNCGFXGPPEPDVSAPEQFRLKNWSP
HLISMLXNSCKLAIKELKKWMKPEKVKTSIAVFPASAEIVSEPLGAILVISAWNYPFLLSLDPVVGAIAS
GNVVVLKPSELAPATSSLLAKLLXEYMDSS
CIRVVEGAVAETSALLEQKWDKICYTGNGRVGRIVMASAAKHLTPVLLELGGKSPVIVDSGINLQVACRR
IIVGKWGCNNGQACISPDYIITTKDFAPKL
VDSLKQELENFYGKNQLESKDLSRIVNPHHFARLTKLLDEDKVSGKIVHGGETDKTNLRIAPTILLDVPQ
DSLIMSEEIFGPLLPILTVEKMEDSFDLIN
SGTKPLAAYLFTNKKKLKEHFVNTVSAGGLVVNDTTIHLAVPSLPFGGVGESGMGAYHGKFSFDAFSHKK
AVVYRGFFGDASIRYPPYTKGKLRILKALI
SGGISSIIRALFGWPKA
>MdALDH3I1
MKGLCIDLFKNYSGMVPNQFGFGGYCWDRDWDSEQQSFHSPVSEKKKQLSKPEAFLFLQTYMLFLLVEEA
KQTFNPEKAGLLVKELR
KSFNSGRTKSYEWRRXQLENIAKMLEEKEKEITEALYKDLSKPEIEAFISEIVQXRSSCNEALKELKHWM
VPQKVXTSITTYPSSAEIVSEPLGVVLVIS
TWNFPFLLSLDPVIGAISAGNAVVLKPSEIAPATSSLLANLVEEYLDNSAIKVVEGAVPETTALLEQKWD
KILYTGSARVGRIVMAAAAKHLTPVILELG
GKSPAVVDSDVNLEVAVRRIMAGKWALNNGQACIGVDYIITTKDFAPKLIEALKYGLEQFFGKDPMNSKD
ISRIVSSTQFTRLAKLLDEDKVSNKIVLGG
QMDEKQLKIAPTILLDVPEDAQIMQEEIFGPLMPIVTVEKIEDSFSVINSKPKPLAVYAFTNNEQLKKGF
VDNVSSGGMLINDTVLHVSISGLPFGGVGE
SGMGSYHGKFSFDGFSHKKAVLYRGFAGDSDLRYPPYTPEKQRLFRAVINGDIFTIILALIGWSK
>MdALDH3I2
MFRXELDPSPDPRDLSQLSNAFGPTKPCSATLSAVTAEVEEAKQTFNPEKAGLLVKELRKSFNSGRTKSY
EWRRXQLENIAKMLEEK
EKEITEALYKDLSKPEIEAFISEIVQXRSSCNEALKELKHWMVPQKVXTSITTYPSSAEIVSEPLGVVLV
ISTWNFPFLLSLDPVIGAISAGNAVVLKPS
EIAPATSSLLANLVEEYLDNSAIKVVEGAVPETTALLEQKWDKILYTGSARVGRIVMAAAAKHLTPVILE
LGGKSPAVVDSDVNLEVAVRRIMAGKWALN
NGQACIGVDYIITTKDFAPKLIEALKYGLEQFFGKDPMNSKDISRIVSSTQFTRLAKLLDEDKVSNKIVL
GGQMDEKQLKIAPTILLDVPEDAQIMQEEI

FGPLMPIVTVEKIEDSFSVINSKPKPLAVYAFTNNEQLKKGFVDNVSSGGMLINDTVLHVSISGLPFGGV
GESGMGSYHGKFSFDGFSHKKAVLYRGFAG
DSDLRYPPYTPEKQRLFRAVINGDIFTIILALIGWSK
>MdALDH5F1
MTFRASRMAARSFRLLSHLPSMHAPTPLLSRQLQYPHALNREQHYMDAKSFVSQLNGAGLLRSQGLIGGK
WSDAYDGSIIKVXNPAT
GEVITTVPCMGQKETNDAISSAYGAFSSWSKLAASERSKYLRKWYDLLISHKEELGQLITLEQGKPLREA
IGEVNYGAAFIEFYAEEAKRVYGDIIPATL
ADRRLFVLKQPIGVVGAVTPWNFPLAMITRKVGPALACGCTVVIKPSEFTPLTALAAAELALQAGIPPGV
VNVVMGSASEIGDALLASPQVRKITFTGST
TVGKKLMAGAAGTVKKVSLELGGNAPCIVFDDADLDVAVKGALAAKFRNSGQTCVCANRIIVQEGIYEKF
MDAFVKAVQNLQVGNGFSEGVVQGPLINEA
AVQKVESFVQDAVSKGAQVVVGAKRHSLGMTFYEPTVLRDVKSDMLISREEVFGPVAPLLRFKTEEEAIR
IANDTNAGLAAYIFTNSIQRSWRVSEALEY
GLVGVNEGLISTEVAPFGGVKQSGLGREGSKYGMDEYLEVKYVCMGNMSSN
>MdALDH5F2
MTFRASSMAGRSFKFLSHLPYMHAPTPLSRQLQHPHTLNCEQAALLISMDAKSFVSQLNGAGLLRSQGLI
GGRWSDAYDGNTIKVHN
PATGEVITTVPCMGQKETNDAISSAYELALILNLVFHFPPHPSRYDLIISHKEELGQLITLEQGKPLKEA
IGEVNYGAAFIEFYAEEAKRVYGDIIPATL
ADRRLFVLKQPVGVVGAVSPWNFPLAMITRKVGPALACGCTVVIKPSEYTPLTALAAAELALQAGIPPGV
VNVVMGNASEIGDALLASPQVRKITFTGST
AVGKKLMAGAAGTVKRVSLELGGNAPCIVFDDADLDVAVKGTLAAKFRNSGQTCVCANRIIVQEGIYEKF
MDAFVKAVQNLQVGDGFSEGVVQGPLINEA
AVQKVESFVQDALSKGAQVVLGAKRHSLGMTFYEPTVLRDVKSDMLISREEVFGPVAPLLRFKTEEEAIR
IANDTNAGSFFELPFHTLIPHPPLVPREPG
ELFHSNFYPAAHVHIISHTFTGLAAYIFTNSIQRSWRVSEALEYGLVGVNEGLVSTEVAPFGGVKQSGLG
REGSKYGMDEYLELTKHVKMSRCISRFKCC
KMSVRTYLVDEIIRHYIQYSPTNDNL
>MdALDH6B7
METQSQTEVTXQNKMLPPQSGTFEDREDLVKYVRDFGASQGYVVTIKKSRKDRRVILGCDRGGVYRNRRK
IDESKRKRKASSRLINC
PFEAIGKKEDDLWVLTVKNGEHNHEALKDMSEHPYSRRFTEEEVRQIKQMTEAGVKPRQVLKALKQINPE
LQSTPRHLYNLKAKIRQGNLSEKSFKSWRP
DRSALVSTIATASGESLKQNNQPLKVPNFIGGKFVDSQGCSVVDVLNPATQEVVSHVPLTTYEEFKAAVS
SAKQAFPSWKNTPITTRQRIMFKLQELIRR
DIDKLAMNITIEQGKTLKGAESDVLRGLEVVEHACGMATLQIGEFVPNASNGIDTYCIREPLGVCAGICP
FNFPAMIPLWMFPIAVTCGNTFVLKPCEKN
PGASMILAALAKEAGLPDGVLNIVHGTHFIADKFLLLENQCEQDTINYICDDDDIKAVSLVGSNTAGMHX
HARAVAGGKLVQSSIGGKNHAIIMPDASMD
ATLNSLVTAGFGAAGQRCMALNTAVFIGGSRPWEGELVDRAKALKVNVGTDPSADLGPVITKELFTCYLN
SQVKDSICRLVQTSVESGTRLILDGRNLTV
PGYENGNFIGPTILCDVTTNMDCFKTWLITILSERRVEPKTWHILDRTVSCYEQEEIFGPVLLCMQAASL
EEAISIVNRNRFGNGASIFTTSGIAARKFQ
NEVEAGLVGINVPVPIPLPFSSSNGSKASFGSNLNFSGKAGVQFYTQIKTVAQQWKDXPSLEVSLGRPPS
SETEMTGRGVSSGLPSTSERDSPSQRVSAD
MHSESESDSPSHGAPLSITPTSEADRPNPGVSSVSTTYRNLSSQGIPLXIPATSERDLSSADISLALHPE
SEREIPSQVVSLRPSQSSERIXMPQMSHWT

ETSTSQRTENLPQTSHWMXTSRSTSLRTENLPPTSQWMETSTPTSQRTENIPPNSERNHVPXSQRNGSTA
LTSQRIDTTMGLTSERAYVPTSHDNMVPVS
HRNDGISLTSQRIDATLHPTSERVYMLAGSHLNENTIFSTSERLYMPETSHWHDHMGSTSQRTESTLHPN
SERIYVSIASQRNDDLAAASHRASDAVPSS
SERLY
>MdALDH6B8
ATQEIVSQVPLTTTEEFKAAVSAAKKAFQSWRNTPVTARQRVMFKLQELIRRDIDKLALNITTEQGKTLK
DAHGDVFRGLEVVEHAC
GTATLQMGEYVSNVSNGIDTYSVREPLGVCAGICPFNFPAMIPLWMFPVAVTCGNTFILKPSEKDPGASV
ILAELAMEAGLPDGVLNIVHGTNDIVNAIC
DDDDIRAVSFVGSNTAGMHIYSRAAAKGKRVQSNMGAKNHAIIMPDASAEATLNALVAAGFGAAGQRCMA
LSTVVFVGDSKSWENKLVEAAKGLKVSAGT
EPDADLGPVISRQAKERICKLIQSGVESGAKLLLDGRSIXVPGYEHGNFIGPTIISNVTADMECYKEEIF
GPVLLCMEADSLDEAISIVNRNKYGNGASI
FTTSGVAARKFQTEIEAGQVGINVPIPVPLPFFSFTGNKASFAGDLNFYGCSAAYITRFAPYSGASYRLR
PVSAVSNSNAARTPLLTLRLLCLENMNALG
GGGYGGLWGWNSSSPRKPKQPRRRPFDSKSQSSSNSNSVDATGRARHWFPLMQAATAGSLALTGDTIAQL
TQRWRKAEAENQQDVKRALLSDHDWLRALR
MTSYGFLLYGPGSYAWYQYLDHSLPAKTVENLLLKVLLNQIVLGPCVIAVVFAWNNLWQGKVSQLPGKYQ
RDALPTLLYGWFGSLTSDRPQV
>MdALDH7B5
MGFAKKEHEFLSAIGLAPENPGGFINGKWKASGPVISTVSPSNNQEIAKVTEVSMEEYEEGLRSCNDAAK
TWKSLPAPKRGEIVRQI
GDALREKLQHLGKLVSLEMGKILAEGIGEVQEVIDMCDFAVGLSRQLNGSIIPSERPDHMMFEVWNPLGI
VGVITAFNFPCAVLGWNACIALVCGNCVVW
KGAPTTPLVTIAVTKLIAEVLEKNNLPAAIFTAFCGGAEIGEAIAKDTRIPLVSFTGSSKVGAKVQQIVT
ERFGKCLLELSGNNALIVMDDADVGLAVRS
IFFAAVGTAGQRCTTCRRLYLHESIYQNVLDKLVGLYNQVKIGDPLEEGTLVGPVHTKASRENFEKGIST
IKSQGGKILTGGSVIESDGNFVQPTIVEIA
SNASVVKEELFGPVLYVMKFKTLEEAIALNNSVPQGLSSSIFTSKPNTIFKWIGSYSVHKAPALRRVWER
PHGSDCGIVNVNIPTNGAEIGGAFGGEKAT
GGGREAGSDSWKQYMRRSTCTINYGTELPLAQGINFG
>MdALDH7B8
MGFAKKEHEFLSAIGLAPENLGGFINGNWKASGPVISTVTPSNNQEIAKVTEASMEDYEEGLRSCNEAVK
TWKTLPAPKRGEIVRQI
GDALREKLQHLGKLVSLEMGKILAEGIGEVQEVIDMCDYAVGLSRQLNGSIIPSEPNNDLHFYTPQVWNP
LGIVGVITAFNFPCAVLGWNACIALVCGNC
VVWKGAPTTPLVTIAVTKLIAEVLKRNNLPGAIFTAFCGGAEIGEAIAKDTRIPLVSFTGSSKVGSKVQQ
IVTERFGRCLLELSGNNALIVMDDADVGLA
VRSIFFAAVGTAGQRCTTCRRLGGKILTGGSVIESDGNFVQPTIVEIASNASVVKEELFGPVLYVMKFKT
LEEAIELNNSVPQGLSSSIFTSKPNTIFKW
IGPHGSDCGIVNVNIPTNGAEIGGAFGGEKATGGGREAGSDSWKQYMRRSTCTINYGTELPLAQVYIKRA
QQMIVVAESPFRPIAAKVDRPNECGRYLLR
SLTTCIQPTLAGGLTTTVHPLRGSERLTDVFQPSLATII
>MdALDH10A8
MAIQIPSRQLFIDGEWREPVLKKRIPIINPATEQIIGDIPAATAEDVEIAVEAARKALARNKGRDWALAP
GAVRAKYLRAIAAKIAE
RKSEIAKLEAIDCGKPLDEAAWDXDDVSGCFEYYADLAEGLDAQQKTPISLPMEQFKSHVLKEPIGVVGL

ITPWNYPLLMATWKVAPALAAGCAAILKPS
ELASVTCLELADVCREVGLPPGVLNILTGLGHEAGAPLASHPHVDKIAFTGSTMTGSKIMTAAAQLVKPV
SLELGGKXPIVVFDDVDIDKAAEWTAFGIF
WTNGQICSATSRLIIHENIAAEFLDRLVQWCKNIKIADPLEEGCRLGPVVSGGQYXKILKFIATAKSEGA
RVLSGGARPEHLKKGFFIEPTIITDVTTSM
QIWREEVFGPVLCVKTFSSEDEALELANDSHYGLGAAVISKDLERCERVSKALQAGIVWINCSQPCFCQA
PWGGNKRSGFGRELGKWGLDNYLTVKQVTE
YVSDDPWGWYKSPSKL
>MdALDH10A9
MAVRVPRRQLFIDGEWREPSLKKRIPVINPATEETIGLSLPRLSMLFYILIVEILICGDIPAATAQDVEI
AVXAARKALARDKAKDW
SSAPGAVRAKYLRAIASKIKERKSELAKLEAMDSGKPLDETTSDIGEVASCFEYYAGLAEGLDAKQKTPV
SXSTEKFKTHVLKEPVGVVGLITAWNYPLL
MAAWKVAPALAAGCAAVLKPSELASIWYVDFSRLCISFVLSINSRTCLELAEVCINVGLPPGVLNIVTGL
GNEAGAILTSHPNVDKPPGCNCYMXNXQQI
AFTGSTITGSKIMAAGAPXIKPVALELGGKSPIVVFEDVDIDKAVEWTCFGIFLTNGQICSATSRLIVHE
SIAAEFMDRLVKWSKNIKISDPMEQGCRLG
PVVSKGQYEKILKFISTAKSEGAKVVYGGDRPEHLKKGFFIEPTIITDVTPSMQIWREEVFGPVLCVKTF
SSEEEALELANDTHYGLGAAVMSKDLERCE
RFSKGLQAGIVWINSSQPSFIQAPWGGNKRSGFGRELGEWYMDFLTXWKKGNGXIFVSQQGTGALLERQA
GHIPAATAEDVELAVEAARRALSRNKGRDW
ASAPGAVRAKYLRAIAAKIGERKPEIAKLEAIDCGKPLDEAAWDIDDVSGCFEYYAELAEGLDAQQKAPI
SLPMEQFKXHVLKEPIGVVGLITPWTCLEL
ADVCREVGLPPGVLNILTGLGHEAGAPLVSHPHVDKIAFTGSTMTGSKIMTAAAQLVKPVSLELGGKSPI
VVFDDVDIDKAAEWTAFGCFWTNGQICSAT
SRLILHENIATEFLDRLXKWCKNIKIADPLEEGCRLGPVVSGGQYEKILKSIETAKSEGARVLSGGXRPE
HLKKGFFIEPTIITDVTTSMQIWREEVFGP
VLCVKTFSSEDEALELANDTHYGLGAAVISKDLERCDRFSKGLQAGIVWINCSQPCFCQAPWGGNKRSGF
GRELGKWGLDNYLTVKQVTEYVSDDPWGWY
TSPSKL
>MdALDH11A3
MAGSGLFAEIVDGEAYKYYADGEWKKSSSGKLVPIINPTTRKVHYKVQACTQEEVNKVMETAKIAQKTWA
KTPLWKRAELLHKAASI
LKEHKAPIGESLXKEIAKPAKDAITEVVRSGDLVSYCAEEGVRILGEGKFLVSDSFPGNDRTKYCLTSKI
PLGVVLAIPPFNYPVNLAVSKIAPALIAGN
ALVLKPPTQGAVSCLHMVHCFHLAGFPKGLISCVTGKGSEIGDFLTMHPGVDCISFTGGDTGXAISKKAG
MIPLQMELGGKDACIVLEDADLDLVAANII
KGGFSYSGQRCTAVKVVLVMESVADALVAKVNARVAKLTVGPPEENSDITPVVSESSANFIEGLVVDAKQ
KGATFCQEYKREGNLIWPLLLDNVRPDMRI
AWEEPFGPVLPVIRITTVEEGIHHCNASNFGLQGCVFTKDINKAILIGDAMETGTVQINSAPARGPDHFP
FQGIKDSGIGSQGITNSINMMTKIKTTVIN
LPTPSYTMG
>MdALDH11A4
MAGSGLFAEIVDGEAYKYYADGEWKKSSSGKLVPIINPTTRKVHYKVQACTQEEVNKVMETAKIAQKTWA
KTPLWKRAELLHKAASI
LKEHKAPIGESLXKEIAKPAKDAITEVVRSGDLVSYCAEEGVRILGEGKFLVSDSFPGNDRTKYCLTSKI
PLGVVLAIPPFNYPVNLAVSKIAPALIAGN
ALVLKPPTQGAVSCLHMVHCFHXAGFPKGLISCVTGKGSEIGDFLTMHPGVDCISFTGGDTGXAISKKAG

MIPLQMELGGKDACIVLEDADLDLVAANII
KGGFSYSGQRCTAVKVVLVMESVADALVAKVNARVAKLTVGPPEENSDITPVVSESSANFIEGLVVDAKQ
KGATFCQEYKREGNLIWPLLLDNVRPDMRI
AWEEPFGPVLPVIRITTVEEGIHHCNASNFGLQGCVFTKDINKAILIGDAMETGTVQINSAPARGPDHFP
FQGIKDSGIGSQGITNSINMMTKIKTTPCS
REMQEMNSLLREMQEMNSLLNSSASSSQLSLQDHHHLQQQPHSHSQMHHQIPTPSASHFDSATHDDFLEQ
MLSTLGPSWASAADDAPPPLSSNPDNVVFS
YDDSATLAAKFRSQQISAGSGANKSASASAAAAXAMMLQHQLMMSRSGAADSGFGPTGLSLGNNGDFGRS
NNDVGDGSSFKSPNQVGGQLQAQNYGGAGA
AXNQAPAGGSAGAAPVQPRPRVRARRGQATDPHSIAERLRRERIAERMKALQELVPNANKADKASMLDEI
IDYVKFLQLQVKVLSMSRLGGAAAVAPLVA
DMSSEGGGDCIQASANGGTRGRSSNGNQTASSSNDNSMTVTEHQVAKLMEKDMGSAMQYLQGKGLCLMPI
SLATAISTATCHSRNPLLHNNSNNHQVVPS
NGGDGPSSPSMSVLTVQSATMGNGGVDGSVKDATSVSKP
>MdALDH11A5
MAQLFSSFXVLADTNGVVKLSFQQVGGKRGVPTSSYLPNRSVLRIRSCKTNSYSEILDGEAYKYYGDGEW
KKSSSGKLVPIINPTTR
KVQYKVQACTQEEVNKVMETAKIAQKAWAKTPLWKRAELLHRAASILKEHKAPIGESLVKEIAKPAKDAI
TEVVRSGDLVSYCAEEGVRILGEGKFLVSD
SFPGNDRTKYCLTSKIPLGVVLAIPPFNYPVNLAVSKIAPALIAGNALVLKPPTQGAVSCLHMVHCFHLA
GFPKGLISCVTGKGSEIGDFLTMHPGVNCI
SFTGGDTGIAISKKAGMIPLQMELGGKDACIVLEDADLDLVAANIIKGGFSYSGQRCTAVKVVLVMESVA
DALVAKVNARVAKLTVGPPEDNSDITPVVS
ESSANFIEGLVVDAKQKGATFCQEYKRDGNLIWPLLLDNVRPDMRIAWEEPFGPVLPVIRITTVEEGIHH
CNASNFGLQGCVFTKDINKAMLIGDAMETG
TVQINSAPARGPDHFPFQGIKDSGIGSQGITNSINMMTKIKTTVINLPTPSYTMAAHLVASFVFKEGGXH
SCNEFKLVWLFNTXTXDPCSREMQEMNSLL
NASASASQLSLQDHHHLQHHQQQSHSHSSQMHPQIPNPSASHFDSATHDDFLEQMLSTLGPSWASAADD
APPPSSSNPDNVVFSYDDSATLASKFRSQQ
ISAGSGANKSASASAAAAAAMMLQHQLMMSRSGAADSGFSPMGLSLGNNGDFDRSNNDVGDVSSFKSPNT
VVRRSAASAELRRCRGGDEPSSGGWVGWGC
AGPTQAQSQGEKRSSH
>MdALDH12A1
MNRILVSRQLRARAPQTALSWFTSLNISRSIHGVPFATVEVEEISGSQPAEVLNLVQGKWKNTNRWNTIL
DPLNGEPFIRVCEVDET
GIQPFVESLSKCPKHGLHNPFKAPERYLMLGDISAKAGHMLSLPKVSDFFTRLIQRVAPKSYQQASGEVY
VTQKFLENFSSDQVRFLARSFGVPGNHLGQ
QSHGFRWPYGPVAIITPFNFPLEIPVLQLMGALYMGNKPVLKVDSKVSIVMEQMMRLLHYCGLPTEDVDF
INSDGQTMNKLLLEGNPXMTLFTGSSRVAD
KLAGDLKGRVKLEDAGFDWKILGPDVHEEDYVAWVCDQDAYACSGQKCSAQSMLFIHENWSKTSLLSKMK
DLAERRNLEDLTIGPVLTFTTEAMLEHKNK
LLQIPGSKVLFGGEPLTNHSIPPVYGAIEPTAIFVPLEEILKDKNYELVTREIFGPFQIVTDYKNDQLPL
VLDALERMHAHLTAAVVSNDPLFLQARFDL
FIGNTVNGTTYAGLRARTTGAPQNHWFGPAGDPRGAGIGTPEAIKLVWSCHREIIYDVGPVPKQWQTPPS
T
>MdALDH12B1
MXYFFSVQGKWKNTDRWNTILDPLNGEPFIRVCEVDETGIQPFVESLSKCPKHGLHNPFKAPERFVSDFF
TRLIQRVAPKSYQQASG

EVYVTQKFLENFSADQVRFLARSFGVPGNHLGQQSHGFRWPYGPVCIVMEQMMRLLHYCGLPTEDVDFIN
SDGKTMNKLLXEGNPRMTLFTGSSRVADKL
AGDLKGRVKLEDAGFDWKILGPDVQEEDYVAWVCDQDAYACSGQKCSAQSMLFMHENWSKTSILSKMKDL
AERRNLEDLTIGPVLTFTTEAMLEHKNKLL
QIPGSKLLFGGEPLTDHSIPPVYGAIKPTAVYVPLEEILKEKNYELVTREIFGPFQVTKKRLATKIVTDY
KNDQLPLVLDALERMHAHLTAAAQYQKIGP
XLAKTLFPLSCPRTKTLPPSAVGTCNFFPISALSAADDATLGKLCDSSAMSLLEVIARASAKSKTQSAPS
DYPIVLDPEPIFENLKPKFDDPNASAAAIP
IDGWKISQTDSVLIDSGKKFFTKLQKKLKNPTNFTKVEFLGILNPFLENIWEKRKAGESIGVDSSNDGYS
RVLIEKVGNLIGKDVAGLVLDSCVVLEIWD
LVGALIANGVFPNSCYQHLVPKLVSKRRSELLCLCVKHASDLGSSELLLNLKYFLDPPKDSYTSSMDVRK
EWESRALAAAQKAGDQSLTDKKLRVAKDAA
VLLMVAYDGFSSAELCLHYPLASQNLDEVMFSAAISKLSGKEMKSLLRSMRGFLRHHQRRA
>MdALDH18B1
MEEVDSSRGFLKDVKRLVLKVGTAVVTRNDGRIALGRLGALCEQLKELNSQGYEIILVSSGAVGLGRQRL
RYRKLVNSSFADLQKPQ
VELDGKACAAVGQNCLMALYDTLFSQLDVSSAQLLVTDSDFRDRDFRKQLGETMKSLLSLRVIPIFNEND
AVSTRKAPYEDSSGIFWDNDSLAALLALEL
KADLLILLSDVDGLYSGPPSDPRSKLIHTYVKEKHQTEITFGDKSRVGRGGMTAKVKAAVNAAYAGIPVV
ITSGFAAGNISKVLQGQRIGTLFHQDANLW
TPVKEIDARGMAVAARESSRRLQAMTSEDRKKILLDVADALEANVKLINIENEADVSAAQRAGYEKSLIS
RLALKPGKITSLAKSIRVLANMEDPIGRVL
KKTELADGLVLEKTSSPLGVLLIVFESRPEALVQIASLAIRSGNGLLLKGGKEAKRSNAILHKIITEAIP
ESVGGKLIGLVTSREEIPDLLKHDDMIDLV
IPRGSNKLVSQVKNSTKIPVLGHADGICHVYIDKSANMDMAKRIVLDAKIDYPAACNAMETLLVHNDLKS
TAAFNDLVVELRTAGVTLYGGPRASVLLRI
PEAHSFHHEYSSMACTVEFVDDVHAAIDHIHEHGSAHTDCVIAEDQEAVDAFLGKVDSAAVFHNASPRFC
DGARFGLGAEVGISTSRIHARGPVGVEGLL
TTRWILRGHGQIVDGDKGVTYTHKDLPIEP
>MdALDH18B2
MEEVDHSRAFLKDVKRLVIKVGTAVVTRNDGRIALGRLGALCEQLKELNSQGYEIILVSSGAVGLGRQRL
RYRKLVNSSFADLQKPQ
VELDGKACAAVGQNCLMALYDTLFSQLDVSSAQLLVTDSDFRDRDFRRQLGETMKSLLSLKVIPIFNEND
AVSTRRAPYEDSSGIFWDNDSLAALLALEL
KADLLILLSDVDGLYSGPPSDQRSKLIHTYIKEKHQTEITFGDKSRVGRGGMTAKVKAAVNAAYAGIPVV
ITSGFAAGNIFKVLQGQRIGTLFHQDAHLW
APVKEIDARGMAVAARESSRRLQAMTSEERKKILLDVADALETNEKLINVENEADVSEAQRAGYEKSLIA
RLALKPGKITSLAKSVRVLANMEDPIGRVL
KKTELADGLVLEKTSSPLGVLLIVFESRPEALVQIASLAIRSGNGLLLKGGKEAKRSNAILHKIITEAIP
ESVSGRLIGLVTSREEIPDLLKHDDVIDLV
IPRGSNKLVSQVKNSTKIPVLGHADGICHVYIDKSANMDMAKRIVLDAKIDYPAACNAMETLLVHNDLKS
TTAFNDLVVELCIAGVTLYGGPRASALLGI
PEAHSFHHEYSSMACTVEFVDDVHAAIGHIHEHGSAHTDCIIAEDQEVVDAFLGQVDSAAVFHNASPRFC
DGARFGLGAEVGISTSRIHARGPVGVEGLL
TTRWILRGNGQIVDGDKEITYTHKGLPIEP
>MdALDH18B3
MSSKTPLGKQTMDSVDPTRAFVKNVKRIVVKVGTAVVTRGDGRLALGRLGALCEQLKDINSQGYEVILVT
SGAVGLGRQRLRFRRLA

NSSFADLQNPQNDFDGKACAAVGQSSLMALYDTMFSQLDVTSSQLLVTDSVFRDEAFRKQLSETVKSLLK
LRVVPIFNENDAVSTRKAPYEDSSGIFWDN
DSLAGLLALELKADLLVLLSDVEGLYSGPPTDPKSKLIHTYVKEKHQGEITFGDKSRVGRGGMTAKVNAA
VCAAYGGIPVVITSGYADNIKKVLRGDRIG
TVFHQDAHLWTLVKEEGARDMAVAARESSRQLRALGSQERRKILLDVADALEANEGEILAANEADVAAAQ
DDRYEKALISRLALKPGKISALAKSIRKLA
DMEEPIGRVLKKTELAEGLVLEKLSCPLGVLLVVFESRPDALVQIASLAIQSRNGLLLKGGKEAKRSNAI
LHKVITSAIPEPVGGKLIGLVNTRDEIPDL
LKLDNVIDLVIPRGSNKLVSQIKESTKIPVLGHADGICHVYVDKSANLDMAKRIVLDAKVDYPAACNAME
TLLVHKDLLSGDGGFKQIISELQVSVNDRK
SVADVTLYGGPRAATALNLPETNSFHYEYSSLACSVEIVDDVNAAIDHIHEHGSAHTDCIVAEDRQVAET
FLTQVDSAAVFHNASTRFCDGARFGLGAEV
GISTSRIHARGPVGVEGLLTTRWVLRGNGQIVDADRGVEYTHKDLLNS
>MdALDH18B4
MDSVDPTRAFIKNVKRIVVKVGTAVVTRGDGRLALGRLGALCEQLKDINSQGYEVILVTSGAVGLGRQRL
RYRRLANSSFADLQNPQ
NDFDGKACAAVGQISDLPLVTLTILAFFXLQLDVTSSQLLVTDSVFRDEAFRKQLSETVKSLLKLRVIPI
FNENDAVSTRKAPYEDSSGIFWDNDSLAGL
LALELKADLLVLLSDVEGLYSGPPTDPKSKLIHTYVKEKHQGEITFGDKSRVGRGGMTAKVNAAVCAAYG
GXPVVITSGYDXDNIKKVLRGDRIGTVFHQ
DAHLWTVVKEEGARDMAVAAQEASRQFRALGSQERRKILLDVADALEANEGEIIAANEADVATAQEVGYE
KALISRLALKPGKVSLQISALAKSVRKLAD
MEEPIGRVLKKTELAEGLVLEKLSCPLGVLLVVFESRPDALVQFLDQFTNTFXFQMVQQIAALAIQSRNG
LLLKGGKEAKRSNAILHKVITSAIPEPVGG
KLIGLVNTXDEIPDLLKLXDVIDLVIPRGSNKLVSKIKESTKIPVLGHADGICHVYVDKSANLDMAKRIV
LDAKVDYPAACNAMETLLVHKDLLSDGGFK
QIISELKDSGVSLYGGPRAATALHLPETDSFHYEYSSLACSVEIVDDVNAAIDHIHEHGSAHTDCIVTED
GQVAEVFLTQVDSFTQKFPNVTHLDGCTVC
INSAAVFHNVSTRFCDGARFGLGAEVGISTSRIHARGPVGVEGLXTTRWILRGNGQIVDADRGVEYTHKD
LLSX
>MdALDH22A1
MAFWWALFVLGFAYAVCRFLLMLIPPNVPSIEVDASDVLDDGNQTQENSFIYVPPRGRTPQAETKVQCYE
PATMKYLGYFPALTPIE
VKERVAQARKAQKVWAKSSFKQRRQFLRILLKYIIEHQELICEISSRDTGKTMVDASLGEIMTTCEKITW
LLSEGERWLKPEYRCCGRSMLHKRSKVEFH
PLGVVGAIVSWNYPFHNIFNPMLAAVFSGNSIVIKVSEHASWSGCFYLRIIQSALAAVGAPENLVDVVTG
FAETGEALVSSVDKIIFVGSPGVGKMIMKS
AAENLTPVTLELGGKDAFIVCEDVDVEHVSQIAVRAVLQSSGQNCAGAERFYVHEKIYSSFVSQVSKIVK
SVSAGPPLTGRYDMGAICLQEHSERLQYLV
NDALDKGAKFVVRGSVGHIGEGAVDQFFPPTVIENVNHTMKLMQEEAFGPIMPIMKFSTDEEAVKLANDS
KYGLGCAVFSGSQRRAKEIASQIHCGVAAI
NDFASTYMCQSLPFGGVKDSGFGRFAGVEGLRACCLVKSVVEDRWWPYIKTKIPKPIQYPVAENGFDFQE
SLVEALYGMNIWERLRALVNVLKILREQNS
PGNNKRRDD
>MdALDH22A2
MAFWWALFVLGFAYAVCRFLLLLIPHNVPSIDVDASDVLDDGNQTQENSFIYVSFSKVPPRGRTPQAETK
VQCYEPATMKYLGYFPA
LTPVEVKKRVAQARKAQKAWAKSSFKQRRQFLRILLKYIIEHQELICEISSRDTGKTMVDASLGEIMTTC

EKITWLLSEGERWLKPEYRCCGRSMLHKRS
KVEFHPLGVVGAIVSWNYPFHNIFNPMLAAVFSGNGIVIKASEHATWSGCFYLRIIQSALAAVGAPENLV
DVVTGFAETGEALVSSVDKIIFVGSPGVGK
MIMRRAAENLTPVTLELGGKDAFIVCEDVDVEHVSQIAVRAVLQSSGQNCAGAERFYVHEKIYSSFVSQV
SKIVKSVSAGPPLTGKYDMGAICLQEHSER
LQNLVNDALDKGAKFAVRGSVGHIGEGAVDQFFPPTVIENVNHTMKLMQEEAFGPIMPIMKFSTDEEAVK
LANDSKYGLGCAVFSGSQRRAKEIASQIHC
GVAAINDFASTYMCQMSRTLTNFQSLPFGGVKDSGFGRFAGVEGLRACCLVKSVVEDRWWPYIKTKIPKP
IQYPVAENGFEFQESLVEALYGMNIWDRLR
ALVNVLKIVTEQNSPANNKRRDD
